# *N*-Oxide Insertion into LDA Dimeric Aggregates for Azomethine Ylide Formation: Explicit Solvation in Quantum Mechanical Treatment of Polarized Intermediates

**DOI:** 10.1021/acs.joc.4c03090

**Published:** 2025-03-01

**Authors:** Martin J. Neal, Eric J. Chartier, Aiden M. Lane, Sarah L. Hejnosz, Luke T. Jesikiewicz, Peng Liu, Jeffrey J. Rohde, Paul Lummis, Douglas J. Fox, Jeffrey D. Evanseck, Thomas D. Montgomery

**Affiliations:** † Department of Chemistry and Biochemistry, Center for Computational Sciences, Duquesne University, 600 Forbes Avenue, Pittsburgh, Pennsylvania 15282, United States; ‡ Department of Chemistry, University of Pittsburgh, 219 Parkman Avenue, Pittsburgh, Pennsylvania 15260, United States; § Department of Mathematics and Physical Sciences, Franciscan University of Steubenville, 1235 University Boulevard, Steubenville, Ohio 43952, United States; ∥ Gaussian Inc., 340 Quinnipiac St #40, Wallingford, Connecticut 06492, United States

## Abstract

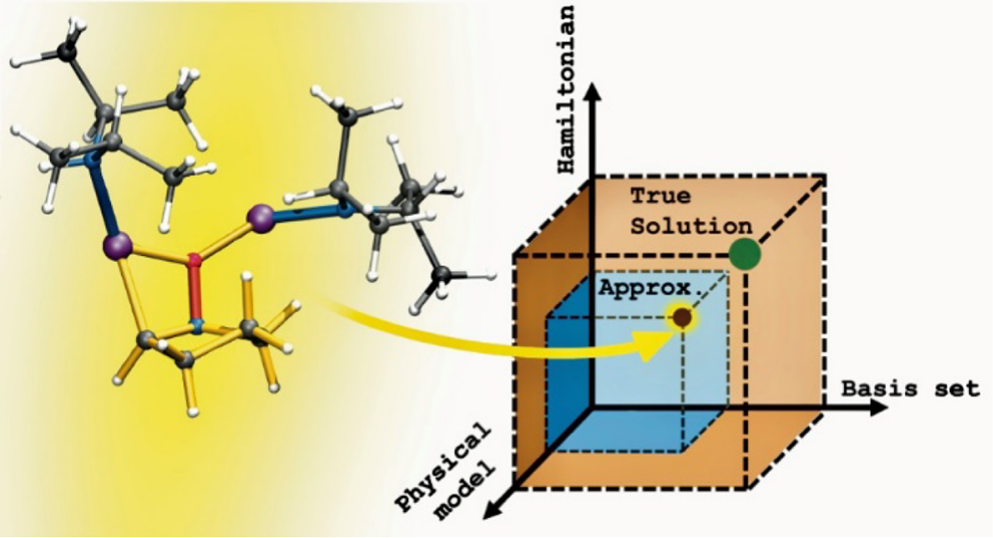

The physical model used to compute natural phenomena is crucial for accurate structural and mechanism elucidation. Specifically, we examine the mechanistic consequences of an explicit LDA dimer, THF, and *N*-oxide aggregate formation at the rate-limiting step for two competing reaction pathways involving nitrogen–oxygen dissociation and alpha-hydrogen deprotonation for azomethine ylide formation. We compute the free energies of activation using the M06-2x, B3LYP, and HCTH407 functionals and second-order Møller–Plesset perturbation theory with Dunning’s correlation consistent basis sets cc-pV[D,T]Z, and corrected entropy by using Whitesides’ free volume theory. Our discrete-continuum approach uses Tomasi’s polarizable continuum model to complement the quantum system by incorporating bulk solvent effects. Building off the LDA aggregation work developed by Collum and coworkers, we demonstrate that the explicit inclusion of solvent can have a profound impact on the predicted free energy barriers and alignment with experimental product distributions. In the polarized *N-*oxide system, the use of a more sophisticated and balanced model of the reaction mechanism underscores the importance of explicit solvent and the correct pattern of aggregation. Our results identify a unique aggregate that incorporates the *N*-oxide, THF, and LDA for azomethine ylide formation, which suggests a third dimension to the John Pople diagram to enhance the accuracy through model sophistication.

## 1 Introduction

Solvation effects are central in organic synthesis,^1^ where it has been well documented that the judicious choice of solvent can significantly alter the kinetics, equilibrium, and stereoselectivity across a spectrum of organic chemistry reaction types.^2,3^ Of interest in this work are polarized systems, such as the generation of an azomethine ylide from a zwitterionic tertiary amine *N-*oxide for 1,3-dipolar cycloadditions^4^ with polar organolithium reagents, such as LDA with known aggregation effects.^5^ The impact of solvent models on polarized systems has received comparatively less computational attention and is more challenging^6^ to dissect and understand fundamentally.^7−16^

**Figure 1:**
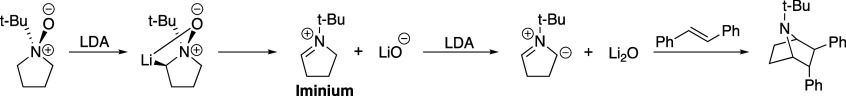
Mechanism by Roussi for *N-*oxide double deprotonation, forming the azomethine ylide for reaction with *trans*-stilbene.

In 1982, Roussi et al. published a route to generate pyrrolidines through a [3 + 2] cycloaddition where an azomethine ylide was generated via a double deprotonation of a tertiary amine *N*-oxide using LDA in THF (Figure 1).^17^ The originally postulated mechanism stated that deprotonation and loss of oxygen would yield an iminium,^18^ a highly reactive electrophilic species. This iminium was then deprotonated to provide an azomethine ylide intermediate capable of participating in 1,3-dipolar cycloadditions.

However, in 1992, Roussi demonstrated greater substrate flexibility than expected when utilizing *N-*oxide containing unprotected alcohols.^19^ While minor amounts of oxazolidine product were observed, the major products remained as the outcome of cycloadditions, suggesting that the mechanism was unlikely to move through the iminium. Yet, with this new experimental evidence, no further work was published to postulate a new mechanism.

One reason for the paucity of computational data is the sheer size of the physical model. However, quantum mechanical models for efficient and accurate prediction of energies and geometries for condensed phase reactions have evolved significantly.^20−23^ Two broad techniques, referred to as implicit and discrete models of solvation, are commonly used to model solute–solvent interactions to balance the interplay between accuracy and cost.^24^ The use of continuum models defining the implicit approach is a popular technique characterized by the application of a dielectric constant on a molecular cavity.^25−29^ An attractive feature of continuum models with *ab initio* quantum mechanics is the computational efficiency realized with good accuracy, especially for capturing long-range, bulk phase effects.^26,30,31^ However, continuum approaches have limitations, especially with simulating stronger noncovalent interactions such as hydrogen bonding or dipole effects in charged systems, where more sophisticated approaches are required to model experimental conditions in these systems correctly.^20,22^ On the other hand, discrete solvation models include solvent explicitly in the quantum system. The main drawback is the computer resources required to include even a modest number of solvent molecules by using higher levels of quantum theory.

With both discrete and continuum models possessing complementary strengths, efforts have been made to explore a *discrete-continuum* approach, utilizing a continuum method with strategically added explicit solvent, hydrogen bonds, and dipolar acceptors in the quantum system to describe solvent–solute interactions.^21,23,32−34^ Many implementations of continuum models have been used and tested,^35^ where absolute and relative free energies have been benchmarked.^36,37^ Specifically, several commonly used solvation models, including Tomasi’s polarizable continuum model (PCM), over a broad range of systems, were validated with other developed models.^14,38−40^ This allows for modeling of both bulk long-range solvent effects using resource-efficient PCM and stronger short-range intermolecular interactions between solvent and solute explicitly.^41−44^ While continuum methods provide accurate calculations for chemical reactions containing neutral species and aprotic solvent,^24^ utilization of a discrete-continuum approach with polar reactions improves the accuracy of energy barriers and leads to better agreement with experimental data.^45,46^ Error in predicted energy barriers can lead to flawed mechanistic analysis, highlight incorrect intermediates, and provide misdirecting theoretical interpretations.^47^ This is particularly important as many chemical reactions occur in polar, or polarizable, solvents and involve charged or polarized intermediates.

Using a discrete-continuum computational approach, our previous work reinvestigated Roussi’s mechanism for the generation of an azomethine ylide from a tertiary amine *N-*oxide.^33,48^ Through the modeling of different transition structures, we found that it was more energetically favorable to proceed with two sequential deprotonation steps before undergoing the nitrogen–oxygen dissociation to generate the azomethine ylide.

**Figure 2:**
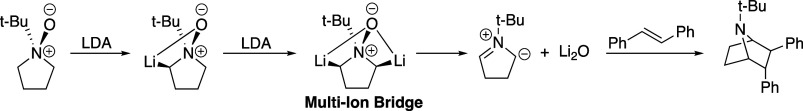
Mechanism for *N-*oxide double deprotonation passing through the multi-ion bridge forming the azomethine ylide for reaction with *trans*-stilbene.

Therefore, the mechanism proceeds through a multi-ion bridge intermediate, which is nonelectrophilic, prior to the generation of the 1,3-dipole (Figure 2) to avoid side reactions and enhance reaction yield. We have reported^33^ that *N-*oxides are polarized systems that require the correct coordination of lithium with explicit solvent to produce a reliable mechanism in agreement with experimental evidence.^49^ However, we did not describe our systematic, logical progression of model expansion to stay aligned with known experimental solvent behaviors and aggregation effects, which guide the quantum mechanical treatment of polarized intermediates.^50,51^

**Figure 3:**
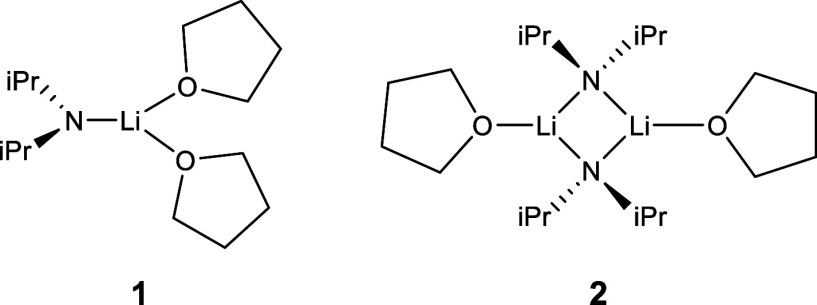
Monomeric (**1**) and dimeric (**2**) forms of LDA in THF solvent.^52,53^

Crucial to the conceptual understanding and employed computational model in this work is the coordination geometry of Li and its bridging of diisopropylamide in a dimeric form, as shown in Figure 3. In 1984, the first structural data for lithium diisopropylamide (LDA) in tetrahydrofuran (THF) were established, showing the presence of both a monomeric and dimeric form.^53^ By 1988, X-ray crystallography and NMR studies had further confirmed the dimeric aggregation of LDA in THF (Figure 3).^52,54^ Organolithium reagents’ aggregation and solvation effects, especially LDA, have been shown to impact reaction kinetics.^55^ Collum has extensively studied LDA aggregation effects in organic mechanisms, concluding that in certain reactions, the rate-limiting step results in the disaggregation of the LDA dimer.^56^ Additionally, the community has emphasized the importance of mixed aggregate effects on the synthetic necessity for excess organolithium to improve reaction yields.^57^

We chose the generation of azomethine ylides from *N*-oxides as the target of this work since it is of interest to our wider research program. The work reported here demonstrates the impact of aggregation effects along reaction pathways of polarized systems, specifically how systematic expansion of the physical model influences the *N-*oxide double deprotonation passing through the multi-ion bridge, forming an azomethine ylide. We begin by first outlining the role solvation plays in the two transition structures, nitrogen–oxygen dissociation and the second alpha-hydrogen deprotonation, which compete to determine if either the iminium or multi-ion bridge intermediate is formed. This is followed by highlighting the limitation of the bridging of THF, which was observed in attempts to model the solvation without the presence of LDA aggregation. In our second effort, we compare the stability of the bridging THF solvation with the previously reported LDA dimer structure. Finally, we discuss an improved solvation model where a bridging amide rather than a THF across two equivalents of LDA forms aggregates to describe *N*-oxide transformation to azomethine ylides consistent with experimental outcomes.

## 2 Results and Discussion

**Figure 4:**
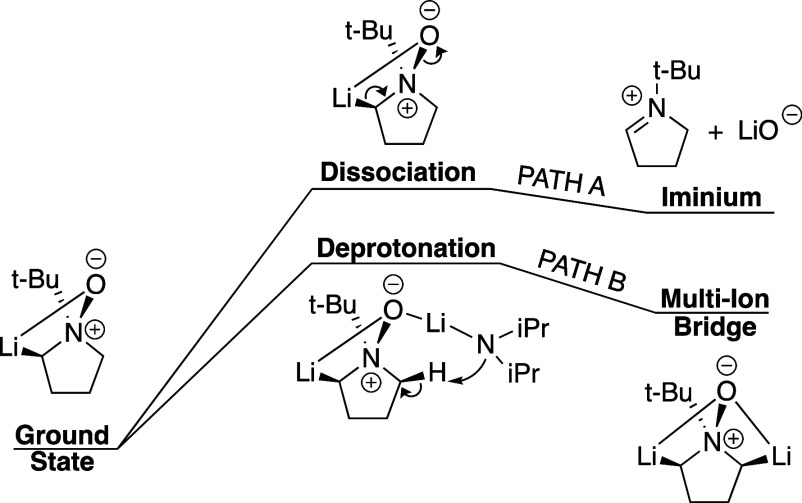
Qualitative energy potential diagram showing the two-competing paths which determine the formation of either the iminium ion (Path A) or multi-ion bridge intermediate (Path B). Solvent and dimeric LDA included in the common ground state are omitted here for the sake of clarity.

There are two proposed mechanistic pathways for the double deprotonation reactions that convert *N*-oxides into azomethine ylides: the first pathway advances through a discrete iminium ion and the second through a multi-ion bridged intermediate (Figure 4). Our previous research using *tert*-butyl pyrrolidine *N*-oxide focused on the competition between these two transition structures stemming from the same ground state (Figure 4). Path A involves the dissociation of the nitrogen–oxygen bond, forming both the lithium oxide anion (LiO^–^) and an iminium cation. Alternatively, Path B involves a second directed deprotonation, generating a multi-ion bridged intermediate. Prior work indicates that Path B is more likely,^58^ and this was in agreement with our proposed mechanism when an appropriately selected discrete-continuum solvation model was employed. Having a firm understanding of the reaction pathway will empower synthetic chemists to make the best use of this reaction, understanding its limitations and capabilities.

Here, we discuss the role that explicit solvent/environment plays in the distinction between the key transition structures along each pathway. For a direct comparison of different models, a common ground state was selected, consisting of two LDA/THF dimers, one free THF, and the *N*-oxide. The preferred energy pathway can switch depending on the solvent model utilized, highlighting the importance of selecting a balanced system that is reflective of known aggregation and solvent effects.

### 2.1 Preliminary Studies into Inclusion of Solvent

**Figure 5:**
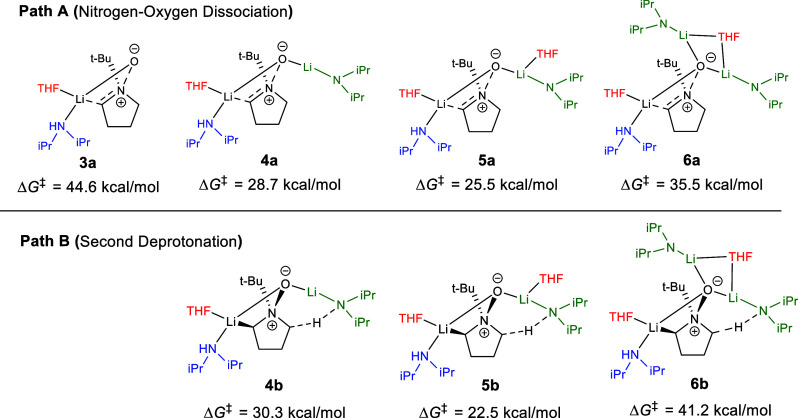
Transition structures compare competing Paths A and B with different solvation models and their corresponding transition state energies. The reported Δ*G*^⧧^ is the free volume corrected transition structure energy relative to the common ground state in kcal/mol (see Figures S47a–S47d).

We started the investigation by incorporating a single LDA and an explicit THF molecule into the model. Following deprotonating loss of LiO^–^ as postulated by Roussi led to a 44.6 kcal/mol free energy barrier (**3a**) as compared to the common ground state (see Figure 5). We realized the importance of stabilizing the negative charge on the forming oxide, leading to the incorporation of an additional LDA in the model. Comparison of the dissociation activation free energies of **3a** (44.6 kcal/mol) and **4a** (28.7 kcal/mol) demonstrates the impact of the explicit solvent model. Next, we investigated the competition between transition structure **4a** for nitrogen–oxygen dissociation (Path A yielding iminium) and the second deprotonation reaction pathway **4b** (Path B yielding the multi-ion bridged structure) (Figure 5). The activation free energy of deprotonation transition structure **4b** was calculated to be higher (1.6 kcal/mol) than dissociation structure **4a**. However, the **4a** and **4b** structures have only two groups coordinated to the lithium (green) of the second equivalent of LDA added (Figure 5). Completing lithium’s coordination sphere^59^ with an additional THF gives transition structures **5a** and **5b**, switching the preference and strongly favoring deprotonation by ca. 3.0 kcal/mol. The completion of lithium’s coordination sphere in **5a** decreased the activation energy needed for the dissociation pathway from **4a** to **5a** (−3.2 kcal/mol). For the deprotonation, when the coordination sphere is completed, it alters the ground state geometry at lithium, changing it from a more linear (**4b**, ∠O–Li–N = 141.7°) to a trigonal planar (**5b**, ∠O–Li–N = 127.6°) arrangement, decreasing the distortion needed for deprotonation (Table 1) and thereby lowering the activation energy (−7.8 kcal/mol). The coordination consequence is that even though the key N···H···C bond distances were maintained in the transition structure with only 0.01 Å change, the ground state H···N distance compressed by 0.16 Å, from 2.56 to 2.40 Å, in **5b** for better positioning to reach the transition structure (Table 1). This corresponds to known trends where coordinating solvents increase the reactivity of lithium anions.^60−62^

**Table 1:** Bond Distances (Å) and Angles (Deg) for Second Deprotonation Transition Structures Listed in Figure 5

	Transition Structure Bond Distance (Å)		Ground Structure Bond Distance (Å)		Difference in Bond Distance (Å)	
Structure	C–H	H–N	C–H	H–N	C–H	H–N
**4b**	1.55	1.23	1.09	2.56	0.46	–1.33
**5b**	1.56	1.22	1.10	2.40	0.46	–1.19
**6b**	1.46	1.28	1.09	2.35	0.37	–1.07

Structure	Transition Structure Bond Angle (◦)		Ground Structure Bond Angle (◦)		Difference in Bond Angle (◦)	
	C–H–N	O–Li–N	C–H–N	O–Li–N	C–H–N	O–Li–N
**4b**	167.6	131.0	157.7	141.7	9.8	–10.7
**5b**	167.8	114.4	160.7	127.6	7.0	–13.1
**6b**	175.4	135.1	178.8	141.1	–3.4	–6.1

Experimentally, it has been determined that three equivalents of LDA are required to achieve complete conversion of starting materials to products.^48,63^ Consequently, a third LDA was introduced in structures **6a** and **6b** (Figure 5) to further stabilize the oxide negitive charge. To our surprise, an addition of 1 equiv of LDA predicted that the dissociation pathway should be favored by 5.7 kcal/mol, a flip from the energetic difference between **5a** and **5b**. It was interesting to see an activation energy increase from **5a** to **6a**, opposite to the energy barrier decrease going from **3a** to **4a**, since we anticipated that the additional equivalent of LDA would further stabilize the anionic oxygen during dissociation. Moreover, transition structure **6b** had a significant energy barrier increase (+18.7 kcal/mol) compared to that of **5b**. The reason for the selective stabilization of Path A over Path B seemed to transcend more than charge and coordination arguments, which necessitated changes in our approach. At this point, our model contained the number of LDA moieties found to be experimentally optimal with a 3:1 ratio between LDA and the *N*-oxide. Further attempts to add THF did not result in lower energy complexes (Figures S28–S33). It was unacceptable at this stage of research that further expansions of the physical model were not leading to a converged prediction of a preferred pathway. Given that our most sophisticated model was predicting a result contrary to observed experimental outcomes, and both distortion/interaction energy and geometric analysis indicated that **6** deviated from prior aggregation patterns (Tables 1 and S9), we decided to investigate the solvent model more deeply by incorporating lithium aggregation effects according to experimental studies.^64,65^

### 2.2 Bridging THF versus LDA Dimer Aggregates

**Figure 6:**
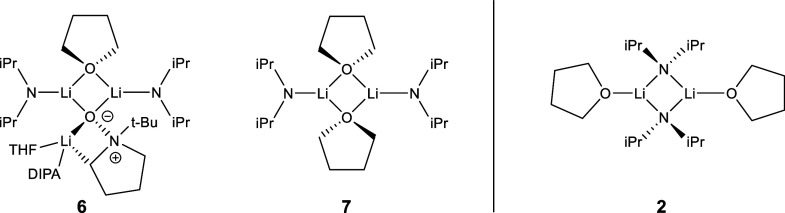
Three possible LDA in THF aggregate structures, one featuring bridged THFs (**7**), similar to those identified in our mechanistic calculations (**6**), and the other aggregate structure (**2**) with bridged diisopropyl amides as reported in the literature.^52,53^

As the complexity of our system grew, we wanted to ensure that we were incorporating discrete solvent into our model in a manner consistent with the experiment. It is well established that LDA, especially when solvated with THF, forms aggregate structures.^52,53,66,67^ However, spectroscopically observed aggregates do not involve bridging THFs, such as in **6** and **7**, but have bridging amides as shown in **2** (Figure 6).^68^ Since our computational models (e.g., **6**) invoke structures similar to **7** as opposed to the experimentally observed **2**, we strove to understand the energetic differences between LDA dimerized with bridging THFs, **7**, and the observed LDA dimer **2**. Therefore, we optimized the geometries of both **2** and **7** in Figure 6 using three different density functionals and Møller–Plesset perturbation theory (MP2) along with three different basis sets (Table 2). Across the different levels of theory, dimer **2** was consistently lower in energy by 13–22 kcal/mol (Table S5) compared to dimer **7**, in alignment with experimental observations.

**Table 2:** Free Energy Differences (kcal/mol) when Moving from Structure **7** to **2** Computed with Different Functionals and Basis Sets^a^

	Basis Set	mAUG-cc-pvDz	mAUG-cc-pvTz	JUL-cc-pvDz
Functional	Structure	difference	difference	difference
M06-2x	**7**	–14.0	–18.6	–19.4
	**2**			
B3LYP	**7**	–16.8	–17.5	–18.3
	**2**			
HCTH407	**7**	–13.0	–13.8	–13.2
	**2**			
MP2	**7**	–21.9	–19.4*	–16.1
	**2**			

a The thermodynamic correction from the MP2/mAUG-cc-pvDz was used to correct the electronic energy at MP2/mAUG-cc-pvTz to calculate the energy difference.^71^

To gain further insight into the dimerization preference, a distortion/interaction energy analysis^69^ was carried out on the individual components of **2** and **7**. The LDAs in **2** are only 4.4 kcal/mol more distorted than **7**, resulting from the nitrogens distorting from trigonal planar toward tetrahedral geometry, with the average ∠C–N–Li angle decreasing by 8.3° from **7** (123.0°) to **2** (114.7°) (Figures S14/S2). Most significantly, there is an increase of 22.8 kcal/mol in interaction energy when the nitrogens bridge (**2**) instead of the oxygens in THF (**7**) (Table S10). This correlates well with the change in energy (19.4 kcal/mol) between the two complexes (**2** and **7**).

Utilizing NBO population analysis^70^ on **2** and **7**, specifically the second-order perturbation approximation of charge-transfer between donor and acceptor orbitals, we observe that the bridging THFs in **7** contribute 22.5 kcal/mol from the oxygen lone pairs into the acceptor orbitals of the lithium atoms. Analogously, in **2**, the bridging LDAs contribute 35.1 kcal/mol from the nitrogen lone pairs (Table S11). These findings are consistent with the expectation that amides are better Lewis bases than ethers. Consequently, these results provide strong evidence that the bridged THF solvation observed in transition structures **6a** and **6b** (Figure 5) is an unrealistic aggregation, which is impacting our model’s accuracy by trapping the complex in a high-energy minimum. In order to provide a more accurate solvation model with two or more solvating LDAs, we needed to determine a lower-energy solvation aggregate to evaluate whether this model still reflects the experimental results.

### 2.3 LDA Aggregation with Model *N*-Oxide Systems

**Figure 7:**
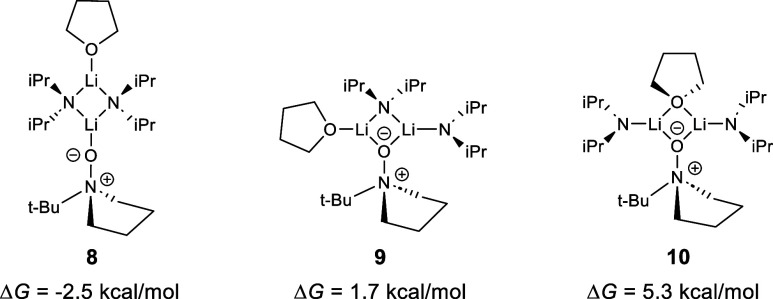
Different LDA/THF/*tert*-butyl pyrrolidine *N-*oxide aggregates and their stabilization free volume corrected free energies (kcal/mol) compared to the free energy of the common ground state.

To build different dimer aggregates in our *N*-oxide systems, we started with our previously reported ground state structures of *tert*-butyl pyrrolidine *N-*oxide prior to the first deprotonation.^33^ Three different ground state aggregates were constructed (Figure 7): **8** has both LDA nitrogens bridging the lithiums, with the *N*-oxide and THF external to the dimer; **9** places the anionic *N*-oxide oxygen and one LDA nitrogen bridging; and **10** has the anionic *N*-oxide oxygen and a THF oxygen bridging. Structure **8** closely mimics the experimentally observed LDA dimer **2**, with structure **10** most closely mimicking structure **6** as initially found in our computations, and structure **9** sits as a hybrid between the two. The system with bridging LDA nitrogen molecules in **8** was computed to be 7.8 kcal/mol lower in free energy compared to **10** with both oxygens bridging. Unsurprisingly, the mixed aggregate system **9** was energetically between **8** and **10** (Figure 7). Despite **8** having the lowest energy, both nitrogens are unavailable for proton abstraction in the dimer aggregation, where the deprotonation transition structure requires significant geometric distortion from **8** (4.5 Å), resulting in higher activation energies than the deprotonation structures from **9** (2.5 Å).^33^ This geometry is likely a resting state for the system, which exists outside of the mechanistic pathway similar to patterns observed experimentally.^60^ Consequently, the expected initial aggregate formation should follow the pattern established for model system **9**.

To further dissect the stability of dimeric model systems **8**–**10**, we conducted a distortion/interaction energy analysis^72^ on the ground structures in Figure 7 and Table 3. Structure **8** does not include the *N*-oxide oxygen directly into the dimer, so it should be most similar to **2**. In fact, both **2** and **8** have total LDA distortion energies of 4 kcal/mol along with LDA shifting its geometry by 17 degrees from trigonal planar to tetrahedral (Figures S2, S15–S17). As expected, the interaction energy of **8** is large (−57.9 kcal/mol, Table 3) similar to that of **2** of −59.2 kcal/mol (Table S10), where **2** is enhanced due to the greater donation ability of the *N*-oxide anionic oxygen as compared to THF. When the anionic oxygen of the *N*-oxide interdigitates into the dimer for **9** and **10,** we see greater interaction and distortion in **9** due to the more Lewis basic amide and oxide. On the other hand, dimer complex **10** loses stability compared to **9** without the bridging amide. To gain deeper insight into the electronics of the aggregation, we conducted an NBO analysis. Second-order perturbation approximation of charge-transfer in **8**–**10** shows increased transfer from the nitrogens to the lithiums as compared to that of either of the oxygen donors. A total of 33.5 kcal/mol is transferred from the amide donors into the acceptor orbitals of the lithiums in **8**, compared to 23.4 and 20.0 kcal/mol from the bridging heteroatoms in **9** and **10**, respectively (Table S12). The difference in energy between **8** and **10** mirrors the differences between **7** and **2** discussed above, with **9** providing a mix between the two. Our conclusion from this analysis is that an amide should be included in any aggregate structure, strongly indicating that structure **6** is not mechanistically relevant.

**Table 3:** Distortion/Interaction Energy (Electronic Energies) in kilocalorie per Mole of Each Component in the Three LDA/THF/*tert*-Butyl Pyrrolidine *N*-Oxide Aggregates Listed in Figure 7 Computed at M06-2x/jul-cc-pVDZ Level of Theory

	Distortion Energies					
Structure	LDA	LDA	THF	*N*-Oxide	Total Distortion Energy	Interaction Energy
**8**	5.2	5.9	0.1	0.2	11.3	–57.9
**9**	6.4	4.7	0.1	0.6	11.9	–54.2
**10**	4.0	3.8	0.4	0.4	8.5	–46.7

### 2.4 Improved Aggregate Systems

**Figure 8:**
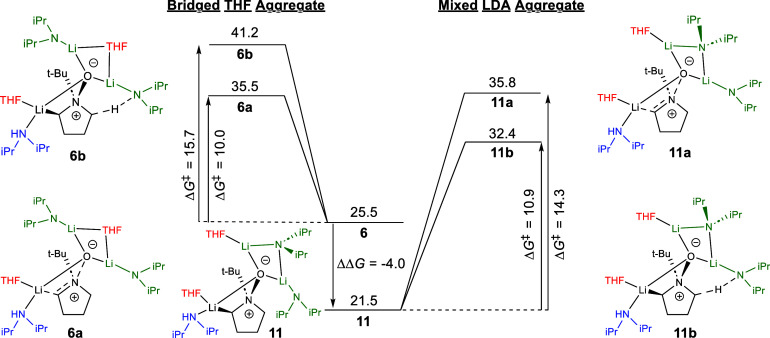
Transition structures for the competing nitrogen–oxygen dissociation and the second deprotonation steps. Comparison of bridged THF versus bridged LDA on both the free volume relative free energy compared to the common reference point of the starting materials and activation free energies of transition structures in kcal/mol. The reported Δ*G*^⧧^ is the transition structure energy minus the common ground state in kcal/mol. Vertical comparison highlights the competition between Paths A and B within a framework of aggregation, whereas horizontal comparison underscores the difference between THF and LDA bridged aggregation.

From lessons learned, as discussed previously, we moved to reevaluate the aggregation pattern of **6** along Paths A and B. Structure **10** (Figure 7) maps well onto our initially identified stationary points for **6a** and **6b**, with the THF and oxygen of the *N*-oxide bridging two lithiums (Figure 8). By changing the system so LDA bridges the dimer center, we built structures **11a** and **11b** (Figure 8) which correlate to the patterns seen in complex **9**, which was favored compared to model system **10**.

N–O dissociation **11a** showed a free energy comparable to that of dissociation **6a** (35.8 vs 35.5 kcal/mol). However, we see a significantly lower free energy for **11b** compared to **6b** (32.4 vs 41.2 kcal/mol) with rearranged solvent aggregation. These data match well with the aggregation pattern seen in our model system (Figure 7). Structure **11**, containing a mixed LDA dimer, is more stable by 4.0 kcal/mol compared to the THF bridged aggregate **6**. Similar to our model systems above, the decrease in energy of **11** is a result of increased stabilization of the dimer, where an NBO analysis shows 27.2 kcal/mol donated from the bridging heteroatoms into the lithiums’ acceptor orbitals compared to 20.3 kcal/mol in **6** (Table S13). When the activation energies were calculated, the second deprotonation was the more energetically favorable transition by 3.4 kcal/mol, bringing this back in line with experiment.

**Figure 9:**
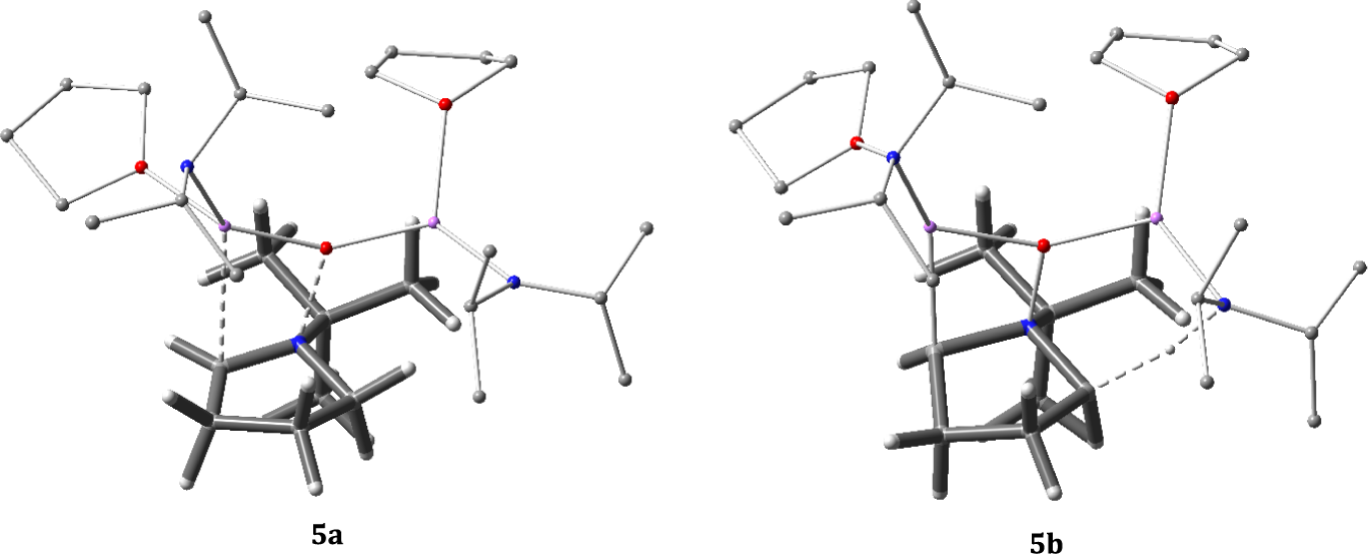
Three-dimensional plots of the transition structures for the competing nitrogen–oxygen dissociation (**5a**) and second deprotonation step (**5b**). The pyrrolidine *N*-oxide (tube) is highlighted, and hydrogens on the LDAs and THFs (wire) are omitted for clarity. Gray = carbon, blue = nitrogen, red = oxygen, pink = lithium, white = hydrogen.

**Figure 10:**
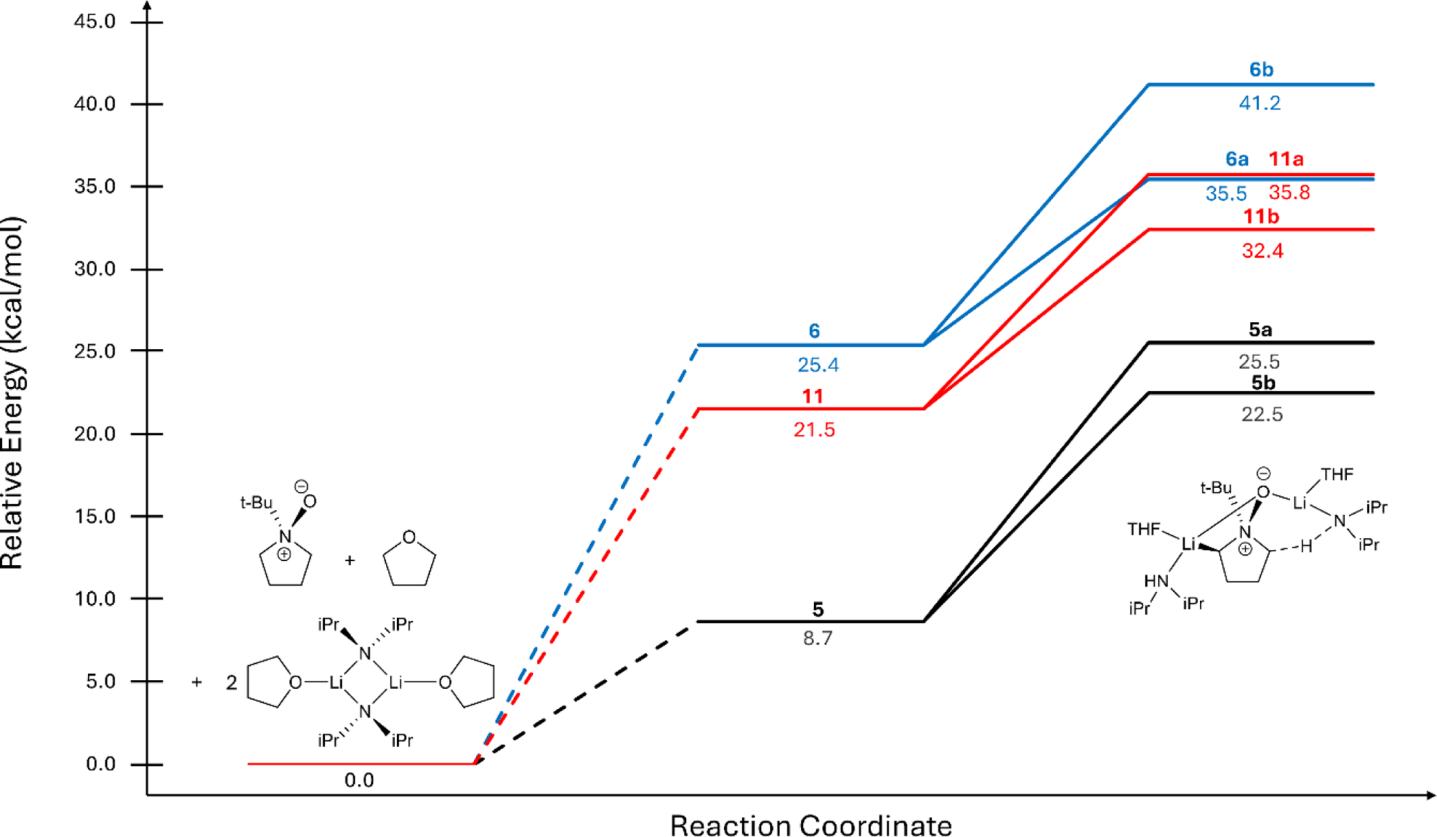
Direct comparison of the free energy diagrams between **5** (black), **6** (blue), and **11** (red). The common ground state (0.0 kcal/mol) is comprised of *tert*-butyl pyrrolidine *N*-oxide, THF, and dimeric LDA. Free volume correction has been applied to all free energy values.

However, when looking at each reaction pathway with different levels of solvation, referenced back to a common ground state, we found that complexes **5a** and **5b** (Figure 9) were favored in an absolute energetic sense (Figure 10), with the chelation of two LDAs and two THFs. This was driven by the strong entropic penalty from disrupting the LDA/THF dimer. This stands in contrast to the uncorrected translational entropy, where **11** was favored over both **5** and **6**. Consequently, we find that only two LDAs aggregate with the *N*-oxide and that the third (as found experimentally) has an undefined role in the solvent or external field.

## 3 Conclusion

Here we show that within polarized systems, such as zwitterionic *N*-oxides and 1,3-dipoles, coordination and placement of explicit solvent play significant roles in accurately modeling the energy barrier for these systems. Specifically, we found it is critical to evaluate the amount of explicit solvent added, how the solvent aggregates with the solute, account for translational entropy, and adjust for the standard state. For our system, it was important to ensure the lithium centers were properly coordinated with either three or four ligands. Of particular note is, as is true in all quantum mechanical computation, the starting geometry dictates the resulting minimized aggregates that are located. It has been shown by experiment that LDA has an important aggregate role in mechanisms and that LDA in THF prefers to exist in a dimerized form with bridging LDA molecules. Our initial models started with THF molecules in the bridging position, resulting in a higher energy local minimum. Systematic investigation into the number of equivalents of LDA added showed that only two LDAs aggregated to produce a lower and reasonable rate-determining step in free energy diagrams. This suggests that the third equivalent of LDA found to be experimentally optimal has an indirect role in the overall reaction. Only by correcting the entropy and utilizing a common ground state were we able to find the lowest free energy pathway, bringing our theoretical model into strong alignment with experimental observations. When studying the mechanism of polarized intermediates, it is important to account for the role explicit solvation plays within the quantum system and how solvent aggregates in order to draw accurate structural, energetic, and mechanistic conclusions.

John Pople taught the theoretical and computational community that a successful *theoretical model* has two components, the quality of the Hamiltonian and wave function, influencing the reproduction and prediction of natural phenomena, which has been coined as the “Pople Diagram”.^73,74^ During the intervening decades, there have been tremendous advances both in the power of computers^75^ and in the methodology of quantum chemical calculations.^76^ Contemporary calculations at the highest level of accuracy for over 4000 electrons have been reported,^77^ now increasing the size of molecular systems investigated by over 3 orders of magnitude from Pople’s original work.^74^ Consequently, it is now possible to carry out *ab initio* calculations that are of sufficient accuracy to answer chemically important questions for reasonably complex molecules and generate potential surfaces describing their reactions. In this study, we demonstrate a third axis to the “Pople Diagram”, the overall sophistication of the chemical model accounting for the inclusion of solvent in the quantum system.

## 4 Experimental Methods

Quantum calculations were carried out at the Center for Computational Sciences (CCS) at Duquesne University^78^ using Gaussian 16.^79^ The M06-2x, B3LYP, and HCTH407 functionals^80^ with Dunning’s [maug,jul]-cc-pV[D,T]Z basis sets^81^ were used primarily to calculate electronic, enthalpic, and free energies for both ground and transition structures (see Tables S1–S8). Free energies are given throughout the manuscript. We verified our theory level, while also using jul-cc-pVDZ, with second-order Møller–Plesset (MP2) calculations (see Table S14).^82^ Vibrational frequency calculations were used to confirm all stationary points as either minimum or transition structures and provide thermodynamic corrections for enthalpies and free energies. Implicit solvation models were integrated into each stage of our analysis. The integral equation formalism polarizable continuum model (IEFPCM, typically referred to as PCM) was developed by Tomasi et al.^83^ and reviewed elsewhere.^84−86^ Briefly, PCM allows the model to include bulk solute electrostatic interactions that impact the stability of stationary states; in this model, we use the dielectric constant of 7.2457 for THF, the UFF cavity model with an alpha scaling factor of 1.1.^87^ It has also been reported that the inclusion of explicit solvent, in combination with PCM, provides an improved model.^88,89^ Intrinsic reaction coordinate (IRC) calculations using the local quadratic approximation for the predictor step were used to validate the reactant and product from the vibrational frequency of each transition structure.^90^ The theory and implementation of IRC calculations have been described elsewhere.^91,92^ In this study, the reactants and products were identified through IRC calculations by tracing the reaction path to provide the electronic energies necessary to provide both the activation energy and the energy of the reaction.

The prediction of accurate free energies and solvent effects on reaction mechanisms in solution continues to be an area of important and challenging inquiry.^34,93−95^ Solution phase free energies obtained directly from electronic structure calculations are carried out in the gas phase at 1 atm and 298.15 K, with the translational entropy component computed using the ideal gas approximation in the Sackur–Tetrode equation.^102^ Corrections that adjust the standard state for the condensed phase (1 M) based upon the ideal gas equation have been reported.^85,96^ In our work, we employ standard state and translational entropy corrections in THF solution using the free volume theory developed by Whitesides et al.^97^ and applied using the GoodVibes software package^98^ for all free energies reported in this manuscript.^99^ The volume of THF was calculated using M06-2x with the jul-cc-pVDZ Dunning basis set using Gaussian 16’s “volume = tight” keyword. Note that a concentration of 12.25 M was used for THF solvent molecules to match measured density, while the standard 1 M concentration was used for all other species.

Tomasi et al. report that the nonelectrostatic terms of dispersion energy and cavitation energy are both small and relatively constant, so omitting them does not affect predicted structures and relative energies in solvent.^100^ As such, the current implementation of PCM^27^ in G16^79^ treats only the electrostatic components by default.^101^ However, it is important to include the solution translational entropy when describing the Gibbs free energy, even if approximated by the ideal gas equation as formulated by the Sackur–Tetrode equation,^102^ as we have described.

**Figure 11:**
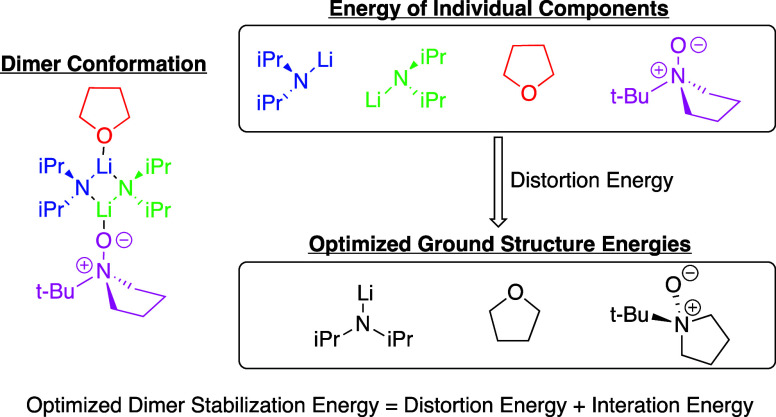
Graphic representation of the distortion/interaction energy analysis employed in this work.

We implemented the Distortion/Interaction Model method described by Ess and Houk, where they decomposed the activation barrier Δ*E*^⧧^ into a distortion energy () and an interaction energy () to describe 1,3-dipolar and Diels–Alder cycloadditions.^69,103−106^ is the energy required to distort the diene and the dienophile complex into TS geometries without allowing interaction between them. The activation barrier is then the sum of the distortion and interaction energies: , where the interaction energy term includes all of the stabilizing and repulsive interactions between the fragments at the TS or in the complex.^69,103,104^ As shown in Figure 11, to determine the distortion energy within our complexes, individual single-point energy calculations were performed on the discrete components at the geometries each component adopts in the complex. The calculated energies are then compared to the components’ energies within the ground structure, providing the energy needed to distort each component into its complexation geometry. Interaction energies can then be calculated from the equation specified above.

Additionally, natural population analysis (NPA),^107^ electrostatic potentials using a grid-based method (CHelpG),^108^ and Hirshfeld population analyses^109−111^ were utilized to calculate the charge distributions in the molecules of interest. While similar trends were observed across all three analyses, the Hirshfeld method was chosen due to its ability to describe noncovalent interactions accurately, as presented by Wiberg and Rablen.^112^ Throughout the paper, calculations to determine partial charges of atoms were conducted using Hirshfeld population analysis, unless otherwise noted. All calculations discussed were carried out using M06-2x/jul-cc-pDVZ within the polarizable continuum model using the dielectric constant for THF, unless stated otherwise.

## Data Availability

The data underlying this study are available in the published article and its Supporting Information.

## Supporting Information Available

Information on correlation between reported results with higher levels of theory, larger figures for each structure, input/output files for each structure, effects of aggregation on the first deprotonation, additional distortion/interaction energy details, and additional NBO details (PDF)
