# Supplementary material for: N-Oxide Insertion into LDA Dimeric Aggregates for Azomethine Ylide Formation: Explicit Solvation in Quantum Mechanical Treatment of Polarized Intermediates
Source: J Org Chem. 2025 Mar 1;90(10):3673–83. doi: 10.1021/acs.joc.4c03090 (PMC11915385; doi:10.1021/acs.joc.4c03090)
Supplement: Supplementary file 1 — jo4c03090_si_001.pdf [file jo4c03090_si_001.pdf]

N-oxide insertion into LDA dimeric aggregates for azomethine ylide formation: Explicit solvation in quantum mechanical treatment of polarized intermediates

Martin J. Neal[1†], Eric J. Chartier[1†], Aiden M. Lane[1], Sarah L. Hejnosz[1], Luke T. Jesikiewicz[2], Peng Liu[2], Jeffrey J. Rohde[3], Paul Lummis[1], Douglas J. Fox[4], Jeffrey D. Evanseck[1]*, and Thomas D. Montgomery[1]*

[1]Department of Chemistry and Biochemistry, Center for Computational Sciences, Duquesne University, 600 Forbes Avenue, Pittsburgh, PA 15282, United States

[2]Department of Chemistry, University of Pittsburgh, 219 Parkman Avenue, Pittsburgh, PA 15260, United States

[3]Department of Mathematics and Physical Sciences, Franciscan University of Steubenville, 1235 University Boulevard, Steubenville, OH 43952, United States

[4]Gaussian Inc. 340 Quinnipiac St #40, Wallingford, CT 06492, United States

† - MJN and EJC contributed equally to this work.

** Corresponding author:* montgomeryt1@duq.edu and evanseck@duq.edu

Supporting Information

Content

Aggregate Data Tables ***S3***

Calculated Energies for Transition and Ground State Structures 1-7 .... ***S10***

LDA – THF – tert-Butyl Pyrrolidine N-Oxide Dimers 8-10 .... ***S80***

Calculated Energies for Transition and Ground State Structures 11 .... ***S87***

Structures for the Analysis of Aggregation on the First Deprotonation S1-S3 .... ***S99***

Calculated Energies for Transition & Ground Structure with Additional THF S4 .... ***S113***

LDA – THF – Trimethyl N-oxide Dimer Structures S5-S7 .... ***S132***

Structures for Confirmation of Activation Energy with Higher Order Theory S8-S10 .... ***S139***

THF and tert-Butyl Amine N-Oxide Structures ***S156***

Energy Decomposition Analyses on Solvent Aggregates ***S159***

NBO Analyses on Solvent Aggregates ***S172***

Aggregation Effects on First Deprotonation ***S174***

Confirmation of Activation Energy with Higher Order Theory* *S175

Procedures for the synthesis of 7-Azanorbornane S45* *S176

Pertinant Free Energy Diagrams* *S179

General Methods: Calculations of structures, energies, and frequencies employed standard procedures in Gaussian16 unless otherwise noted. Complete structures and energetics are provided in sections below. All absolute energies are in Hartrees. All relative energies are presented in kcal/mol. Coordinates are given by atomic number followed by Cartesian coordinates (Å). The free volume method developed by Whitesides *et. al.* was used to correct for translational entropy. A standard state of 1 M was assumed for all species, except for THF, where a molarity of 12.25 M was assumed. A common ground state consisting of the *tert*-butyl pyrrolidine *N*-oxide, 2 LDA/THF dimers (**2**), and 1 free THF was used so that the major transition state free energies could be directly compared.

Aggregate Data Tables

Table S1. Absolute energies (Hartrees) and entropies (cal/mol K) for all the stationary points computed in the manuscript using m062x/jul-cc-pvDz with PCM (THF). The dissociation path (dis) and the proton abstraction path (abs) are labeled for clarity. The free volume correction to the entropy and free energy are shown. Free volume corrections are applied assuming 1 M species concentration, except THF (denoted with a star (*)) which assumes 12.25 M since it is the solvent. The double star (**) denotes the common ground state used throughout the manuscript.

Structure	E_{elec}	E_{298}	H_{298}	S_{298}	G_{298}	$S_{FV,298}$	$G_{FV,298}$
**1**	-764.02804967	-763.573580	-763.572636	162.789	-763.649982	146.420	-763.642196
**2**	-1063.33360667	-1062.670849	-1062.669905	212.435	-1062.770840	196.074	-1062.763054
**3** GS	-976.504723951	-975.912366	-975.911422	184.200	-975.998941	167.834	-975.991155
**3a** TS	-976.461969115	-975.872102	-975.871158	180.780	-975.957052	164.413	-975.949266
**4** GS	-1275.79308345	-1274.993109	-1274.992165	234.215	-1275.103448	217.856	-1275.095662
**4a** TS-dis	-1275.76415214	-1274.966275	-1274.965330	237.042	-1275.077957	220.685	-1275.070171
**4b** TS-abs	-1275.75886609	-1274.965045	-1274.964101	234.347	-1275.075447	217.989	-1275.067661
**5** GS	-1508.18382220	-1507.258873	-1507.257929	263.808	-1507.383272	247.451	-1507.375486
**5a** TS-dis	-1508.15215008	-1507.229178	-1507.228234	269.649	-1507.356353	253.295	-1507.348567
**5b** TS-abs	-1508.15405728	-1507.235207	-1507.234263	267.172	-1507.361205	250.817	-1507.353419
**6** GS	-1807.46624086	-1806.332829	-1806.331885	306.322	-1806.477428	289.971	-1806.469642
**6'** GS	-1807.46631068	-1806.333306	-1806.332362	302.771	-1806.476218	286.420	-1806.468432
**6a** TS-dis	-1807.44773114	-1806.316412	-1806.315468	307.195	-1806.461426	290.845	-1806.453640
**6b** TS-abs	-1807.43805166	-1806.310326	-1806.309381	300.832	-1806.452316	284.482	-1806.444530
**7**	-1063.30316080	-1062.640753	-1062.639808	210.589	-1062.739866	194.228	-1062.732080
**8**	-1275.81704935	-1275.017838	-1275.016894	233.300	-1275.127695	216.841	-1275.119909
**9**	-1275.80962993	-1275.010931	-1275.009987	233.750	-1275.121049	217.391	-1275.113263
**10**	-1275.80361732	-1275.004487	-1275.003543	235.284	-1275.115334	218.925	-1275.107548
**11** GS	-1807.47526309	-1806.341963	-1806.341019	300.104	-1806.483608	283.753	-1806.475822
**11'** GS	-1807.45762627	-1806.324305	-1806.323360	300.816	-1806.466288	284.467	-1806.458502
**11a** TS-dis	-1807.45061478	-1806.318895	-1806.317951	300.952	-1806.460943	284.602	-1806.453157
**11b** TS-abs	-1807.45170107	-1806.324302	-1806.323358	300.827	-1806.466290	284.475	-1806.458504

**S1** GS	-1275.82012015	-1275.020173	-1275.019229	228.477	-1275.127786	212.118	-1275.120000
**S1** TS	-1275.78488607	-1274.990374	-1274.989430	233.611	-1275.100426	217.252	-1275.092640
**S2** GS	-1275.80993451	-1275.010566	-1275.009622	232.360	-1275.120024	216.001	-1275.112238
**S2** TS	-1275.79060851	-1274.996458	-1274.995514	230.597	-1275.105078	214.237	-1275.097292
**S3** GS	-976.519032466	-975.927746	-975.926801	184.611	-976.014516	168.246	-976.006730
**S3** TS	-976.496302776	-975.910195	-975.909251	181.075	-975.995285	164.708	-975.987499
**S4** GS	-2039.84920033	-2038.590974	-2038.590029	333.910	-2038.748681	317.565	-2038.740895
**S4a** TS-dis	-2039.83149356	-2038.575127	-2038.574183	335.147	-2038.733422	318.801	-2038.725636
**S4b** TS-abs	-2039.82240227	-2038.569725	-2038.568781	335.115	-2038.728005	318.769	-2038.720219
**S4'** GS	-2039.84884455	-2038.589063	-2038.588119	327.157	-2038.743561	310.808	-2038.735775
**S4'a** TS-dis	-2039.81953210	-2038.561866	-2038.560922	328.248	-2038.716883	311.901	-2038.709097
**S4'b** TS-abs	-2039.81994294	-2038.564948	-2038.564004	322.495	-2038.717232	306.148	-2038.709446
**S5** GS	-1080.51844422	-1079.845630	-1079.844686	213.476	-1079.946115	197.114	-1079.938329
**S6** GS	-1080.50645754	-1079.833803	-1079.832859	219.880	-1079.937331	203.519	-1079.929545
**S7** GS	-1080.50265789	-1079.829904	-1079.828959	214.053	-1079.930663	197.693	-1079.922877
**S8** TS	-744.093122735	-743.631684	-743.630740	146.829	-743.700503	130.458	-743.692717
**S9** TS	-744.104645696	-743.643618	-743.642673	148.130	-743.713055	131.761	-743.705269
**S10** GS	-744.121165589	-743.654826	-743.653881	152.674	-743.726422	136.306	-743.718636
**THF***	-232.372503716	-232.250251	-232.249307	71.901	-232.283470	*50.535*	*-232.273315*
**LDA**	-299.257658880	-299.053792	-299.052848	96.441	-299.098670	80.064	-299.090884
***N*-Oxide**	-444.847205800	-444.588570	-444.587630	97.620	-444.634007	81.243	-444.626221
N*-Oxide** + 2x**2** + **THF *	-2803.88692286	-2802.18051900	-2802.17674700	594.391	-2802.45915700	523.926	-2802.42564401

Table S2. Activation energies (kcal/mol) and entropies (cal/mol K) for all the stationary points using m062x/jul-cc-pvDz with PCM (THF) to the previous ground state. Note that this table does not provide transition state energies relative to the common ground state so the values cannot be directly compared.

Structure	$\Delta E^{\ddagger}_{elec}$	$\Delta E^{\ddagger}_{298}$	$\Delta H^{\ddagger}_{298}$	$\Delta S^{\ddagger}_{298}$	$\Delta G^{\ddagger}_{298}$	$\Delta S^{\ddagger}_{FV,298}$	$\Delta G^{\ddagger}_{FV,298}$
**3a**	26.8	25.3	25.3	-3.42	26.3	-3.42	26.3
**4a**	18.2	16.8	16.8	2.83	16.0	2.83	16.0
**4b**	21.5	17.6	17.6	0.13	17.6	0.13	17.6
**5a**	19.9	18.6	18.6	5.84	16.9	5.84	16.9
**5b**	18.7	14.9	14.9	3.36	13.8	3.37	13.8
**6a**	11.6	10.3	10.3	0.87	10.0	0.87	10.0
**6b**	17.7	14.1	14.1	-5.49	15.8	-5.49	15.8
**11a**	15.5	14.5	14.5	0.85	14.2	0.85	14.2
**11b**	14.8	11.1	11.1	0.72	10.9	0.72	10.9
**S1**	22.1	18.7	18.7	5.13	17.2	5.13	17.2
**S2**	12.1	8.9	8.9	-1.76	9.4	-1.76	9.4
**S3**	14.3	11.0	11.0	-3.54	12.1	-3.54	12.1
**S4a**	11.1	9.9	9.9	1.24	9.6	1.24	9.6
**S4b**	16.8	13.3	13.3	1.20	13.0	1.20	13.0
**S4'a**	18.4	17.1	17.1	1.09	16.7	1.09	16.7
**S4'b**	18.1	15.1	15.1	-4.66	16.5	-4.66	16.5
**S8**	17.6	14.5	14.5	-5.85	16.3	-5.85	16.3
**S9**	10.4	7.0	7.0	-4.54	8.4	-4.55	8.4

Table S3. Relative transition state energies (kcal/mol) and entropies (cal/mol K) compared to the common ground state using m062x/jul-cc-pvDz with PCM (THF). These values can be compared with all other values relative to the common ground state. See Figures Sa-d to visualize all the species present at each transition state for for **3a, 4a**/**4b, 5a**/**5b, 6a**/**6b,** and **11a**/**11b**. For the other entries we get the following transition states from the common ground point: the transition state **S1**/**S2** + THF/LDA dimer (**2**) + 2 free THF; the transition state **S3** + THF/LDA dimer (**2**) + THF/LDA monomer (**1**); transition state **S4a/S4b/S4'a/S4'b** + LDA/THF monomer (**1**); and transition state **S8/S9** + LDA/THF dimer (**2**) + LDA/THF monomer (**1**) + free THF from the common ground state.

Structure	$\Delta E^{\ddagger}_{elec}$	$\Delta E^{\ddagger}_{298}$	$\Delta H^{\ddagger}_{298}$	$\Delta S^{\ddagger}_{298}$	$\Delta G^{\ddagger}_{298}$	$\Delta S^{\ddagger}_{FV,298}$	$\Delta G^{\ddagger}_{FV,298}$
**3a**	39.7	40.2	39.6	-38.39	51.0	-17.02	44.6
**4a**	27.7	26.9	26.9	-1.11	27.2	-6.10	28.7
**4b**	31.0	27.7	27.7	-3.81	28.8	-8.79	30.3
**5a**	18.0	19.0	18.4	-40.41	30.4	-24.02	25.5
**5b**	16.8	15.2	14.6	-42.88	27.4	-26.50	22.5
**6a**	24.2	25.3	24.7	-52.51	40.3	-36.13	35.5
**6b**	30.3	29.1	28.5	-58.87	46.1	-42.49	41.2
**11a**	22.4	23.7	23.1	-58.75	40.6	-42.37	35.8
**11b**	21.8	20.3	19.7	-58.87	37.3	-42.50	32.4
**S1**	14.7	11.8	11.8	-4.54	13.1	-9.53	14.6
**S2**	11.1	8.0	8.0	-7.56	10.2	-12.54	11.7

**S3**	18.2	16.3	15.7	-38.09	27.0	-16.72	20.6
**S4a**	17.2	20.0	18.8	-96.46	47.5	-58.70	36.3
**S4b**	22.9	23.4	22.2	-96.49	50.9	-58.74	39.7
**S4'a**	24.7	28.3	27.1	-103.35	57.9	-65.60	46.7
**S4'b**	24.4	26.4	25.2	-109.11	57.7	-71.36	46.4
**S8**	37.4	34.0	34.0	-0.44	34.1	-0.44	34.1
**S9**	30.2	26.5	26.5	0.86	26.2	0.86	26.2

Table S4. Absolute energies (Hartrees) and entropies (cal/mol K) for **2** and **7** computed in the manuscript using different levels of theory all with PCM (THF). Entry labeled with a star (*) indicates that the thermodynamic corrections of the MP2/maug-cc-pvTz were approximated by using the MP2/maug-cc-pvDz computed values. Entry labeled with a double star (**) shows the difference between the approximated MP2/maug-cc-pvDz and computed MP2/maug-cc-pvTz thermodynamic corrections. The free volume corrected free energies are shown.

Structure	E_{elec}	E_{298}	H_{298}	S_{298}	G_{298}	$S_{FV,298}$	$G_{FV,298}$
**7**							
M062x<br>jul-cc-pvDz	-1063.30316080	-1062.640753	-1062.639808	210.589	-1062.739866	194.228	-1062.732080
M062x<br>maug-cc-pvDz	-1063.26517884	-1062.604164	-1062.603220	220.789	-1062.708124	204.428	-1062.700338
M062x<br>maug-cc-pvTz	-1063.57648601	-1062.911219	-1062.910274	209.311	-1063.009725	192.950	-1063.001939
**7**							
B3LYP<br>jul-cc-pvDz	-1063.79603239	-1063.139250	-1063.138306	220.675	-1063.243156	204.315	-1063.235370
B3LYP<br>maug-cc-pvDz	-1063.75727618	-1063.102265	-1063.101321	225.074	-1063.208261	208.714	-1063.200475
B3LYP<br>maug-cc-pvTz	-1064.09055124	-1063.432776	-1063.431831	224.856	-1063.538668	208.497	-1063.530882
**7**							
HCTH407<br>jul-cc-pvDz	-1063.80796784	-1063.155830	-1063.154886	226.146	-1063.262335	209.786	-1063.254549
HCTH407<br>maug-cc-pvDz	-1063.77568257	-1063.124733	-1063.123788	223.311	-1063.229891	206.952	-1063.222105
HCTH407<br>maug-cc-pvTz	-1064.03483156	-1063.383617	-1063.382673	224.045	-1063.489124	207.685	-1063.481338
**7**							
MP2<br>jul-cc-pvDz	-1060.4311367404	-1059.768939	-1059.767995	220.824	-1059.872916	204.464	-1059.865130
MP2<br>maug-cc-pvDz	-1060.2960221722	-1059.631237	-1059.630292	218.057	-1059.733898	201.696	-1059.726112
MP2<br>maug-cc-pvTz*	-1061.3678946189	0.664786<br>-1060.703109	0.665730<br>-1060.702164	218.057	0.562124<br>-1060.805770	201.696	-1060.797984

**2**							
M062x<br>jul-cc-pvDz	-1063.33360667	-1062.670849	-1062.669905	212.435	-1062.770840	196.074	-1062.763054
M062x<br>maug-cc-pvDz	-1063.29074183	-1062.629326	-1062.628382	214.698	-1062.730391	198.335	-1062.722605
M062x<br>maug-cc-pvTz	-1063.60463515	-1062.939344	-1062.938400	212.460	-1063.039347	196.099	-1063.031561
**2**							
B3LYP<br>jul-cc-pvDz	-1063.82176999	-1063.165304	-1063.164360	227.069	-1063.272247	210.707	-1063.264461
B3LYP<br>maug-cc-pvDz	-1063.78225037	-1063.127392	-1063.126447	228.615	-1063.235070	212.257	-1063.227284
B3LYP<br>maug-cc-pvTz	-1064.11554994	-1063.457950	-1063.457006	230.700	-1063.566619	214.341	-1063.558833
**2**							
HCTH407<br>jul-cc-pvDz	-1063.82848024	-1063.175585	-1063.174641	228.707	-1063.283307	212.347	-1063.275521
HCTH407<br>maug-cc-pvDz	-1063.79606844	-1063.144334	-1063.143389	225.597	-1063.250578	209.238	-1063.242792
HCTH407<br>maug-cc-pvTz	-1064.05487643	-1063.402938	-1063.401994	229.592	-1063.511081	213.233	-1063.503295
**2**							
MP2<br>jul-cc-pvDz	-1060. 4589811425	-1059.796120	-1059.795176	217.674	-1059.898600	201.313	-1059.890814
MP2<br>maug-cc-pvDz	-1060.3262374820	-1059.661733	-1059.660788	227.359	-1059.768814	211.000	-1059.761028
MP2<br>maug-cc-pvTz	-1061.3940950105	-1060.728356	-1060.727412	231.010	-1060.837173	214.652	-1060.829387
MP2<br>maug-cc-<br>pvTz**		0.664505<br>-1060.729590<br>-0.8	0.665449<br>-1060.728646<br>-0.8	227.359<br>-3.7	0.557424<br>-1060.83667<br>0.3	210.996	-1060.828884

Table S5. Relative energies (kcal/mol) for the difference between **2** and **7** (**2** – **7)** using different levels of theory all with PCM (THF). Entry labeled with a star (*) indicates that the thermodynamic corrections of the MP2/maug-cc-pvTz were approximated by using the MP2/maug-cc-pvDz computed values.

Structure	ΔE_{elec}	ΔE_{298}	ΔH_{298}	ΔS_{298}	ΔG_{298}	$\Delta S^{\ddagger}_{FV,298}$	$\Delta G^{\ddagger}_{FV,298}$
**2-7**							
M062x<br>jul-cc-pvDz	-19.1	-18.9	-18.9	1.8	-19.4	1.8	-19.4
M062x<br>maug-cc-pvDz	-16.0	-15.8	-15.8	-6.1	-14.0	-6.1	-14.0
M062x<br>maug-cc-pvTz	-17.7	-17.6	-17.6	3.1	-18.6	3.1	-18.6
**2-7**							

B3LYP jul-cc-pvDz	-16.2	-16.3	-16.3	6.4	-18.3	6.4	-18.3
B3LYP maug-cc-pvDz	-15.7	-15.8	-15.8	3.5	-16.8	3.5	-16.8
B3LYP maug-cc-pvTz	-15.7	-15.8	-15.8	5.8	-17.5	5.8	-17.5
**2-7**							
HCTH407 jul-cc-pvDz	-12.9	-12.4	-12.4	2.6	-13.2	2.6	-13.2
HCTH407 maug-cc-pvDz	-12.8	-12.3	-12.3	2.3	-13.0	2.3	-13.0
HCTH407 maug-cc-pvTz	-12.6	-12.1	-12.1	5.5	-13.8	5.5	-13.8
**2-7**							
MP2 jul-cc-pvDz	-17.5	-17.1	-17.1	-3.2	-16.1	-3.2	-16.1
MP2 maug-cc-pvDz	-19.0	-19.1	-19.1	-9.3	-21.9	-9.3	-21.9
MP2 maug-cc-pvTz*	-16.4	-15.8	-15.8	13.0	-19.7	13.0	-19.7

Table S6. Relative stabilization energies (kcal/mol) and entropies (cal/mol K) for LDA/THF/tert-butyl pyrollidine *N*-oxide aggregates (**8-10**) compared to the common reference point using m062x/jul-cc-pvDz with PCM (THF). Note that the reaction yields **8**/**9**/**10** + **2** + 2 free THF from the common ground state.

Structure	ΔE_{elec}	ΔE_{298}	ΔH_{298}	ΔS_{298}	ΔG_{298}	$\Delta S_{FV,298}$	$\Delta G_{FV,298}$
**9**	-5.5	-5.4	-5.4	-4.85	-4.0	-9.94	-2.5
**10**	-0.8	-1.1	-1.1	-4.40	0.2	-9.39	1.7
**11**	2.9	2.9	2.9	-2.87	3.8	-7.86	5.3

Table S7. Absolute energies (Hartrees) and entropies (cal/mol K) for **S8 TS** and **S9 TS** and **S10 GS** computed in the manuscript using different levels of theory all with PCM (THF).The free volume corrected values for the entropy and free energy are also given.

Structure	E_{elec}	E_{298}	H_{298}	S_{298}	G_{298}	$S_{FV,298}$	$G_{FV,298}$
**S8 TS**							
M062x jul-cc-pvDz	-744.093122735	-743.631684	-743.630740	146.829	-743.700503	130.458	-743.692717
M062x jul-cc-pvTz	-744.284643579	-743.822281	-743.821337	146.273	-743.890836	129.903	-743.883050
M062x aug-cc-pvDz	-744.106408135	-743.645007	-743.644060	145.393	-743.713144	129.029	-743.705358
MP2 jul-cc-pvDz	-739.373536415	-741.636419	-741.635474	149.452	-741.706484	133.083	-741.698698
**S9 TS**							

M062x jul-cc-pvDz	-744.104645696	-743.643618	-743.642673	148.130	-743.713055	131.761	-743.705269
M062x jul-cc-pvTz	-744.296553410	-743.834639	-743.833694	149.021	-743.904499	132.652	-743.896713
M062x aug-cc-pvDz	-744.117813607	-743.656963	-743.656019	147.419	-743.726062	131.048	-743.718276
MP2 jul-cc-pvTz	-739.388070291	-741.647375	-741.646431	149.527	-741.717476	133.157	-741.709690
**S10 GS**							
M062x jul-cc-pvDz	-744.129564694	-743.663555	-743.662611	153.640	-743.735610	137.270	-743.727824
M062x jul-cc-pvTz	-744.324404436	-743.857282	-743.856338	152.668	-743.928876	136.300	-743.921090
M062x aug-cc-pvDz	-744.143052653	-743.677212	-743.676267	152.421	-743.748687	136.051	-743.740901
MP2 jul-cc-pvDz	-739.434485430	-741.665897	-741.664953	155.577	-741.738873	139.209	-741.731087

Table S8. Activation energies (kcal/mol) and entropies (cal/mol K) for **S8** and **S9** from **S10** computed in the manuscript using different levels of theory all with PCM (THF). Note that this table does not provide transition state energies relative to the common ground state.

Structure	$\Delta E^{\ddagger}_{elec}$	$\Delta E^{\ddagger}_{298}$	$\Delta H^{\ddagger}_{298}$	$\Delta S^{\ddagger}_{298}$	$\Delta G^{\ddagger}_{298}$	$\Delta S^{\ddagger}_{FV,298}$	$\Delta G^{\ddagger}_{FV,298}$
**S8**							
M062x jul-cc-pvDz	22.9	20.0	20.0	-6.8	22.0	-6.8	22.0
M062x jul-cc-pvTz	25.0	22.0	22.0	-6.4	23.9	-6.4	23.9
M062x aug-cc-pvDz	23.0	20.2	20.2	-7.0	22.3	-7.0	22.3
MP2 jul-cc-pvDz	38.2	18.5	18.5	-6.1	20.3	-6.1	20.3
**S9**							
M062x jul-cc-pvDz	15.6	12.5	12.5	-5.5	14.2	-5.5	14.2
M062x jul-cc-pvTz	17.5	14.2	14.2	-3.6	15.3	-3.6	15.3
M062x aug-cc-pvDz	15.8	12.7	12.7	-5.0	14.2	-5.0	14.2
MP2 jul-cc-pvDz	29.1	11.6	11.6	-6.1	13.4	-6.1	13.4

Calculated Energies for Transition and Ground State Structures 1-7

Ground State Structure 1

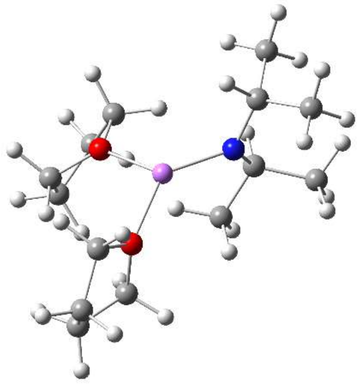

Figure S1. Representative ball and stick model for ground state structure **1**.

#m062x/jul-cc-pvDz opt=(calcfc,noeigen) optcyc=50 freq scrf=(pcm,solvent=THF)

EE =	-764.028050(Hartree)
ZPE_{298} =	-763.596580
E_{298} =	-763.573580
H_{298} =	-763.572636
G_{298} =	-763.649982
S =	162.789 (cal/mol·K)

3	0.548774	1.250832	0.384148
7	2.250170	0.718348	-0.235820
8	-0.971300	0.630836	-0.687179
6	-0.651754	-0.139981	-1.857170
6	-1.371131	-1.493384	-1.686777
6	-2.270694	-1.293137	-0.448357
6	-2.271412	0.222367	-0.265068
1	-1.020831	0.397148	-2.743225
1	0.440719	-0.207280	-1.893433
1	-1.964470	-1.729306	-2.576660
1	-0.656538	-2.309338	-1.533505
1	-3.280600	-1.692932	-0.587850
1	-1.830438	-1.778380	0.431674
1	-3.030717	0.700203	-0.905071
1	-2.408188	0.557372	0.769016
6	2.568031	-0.685225	-0.309955
6	3.022536	1.608642	-1.084098
1	2.415792	-1.099459	-1.340947
6	4.002092	-1.095184	0.081222
6	1.593415	-1.442239	0.596800
1	2.404982	2.521151	-1.211416
6	4.346900	2.126632	-0.478098

6	3.280905	1.091042	-2.510290
1	4.743650	-0.715360	-0.631768
1	4.244314	-0.691243	1.076369
1	4.099711	-2.192189	0.112628
1	0.551445	-1.211289	0.329466
1	1.730792	-2.531109	0.528462
1	1.753316	-1.137061	1.643219
1	4.158602	2.522300	0.528970
1	5.101811	1.333551	-0.396580
1	4.771135	2.935802	-1.095258
1	2.341079	0.767372	-2.980026
1	3.724705	1.879370	-3.135591
1	3.972043	0.235959	-2.513501
8	-0.336146	1.321679	2.124862
6	-0.670724	0.165661	2.915814
6	-1.864539	0.584238	3.765959
6	-1.592035	2.075822	3.974488
6	-1.035609	2.481364	2.615342
1	-0.882288	-0.667662	2.234481
1	0.197353	-0.094418	3.537507
1	-2.802454	0.440717	3.212395
1	-1.923280	0.018214	4.701141
1	-2.485623	2.648215	4.243222
1	-0.833597	2.219826	4.754523
1	-1.842052	2.731843	1.909363
1	-0.326357	3.315393	2.660671

Ground State Structure 2

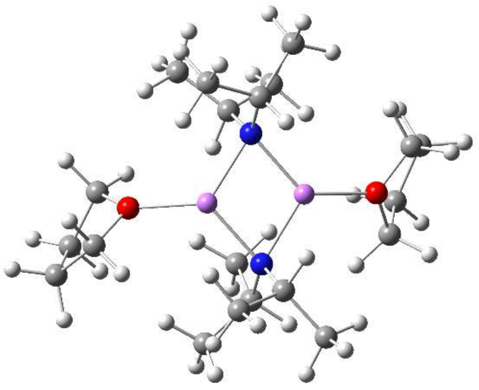

Figure S2. Representative ball and stick model from ground state structure **2**.

M06-2x/mAUG-cc-pVDZ, Structure 2

#m062x/maug-cc-pvDz opt=(calcfc,noeigen) optcyc=50 freq scrf=(pcm,solvent=THF) nosymm

EE = -1063.290742 (Hartree)
ZPE_{298} = -1062.663631
E_{298} = -1062.629326
H_{298} = -1062.628382
G_{298} = -1062.730391
S = 214.698 (cal/mol·K)

3	0.169992	0.718619	0.436067
7	-0.191820	0.928541	2.359577
7	2.100156	0.372950	0.253720
8	-1.013805	0.250537	-1.015406
3	1.740748	0.556887	2.202316
8	2.828813	-0.267523	3.599017
6	-0.602431	-0.741985	-1.959079
6	-1.571374	-1.895347	-1.719322
6	-2.886087	-1.174214	-1.349265
6	-2.445285	0.272652	-1.048696
6	2.718081	-1.503016	4.307316
6	4.033893	-2.210758	4.010660
6	5.045971	-1.048537	3.997521
6	4.169995	0.205155	3.795123
1	-0.704466	-0.335251	-2.981238
1	0.452048	-0.970201	-1.758111
1	-1.671967	-2.544211	-2.598255
1	-1.215697	-2.507099	-0.877667
1	-3.605302	-1.192375	-2.178195
1	-3.365579	-1.638603	-0.477796
1	-2.768611	0.963478	-1.844486
1	-2.789997	0.653221	-0.078863
1	2.602419	-1.297624	5.386800
1	1.823151	-2.023799	3.943219
1	4.273498	-2.978966	4.756360
1	3.978669	-2.688208	3.021344
1	5.590531	-0.983799	4.948529
1	5.784901	-1.163403	3.194074
1	4.188989	0.857817	4.682880
1	4.439107	0.796096	2.909353
6	-0.781379	2.180222	2.814914
6	-0.571643	-0.326327	2.999168
6	2.702302	-0.872238	-0.177510
6	2.549636	1.587488	-0.425015
1	-0.525376	2.914867	2.024472
6	-2.316388	2.225567	2.927207
6	-0.172806	2.778660	4.104501
1	0.224386	-1.050398	2.709808
6	-0.593450	-0.359394	4.538056
6	-1.860934	-0.978862	2.452123
1	2.589888	-1.032550	-1.277210
6	4.207306	-1.027816	0.114397

6	1.959925	-2.022934	0.506376
1	1.715999	2.322896	-0.343757
6	3.743938	2.300925	0.244018
6	2.809309	1.440298	-1.931809
1	-2.789496	1.883520	1.993940
1	-2.682616	1.587016	3.746701
1	-2.661683	3.252546	3.130845
1	0.927273	2.743329	4.055009
1	-0.476751	3.832129	4.228171
1	-0.488974	2.234777	5.006918
1	0.341146	0.051812	4.949389
1	-1.432534	0.225922	4.945108
1	-0.707303	-1.394750	4.900609
1	-1.803292	-1.041584	1.353326
1	-1.990433	-2.001770	2.846236
1	-2.758671	-0.398818	2.714256
1	4.812025	-0.309936	-0.457638
1	4.394016	-0.851399	1.188083
1	4.562837	-2.041040	-0.140408
1	0.874225	-1.955313	0.324218
1	2.313833	-3.006464	0.159320
1	2.117414	-1.971999	1.598132
1	3.516558	2.501919	1.303653
1	4.656665	1.686871	0.205927
1	3.961715	3.267577	-0.242139
1	1.946410	0.974142	-2.433556
1	2.985165	2.423900	-2.394102
1	3.694960	0.816168	-2.132983

M06-2x/mAUG-cc-pVTZ, Structure 2

#m062x/maug-cc-pvTz opt=(calcfc,noeigen) optcyc=50 freq SCF=XQC scrf=(pcm,solvent=THF,read) nosymm

EE = -1063.604635 (Hartree)
ZPE_{298} = -1062.973355
E_{298} = -1062.939344
H_{298} = -1062.938400
G_{298} = -1063.039347
S = 212.460 (cal/mol·K)

3	0.179069	0.712267	0.437967
7	-0.193005	0.916982	2.354453
7	2.094778	0.331595	0.270079
8	-0.979995	0.303082	-1.047973
3	1.728469	0.530495	2.207295
8	2.837639	-0.212961	3.621197
6	-0.588381	-0.731842	-1.951020
6	-1.574074	-1.853980	-1.664755
6	-2.879049	-1.092716	-1.365915

6	-2.410926	0.341030	-1.052811
6	2.743360	-1.450122	4.325488
6	4.048718	-2.155034	3.997829
6	5.057820	-0.995523	3.992752
6	4.189045	0.251876	3.746161
1	-0.684063	-0.369841	-2.979616
1	0.452066	-0.972297	-1.744022
1	-1.669576	-2.547881	-2.496263
1	-1.246484	-2.410831	-0.786978
1	-3.543188	-1.095390	-2.227311
1	-3.415164	-1.531213	-0.527376
1	-2.742351	1.043492	-1.819834
1	-2.731045	0.700128	-0.077229
1	2.652363	-1.253509	5.398298
1	1.849352	-1.966558	3.981973
1	4.298233	-2.930009	4.718385
1	3.979459	-2.607070	3.008435
1	5.565713	-0.918552	4.951745
1	5.814066	-1.119543	3.221099
1	4.246255	0.951204	4.581912
1	4.437049	0.776677	2.825221
6	-0.782102	2.159181	2.823737
6	-0.573350	-0.343547	2.973717
6	2.698707	-0.906729	-0.165631
6	2.527138	1.546758	-0.410926
1	-0.518760	2.901496	2.057559
6	-2.315675	2.206111	2.923829
6	-0.192178	2.732342	4.131257
1	0.216093	-1.060366	2.683312
6	-0.606391	-0.395419	4.509620
6	-1.860721	-0.986672	2.416835
1	2.587576	-1.060941	-1.255115
6	4.202684	-1.059006	0.124162
6	1.964328	-2.061619	0.513648
1	1.694354	2.268841	-0.330916
6	3.715674	2.271649	0.250454
6	2.786518	1.399295	-1.914988
1	-2.777017	1.871971	1.993754
1	-2.683732	1.569899	3.730390
1	-2.655344	3.224143	3.128924
1	0.897619	2.673692	4.108544
1	-0.476627	3.781080	4.253178
1	-0.540934	2.192738	5.011423
1	0.310535	0.020587	4.929044
1	-1.450108	0.167026	4.911849
1	-0.706054	-1.428159	4.853135
1	-1.801119	-1.040541	1.327417
1	-1.992771	-2.002454	2.800451
1	-2.747138	-0.408812	2.679080
1	4.798756	-0.343541	-0.440993

1	4.386345	-0.888376	1.188854
1	4.555489	-2.062099	-0.131717
1	0.887405	-1.992856	0.339963
1	2.315539	-3.032519	0.159400
1	2.128452	-2.018952	1.594804
1	3.488294	2.482337	1.297699
1	4.619868	1.662878	0.219999
1	3.928467	3.223410	-0.244711
1	1.936826	0.923705	-2.408708
1	2.945921	2.376393	-2.373747
1	3.673026	0.793114	-2.111518

M06-2x/JUL-cc-pVDZ, Structure 2

#m062x/jul-cc-pvDz opt=(calcfc,noeigen) optcyc=50 freq SCF=XQC scrf=(pcm,solvent=THF,read) nosymm

EE = -1063.333607 (Hartree)
ZPE298 = -1062.704883
E298 = -1062.670849
H298 = -1062.669905
G298 = -1062.770840
S = 212.435 (cal/mol·K)

3	0.286417	0.472985	0.315744
7	-0.279869	0.339130	2.180369
7	2.216319	0.183533	0.221804
8	-0.810407	0.728185	-1.241793
3	1.717777	0.244592	2.149232
8	2.904003	0.064456	3.657717
6	-0.664501	-0.456294	-2.043560
6	-1.781595	-1.366293	-1.556984
6	-2.929162	-0.363157	-1.388642
6	-2.210992	0.918455	-0.938272
6	2.518428	0.197077	5.038445
6	3.412118	-0.774408	5.795472
6	4.711396	-0.681101	4.992049
6	4.191237	-0.578272	3.561421
1	-0.791135	-0.198601	-3.106492
1	0.351338	-0.836494	-1.880145
1	-2.013181	-2.170589	-2.262917
1	-1.505505	-1.809250	-0.588828
1	-3.429596	-0.202050	-2.351175
1	-3.682693	-0.689531	-0.664062
1	-2.569386	1.808398	-1.470010
1	-2.284760	1.088563	0.144075
1	2.692780	1.235877	5.356074
1	1.449718	-0.029276	5.110916
1	3.530114	-0.497864	6.848289
1	2.997723	-1.790115	5.740521

1	5.262020	0.228138	5.265691
1	5.372038	-1.543472	5.127647
1	4.827681	0.023506	2.901780
1	4.047297	-1.571541	3.112210
6	-1.085881	1.392407	2.761205
6	-0.761829	-1.030187	2.365430
6	2.800046	-0.955521	-0.455115
6	2.634856	1.502451	-0.249745
1	-2.147012	1.328172	2.428132
6	-1.136987	1.435811	4.298782
6	-0.559998	2.734687	2.249766
1	-0.372113	-1.610697	1.504866
6	-0.194745	-1.765346	3.598639
6	-2.285762	-1.206394	2.319525
1	2.600040	-0.929332	-1.551923
6	4.325614	-1.118730	-0.324604
6	2.124889	-2.222610	0.071545
1	1.818364	2.206372	0.024358
6	3.876274	2.084505	0.458720
6	2.783793	1.635411	-1.772253
1	-1.562809	0.514965	4.715103
1	-0.122010	1.560899	4.704569
1	-1.755096	2.277952	4.646845
1	-0.634955	2.793829	1.153861
1	-1.120497	3.579353	2.674519
1	0.500083	2.851671	2.526894
1	0.904855	-1.729941	3.591390
1	-0.544811	-1.315149	4.537353
1	-0.497414	-2.824465	3.595092
1	-2.709362	-0.719788	1.430371
1	-2.545370	-2.273714	2.283645
1	-2.772190	-0.773396	3.204895
1	4.864295	-0.295435	-0.807845
1	4.615536	-1.141821	0.736118
1	4.656760	-2.055961	-0.797764
1	1.043850	-2.203363	-0.127441
1	2.543735	-3.127099	-0.391224
1	2.263100	-2.296383	1.162683
1	3.736794	2.044963	1.548346
1	4.786631	1.522527	0.214084
1	4.036593	3.135629	0.171190
1	1.883178	1.262897	-2.280644
1	2.928262	2.687922	-2.053064
1	3.647418	1.068329	-2.146628

B3LYP/mAUG-cc-pVDZ, Structure 2

#B3LYP/maug-cc-pvDz opt=(calcfc,noeigen) optcyc=50 freq SCF=XQC scrf=(pcm,solvent=THF,read) nosymm

EE = -1063.782250 (Hartree)
ZPE_{298} = -1063.162983
E_{298} = -1063.127392
H_{298} = -1063.126447
G_{298} = -1063.235070
S = 228.615 (cal/mol·K)

3	0.124646	0.224875	0.473101
7	-0.294967	0.408485	2.435404
7	2.136519	0.037827	0.326502
8	-1.086004	0.052956	-1.118674
3	1.716505	0.103111	2.307181
8	3.023797	-0.283213	3.809739
6	-0.747090	-0.595903	-2.361724
6	-2.030825	-1.318501	-2.794017
6	-3.165106	-0.492867	-2.124975
6	-2.416351	0.577365	-1.310306
6	3.363908	-1.617945	4.269859
6	4.579783	-1.447386	5.180754
6	4.351756	-0.044061	5.762458
6	3.774229	0.704928	4.562939
1	-0.450431	0.173990	-3.098174
1	0.109066	-1.255252	-2.171312
1	-2.120811	-1.353238	-3.889482
1	-2.033266	-2.354316	-2.423297
1	-3.837633	-0.028731	-2.860945
1	-3.776050	-1.128837	-1.467663
1	-2.345834	1.531217	-1.865220
1	-2.841823	0.772017	-0.317780
1	2.497566	-2.028315	4.817582
1	3.558250	-2.247305	3.388632
1	4.637574	-2.233692	5.947288
1	5.510465	-1.471958	4.590471
1	3.617665	-0.081127	6.584178
1	5.271106	0.424926	6.142088
1	3.087710	1.519471	4.835750
1	4.571658	1.109627	3.914892
6	-0.819432	1.649978	3.013960
6	-0.946046	-0.811654	2.933889
6	2.724222	-1.147243	-0.288934
6	2.596170	1.339267	-0.185906
1	-1.820241	1.489234	3.470040
6	0.073267	2.238292	4.135713
6	-1.014659	2.730212	1.931250
1	-0.392936	-1.657646	2.472504
6	-0.866341	-1.049473	4.463584
6	-2.418474	-0.986861	2.477405
1	2.600842	-1.139734	-1.400666
6	4.241224	-1.369989	-0.051516

6	1.975687	-2.398438	0.204749
1	1.801087	2.068035	0.089793
6	3.875984	1.924626	0.475575
6	2.731281	1.453209	-1.721624
1	0.227127	1.511409	4.947649
1	1.068257	2.500875	3.731014
1	-0.360092	3.156633	4.575689
1	-1.750126	2.405075	1.177216
1	-1.362031	3.689421	2.355479
1	-0.061817	2.927499	1.405813
1	0.177167	-1.003922	4.815419
1	-1.441726	-0.290818	5.022055
1	-1.277032	-2.037958	4.738027
1	-2.486865	-0.959679	1.377754
1	-2.843450	-1.947684	2.821456
1	-3.063285	-0.183255	2.874091
1	4.850828	-0.580181	-0.514879
1	4.467330	-1.376031	1.030143
1	4.574988	-2.333781	-0.479396
1	0.898349	-2.350154	-0.027178
1	2.375195	-3.317032	-0.257766
1	2.073803	-2.509756	1.300000
1	3.795588	1.894029	1.574474
1	4.785063	1.368991	0.194974
1	4.026316	2.980184	0.180072
1	1.817434	1.105158	-2.230227
1	2.906326	2.501170	-2.020850
1	3.576751	0.859839	-2.109833

B3LYP/mAUG-cc-pVTZ, Structure 2

#B3LYP/maug-cc-pvTz opt=(calcfc,noeigen) optcyc=50 freq SCF=XQC scrf=(pcm,solvent=THF,read) nosymm

EE = -1064.115550 (Hartree)
ZPE_{298} = -1063.493723
E_{298} = -1063.457950
H_{298} = -1063.457006
G_{298} = -1063.566619
S = 230.700 (cal/mol·K)

3	0.131813	0.222371	0.478943
7	-0.294793	0.409870	2.434383
7	2.138044	0.028104	0.329550
8	-1.078064	0.059027	-1.114813
3	1.712024	0.103375	2.303729
8	3.020520	-0.276701	3.808784
6	-0.749775	-0.618083	-2.340191
6	-2.036256	-1.336125	-2.738249
6	-3.150422	-0.408821	-2.194927

6	-2.403994	0.593117	-1.294059
6	3.388228	-1.606468	4.246579
6	4.593975	-1.426382	5.162361
6	4.346356	-0.035291	5.756224
6	3.744765	0.714664	4.574276
1	-0.455380	0.120201	-3.093291
1	0.092418	-1.275275	-2.141884
1	-2.101897	-1.486104	-3.814311
1	-2.082778	-2.313070	-2.259029
1	-3.671389	0.110687	-2.997081
1	-3.890446	-0.973513	-1.631615
1	-2.328828	1.574989	-1.769128
1	-2.843976	0.712253	-0.307817
1	2.539629	-2.042843	4.778720
1	3.599538	-2.210851	3.365618
1	4.657989	-2.209813	5.915184
1	5.517878	-1.433589	4.582489
1	3.629454	-0.092183	6.576555
1	5.252412	0.440486	6.126885
1	3.045570	1.497270	4.863247
1	4.520293	1.148939	3.938384
6	-0.826258	1.643063	3.010128
6	-0.931981	-0.812569	2.930035
6	2.720478	-1.153341	-0.285154
6	2.590831	1.325717	-0.183631
1	-1.821409	1.478311	3.450345
6	0.049539	2.230150	4.141076
6	-1.011998	2.726091	1.934326
1	-0.380064	-1.645908	2.471433
6	-0.847890	-1.051291	4.455869
6	-2.401254	-0.998817	2.479911
1	2.597157	-1.142033	-1.385013
6	4.234321	-1.379641	-0.050776
6	1.974687	-2.401954	0.205924
1	1.805149	2.047539	0.091655
6	3.870851	1.913105	0.467787
6	2.719301	1.437303	-1.716393
1	0.193258	1.507824	4.943444
1	1.036613	2.494163	3.749869
1	-0.390364	3.135871	4.572028
1	-1.728518	2.404132	1.177238
1	-1.366652	3.669560	2.358635
1	-0.061703	2.928562	1.431039
1	0.185948	-0.997824	4.800245
1	-1.423459	-0.305131	5.008193
1	-1.245826	-2.034422	4.723385
1	-2.471239	-0.969181	1.391797
1	-2.808524	-1.955714	2.819909
1	-3.043717	-0.210671	2.879156
1	4.837169	-0.597263	-0.508896

1	4.456865	-1.389488	1.019631
1	4.558527	-2.334103	-0.477697
1	0.908638	-2.352345	-0.023751
1	2.371773	-3.309095	-0.254966
1	2.074327	-2.511335	1.289435
1	3.795232	1.884857	1.555647
1	4.768463	1.363178	0.184465
1	4.013232	2.957194	0.170028
1	1.811534	1.092400	-2.213614
1	2.891503	2.475023	-2.011662
1	3.554300	0.847835	-2.100337

B3LYP/JUL-cc-pVDZ, Structure 2

#B3LYP/jul-cc-pvDz opt=(calcfc,noeigen) optcyc=50 freq SCF=XQC scrf=(pcm,solvent=THF,read) nosymm

EE = -1063.821770 (Hartree)
ZPE_{298} = -1063.200797
E_{298} = -1063.165304
H_{298} = -1063.164360
G_{298} = -1063.272247
S = 227.069 (cal/mol·K)

3	0.107443	0.156578	0.502317
7	-0.302243	0.360515	2.461239
7	2.116895	-0.014749	0.349774
8	-1.112124	-0.012539	-1.085301
3	1.704684	0.092257	2.323365
8	3.026437	-0.235309	3.833170
6	-0.851389	-0.903134	-2.199581
6	-2.217985	-1.188736	-2.813435
6	-2.937633	0.155551	-2.621181
6	-2.399011	0.640785	-1.270424
6	3.462256	-1.553162	4.257038
6	4.706279	-1.326475	5.116287
6	4.436764	0.059152	5.723267
6	3.742338	0.787371	4.572923
1	-0.184535	-0.397681	-2.916250
1	-0.340345	-1.790247	-1.806986
1	-2.145675	-1.491105	-3.865828
1	-2.728698	-1.988324	-2.257647
1	-2.659436	0.855309	-3.421989
1	-4.030839	0.062492	-2.616042
1	-2.246709	1.727518	-1.233588
1	-3.049290	0.346136	-0.434621
1	2.648253	-2.020907	4.832560
1	3.653720	-2.155102	3.359542
1	4.833803	-2.111559	5.872178
1	5.608613	-1.303399	4.488524

1	3.764377	-0.022664	6.589379
1	5.350667	0.574034	6.045287
1	3.015107	1.539875	4.902529
1	4.468996	1.264782	3.896947
6	-0.859305	1.585091	3.038945
6	-0.910503	-0.874605	2.970669
6	2.720506	-1.206866	-0.230661
6	2.544745	1.274750	-0.210005
1	-1.844434	1.392590	3.510527
6	0.028924	2.208411	4.143663
6	-1.109246	2.648854	1.952139
1	-0.342646	-1.703876	2.503025
6	-0.797702	-1.106036	4.498638
6	-2.383213	-1.090494	2.536454
1	2.574142	-1.245778	-1.335920
6	4.246057	-1.383820	-0.015995
6	2.009967	-2.452278	0.326867
1	1.740937	1.996028	0.050433
6	3.818966	1.906375	0.417401
6	2.658153	1.337560	-1.749615
1	0.211333	1.492782	4.956169
1	1.007183	2.496531	3.723977
1	-0.430094	3.112851	4.579995
1	-1.856268	2.295623	1.227204
1	-1.467997	3.599918	2.379144
1	-0.177260	2.861646	1.401977
1	0.248635	-1.029744	4.827639
1	-1.385197	-0.365855	5.063237
1	-1.173525	-2.104920	4.776011
1	-2.465539	-1.061284	1.440645
1	-2.768486	-2.063873	2.884003
1	-3.043094	-0.310257	2.946247
1	4.823460	-0.604248	-0.528575
1	4.493299	-1.337352	1.056890
1	4.587416	-2.357334	-0.407768
1	0.930390	-2.427495	0.117450
1	2.416280	-3.375729	-0.113991
1	2.136423	-2.519797	1.419795
1	3.750681	1.898461	1.514533
1	4.735198	1.369587	0.135032
1	3.932829	2.955545	0.093389
1	1.745081	0.955602	-2.228630
1	2.806642	2.376948	-2.082201
1	3.508292	0.749219	-2.125930

HTCH407/mAUG-cc-pVDZ, Structure 2

#HCTH407/maug-cc-pvDz opt=(calcfc,noeigen) optcyc=50 freq SCF=XQC scrf=(pcm,solvent=THF,read) nosymm

EE = -1063.796068 (Hartree)
ZPE_{298} = -1063.179857
E_{298} = -1063.144334
H_{298} = -1063.143389
G_{298} = -1063.250578
S = 225.597 (cal/mol·K)

3	0.099788	0.241662	0.463171
7	-0.392893	0.502465	2.449498
7	2.154188	-0.029991	0.418891
8	-1.099167	-0.075713	-1.266821
3	1.648671	0.129085	2.410365
8	2.975694	-0.280430	4.038126
6	-0.773834	-0.873048	-2.414440
6	-2.110780	-1.417085	-2.918915
6	-3.144009	-0.387785	-2.410265
6	-2.302272	0.620840	-1.623424
6	3.277604	-1.545818	4.637445
6	4.758005	-1.470561	5.014818
6	5.021706	0.043557	5.170634
6	3.675771	0.689267	4.827399
1	-0.286183	-0.233445	-3.176246
1	-0.058653	-1.643025	-2.095317
1	-2.113504	-1.516954	-4.013854
1	-2.309079	-2.412232	-2.494361
1	-3.689345	0.104434	-3.228271
1	-3.888123	-0.868283	-1.758180
1	-2.041241	1.500421	-2.244332
1	-2.770213	0.973720	-0.694499
1	2.638698	-1.689440	5.531580
1	3.036111	-2.328822	3.904810
1	4.965730	-2.036051	5.934541
1	5.382223	-1.895016	4.214506
1	5.343717	0.314598	6.186402
1	5.804278	0.378115	4.474002
1	3.087948	0.909745	5.740301
1	3.753578	1.610381	4.232310
6	-0.879757	1.769150	2.982626
6	-1.091733	-0.682013	2.935106
6	2.737877	-1.259346	-0.078163
6	2.635165	1.227228	-0.148948
1	-1.856051	1.644265	3.497544
6	0.058622	2.405180	4.035236
6	-1.137255	2.802460	1.869846
1	-0.562993	-1.546328	2.477468
6	-1.030786	-0.944086	4.460867
6	-2.560839	-0.825711	2.461986
1	2.652361	-1.343425	-1.192143
6	4.241801	-1.496912	0.214073

6	1.951606	-2.459631	0.472021
1	1.830450	1.970578	0.055753
6	3.876419	1.866784	0.530385
6	2.819602	1.275871	-1.680139
1	0.233445	1.722755	4.880314
1	1.044109	2.638231	3.591778
1	-0.347678	3.349165	4.443299
1	-1.915623	2.450101	1.174858
1	-1.463428	3.778628	2.270165
1	-0.220790	2.983394	1.279453
1	0.009812	-0.934443	4.822085
1	-1.586338	-0.182743	5.033669
1	-1.467852	-1.924541	4.720628
1	-2.621689	-0.779717	1.363008
1	-3.003604	-1.785713	2.782534
1	-3.204828	-0.024954	2.863219
1	4.882754	-0.762110	-0.292809
1	4.451833	-1.429090	1.295979
1	4.563239	-2.495670	-0.133721
1	0.882402	-2.407133	0.210124
1	2.340319	-3.411886	0.076117
1	2.015953	-2.514519	1.573480
1	3.751401	1.888002	1.624551
1	4.809821	1.322561	0.317823
1	4.019784	2.910513	0.194361
1	1.925723	0.902638	-2.204688
1	2.995189	2.310517	-2.019038
1	3.678896	0.677362	-2.024331

HTCH407/mAUG-cc-pVTZ, Structure 2

#HCTH407/maug-cc-pvTz opt=(calcfc,noeigen) optcyc=50 freq SCF=XQC scrf=(pcm,solvent=THF,read) nosymm

EE = -1064.054876 (Hartree)
ZPE_{298} = -1063.438861
E_{298} = -1063.402938
H_{298} = -1063.401994
G_{298} = -1063.511081
S = 229.592 (cal/mol·K)

3	0.108783	0.241519	0.472332
7	-0.390754	0.508694	2.448150
7	2.155724	-0.033950	0.427534
8	-1.086994	-0.052947	-1.281846
3	1.643422	0.128248	2.408889
8	2.970660	-0.285782	4.050446
6	-0.775010	-0.865949	-2.417641
6	-2.111615	-1.432869	-2.882718

6	-3.137052	-0.378330	-2.420020
6	-2.301508	0.626368	-1.622208
6	3.295070	-1.552851	4.627148
6	4.772031	-1.463239	5.007662
6	5.023004	0.051242	5.159787
6	3.668959	0.682913	4.836736
1	-0.314598	-0.243597	-3.197623
1	-0.053186	-1.619107	-2.103059
1	-2.126651	-1.589827	-3.961952
1	-2.304392	-2.394605	-2.404834
1	-3.638067	0.108138	-3.257802
1	-3.906721	-0.831593	-1.794110
1	-2.062871	1.512535	-2.225756
1	-2.769764	0.953903	-0.694386
1	2.665599	-1.724067	5.511659
1	3.072175	-2.323000	3.887851
1	4.979892	-2.016688	5.924221
1	5.398674	-1.881338	4.218552
1	5.355879	0.321013	6.162784
1	5.784589	0.389597	4.455958
1	3.097331	0.891031	5.751849
1	3.734953	1.602193	4.253377
6	-0.882222	1.770840	2.976256
6	-1.085963	-0.674998	2.928046
6	2.734251	-1.258891	-0.074264
6	2.634336	1.221772	-0.135231
1	-1.853255	1.642993	3.481781
6	0.046571	2.411300	4.032024
6	-1.136042	2.804970	1.866378
1	-0.558116	-1.532318	2.478073
6	-1.034561	-0.938318	4.452443
6	-2.551418	-0.824070	2.450396
1	2.654587	-1.331169	-1.180026
6	4.234675	-1.508091	0.220792
6	1.944575	-2.460251	0.461188
1	1.839547	1.962098	0.071074
6	3.877548	1.858706	0.539713
6	2.816438	1.275809	-1.664682
1	0.215217	1.735552	4.872121
1	1.024260	2.644481	3.594271
1	-0.364976	3.346793	4.430290
1	-1.900639	2.452139	1.170519
1	-1.469042	3.768434	2.267760
1	-0.221843	2.991211	1.291469
1	-0.004006	-0.926056	4.815741
1	-1.593422	-0.185533	5.015894
1	-1.466369	-1.914085	4.701306
1	-2.605671	-0.782870	1.359779
1	-2.985178	-1.778023	2.770862
1	-3.192315	-0.030071	2.845064

1	4.874863	-0.776784	-0.272941
1	4.437330	-1.454345	1.295451
1	4.544691	-2.497902	-0.134706
1	0.885662	-2.402051	0.196438
1	2.331872	-3.400638	0.059291
1	2.005502	-2.523820	1.553283
1	3.755015	1.877231	1.625200
1	4.801276	1.318190	0.322437
1	4.015007	2.894370	0.205566
1	1.926299	0.911966	-2.183639
1	2.993536	2.304322	-1.992913
1	3.666168	0.679452	-2.007235

HTCH407/JUL-cc-pVDZ, Structure 2

#HCTH407/jul-cc-pvDz opt=(calcfc,noeigen) optcyc=50 freq SCF=XQC scrf=(pcm,solvent=THF,read) nosymm

EE = -1063.828480 (Hartree)
ZPE_{298} = -1063.211309
E_{298} = -1063.175585
H_{298} = -1063.174641
G_{298} = -1063.283307
S = 228.707 (cal/mol·K)

3	0.105569	0.247557	0.464544
7	-0.397833	0.511144	2.442967
7	2.154856	-0.031682	0.425294
8	-1.081455	-0.044092	-1.279332
3	1.638922	0.126577	2.409501
8	2.955575	-0.283598	4.049450
6	-0.762510	-0.855709	-2.420011
6	-2.098321	-1.427838	-2.894718
6	-3.139574	-0.402999	-2.393706
6	-2.307388	0.623620	-1.618704
6	3.281962	-1.554607	4.626698
6	4.766533	-1.468845	4.984899
6	5.019180	0.045374	5.156268
6	3.666855	0.687331	4.829788
1	-0.296336	-0.224748	-3.198035
1	-0.034117	-1.609691	-2.099568
1	-2.116390	-1.548695	-3.985038
1	-2.275967	-2.414926	-2.449375
1	-3.687213	0.073024	-3.216572
1	-3.878715	-0.881985	-1.739308
1	-2.078799	1.507942	-2.240158
1	-2.769450	0.962607	-0.684026
1	2.659386	-1.720769	5.524790
1	3.041586	-2.327804	3.886319
1	4.991990	-2.039443	5.894474

1	5.383412	-1.878311	4.174812
1	5.344337	0.302808	6.172009
1	5.794726	0.393856	4.462742
1	3.093460	0.909426	5.747805
1	3.737534	1.605663	4.233099
6	-0.886328	1.775439	2.977649
6	-1.095601	-0.673836	2.927230
6	2.736011	-1.260982	-0.072463
6	2.642283	1.223010	-0.140790
1	-1.862856	1.648604	3.486643
6	0.047155	2.408148	4.036765
6	-1.138502	2.812391	1.866964
1	-0.567370	-1.535313	2.469315
6	-1.033556	-0.938380	4.452715
6	-2.564863	-0.817755	2.454550
1	2.652682	-1.341452	-1.184088
6	4.238909	-1.502915	0.222039
6	1.944155	-2.460157	0.471994
1	1.841360	1.968412	0.059093
6	3.882263	1.860022	0.543592
6	2.832924	1.268979	-1.671358
1	0.215466	1.723605	4.878102
1	1.031437	2.641680	3.597984
1	-0.364275	3.347641	4.443482
1	-1.915475	2.463110	1.173130
1	-1.462421	3.784637	2.272135
1	-0.221601	2.991369	1.282023
1	0.005730	-0.928399	4.810024
1	-1.590374	-0.182036	5.025226
1	-1.467438	-1.919581	4.704905
1	-2.623290	-0.774013	1.357994
1	-3.001865	-1.777005	2.776791
1	-3.206889	-0.019263	2.855448
1	4.879343	-0.774516	-0.289036
1	4.446920	-1.431401	1.301019
1	4.551710	-2.503249	-0.121091
1	0.879074	-2.399739	0.206201
1	2.330446	-3.408596	0.070782
1	2.006779	-2.519406	1.570572
1	3.748177	1.883884	1.633915
1	4.812972	1.314939	0.336111
1	4.025570	2.900099	0.204257
1	1.942228	0.894458	-2.194982
1	3.005829	2.302671	-2.006458
1	3.693219	0.673711	-2.009040

MP2/mAUG-cc-pVDZ, Structure 2

#MP2/maug-cc-pvDz opt=(calcfc,noeigen) optcyc=50 freq SCF=XQC scrf=(pcm,solvent=THF,read) nosymm

EE = -1060.326237 (Hartree)
ZPE$_{298}$ = -1059.696812
E$_{298}$ = -1059.661733
H$_{298}$ = -1059.660788
G$_{298}$ = -1059.768814
S = 227.359 (cal/mol·K)

3	0.076402	0.422117	0.432321
7	-0.335841	0.644670	2.382507
7	2.046688	0.055256	0.313711
8	-1.021104	0.230047	-1.171506
3	1.624694	0.195225	2.284774
8	2.810902	-0.343194	3.758034
6	-0.545973	-0.460175	-2.346640
6	-1.574100	-1.578755	-2.586103
6	-2.865867	-1.042921	-1.905933
6	-2.445454	0.333969	-1.367451
6	3.060519	-1.687933	4.213439
6	4.483277	-1.654479	4.801744
6	4.712179	-0.152256	5.127512
6	3.363312	0.497316	4.789318
1	-0.525373	0.254192	-3.192497
1	0.475499	-0.804761	-2.133407
1	-1.710544	-1.771413	-3.661754
1	-1.242167	-2.514906	-2.110001
1	-3.711637	-0.958474	-2.606150
1	-3.171452	-1.700486	-1.076724
1	-2.644214	1.133777	-2.106726
1	-2.896525	0.607913	-0.403147
1	2.311749	-1.948178	4.986423
1	2.941503	-2.359045	3.350764
1	4.556070	-2.294860	5.694617
1	5.217963	-2.011552	4.063432
1	4.991326	0.018991	6.178796
1	5.503743	0.267497	4.487069
1	2.681211	0.493928	5.662226
1	3.435981	1.518117	4.387257
6	-0.847129	1.909148	2.913137
6	-0.941399	-0.542120	3.000816
6	2.586607	-1.170918	-0.255109
6	2.666432	1.309950	-0.141162
1	-1.827487	1.773871	3.425389
6	0.109346	2.560892	3.937695
6	-1.086305	2.912166	1.771311
1	-0.370062	-1.419968	2.619143
6	-0.847505	-0.615845	4.542885
6	-2.408494	-0.781930	2.572462
1	2.569274	-1.162055	-1.376078
6	4.043116	-1.524190	0.131178

6	1.680165	-2.334381	0.176780
1	1.899666	2.106955	0.010653
6	3.883430	1.790223	0.692681
6	3.025071	1.368260	-1.639450
1	0.301841	1.886561	4.788223
1	1.079640	2.770971	3.445831
1	-0.286986	3.517144	4.333660
1	-1.840833	2.525209	1.063759
1	-1.426691	3.897104	2.143149
1	-0.144466	3.073916	1.210435
1	0.200145	-0.511155	4.874779
1	-1.437592	0.190516	5.015011
1	-1.239168	-1.579551	4.919583
1	-2.468575	-0.886817	1.474945
1	-2.831071	-1.695819	3.033107
1	-3.046944	0.070716	2.868753
1	4.762456	-0.794947	-0.275085
1	4.151168	-1.531683	1.232620
1	4.325270	-2.522360	-0.257631
1	0.633565	-2.160189	-0.132355
1	2.012512	-3.294234	-0.259383
1	1.690661	-2.441878	1.278322
1	3.628161	1.823216	1.767764
1	4.751400	1.119502	0.575413
1	4.194744	2.809562	0.390061
1	2.159136	1.086651	-2.264258
1	3.336233	2.390153	-1.922960
1	3.859655	0.687730	-1.885730

MP2/mAUG-cc-pVTZ, Structure 2

#P MP2/maug-cc-pvTz MAXDISK=3000GB opt=(mndofc,noeigen) optcyc=50 freq=numer SCF=XQC scrf=(pcm,solvent=THF,read) nosymm

EE = -1061.394095 (Hartree)
ZPE_{298} = -1060.763725
E_{298} = -1060.728356
H_{298} = -1060.727412
G_{298} = -1060.837173
S = 231.010 (cal/mol·K)

3	0.160122	0.478280	0.391849
7	-0.263219	0.670868	2.335412
7	2.109550	0.046346	0.280880
8	-0.990138	0.373638	-1.193358
3	1.693030	0.245157	2.242167
8	2.843317	-0.284969	3.755061
6	-0.636121	-0.525439	-2.255115
6	-1.691988	-1.615350	-2.171511

6	-2.958598	-0.834199	-1.767749
6	-2.418251	0.520882	-1.278755
6	3.169144	-1.628944	4.130611
6	4.541254	-1.510627	4.779311
6	4.499869	-0.113671	5.436893
6	3.208978	0.512894	4.891090
1	-0.687018	0.006768	-3.209759
1	0.382758	-0.857560	-2.074746
1	-1.808981	-2.153570	-3.108918
1	-1.418272	-2.327121	-1.394941
1	-3.625542	-0.693340	-2.614732
1	-3.509710	-1.349638	-0.985661
1	-2.645126	1.316483	-1.990920
1	-2.773306	0.807945	-0.293148
1	2.421276	-1.995681	4.840595
1	3.146927	-2.239776	3.232586
1	4.724350	-2.308261	5.494774
1	5.312655	-1.548946	4.013712
1	4.467376	-0.175733	6.521629
1	5.369697	0.473853	5.156716
1	2.404433	0.466691	5.629296
1	3.320832	1.537955	4.549261
6	-0.864807	1.872576	2.887489
6	-0.824762	-0.570011	2.865436
6	2.594671	-1.188928	-0.295085
6	2.727855	1.272897	-0.221404
1	-1.882564	1.680198	3.263217
6	-0.064406	2.478872	4.053183
6	-0.988158	2.941562	1.800261
1	-0.193705	-1.386549	2.481521
6	-0.814524	-0.711348	4.396408
6	-2.244262	-0.867448	2.350144
1	2.510701	-1.194795	-1.398933
6	4.059817	-1.550093	0.009995
6	1.713119	-2.330061	0.209889
1	2.013240	2.086000	-0.010158
6	4.023384	1.711931	0.493431
6	2.959299	1.312224	-1.736868
1	0.068252	1.759982	4.858797
1	0.926978	2.766557	3.692704
1	-0.548495	3.371149	4.461696
1	-1.643873	2.604902	0.996322
1	-1.375429	3.884958	2.191373
1	-0.000923	3.137861	1.371894
1	0.191894	-0.563733	4.789805
1	-1.472836	0.025832	4.859359
1	-1.162121	-1.702195	4.697809
1	-2.240291	-0.928471	1.261248
1	-2.633038	-1.809358	2.746079
1	-2.933213	-0.072475	2.642147

1	4.753762	-0.864410	-0.472257
1	4.233135	-1.503312	1.088023
1	4.294644	-2.559185	-0.340004
1	0.661554	-2.147655	-0.018519
1	1.998769	-3.285189	-0.234553
1	1.804051	-2.422870	1.294862
1	3.881123	1.716080	1.575330
1	4.852886	1.042551	0.271232
1	4.312511	2.721174	0.186376
1	2.045707	1.056616	-2.275575
1	3.274521	2.309026	-2.050325
1	3.737989	0.610206	-2.038634

MP2/JUL-cc-pVDZ, Structure 2

#P MP2/jul-cc-pvDz opt=(calcfc,noeigen) optcyc=50 freq SCF=XQC scrf=(pcm,solvent=THF,read) nosymm

EE = -1060.458981 (Hartree)
ZPE_{298} = -1059.830842
E_{298} = -1059.796120
H_{298} = -1059.795176
G_{298} = -1059.898600
S = 217.674 (cal/mol·K)

3	0.042857	0.480583	0.405393
7	-0.516377	0.648125	2.317517
7	2.020710	0.086186	0.415246
8	-0.908964	0.479380	-1.341552
3	1.445351	0.128192	2.356099
8	2.457137	-0.419766	3.981654
6	-0.330168	-0.396813	-2.354650
6	-1.268038	-1.626602	-2.453657
6	-2.419546	-1.298626	-1.471292
6	-2.335346	0.223148	-1.372089
6	2.921227	-1.780783	4.198719
6	4.455890	-1.694893	4.393253
6	4.755249	-0.176388	4.341617
6	3.396711	0.436385	4.675702
1	-0.284987	0.155730	-3.308606
1	0.687612	-0.632155	-2.017544
1	-1.649420	-1.736472	-3.479792
1	-0.749611	-2.557656	-2.182556
1	-3.399586	-1.649460	-1.828201
1	-2.226438	-1.741674	-0.481152
1	-2.776791	0.712985	-2.260214
1	-2.764969	0.657050	-0.458533
1	2.420018	-2.184896	5.094858
1	2.617492	-2.367364	3.320615
1	4.741439	-2.119454	5.367147

1	4.997189	-2.245280	3.610376
1	5.541584	0.126317	5.049181
1	5.053105	0.129199	3.327053
1	3.188001	0.403291	5.761622
1	3.245085	1.460677	4.307571
6	-0.887623	1.937273	2.909453
6	-1.242126	-0.489326	2.903260
6	2.594520	-1.127069	-0.150174
6	2.659971	1.350046	0.009245
1	-1.852599	1.870472	3.459708
6	0.166924	2.454184	3.916100
6	-1.079082	3.002374	1.813806
1	-0.801534	-1.403516	2.445226
6	-1.086053	-0.654637	4.435460
6	-2.749859	-0.519136	2.553632
1	2.641752	-1.093071	-1.268462
6	4.028585	-1.483952	0.312408
6	1.666019	-2.300160	0.205489
1	1.886803	2.143148	0.137765
6	3.836899	1.816123	0.905460
6	3.088994	1.428749	-1.470508
1	0.340032	1.720264	4.718684
1	1.126912	2.615873	3.389922
1	-0.130137	3.415657	4.377492
1	-1.887393	2.707757	1.123874
1	-1.322479	3.994438	2.236753
1	-0.147915	3.110062	1.226213
1	-0.020935	-0.693916	4.716234
1	-1.553136	0.190581	4.970300
1	-1.578000	-1.580985	4.784841
1	-2.889098	-0.529936	1.461158
1	-3.248769	-1.410839	2.976630
1	-3.262963	0.372251	2.954306
1	4.766274	-0.748474	-0.042868
1	4.073473	-1.508137	1.415777
1	4.333310	-2.474985	-0.074016
1	0.642136	-2.122327	-0.164826
1	2.027166	-3.250805	-0.224759
1	1.611159	-2.423277	1.301912
1	3.529011	1.824287	1.964623
1	4.711588	1.153338	0.810999
1	4.154418	2.842079	0.637814
1	2.254938	1.155878	-2.138189
1	3.408143	2.455252	-1.722483
1	3.936018	0.755501	-1.685794

Ground State Structure 3a

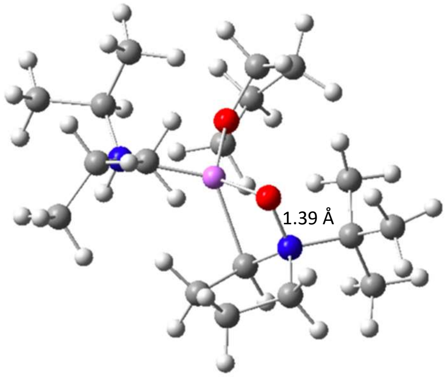

Figure S3. Representative ball and stick model for ground state structure **3a**.

#m062x/jul-cc-pvDz opt=(calcfc,noeigen,z-matrix) optcyc=50 freq scrf=(pcm,solvent=THF)

EE =	-976.504724 (Hartree)
ZPE_{298} =	-975.940862
E_{298} =	-975.912366
H_{298} =	-975.911422
G_{298} =	-975.998941
S =	184.200 (cal/mol·K)

7	2.105378	-0.893292	0.017918
8	1.133508	-0.852379	1.016851
6	3.187872	0.154952	0.316232
6	2.671251	-2.290230	0.020080
6	1.398680	-0.715907	-1.321191
6	1.637865	-3.135311	-0.720089
6	0.776563	-2.107826	-1.504419
1	2.833245	-2.594063	1.057112
1	3.626990	-2.273175	-0.513437
1	1.029288	-3.713969	-0.014412
1	2.139843	-3.847984	-1.385405
1	-0.248839	-2.157727	-1.116672
1	0.716647	-2.350039	-2.574617
1	2.207138	-0.624952	-2.061842
6	3.944175	-0.272989	1.574778
6	4.162815	0.315364	-0.853131
6	2.503457	1.493654	0.570141
1	4.634149	-1.102986	1.378686
1	4.535944	0.578182	1.934479
1	3.234874	-0.564159	2.357090
1	4.579821	-0.637465	-1.198413
1	3.691415	0.824247	-1.700517

1	5.000149	0.935512	-0.507193
1	1.846603	1.746153	-0.266966
1	1.914077	1.455191	1.490885
1	3.278279	2.265422	0.670477
3	-0.214886	-0.018294	-0.028481
8	-0.629662	1.940623	-0.104761
6	-0.471811	2.586608	-1.382921
6	-0.442650	4.088545	-1.098003
6	0.062907	4.139245	0.346864
6	-0.625163	2.919799	0.942751
1	0.462803	2.220112	-1.827893
1	-1.306720	2.287668	-2.031029
1	0.199843	4.626572	-1.802501
1	-1.453738	4.511486	-1.157563
1	1.152670	4.015955	0.382717
1	-0.206179	5.065046	0.866218
1	-0.108406	2.486848	1.807268
1	-1.664000	3.153293	1.224888
7	-2.286246	-0.788328	-0.247623
1	-2.247170	-0.983815	-1.247850
6	-3.246922	0.324566	-0.069768
6	-2.679785	-2.044082	0.449033
1	-2.819910	1.166073	-0.629763
6	-4.635670	0.035439	-0.643877
6	-3.312494	0.736869	1.396558
1	-3.662475	-1.886610	0.916392
6	-2.839642	-3.198730	-0.536117
6	-1.685442	-2.377200	1.555703
1	-4.567895	-0.221486	-1.709756
1	-5.133558	-0.791585	-0.119832
1	-5.274362	0.923632	-0.547805
1	-2.304927	0.917270	1.791112
1	-3.901145	1.657561	1.500296
1	-3.792628	-0.035331	2.013081
1	-3.587683	-2.956436	-1.303495
1	-1.888436	-3.427285	-1.036228
1	-3.169247	-4.103337	-0.009284
1	-0.677795	-2.520902	1.144143
1	-1.626298	-1.553602	2.279590
1	-1.998437	-3.285639	2.088951

Transition Structure 3a

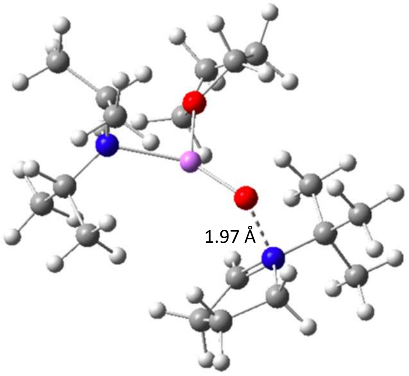

Figure S4. Representative ball and stick model for transition structure **3a**.

#m062x/jul-cc-pvDz opt=(calcfc,noeigen,z-matrix,ts) optcyc=50 freq scrf=(pcm,solvent=THF)

EE = -976.461969 (Hartree)
ZPE_{298} = -975.900013
E_{298} = -975.872102
H_{298} = -975.871158
G_{298} = -975.957052
S = 180.780 (cal/mol·K)

7	2.449180	-0.834107	0.031295
8	0.907915	-0.526466	1.221204
6	3.294165	0.362224	0.392775
6	2.791525	-2.136364	0.652757
6	2.112008	-1.041626	-1.230657
6	1.930730	-3.125658	-0.126064
6	1.790997	-2.478287	-1.517933
1	2.559040	-2.089321	1.713767
1	3.864315	-2.319606	0.486190
1	0.952584	-3.189705	0.359473
1	2.384318	-4.122012	-0.160972
1	0.787663	-2.627356	-1.945041
1	2.503485	-2.910287	-2.239905
1	1.969184	-0.209087	-1.910660
6	3.545630	0.377986	1.897223
6	4.631526	0.265683	-0.353026
6	2.548291	1.623941	-0.028011
1	4.176107	-0.465994	2.205673
1	4.078429	1.303174	2.151938
1	2.580734	0.328800	2.408946
1	5.166827	-0.659794	-0.106015
1	4.485661	0.311287	-1.438015
1	5.262655	1.110531	-0.047229
1	2.505642	1.726536	-1.120576

1	1.537841	1.568862	0.389250
1	3.078793	2.500312	0.366585
3	-0.576994	0.022241	0.465669
8	-0.970703	1.928276	-0.090943
6	-0.761037	2.316584	-1.459030
6	-0.719671	3.839363	-1.445671
6	-0.065880	4.117901	-0.089485
6	-0.678112	3.031205	0.789860
1	0.191123	1.891153	-1.809516
1	-1.580036	1.904338	-2.062369
1	-0.155761	4.246575	-2.291241
1	-1.738442	4.247183	-1.472787
1	1.021138	3.986154	-0.157767
1	-0.273945	5.122552	0.292577
1	-0.004744	2.671680	1.578139
1	-1.621441	3.363268	1.246287
7	-2.549424	-0.507200	-0.048165
1	-2.697372	0.246969	-0.716813
6	-3.311627	-0.122688	1.162024
6	-2.940801	-1.776412	-0.697239
1	-2.817025	0.791293	1.528043
6	-4.769374	0.230055	0.861143
6	-3.202522	-1.181781	2.251874
1	-3.963361	-2.054861	-0.394085
6	-2.930422	-1.574758	-2.208509
6	-1.990561	-2.904441	-0.305741
1	-4.828012	1.020590	0.100526
1	-5.328647	-0.643444	0.499018
1	-5.267110	0.593440	1.769765
1	-2.152516	-1.423725	2.459811
1	-3.664561	-0.806967	3.173408
1	-3.726639	-2.104075	1.965072
1	-3.681030	-0.832816	-2.513882
1	-1.938000	-1.222175	-2.529764
1	-3.142977	-2.516699	-2.728641
1	-0.987699	-2.695610	-0.701873
1	-1.910059	-3.013895	0.780898
1	-2.340352	-3.856880	-0.726962

Ground State Structure 4a

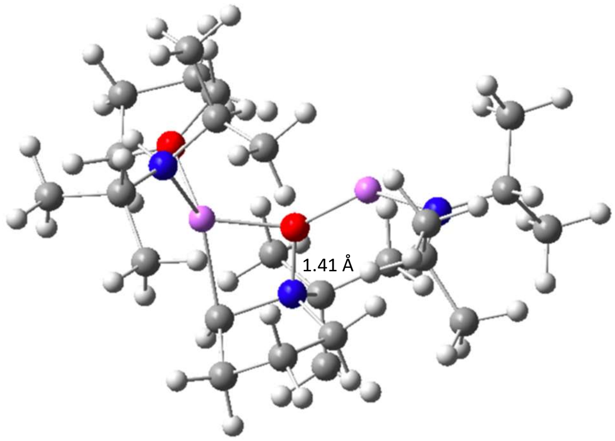

Figure S5. Representative ball and stick model for ground state structure **4a**.

#m062x/jul-cc-pvDz opt=(calcfc,noeigen) optcyc=50 freq scrf=(pcm,solvent=THF)

EE = -1275.793083 (Hartree)
ZPE_{298} = -1275.032728
E_{298} = -1274.993109
H_{298} = -1274.992165
G_{298} = -1275.103448
S = 234.215 (cal/mol·K)

7	0.122453	-1.521212	1.144182
8	-0.007494	-0.726670	-0.009680
6	0.143163	-2.994659	0.699837
6	1.399118	-1.109624	1.828865
6	-1.029801	-1.169450	2.116138
6	0.964119	0.007996	2.757438
6	-0.319033	-0.580244	3.342185
1	2.141572	-0.844214	1.069945
1	1.759480	-1.952227	2.427584
1	0.745343	0.915228	2.179103
1	1.737990	0.236184	3.500227
1	-0.925963	0.182159	3.849009
1	-0.040196	-1.331255	4.107797
1	-1.517406	-2.115378	2.373006
6	1.230373	-3.188342	-0.356939
6	0.424422	-3.921476	1.887264
6	-1.218158	-3.328611	0.089436
1	2.206593	-2.822885	-0.015736
1	1.326748	-4.263935	-0.553293
1	0.964928	-2.692736	-1.296905
1	1.491060	-3.964259	2.134599
1	-0.138896	-3.628888	2.780206

1	0.112977	-4.935173	1.606341
1	-1.988269	-3.459409	0.857268
1	-1.524734	-2.535443	-0.597526
1	-1.127711	-4.271252	-0.465232
3	-1.648039	0.054543	0.499354
8	-3.002371	-0.499182	-0.882611
6	-4.201497	-1.211562	-0.531025
6	-4.787651	-1.686439	-1.854361
6	-3.520755	-1.963073	-2.668511
6	-2.607605	-0.821340	-2.229357
1	-3.934258	-2.058534	0.118129
1	-4.852202	-0.525914	0.024288
1	-5.426902	-2.566975	-1.733611
1	-5.375981	-0.885284	-2.320209
1	-3.089534	-2.930885	-2.381502
1	-3.689945	-1.962839	-3.750111
1	-1.541500	-1.083674	-2.215428
1	-2.751748	0.072168	-2.854621
7	-2.060140	2.053618	0.082256
1	-2.961667	1.780697	-0.307194
6	-1.205782	2.388427	-1.077636
6	-2.300488	3.126818	1.068953
1	-1.041798	1.429822	-1.598296
6	-1.891084	3.340974	-2.058489
6	0.157121	2.914920	-0.649124
1	-2.142500	4.114063	0.603219
6	-3.753144	3.042911	1.525569
6	-1.360412	2.986694	2.262807
1	-2.851736	2.926890	-2.394301
1	-2.077863	4.320070	-1.596275
1	-1.259621	3.500706	-2.942280
1	0.660553	2.213258	0.029819
1	0.791010	3.047196	-1.534628
1	0.071676	3.890346	-0.151015
1	-4.440648	3.216992	0.686418
1	-3.955254	2.045016	1.943169
1	-3.960758	3.788635	2.302267
1	-1.505492	2.000692	2.726059
1	-0.309057	3.075069	1.968735
1	-1.573677	3.765101	3.007716
7	3.184616	0.463121	-0.865651
6	4.295493	0.140449	-1.736087
6	3.532107	1.293475	0.272259
1	5.136535	0.852608	-1.606957
6	4.874883	-1.267110	-1.488815
6	3.865696	0.236450	-3.205328
1	2.616896	1.375331	0.889014
6	4.617220	0.718573	1.204876
6	3.912272	2.741028	-0.100907
1	5.745468	-1.475650	-2.132228

1	5.180123	-1.385825	-0.441964
1	4.100583	-2.021276	-1.701901
1	2.995863	-0.419879	-3.374619
1	3.567136	1.264564	-3.449937
1	4.664052	-0.075515	-3.896203
1	5.584324	0.651913	0.684280
1	4.757124	1.356835	2.090644
1	4.344015	-0.291124	1.543012
1	4.823726	2.761870	-0.716332
1	3.100486	3.202941	-0.677893
1	4.100640	3.354613	0.794033
3	1.416493	-0.115582	-1.023070

Transition Structure 4a

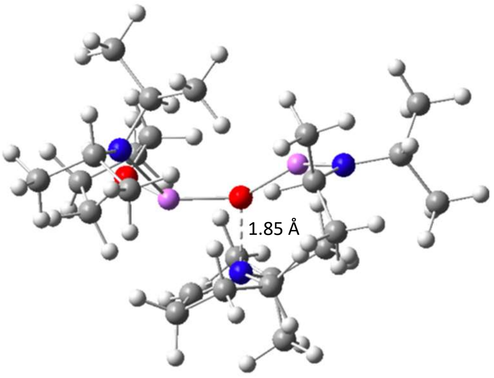

Figure S6. Representative ball and stick model for transition structure **4a**.

#m062x/jul-cc-pvDz opt=(calcfc,noeigen,z-matrix,ts) optcyc=50 freq scrf=(pcm,solvent=THF)

EE = -1275.764152 (Hartree)
ZPE_{298} = -1275.006596
E_{298} = -1274.966275
H_{298} = -1274.965330
G_{298} = -1275.077957
S = 237.042 (cal/mol·K)

7	-0.000233	-1.817152	1.060460
8	0.024009	-0.701051	-0.416914
6	-0.016337	-3.168639	0.371003
6	1.283381	-1.387277	1.687530
6	-0.987306	-1.534667	1.927234
6	0.860866	-0.225379	2.575407
6	-0.546633	-0.627743	3.044178
1	2.016701	-1.119591	0.924970
1	1.646568	-2.228070	2.294914
1	0.810525	0.682613	1.966537

1	1.567488	-0.063379	3.396452
1	-1.194505	0.248540	3.199253
1	-0.506226	-1.162317	4.009153
1	-1.961842	-1.992725	1.814921
6	1.105037	-3.274142	-0.660054
6	0.172477	-4.263380	1.430796
6	-1.367297	-3.341361	-0.317642
1	2.071904	-2.957913	-0.250581
1	1.196408	-4.328550	-0.952525
1	0.873027	-2.676532	-1.544480
1	1.189571	-4.267614	1.840814
1	-0.547072	-4.152074	2.248876
1	0.006019	-5.235233	0.949564
1	-2.172713	-3.543686	0.399608
1	-1.585407	-2.433507	-0.886568
1	-1.307281	-4.198980	-0.999448
3	-1.404212	0.147775	0.235167
8	-3.154488	-0.288161	-0.619758
6	-4.281340	-0.914029	0.017964
6	-5.188963	-1.367671	-1.118174
6	-4.166998	-1.726290	-2.200319
6	-3.125976	-0.626861	-2.022276
1	-3.926005	-1.772619	0.606858
1	-4.742220	-0.182502	0.692354
1	-5.828442	-2.207269	-0.827254
1	-5.826293	-0.538713	-1.452230
1	-3.724694	-2.709848	-1.995400
1	-4.589006	-1.734346	-3.210416
1	-2.099378	-0.925372	-2.270141
1	-3.391256	0.271993	-2.598434
7	-1.827461	2.197758	0.141081
1	-2.825160	2.002403	0.063836
6	-1.349250	2.385814	-1.250975
6	-1.666248	3.357918	1.043343
1	-1.411576	1.390326	-1.718142
6	-2.238977	3.337374	-2.051189
6	0.114789	2.799747	-1.297782
1	-1.567162	4.280534	0.448366
6	-2.916776	3.485735	1.906151
6	-0.426973	3.212037	1.921635
1	-3.282266	2.992357	-2.042547
1	-2.207381	4.356089	-1.640936
1	-1.903555	3.384972	-3.095506
1	0.736839	2.086150	-0.744306
1	0.454098	2.816056	-2.341379
1	0.266541	3.805602	-0.881973
1	-3.801392	3.699085	1.290775
1	-3.089841	2.547831	2.455223
1	-2.802164	4.294077	2.638519
1	-0.565960	2.378854	2.624502

1	0.474633	3.015473	1.330318
1	-0.266309	4.129997	2.502687
7	3.336681	0.300064	-0.832929
6	4.638465	-0.000224	-1.384064
6	3.346699	1.257335	0.256891
1	5.379340	0.790686	-1.141995
6	5.229818	-1.324815	-0.858264
6	4.563888	-0.074558	-2.913843
1	2.306919	1.302497	0.633574
6	4.215375	0.877308	1.473254
6	3.696588	2.697366	-0.174993
1	6.234867	-1.522898	-1.266197
1	5.294288	-1.315369	0.236903
1	4.567831	-2.155592	-1.150096
1	3.800724	-0.815089	-3.203430
1	4.265750	0.897919	-3.327944
1	5.521559	-0.375602	-3.366341
1	5.281266	0.857309	1.200416
1	4.092617	1.604221	2.291161
1	3.942340	-0.118537	1.849541
1	4.723275	2.750465	-0.566467
1	3.011508	3.026375	-0.967666
1	3.623560	3.403657	0.667627
3	1.590882	-0.299354	-1.187533

Transition Structure 4b

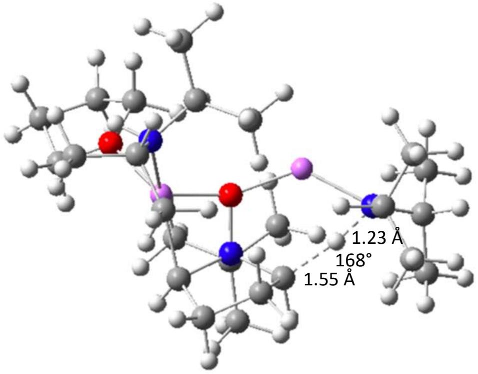

Figure S7. Representative ball and stick model from transition structure **4b**.

#m062x/jul-cc-pvDz opt=(calcfc,noeigen,z-matrix,ts) optcyc=50 freq scrf=(pcm,solvent=THF)

EE = -1275.758866 (Hartree)
ZPE298 = -1275.004687
E298 = -1274.965045
H298 = -1274.964101
G298 = -1275.075447

S = 234.347 (cal/mol·K)

7	0.434125	-1.182870	1.029106
8	-0.041701	-0.553628	-0.157194
6	0.579683	-2.673314	0.704254
6	1.758895	-0.524745	1.401517
6	-0.599501	-0.898781	2.151490
6	1.276148	0.604300	2.307393
6	0.178543	-0.041834	3.158413
1	2.691329	-0.093499	0.239412
1	2.259199	-1.244233	2.069641
1	0.845476	1.431171	1.718715
1	2.103383	1.014138	2.904274
1	-0.462761	0.703739	3.654430
1	0.667616	-0.633213	3.958980
1	-0.838818	-1.872062	2.596145
6	1.439285	-2.825623	-0.551909
6	1.245157	-3.431384	1.857481
6	-0.802055	-3.269591	0.429236
1	1.717483	-3.881732	-0.665703
1	0.887491	-2.522450	-1.449854
1	2.359887	-2.237184	-0.466072
1	2.331223	-3.288347	1.867206
1	0.838065	-3.128859	2.828773
1	1.049318	-4.503090	1.720905
1	-1.365206	-3.441995	1.352849
1	-1.372337	-2.600957	-0.220120
1	-0.670436	-4.236789	-0.074417
3	-1.681531	-0.011723	0.571071
8	-3.143298	-0.906581	-0.530320
6	-4.129418	-1.758168	0.077204
6	-4.801149	-2.481787	-1.082467
6	-3.629784	-2.654927	-2.053370
6	-2.872003	-1.340592	-1.876240
1	-3.623896	-2.463359	0.753759
1	-4.807776	-1.125457	0.661274
1	-5.253709	-3.430226	-0.775143
1	-5.579208	-1.848095	-1.527714
1	-3.003882	-3.501767	-1.743588
1	-3.944221	-2.813373	-3.090079
1	-1.783433	-1.436047	-1.985723
1	-3.239671	-0.567201	-2.567073
7	-2.459828	1.849606	-0.023515
1	-3.345955	1.418720	-0.282578
6	-1.724958	2.075400	-1.284996
6	-2.747952	3.040451	0.801718
1	-1.512709	1.066014	-1.673412
6	-2.550842	2.822627	-2.333039
6	-0.384642	2.752662	-1.030673
1	-2.679397	3.952286	0.185202

6	-4.173820	2.933551	1.331843
6	-1.757745	3.153915	1.957680
1	-3.489257	2.289704	-2.538706
1	-2.796184	3.839344	-1.996742
1	-1.991479	2.905950	-3.274476
1	0.172565	2.205340	-0.260579
1	0.204868	2.766354	-1.956960
1	-0.513871	3.793910	-0.704420
1	-4.901316	2.932865	0.508527
1	-4.289356	1.999852	1.902557
1	-4.405790	3.773126	1.998321
1	-1.841726	2.267274	2.601633
1	-0.723282	3.218011	1.603263
1	-1.973673	4.049004	2.556954
7	3.237179	0.424044	-0.736219
6	4.487286	-0.210388	-1.160267
6	3.364875	1.831953	-0.347247
1	5.245848	0.567381	-1.352211
6	5.066329	-1.142084	-0.085689
6	4.299076	-0.968030	-2.476974
1	2.383594	2.102407	0.078656
6	4.397865	2.088162	0.759032
6	3.613547	2.753544	-1.543374
1	6.022209	-1.573529	-0.415507
1	5.234727	-0.598860	0.852378
1	4.374881	-1.969720	0.127294
1	3.535681	-1.752528	-2.364121
1	3.970460	-0.279574	-3.267031
1	5.233267	-1.449693	-2.799992
1	5.419053	1.872990	0.412788
1	4.368136	3.139973	1.076723
1	4.186204	1.456037	1.630959
1	4.573092	2.520774	-2.027787
1	2.818022	2.637025	-2.291210
1	3.644781	3.806912	-1.229935
3	1.372938	-0.055663	-1.208429

Ground State Structure 5a

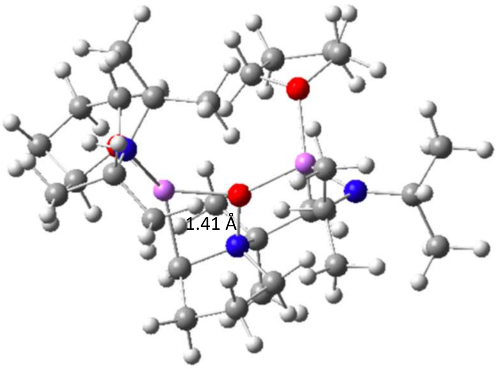

Figure S8. Representative ball and stick model for ground state structure **5a**.

#m062x/jul-cc-pvDz opt=(calcfc,noeigen) optcyc=50 freq scrf=(pcm,solvent=THF)

EE = -1508.183822 (Hartree)
ZPE_{298} = -1507.304900
E_{298} = -1507.258873
H_{298} = -1507.257929
G_{298} = -1507.383272
S = 263.808 (cal/mol·K)

7	0.174581	-0.333144	1.956606
8	0.156216	-0.196417	0.554621
6	0.191703	-1.840237	2.271263
6	1.409870	0.367309	2.466723
6	-1.040155	0.424289	2.538211
6	0.944042	1.780003	2.763381
6	-0.423817	1.534654	3.399281
1	2.201513	0.281809	1.713772
1	1.715929	-0.113030	3.400953
1	0.856109	2.350170	1.831633
1	1.649852	2.297897	3.423952
1	-1.037486	2.447217	3.399427
1	-0.270450	1.250483	4.459294
1	-1.575505	-0.292444	3.171731
6	1.341342	-2.496346	1.504054
6	0.373297	-2.086769	3.772914
6	-1.128973	-2.456178	1.806771
1	2.291917	-1.971638	1.660207
1	1.459874	-3.524371	1.870389
1	1.123583	-2.542651	0.430927
1	1.421125	-2.005588	4.082393
1	-0.232345	-1.400057	4.374862
1	0.046112	-3.110581	3.992407
1	-1.957300	-2.204699	2.477609

1	-1.375645	-2.119366	0.796630
1	-1.015129	-3.548193	1.803408
3	-1.567747	0.597796	0.500489
8	-3.206767	-0.590905	0.052271
6	-4.176333	-0.381002	1.097786
6	-5.426750	-1.120661	0.642619
6	-4.817700	-2.318294	-0.088139
6	-3.621780	-1.680777	-0.788695
1	-3.776294	-0.788567	2.036478
1	-4.314583	0.700632	1.218721
1	-6.070022	-1.403307	1.482169
1	-6.006990	-0.501060	-0.053668
1	-4.474290	-3.069097	0.635737
1	-5.503557	-2.798824	-0.793396
1	-2.783431	-2.377525	-0.910869
1	-3.896445	-1.275925	-1.774586
7	-2.234871	1.967720	-0.919889
1	-3.232994	1.780281	-0.836823
6	-1.805184	1.348674	-2.192505
6	-2.020379	3.426098	-0.794014
1	-2.003933	0.275364	-2.058232
6	-2.619289	1.815873	-3.399695
6	-0.308153	1.525710	-2.411459
1	-1.842881	3.864536	-1.789028
6	-3.274488	4.065064	-0.208974
6	-0.810423	3.710039	0.090320
1	-3.691453	1.643236	-3.233583
1	-2.465566	2.884240	-3.602769
1	-2.318533	1.258345	-4.296704
1	0.254138	1.205837	-1.525128
1	0.019743	0.922638	-3.267434
1	-0.057117	2.575148	-2.620879
1	-4.134681	3.931252	-0.879105
1	-3.509583	3.603776	0.762151
1	-3.124457	5.139635	-0.048783
1	-1.004791	3.332357	1.104578
1	0.092103	3.216180	-0.290639
1	-0.618396	4.790139	0.145720
7	3.355066	0.415111	-0.389697
6	4.767277	0.137445	-0.481505
6	3.010346	1.824319	-0.325959
1	5.328034	0.991514	-0.920169
6	5.428099	-0.164613	0.881593
6	5.011343	-1.062550	-1.402308
1	1.928286	1.862620	-0.089844
6	3.705089	2.649288	0.775575
6	3.175535	2.561520	-1.671249
1	6.511750	-0.343908	0.784123
1	5.276635	0.660722	1.586739
1	4.966382	-1.066765	1.314194

1	4.438785	-1.925908	-1.023912
1	4.663609	-0.840463	-2.420498
1	6.073427	-1.349039	-1.443064
1	4.786342	2.721921	0.584881
1	3.301699	3.673258	0.810576
1	3.561761	2.187639	1.761192
1	4.233198	2.582127	-1.974470
1	2.607006	2.039232	-2.451914
1	2.822793	3.604303	-1.614378
3	1.710574	-0.479913	-0.506752
8	1.034882	-1.522996	-2.015878
6	-0.328589	-1.949596	-1.997636
6	-0.283024	-3.485528	-1.889185
6	1.174822	-3.840276	-2.267676
6	1.740599	-2.510819	-2.768863
1	-0.800730	-1.447441	-1.147610
1	-0.811464	-1.633796	-2.937328
1	-0.518807	-3.819134	-0.871493
1	-1.006717	-3.946639	-2.569906
1	1.733655	-4.176434	-1.386211
1	1.234985	-4.624538	-3.029246
1	2.809265	-2.367851	-2.589919
1	1.525438	-2.364412	-3.839498

Transition Structure 5a

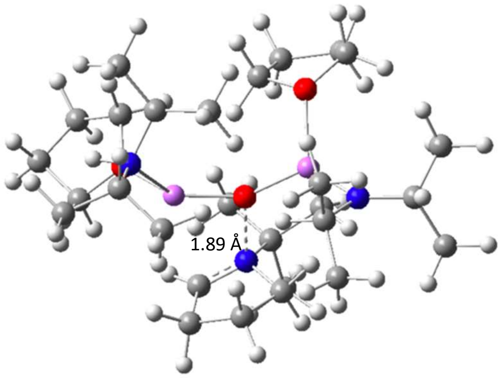

Figure S9. Representative ball and stick model for transition structure **5a**.

#m062x/jul-cc-pvDz opt=(calcfc,noeigen,z-matrix,ts) optcyc=50 freq scrf=(pcm,solvent=THF)

EE = -1508.152150 (Hartree)
ZPE_{298} = -1507.275773
E_{298} = -1507.229178
H_{298} = -1507.228234
G_{298} = -1507.356353
S = 269.649 (cal/mol·K)

7	0.078708	-0.696526	2.226972
8	0.126386	-0.204455	0.399096
6	0.237244	-2.196386	2.113639
6	1.270373	0.100048	2.636634
6	-0.984015	-0.212301	2.870508
6	0.681545	1.471248	2.954457
6	-0.762569	1.173961	3.405242
1	2.007664	0.119409	1.831297
1	1.681894	-0.370218	3.540966
1	0.684875	2.076396	2.043014
1	1.267566	1.990412	3.720517
1	-1.470535	1.921913	3.010446
1	-0.862679	1.205957	4.502257
1	-1.925337	-0.746996	2.855695
6	1.419565	-2.563855	1.218721
6	0.474689	-2.770714	3.517927
6	-1.041513	-2.784457	1.523684
1	2.321294	-1.991554	1.466710
1	1.644071	-3.628022	1.374349
1	1.168473	-2.408613	0.166533
1	1.449740	-2.472165	3.921407
1	-0.313497	-2.456288	4.210063
1	0.465168	-3.865660	3.448156
1	-1.882169	-2.743834	2.227535
1	-1.293380	-2.234732	0.612327
1	-0.859353	-3.839888	1.283780
3	-1.508694	0.473487	0.370315
8	-3.184762	-0.600859	0.150009
6	-4.244808	-0.432851	1.107851
6	-5.486569	-1.004874	0.438031
6	-4.884409	-2.156308	-0.370615
6	-3.582351	-1.538373	-0.868139
1	-3.995193	-0.994843	2.020303
1	-4.317136	0.634516	1.353645
1	-6.238908	-1.328465	1.164268
1	-5.937554	-0.260305	-0.231141
1	-4.673197	-3.011188	0.285003
1	-5.522722	-2.493674	-1.193367
1	-2.775634	-2.268921	-1.005181
1	-3.733207	-0.988688	-1.809288
7	-2.227308	2.016084	-0.824387
1	-3.227013	1.825239	-0.766529
6	-1.773874	1.434859	-2.111785
6	-2.029245	3.472794	-0.661958
1	-1.884927	0.346318	-1.987639
6	-2.649851	1.857363	-3.291035
6	-0.297524	1.708743	-2.364544
1	-1.897531	3.943491	-1.649726
6	-3.272538	4.071493	-0.014460

6	-0.792285	3.759611	0.184247
1	-3.702667	1.602473	-3.105838
1	-2.581115	2.938546	-3.473284
1	-2.328926	1.341493	-4.205718
1	0.298518	1.389725	-1.500133
1	0.033683	1.139975	-3.242870
1	-0.113877	2.774264	-2.561866
1	-4.152979	3.947735	-0.659791
1	-3.470103	3.574641	0.947614
1	-3.132408	5.142074	0.177769
1	-0.953790	3.391820	1.208042
1	0.101075	3.268802	-0.218479
1	-0.605890	4.841138	0.231872
7	3.315060	0.621744	-0.247814
6	4.740173	0.465429	-0.395324
6	2.870691	1.983382	-0.018536
1	5.228229	1.404483	-0.738529
6	5.459726	0.057390	0.910150
6	5.047241	-0.598102	-1.455266
1	1.787828	1.906184	0.191054
6	3.486823	2.709523	1.194819
6	3.005326	2.897463	-1.256217
1	6.549614	-0.039802	0.770814
1	5.276221	0.788493	1.706319
1	5.064636	-0.914050	1.247807
1	4.554470	-1.540883	-1.164544
1	4.647334	-0.290992	-2.431645
1	6.126359	-0.792705	-1.555579
1	4.562393	2.886620	1.041198
1	3.006171	3.688246	1.353805
1	3.365311	2.112342	2.108874
1	4.062985	3.043115	-1.523081
1	2.498014	2.435910	-2.113883
1	2.566676	3.893880	-1.080600
3	1.694761	-0.346883	-0.527368
8	1.237061	-1.409741	-2.195862
6	-0.116688	-1.875154	-2.216327
6	-0.032611	-3.415036	-2.208980
6	1.452221	-3.709382	-2.522809
6	1.986487	-2.347215	-2.964697
1	-0.589786	-1.435174	-1.329905
1	-0.602239	-1.511483	-3.137958
1	-0.319070	-3.824681	-1.233054
1	-0.700103	-3.846562	-2.963197
1	1.980773	-4.039737	-1.620318
1	1.580502	-4.475130	-3.295329
1	3.047682	-2.183859	-2.757756
1	1.793265	-2.174766	-4.036697

Transition Structure 5b

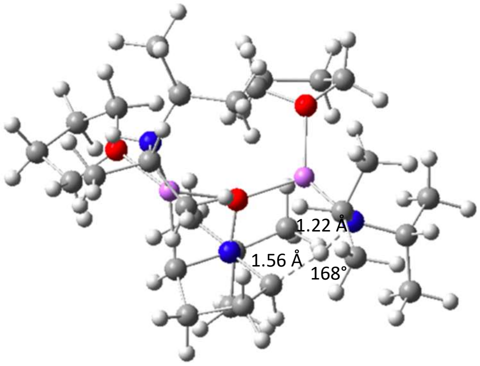

Figure S10. Representative ball and stick model from transition structure **5b**.

#m062x/jul-cc-pvDz opt=(calcfc,noeigen,z-matrix,ts) optcyc=50 freq scrf=(pcm,solvent=THF)

EE = -1508.154057 (Hartree)
ZPE_{298} = -1507.281239
E_{298} = -1507.235207
H_{298} = -1507.234263
G_{298} = -1507.361205
S = 267.172 (cal/mol·K)

7	0.345960	-0.291087	1.821474
8	0.127714	-0.170212	0.419397
6	0.425890	-1.796452	2.109064
6	1.644689	0.429140	2.169698
6	-0.833914	0.419309	2.532824
6	1.141560	1.799647	2.604811
6	-0.159556	1.515908	3.363711
1	2.626254	0.463204	0.957282
1	2.010338	-0.065771	3.082433
1	0.938667	2.444769	1.735904
1	1.895189	2.309943	3.223080
1	-0.794413	2.412899	3.456771
1	0.103024	1.198740	4.394280
1	-1.284563	-0.329457	3.197879
6	1.524918	-2.416010	1.239427
6	0.745089	-2.066818	3.583840
6	-0.907796	-2.456787	1.749198
1	2.453769	-1.836091	1.303604
1	1.727494	-3.434544	1.596384
1	1.202601	-2.497149	0.193479
1	1.812951	-1.955657	3.799046
1	0.179637	-1.402543	4.246242
1	0.464601	-3.103368	3.813015
1	-1.687801	-2.231821	2.484977

1	-1.241171	-2.123543	0.762351
1	-0.762856	-3.545826	1.731455
3	-1.588056	0.580078	0.571949
8	-3.243036	-0.627821	0.220464
6	-4.104666	-0.564068	1.376180
6	-5.207316	-1.587508	1.127284
6	-4.469624	-2.640019	0.297361
6	-3.589015	-1.764287	-0.584755
1	-3.505382	-0.807111	2.263091
1	-4.471686	0.465648	1.471936
1	-5.622887	-1.980351	2.060848
1	-6.021585	-1.143374	0.540110
1	-3.844450	-3.268939	0.945291
1	-5.135273	-3.284558	-0.286121
1	-2.666687	-2.264286	-0.903531
1	-4.132833	-1.416639	-1.477005
7	-2.397165	1.940708	-0.800343
1	-3.373966	1.704652	-0.633675
6	-2.052108	1.369815	-2.118586
6	-2.231571	3.402236	-0.648945
1	-2.222703	0.288256	-2.004261
6	-2.957682	1.856911	-3.251283
6	-0.576863	1.580355	-2.438032
1	-2.145497	3.873991	-1.641052
6	-3.463575	3.972284	0.044823
6	-0.972120	3.712259	0.153387
1	-4.013314	1.674072	-3.008364
1	-2.825413	2.930126	-3.443364
1	-2.722776	1.319899	-4.179978
1	0.047503	1.211620	-1.614413
1	-0.309367	1.037947	-3.354027
1	-0.352747	2.644273	-2.600274
1	-4.364302	3.824227	-0.566517
1	-3.609386	3.470680	1.013503
1	-3.343530	5.046497	0.230919
1	-1.068187	3.291819	1.164588
1	-0.082374	3.269411	-0.310721
1	-0.820699	4.797674	0.230667
7	3.179227	0.552247	-0.123805
6	4.592887	0.184734	-0.113951
6	2.876456	1.910696	-0.578284
1	5.194661	0.986280	-0.575945
6	5.114399	-0.009956	1.314814
6	4.825666	-1.079772	-0.944121
1	1.794825	2.027785	-0.391782
6	3.578766	3.012750	0.225091
6	3.109773	2.089288	-2.080275
1	6.175994	-0.295067	1.314046
1	4.998789	0.912757	1.898374
1	4.543565	-0.800359	1.823919

1	4.206543	-1.902962	-0.552240
1	4.539528	-0.899978	-1.989153
1	5.877964	-1.399421	-0.919402
1	4.667771	2.986683	0.075298
1	3.222894	4.005553	-0.086176
1	3.370316	2.888528	1.294979
1	4.173902	1.961792	-2.328648
1	2.534743	1.344125	-2.647988
1	2.806482	3.093096	-2.412343
3	1.609959	-0.491921	-0.615938
8	1.079574	-1.402152	-2.233230
6	-0.293080	-1.815327	-2.206966
6	-0.263902	-3.351137	-2.102006
6	1.188613	-3.726993	-2.478488
6	1.779996	-2.406446	-2.972460
1	-0.751803	-1.314543	-1.347578
1	-0.778242	-1.487456	-3.140179
1	-0.504415	-3.683111	-1.084766
1	-0.992809	-3.801877	-2.783874
1	1.739140	-4.079339	-1.598312
1	1.237501	-4.506758	-3.245279
1	2.849011	-2.286340	-2.775209
1	1.586113	-2.257272	-4.046108

Ground State Structure 6a

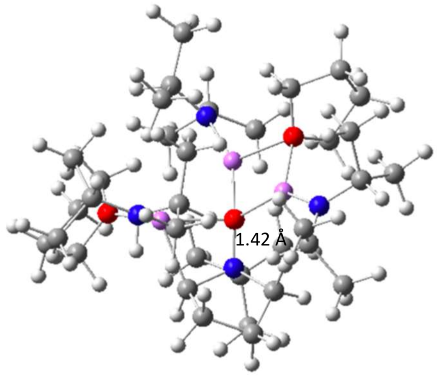

Figure S11. Representative ball and stick model for ground state structure **6a**.

#m062x/jul-cc-pVDZ opt=(calcfc,noeigen) optcyc=50 freq scrf=(pcm,solvent=THF,read) nosymm

EE = -1807.466241 (Hartree)
ZPE_{298} = -1806.389671
E_{298} = -1806.332829
H_{298} = -1806.331885
G_{298} = -1806.477428
S = 306.322 (cal/mol·K)

7	0.830806	-1.975645	0.427973
6	0.944268	-1.828543	1.953738
6	2.243151	-2.200258	-0.108621
6	-0.116190	-3.115488	-0.001452
1	0.625353	-2.797357	2.357825
1	2.579118	-1.244554	-0.515409
1	2.232356	-2.930082	-0.919569
6	2.450573	-1.729331	2.191532
6	3.095025	-2.577221	1.089569
1	4.150322	-2.343142	0.895162
1	3.009991	-3.646315	1.323256
1	2.832082	-0.696909	2.103842
1	2.702298	-2.079188	3.201692
6	0.484350	-4.480947	0.348186
6	-1.460648	-2.949710	0.705297
6	-0.342344	-3.049313	-1.512078
1	0.731589	-4.569898	1.409642
1	-0.271143	-5.240400	0.113949
1	1.375638	-4.710699	-0.246227
1	-1.384561	-3.139564	1.780179
1	-1.880670	-1.949775	0.547843
1	-2.160307	-3.678342	0.274011
1	0.589238	-2.967851	-2.082054
1	-0.848659	-3.971153	-1.823706
1	-0.993792	-2.217278	-1.787606
7	3.205913	0.723271	-1.764878
6	3.631909	1.273182	-3.040774
6	4.305590	0.561858	-0.828657
1	4.732494	1.226797	-3.151869
6	3.060652	0.463605	-4.210676
6	3.246225	2.755237	-3.200251
1	3.863592	0.276305	0.145891
6	5.277092	-0.576295	-1.208065
6	5.127738	1.836997	-0.556083
1	3.313427	0.903975	-5.188037
1	3.441175	-0.566288	-4.178064
1	1.967140	0.421950	-4.128733
1	2.158086	2.864129	-3.077508
1	3.728715	3.361007	-2.423121
1	3.530228	3.159940	-4.185523
1	5.782426	-0.352325	-2.159044
1	6.054588	-0.721325	-0.441314
1	4.728407	-1.519723	-1.333376
1	5.702540	2.133465	-1.445625
1	4.466874	2.672069	-0.289985
1	5.843503	1.679274	0.265095
3	1.399288	0.260850	-1.416424
8	0.338051	-0.763625	-0.116099
3	-0.270195	-0.127068	1.809680

7	0.844222	1.370651	2.767774
1	1.290389	0.578264	3.232451
6	1.842615	1.904258	1.815135
6	0.397352	2.326039	3.809503
6	1.266873	3.065189	1.013961
6	3.163796	2.267883	2.493267
1	2.035783	1.083018	1.108687
6	-1.067323	2.697255	3.604425
6	0.646041	1.743056	5.196070
1	0.992822	3.247393	3.728918
1	0.327368	2.771267	0.527458
1	1.065247	3.938094	1.650012
1	1.981753	3.369868	0.239144
1	3.033136	3.080615	3.220639
1	3.577032	1.397103	3.021502
1	3.900316	2.594993	1.751165
1	-1.217362	3.128297	2.607022
1	-1.699968	1.802843	3.688468
1	-1.384430	3.435941	4.353173
1	0.130218	0.778676	5.305453
1	1.719294	1.579991	5.362777
1	0.273097	2.418934	5.975622
3	-1.289010	-0.156668	-1.022772
7	-3.163969	0.180505	-1.200064
6	-3.618346	1.404001	-0.565334
6	-4.281815	-0.631857	-1.644763
6	-2.451807	2.082295	0.154191
6	-4.260077	2.435352	-1.516539
1	-4.390674	1.203859	0.215114
6	-3.848086	-1.636547	-2.709619
6	-4.999482	-1.399996	-0.515910
1	-5.063965	-0.005882	-2.124278
1	-2.049408	1.452612	0.961329
1	-1.643963	2.298530	-0.562257
1	-2.750730	3.039285	0.604876
1	-3.511870	2.827160	-2.220606
1	-5.077286	1.995546	-2.100710
1	-4.673766	3.284557	-0.951261
1	-3.057725	-2.293930	-2.316120
1	-4.688203	-2.274630	-3.019127
1	-3.465843	-1.118157	-3.598955
1	-5.343408	-0.723811	0.278678
1	-5.880208	-1.937502	-0.899540
1	-4.312469	-2.134214	-0.067549
8	-0.176788	0.605254	-2.607937
6	-0.447396	-0.318980	-3.690914
6	-0.606191	0.524645	-4.966732
1	-1.368781	-0.863804	-3.458651
1	0.393091	-1.020159	-3.729535
6	-0.311164	1.965290	-4.517518

1	-1.630632	0.438712	-5.345214
1	0.077628	0.193601	-5.755287
6	-0.609877	1.916683	-3.028610
1	0.739108	2.223381	-4.690175
1	-0.940381	2.699904	-5.030243
1	-0.058159	2.648507	-2.427508
1	-1.686034	1.995909	-2.828254
8	-1.959108	-0.445093	2.751985
6	-1.880133	-1.216849	3.965077
6	-3.315889	-1.618190	4.308929
1	-1.415069	-0.600598	4.744210
1	-1.233425	-2.080954	3.768661
6	-4.007369	-1.570328	2.944084
1	-3.768393	-0.881088	4.984385
1	-3.361930	-2.601363	4.788630
6	-3.323254	-0.374732	2.302600
1	-3.794701	-2.479848	2.365903
1	-5.093145	-1.443978	3.011961
1	-3.312200	-0.392189	1.207521
1	-3.767435	0.572621	2.651604

Transition Structure 6a

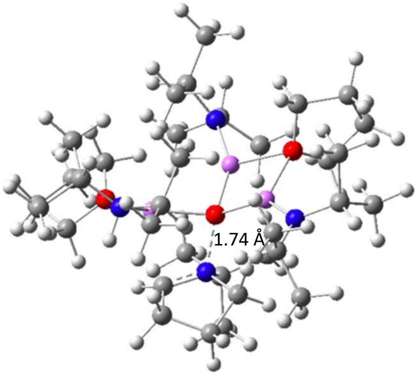

Figure S12. Representative ball and stick model for transition structure **6a**.

#m062x/jul-cc-pVDZ opt=(calcfc,noeigen,ts) optcyc=50 freq scrf=(pcm,solvent=THF,read) nosymm

EE = -1807.447731 (Hartree)
ZPE_{298} = -1806.373475
E_{298} = -1806.316412
H_{298} = -1806.315468
G_{298} = -1806.461426
S = 307.195 (cal/mol·K)

7	-0.210682	0.151036	0.064106
6	-0.122009	0.149426	1.427219

6	1.208172	0.149899	-0.468147
6	-1.139921	-0.877112	-0.582972
1	-0.933173	-0.283431	1.993934
1	1.451351	1.164827	-0.776834
1	1.268000	-0.496166	-1.345039
6	1.299907	0.232323	1.910370
6	2.077839	-0.299157	0.697630
1	3.092379	0.107747	0.604468
1	2.137897	-1.393864	0.741088
1	1.653439	1.251264	2.156827
1	1.454671	-0.379016	2.808762
6	-0.543360	-2.265807	-0.319444
6	-2.532186	-0.790088	0.036417
6	-1.255130	-0.652799	-2.090585
1	-0.394252	-2.433786	0.751905
1	-1.246137	-3.017674	-0.697972
1	0.410614	-2.410299	-0.841208
1	-2.547162	-1.111940	1.083995
1	-2.935696	0.224825	-0.042991
1	-3.189595	-1.465717	-0.526395
1	-0.305301	-0.374544	-2.557050
1	-1.599416	-1.586500	-2.553708
1	-1.995905	0.113325	-2.326826
7	2.080093	2.990593	-2.264076
6	2.502340	3.481059	-3.564825
6	3.181610	2.905490	-1.321751
1	3.603946	3.439644	-3.674393
6	1.939614	2.613155	-4.696583
6	2.105158	4.950772	-3.799716
1	2.750263	2.658575	-0.333548
6	4.181312	1.771763	-1.639729
6	3.971851	4.212554	-1.106459
1	2.198251	3.005054	-5.693093
1	2.323492	1.587830	-4.609186
1	0.846168	2.573971	-4.617681
1	1.015544	5.054831	-3.690734
1	2.575465	5.598802	-3.049626
1	2.392899	5.309742	-4.801821
1	4.683764	1.954570	-2.600940
1	4.960678	1.683818	-0.865863
1	3.652493	0.811613	-1.714957
1	4.510638	4.504664	-2.019417
1	3.293444	5.033531	-0.837516
1	4.717218	4.097318	-0.304252
3	0.249904	2.535767	-1.895946
8	-0.817799	1.645697	-0.574542
3	-1.265121	2.083096	1.210133
7	-0.168470	3.380946	2.416393
1	0.275955	2.644055	2.964196
6	0.842801	3.845972	1.438196

6	-0.654129	4.417038	3.360295
6	0.261675	4.914603	0.520942
6	2.146696	4.294072	2.096889
1	1.053389	2.969184	0.809081
6	-2.131722	4.709425	3.124761
6	-0.388973	3.990380	4.799797
1	-0.093027	5.345704	3.183045
1	-0.650839	4.549115	0.029668
1	0.016995	5.835220	1.068755
1	0.994320	5.160304	-0.257428
1	1.996083	5.178064	2.731782
1	2.561617	3.489706	2.720635
1	2.889534	4.549833	1.332260
1	-2.308637	4.980234	2.076426
1	-2.735651	3.821146	3.354085
1	-2.465252	5.540035	3.761284
1	-0.890084	3.035890	5.017571
1	0.687387	3.865036	4.978544
1	-0.771587	4.740203	5.503278
3	-2.357745	2.080365	-1.590920
7	-4.252798	2.346531	-1.761465
6	-4.696666	3.574588	-1.133499
6	-5.365454	1.507082	-2.159103
6	-3.500792	4.288904	-0.500465
6	-5.416527	4.568570	-2.067084
1	-5.416809	3.383700	-0.301010
6	-4.920396	0.453661	-3.171265
6	-6.070258	0.787008	-0.989633
1	-6.157645	2.098129	-2.667872
1	-2.983614	3.630833	0.213688
1	-2.778920	4.590208	-1.274024
1	-3.803376	5.196744	0.040322
1	-4.738333	4.900458	-2.866784
1	-6.299142	4.117012	-2.536210
1	-5.753612	5.456272	-1.509858
1	-4.105230	-0.155564	-2.751424
1	-5.747109	-0.223455	-3.430534
1	-4.564266	0.928628	-4.094879
1	-6.404638	1.495551	-0.219311
1	-6.955238	0.233743	-1.339843
1	-5.377669	0.071948	-0.519156
8	-1.284110	2.811526	-3.256583
6	-1.586219	1.896654	-4.335158
6	-1.715848	2.741672	-5.614577
1	-2.524274	1.382534	-4.098306
1	-0.770424	1.167908	-4.377067
6	-1.398183	4.176152	-5.160782
1	-2.736965	2.674733	-6.005916
1	-1.028635	2.398395	-6.395036
6	-1.704552	4.124631	-3.672833

1	-0.341799	4.414103	-5.325628
1	-2.010208	4.924619	-5.674586
1	-1.151920	4.850983	-3.065379
1	-2.782092	4.217833	-3.481142
8	-2.912356	1.640994	2.163142
6	-2.929103	0.968925	3.433826
6	-4.388495	0.960766	3.883701
1	-2.257960	1.497949	4.121791
1	-2.555007	-0.055219	3.294032
6	-5.128135	0.972829	2.543241
1	-4.621001	1.872052	4.449921
1	-4.619910	0.092111	4.508545
6	-4.259968	1.924412	1.739062
1	-5.120256	-0.026040	2.084911
1	-6.164798	1.317137	2.620578
1	-4.300937	1.787863	0.652657
1	-4.493870	2.973608	1.982918

Transition Structure 6b

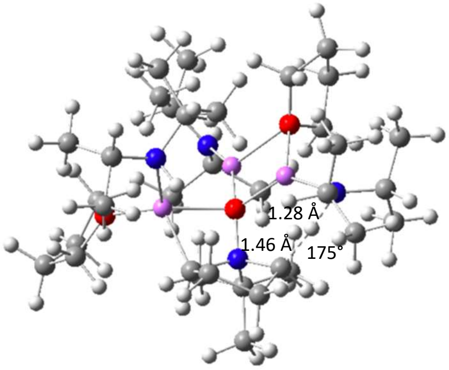

Figure S13. Representative ball and stick model from transition structure **6b**.

#m062x/jul-cc-pVDZ opt=(calcfc,noeigen,ts) optcyc=50 freq scrf=(pcm,solvent=THF,read) nosymm

EE = -1807.438052 (Hartree)
ZPE_{298} = -1806.366468
E_{298} = -1806.310326
H_{298} = -1806.309381
G_{298} = -1806.452316
S = 300.832 (cal/mol·K)

7	-0.067468	0.330771	0.326169
6	-0.148715	0.400003	1.861701
6	1.410064	0.201888	-0.083008
6	-0.931322	-0.790697	-0.279153
1	-0.350922	-0.632236	2.181045
1	2.101622	1.259400	-0.823040

1	1.480520	-0.642458	-0.775874
6	1.303038	0.672774	2.246482
6	2.137740	-0.097852	1.224730
1	3.187413	0.227542	1.186471
1	2.130591	-1.170023	1.495913
1	1.579571	1.743382	2.177371
1	1.488455	0.368334	3.286627
6	-0.469990	-2.167092	0.214742
6	-2.390625	-0.593535	0.131753
6	-0.806956	-0.762507	-1.806548
1	-0.777848	-2.360471	1.246635
1	-0.945275	-2.924316	-0.422228
1	0.614376	-2.293041	0.137312
1	-2.481022	-0.551002	1.221817
1	-2.838309	0.309499	-0.299864
1	-2.967086	-1.456010	-0.231205
1	0.149544	-1.173775	-2.146282
1	-1.605462	-1.383275	-2.230456
1	-0.907835	0.250966	-2.208771
7	2.682307	2.251749	-1.395844
6	3.121557	2.042846	-2.771099
6	3.714975	2.671454	-0.454483
1	4.208334	1.850928	-2.811441
6	2.431988	0.803708	-3.351632
6	2.844684	3.261606	-3.661253
1	3.226634	2.622033	0.536263
6	4.932474	1.730294	-0.387257
6	4.206644	4.109188	-0.657260
1	2.666099	0.683678	-4.418083
1	2.746183	-0.101802	-2.816987
1	1.337601	0.879531	-3.250163
1	1.765069	3.463204	-3.679324
1	3.345139	4.157042	-3.274240
1	3.183355	3.091834	-4.694325
1	5.620975	1.896439	-1.227006
1	5.498856	1.906260	0.538101
1	4.614036	0.679975	-0.405490
1	4.796500	4.185180	-1.582101
1	3.363813	4.808323	-0.729140
1	4.854574	4.421384	0.175057
3	0.728433	2.495413	-1.102998
8	-0.594664	1.571875	-0.155368
3	-1.526481	1.954768	1.621878
7	-0.590318	3.729400	2.233157
1	0.170867	3.051904	2.167828
6	-0.283608	4.765700	1.223643
6	-0.647272	4.171708	3.644063
6	-1.117599	6.029861	1.384071
6	1.207081	5.097967	1.188954
1	-0.554797	4.309779	0.257941

6	-2.086925	4.454821	4.067152
6	-0.039094	3.080409	4.520020
1	-0.047699	5.088132	3.779033
1	-2.187477	5.807863	1.417137
1	-0.840820	6.575985	2.295734
1	-0.928367	6.694239	0.530774
1	1.540048	5.496475	2.158021
1	1.799262	4.201315	0.959925
1	1.420930	5.854133	0.421476
1	-2.537115	5.261986	3.480256
1	-2.694748	3.551486	3.924395
1	-2.118000	4.746185	5.125669
1	-0.533920	2.118477	4.321605
1	1.031523	2.958654	4.307799
1	-0.156378	3.330081	5.581613
3	-2.026049	2.301721	-1.183095
7	-3.857855	2.462121	-1.680069
6	-4.539835	3.614510	-1.131531
6	-4.772789	1.579780	-2.378693
6	-3.610017	4.354756	-0.175755
6	-5.037754	4.630492	-2.179317
1	-5.438006	3.324145	-0.535282
6	-4.010226	0.642880	-3.312375
6	-5.667415	0.729464	-1.451769
1	-5.471983	2.153762	-3.024237
1	-3.261041	3.711628	0.646239
1	-2.728133	4.715689	-0.728970
1	-4.098611	5.232796	0.269636
1	-4.174123	5.077692	-2.695279
1	-5.679653	4.159082	-2.933490
1	-5.615647	5.439355	-1.705978
1	-3.243173	0.095927	-2.746112
1	-4.680953	-0.094588	-3.776462
1	-3.516957	1.207788	-4.114655
1	-6.209900	1.355464	-0.729964
1	-6.413766	0.164932	-2.031611
1	-5.053781	0.011667	-0.887262
8	-0.568210	3.442987	-2.322948
6	-0.946592	2.927312	-3.626466
6	-1.440385	4.136951	-4.398969
1	-1.726312	2.174456	-3.472390
1	-0.066804	2.469973	-4.097128
6	-0.515471	5.243929	-3.884272
1	-2.485474	4.338560	-4.134875
1	-1.367702	3.988918	-5.481130
6	-0.329084	4.872043	-2.415048
1	0.444583	5.217674	-4.413506
1	-0.943171	6.245559	-3.993634
1	0.684089	5.073611	-2.041916
1	-1.059576	5.377753	-1.771749

8	-3.291152	1.659559	2.401115
6	-3.345919	0.766759	3.530639
6	-4.797225	0.297458	3.624983
1	-3.008973	1.311111	4.422482
1	-2.647674	-0.059946	3.341813
6	-5.269022	0.408294	2.172565
1	-5.379098	0.975521	4.262434
1	-4.874011	-0.715404	4.033855
6	-4.560961	1.674991	1.718981
1	-4.921302	-0.451021	1.583411
1	-6.357356	0.482359	2.074113
1	-4.362065	1.722497	0.641676
1	-5.117595	2.573732	2.030281

Ground State Structure 7

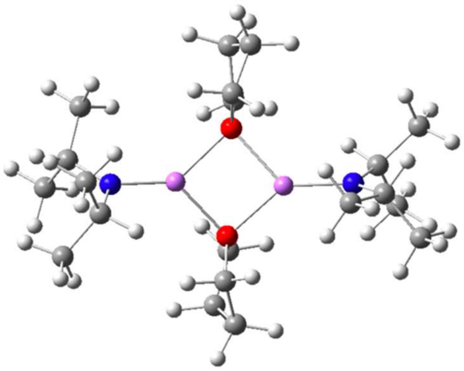

Figure S14. Representative ball and stick model ground state structure **7**.

M06-2x/mAUG-cc-pVDZ, Structure 7

#m062x/maug-cc-pvDz opt=(calcfc,noeigen) optcyc=50 freq scrf=(pcm,solvent=THF)

EE = -1063.265179 (Hartree)
ZPE_{298} = -1062.638716
E_{298} = -1062.604164
H_{298} = -1062.603220
G_{298} = -1062.708124
S = 220.789 (cal/mol·K)

3	1.488686	-0.158247	0.506071
8	0.182336	1.330578	0.259924
8	-0.009843	-1.462028	0.281511
7	3.318735	-0.079922	0.262420
3	-1.279453	0.070433	0.769115
7	-3.045557	0.084579	0.169730
6	0.323873	2.556689	1.022782

6	-0.237389	3.640047	0.119088
6	0.226236	3.158953	-1.257598
6	0.041633	1.649179	-1.155892
6	-0.371707	-1.842490	-1.080694
6	-0.568658	-3.354604	-1.047917
6	-0.978572	-3.598151	0.406029
6	-0.063574	-2.632481	1.137626
1	-0.222368	2.426626	1.965694
1	1.393850	2.713026	1.230749
1	-1.335956	3.650346	0.179180
1	0.142998	4.634823	0.380802
1	-0.354753	3.583579	-2.085438
1	1.287394	3.404013	-1.409423
1	-0.967481	1.333690	-1.462810
1	0.800506	1.080476	-1.711766
1	-1.307317	-1.316145	-1.321484
1	0.435734	-1.519025	-1.753122
1	-1.324954	-3.678804	-1.772937
1	0.375414	-3.874156	-1.268560
1	-2.031139	-3.315497	0.562184
1	-0.832551	-4.635196	0.731568
1	-0.431382	-2.312679	2.121423
1	0.956165	-3.035560	1.242158
6	4.123416	0.897513	0.975134
6	4.068884	-0.951346	-0.622104
6	-3.524975	0.311565	-1.184269
6	-4.052508	-0.259242	1.153404
1	3.411286	1.539362	1.532958
6	5.051877	0.281072	2.043104
6	4.944705	1.850665	0.085609
1	5.145688	-0.982943	-0.349117
6	3.547129	-2.389502	-0.513318
6	4.005343	-0.522520	-2.102153
1	-2.668049	0.109569	-1.862566
6	-4.632356	-0.644995	-1.656906
6	-3.925770	1.767666	-1.511660
1	-4.640536	-1.157169	0.846497
6	-3.352675	-0.646702	2.459423
6	-5.093112	0.832861	1.472842
1	5.803207	-0.380496	1.581128
1	5.596531	1.055119	2.610117
1	4.463819	-0.322074	2.751697
1	4.290407	2.356069	-0.642194
1	5.460313	2.619128	0.685436
1	5.714768	1.297885	-0.478445
1	2.462063	-2.400881	-0.727991
1	4.035893	-3.073397	-1.227165
1	3.692318	-2.778169	0.506225
1	2.957916	-0.566897	-2.450707
1	4.353983	0.512328	-2.227765

1	4.610962	-1.176728	-2.754113
1	-5.583163	-0.464188	-1.130018
1	-4.821836	-0.518307	-2.734360
1	-4.342339	-1.693161	-1.477670
1	-3.110484	2.453038	-1.229338
1	-4.123746	1.894584	-2.590634
1	-4.826509	2.080803	-0.963023
1	-2.766394	0.206327	2.849747
1	-4.071205	-0.932786	3.242352
1	-2.673793	-1.501177	2.302905
1	-4.580206	1.762869	1.774399
1	-5.724697	1.060630	0.602813
1	-5.761044	0.518524	2.293344

M06-2x/mAUG-cc-pVTZ, Structure 7

#m062x/maug-cc-pvTz opt=(calcfc,noeigen) optcyc=50 freq SCF=XQC scrf=(pcm,solvent=THF,read) nosymm

EE = -1063.576486 (Hartree)
ZPE_{298} = -1062.944770
E_{298} = -1062.911219
H_{298} = -1062.910274
G_{298} = -1063.009725
S = 209.311 (cal/mol·K)

3	-1.498290	0.210421	-0.437796
8	-0.157686	-1.262492	-0.609788
8	-0.073501	1.509177	0.127783
7	-3.246004	-0.021962	0.116614
3	1.113368	-0.106870	0.477857
7	2.955384	-0.058844	0.247049
6	-0.442078	-2.541640	-0.002374
6	0.538409	-3.485397	-0.666837
6	0.499167	-2.991387	-2.113672
6	0.345147	-1.479280	-1.956474
6	0.583014	2.308815	-0.894367
6	0.379289	3.750198	-0.463363
6	0.401127	3.621945	1.058780
6	-0.392692	2.346056	1.270357
1	-0.307454	-2.432820	1.071841
1	-1.480205	-2.807820	-0.213149
1	1.532082	-3.343015	-0.238546
1	0.249812	-4.528146	-0.563417
1	1.393977	-3.246458	-2.676134
1	-0.363646	-3.410273	-2.629411
1	1.297428	-0.955937	-2.029046
1	-0.364326	-1.052199	-2.664001
1	1.638620	2.031071	-0.897147
1	0.131795	2.066245	-1.854919

1	1.156003	4.403094	-0.852874
1	-0.591159	4.117051	-0.799037
1	1.425120	3.493819	1.411810
1	-0.045823	4.469869	1.571155
1	-0.124589	1.802412	2.174227
1	-1.467790	2.525927	1.264445
6	-3.565250	-0.888512	1.232607
6	-4.372076	0.705174	-0.427910
6	3.640108	0.023664	-1.028140
6	3.788504	-0.024018	1.426416
1	-2.644231	-1.436769	1.475150
6	-3.948423	-0.129643	2.519008
6	-4.635172	-1.958972	0.954748
1	-5.196872	0.782366	0.297888
6	-3.956676	2.139100	-0.770659
6	-4.967668	0.060467	-1.693751
1	2.909533	0.418613	-1.751794
6	4.818526	1.006905	-1.078189
6	4.087583	-1.326303	-1.630180
1	4.429834	0.876398	1.450907
6	2.886163	0.068408	2.656231
6	4.741644	-1.218505	1.620983
1	-4.867498	0.442774	2.376378
1	-4.112046	-0.810447	3.358672
1	-3.156268	0.570445	2.788975
1	-4.358962	-2.557000	0.084464
1	-4.762263	-2.626640	1.810506
1	-5.603180	-1.494670	0.751141
1	-3.095363	2.116577	-1.447868
1	-4.753039	2.697966	-1.268201
1	-3.660720	2.678342	0.130178
1	-4.208061	0.042219	-2.480698
1	-5.271302	-0.967529	-1.501256
1	-5.834250	0.613720	-2.068783
1	5.658733	0.656890	-0.475026
1	5.176052	1.127138	-2.102507
1	4.519457	1.986490	-0.699548
1	3.260987	-2.038779	-1.605444
1	4.409033	-1.207639	-2.669481
1	4.915198	-1.763594	-1.071971
1	2.244352	-0.817752	2.719655
1	3.460930	0.123174	3.581907
1	2.251045	0.956295	2.602651
1	4.177525	-2.154276	1.571384
1	5.511708	-1.248079	0.851648
1	5.245441	-1.165863	2.590341

M06-2x/JUL-cc-pVDZ, Structure 7

#m062x/jul-cc-pvDz opt=(calcfc,noeigen) optcyc=50 freq SCF=XQC scrf=(pcm,solvent=THF,read) nosymm

EE = -1063.303161 (Hartree)
ZPE_{298} = -1062.674728
E_{298} = -1062.640753
H_{298} = -1062.639808
G_{298} = -1062.739866
S = 210.589 (cal/mol·K)

3	-1.539898	0.239954	1.191505
8	-0.217854	-1.232307	0.804660
8	-0.067811	1.561282	0.879672
7	-3.098988	0.061165	0.192606
3	1.288918	0.067310	1.059506
7	2.913535	-0.030265	0.141156
6	-0.117149	-2.437354	1.600572
6	0.458905	-3.477745	0.656091
6	-0.227329	-3.108482	-0.662076
6	-0.243539	-1.584322	-0.614425
6	-0.014074	1.839110	-0.555843
6	-0.015928	3.356269	-0.674437
6	0.687284	3.778935	0.618053
6	0.104245	2.795726	1.620062
1	0.517594	-2.218216	2.466141
1	-1.122736	-2.715749	1.946606
1	1.546139	-3.345911	0.576884
1	0.244084	-4.499389	0.985221
1	0.307747	-3.478734	-1.543007
1	-1.251435	-3.502873	-0.684712
1	0.660388	-1.155747	-1.065104
1	-1.143306	-1.128836	-1.042581
1	0.923240	1.408724	-0.932826
1	-0.881550	1.346923	-1.011169
1	0.504462	3.690569	-1.577458
1	-1.043417	3.742178	-0.698223
1	1.771442	3.630511	0.529672
1	0.490873	4.818299	0.899577
1	0.754919	2.588375	2.476356
1	-0.879783	3.123278	1.982808
6	-3.604584	-1.297565	0.111972
6	-4.004778	1.076930	-0.297536
6	3.155425	-0.216084	-1.278881
6	4.087608	0.170756	0.963908
1	-2.766478	-1.956451	0.414941
6	-4.745009	-1.601390	1.106632
6	-4.030522	-1.779995	-1.288083
1	-5.065304	0.756118	-0.217900

6	-3.861419	2.344522	0.551480
6	-3.780453	1.468654	-1.774540
1	2.219368	0.071417	-1.799262
6	4.234934	0.696108	-1.883025
6	3.401513	-1.674333	-1.728516
1	4.688386	1.044619	0.622243
6	3.633187	0.506045	2.386620
6	5.074015	-1.011246	1.041051
1	-5.623562	-0.972923	0.900122
1	-5.066545	-2.652999	1.047815
1	-4.414011	-1.396392	2.133705
1	-3.222748	-1.626748	-2.017637
1	-4.287222	-2.850005	-1.272832
1	-4.915668	-1.229258	-1.639445
1	-2.802396	2.652793	0.554275
1	-4.456122	3.182298	0.157357
1	-4.164467	2.152521	1.589594
1	-2.797262	1.952942	-1.883296
1	-3.797174	0.590267	-2.429024
1	-4.547141	2.178198	-2.125775
1	5.240346	0.431081	-1.526375
1	4.238104	0.613961	-2.979224
1	4.043984	1.744944	-1.614276
1	2.615846	-2.327426	-1.323790
1	3.383219	-1.752162	-2.827600
1	4.369036	-2.056855	-1.379389
1	3.050026	-0.333146	2.804568
1	4.484350	0.681624	3.058385
1	3.009667	1.412680	2.393576
1	4.537467	-1.925456	1.340613
1	5.556604	-1.199099	0.074715
1	5.868343	-0.811929	1.777546

B3LYP/mAUG-cc-pVDZ, Structure 7

#B3LYP/maug-cc-pvDz opt=(calcfc,noeigen) optcyc=50 freq scrf=(pcm,solvent=THF,read)

EE = -1063.757276 (Hartree)
ZPE_{298} = -1063.137455
E_{298} = -1063.102265
H_{298} = -1063.101321
G_{298} = -1063.208261
S = 225.074 (cal/mol·K)

3	-1.508614	0.085424	-0.184906
8	-0.079729	-1.385128	-0.541974
8	0.016220	1.513363	-0.102702
7	-3.330386	0.009567	0.264922
3	1.397688	-0.050219	0.129857

7	3.265711	-0.005776	0.334463
6	-0.238709	-2.610939	0.254619
6	0.173051	-3.754289	-0.665906
6	-0.250766	-3.229642	-2.045716
6	0.109112	-1.750682	-1.953310
6	0.230340	2.294264	-1.331116
6	0.042393	3.755593	-0.932544
6	0.486697	3.758483	0.537760
6	-0.064440	2.430806	1.043222
1	0.388936	-2.510262	1.150893
1	-1.296659	-2.680650	0.553007
1	1.263159	-3.908093	-0.627738
1	-0.320846	-4.697212	-0.391574
1	0.270962	-3.725431	-2.876547
1	-1.335521	-3.354667	-2.191521
1	1.165617	-1.569258	-2.208821
1	-0.530849	-1.095660	-2.561433
1	1.253849	2.090302	-1.685251
1	-0.490237	1.944121	-2.084405
1	0.636017	4.429949	-1.565849
1	-1.017079	4.045053	-1.015989
1	1.585685	3.772827	0.612325
1	0.087974	4.610422	1.106621
1	0.516499	1.989892	1.865324
1	-1.122912	2.504820	1.339964
6	-3.735136	-0.792809	1.416006
6	-4.417088	0.685067	-0.432424
6	4.063344	0.513359	-0.775861
6	3.966420	-0.344074	1.559547
1	-2.809624	-1.285553	1.783912
6	-4.262305	0.033815	2.619991
6	-4.728631	-1.948722	1.127967
1	-5.323472	0.768464	0.208165
6	-4.016684	2.129445	-0.795185
6	-4.874971	-0.038389	-1.726660
1	3.349015	1.069729	-1.423658
6	5.151485	1.546466	-0.400936
6	4.680417	-0.552532	-1.726043
1	4.602300	0.502069	1.922312
6	2.930346	-0.590568	2.669539
6	4.916509	-1.570755	1.500353
1	-5.193452	0.570451	2.366478
1	-4.481270	-0.605757	3.495332
1	-3.515968	0.786939	2.922992
1	-4.344607	-2.601640	0.326286
1	-4.896660	-2.570643	2.026552
1	-5.713384	-1.566823	0.806544
1	-3.099712	2.126488	-1.415313
1	-4.801369	2.653450	-1.370950
1	-3.801243	2.715894	0.113246

1	-4.039394	-0.088034	-2.449746
1	-5.189057	-1.071773	-1.514396
1	-5.719161	0.480940	-2.220821
1	5.986684	1.087167	0.155485
1	5.581078	2.009725	-1.306444
1	4.734116	2.351784	0.227598
1	3.910069	-1.281008	-2.030259
1	5.089173	-0.084696	-2.642672
1	5.498754	-1.115444	-1.247598
1	2.277923	-1.445180	2.407006
1	3.407157	-0.826056	3.636336
1	2.288578	0.295436	2.815315
1	4.369497	-2.465532	1.150000
1	5.757982	-1.403282	0.811142
1	5.346718	-1.794703	2.495190

B3LYP/mAUG-cc-pVTZ, Structure 7

#B3LYP/maug-cc-pvTz opt=(calcfc,noeigen) optcyc=50 freq SCF=XQC scrf=(pcm,solvent=THF,read) nosymm

EE = -1064.090551 (Hartree)
ZPE_{298} = -1063.468032
E_{298} = -1063.432776
H_{298} = -1063.431831
G_{298} = -1063.538668
S = 224.856 (cal/mol·K)

3	-1.508997	0.082177	-0.166049
8	-0.078388	-1.392174	-0.540493
8	0.020055	1.520232	-0.098092
7	-3.324933	0.016362	0.275314
3	1.402301	-0.049455	0.135905
7	3.262408	-0.007315	0.334117
6	-0.236473	-2.615548	0.250417
6	0.158419	-3.756964	-0.673309
6	-0.254227	-3.223833	-2.049432
6	0.106202	-1.749885	-1.948868
6	0.229007	2.291338	-1.327317
6	0.055110	3.752879	-0.938299
6	0.487562	3.762251	0.531935
6	-0.060302	2.439114	1.039736
1	0.394357	-2.525340	1.132013
1	-1.280500	-2.681735	0.556919
1	1.234882	-3.925099	-0.634635
1	-0.344228	-4.684630	-0.407474
1	0.267096	-3.712369	-2.870091
1	-1.326927	-3.345190	-2.201721
1	1.150890	-1.571336	-2.206291
1	-0.528604	-1.099724	-2.547676

1	1.237262	2.082882	-1.686822
1	-0.492427	1.948789	-2.066803
1	0.655278	4.410546	-1.563677
1	-0.989294	4.051030	-1.030767
1	1.574581	3.780534	0.613248
1	0.086123	4.607492	1.087301
1	0.516205	2.008051	1.855377
1	-1.106974	2.514849	1.335915
6	-3.727547	-0.791935	1.416177
6	-4.406328	0.686503	-0.424050
6	4.053863	0.510483	-0.774145
6	3.960075	-0.342244	1.555291
1	-2.811452	-1.276702	1.784196
6	-4.266988	0.022746	2.618371
6	-4.711872	-1.948705	1.118307
1	-5.304844	0.769774	0.208160
6	-4.007689	2.125828	-0.791283
6	-4.860683	-0.038414	-1.714865
1	3.346477	1.057523	-1.417833
6	5.136196	1.545087	-0.401368
6	4.676743	-0.551818	-1.719180
1	4.594967	0.493697	1.907000
6	2.928626	-0.578393	2.665743
6	4.900553	-1.572903	1.501564
1	-5.193371	0.543119	2.364450
1	-4.478431	-0.618465	3.479760
1	-3.535613	0.773511	2.922105
1	-4.323686	-2.587532	0.322991
1	-4.876528	-2.568203	2.005129
1	-5.685686	-1.570906	0.798625
1	-3.099984	2.118692	-1.404064
1	-4.786037	2.639554	-1.363150
1	-3.796678	2.708157	0.106867
1	-4.032348	-0.082052	-2.428634
1	-5.166750	-1.061780	-1.501895
1	-5.698198	0.474023	-2.201716
1	5.962192	1.091078	0.150059
1	5.558172	2.002559	-1.299593
1	4.718286	2.340260	0.219668
1	3.918477	-1.279256	-2.014643
1	5.073716	-0.085718	-2.627849
1	5.492970	-1.097499	-1.244059
1	2.285385	-1.427831	2.413371
1	3.404387	-0.801676	3.623224
1	2.294803	0.300883	2.800958
1	4.350665	-2.454465	1.159306
1	5.733583	-1.413770	0.818342
1	5.322654	-1.791450	2.488285

B3LYP/JUL-cc-pVDZ, Structure 7

#B3LYP/jul-cc-pvDz opt=(calcfc,noeigen) optcyc=50 freq SCF=XQC scrf=(pcm,solvent=THF,read) nosymm

EE = -1063.796032 (Hartree)
ZPE_{298} = -1063.174338
E_{298} = -1063.139250
H_{298} = -1063.138306
G_{298} = -1063.243156
S = 220.675 (cal/mol·K)

3	-1.502399	0.074555	-0.182900
8	-0.075213	-1.392826	-0.540580
8	0.014663	1.505882	-0.101969
7	-3.320752	0.006984	0.263843
3	1.392651	-0.056988	0.129180
7	3.255417	-0.008525	0.329658
6	-0.235140	-2.621145	0.250767
6	0.146836	-3.766224	-0.681225
6	-0.261952	-3.223183	-2.058942
6	0.112593	-1.749036	-1.953385
6	0.228644	2.286172	-1.329964
6	0.062797	3.750412	-0.930483
6	0.485559	3.748309	0.546556
6	-0.072962	2.420907	1.043832
1	0.408834	-2.533721	1.133748
1	-1.285031	-2.679564	0.568038
1	1.229179	-3.950420	-0.642827
1	-0.372059	-4.695457	-0.416095
1	0.261259	-3.716798	-2.887002
1	-1.343542	-3.335484	-2.215413
1	1.167734	-1.575822	-2.207704
1	-0.519615	-1.084422	-2.554713
1	1.242387	2.069021	-1.694089
1	-0.502593	1.949153	-2.075111
1	0.678507	4.411892	-1.552022
1	-0.986302	4.060236	-1.030894
1	1.580155	3.763121	0.638907
1	0.077982	4.597798	1.108203
1	0.500424	1.977466	1.866278
1	-1.129984	2.497131	1.334135
6	-3.727188	-0.798955	1.409824
6	-4.405260	0.690111	-0.425425
6	4.054490	0.497707	-0.783075
6	3.952349	-0.317816	1.562395
1	-2.805460	-1.296748	1.773409
6	-4.248045	0.024334	2.617939
6	-4.725687	-1.948040	1.115657
1	-5.305527	0.775070	0.218890

6	-3.998923	2.133340	-0.783964
6	-4.873247	-0.026829	-1.718689
1	3.343564	1.044485	-1.438778
6	5.137238	1.537553	-0.414634
6	4.674195	-0.579240	-1.717699
1	4.558677	0.547489	1.923492
6	2.912926	-0.584253	2.663687
6	4.937609	-1.516175	1.521357
1	-5.174004	0.565617	2.368509
1	-4.467345	-0.621741	3.485214
1	-3.495422	0.766274	2.922597
1	-4.343934	-2.593682	0.310780
1	-4.888750	-2.572448	2.010351
1	-5.707054	-1.559611	0.801909
1	-3.084642	2.127215	-1.402586
1	-4.783224	2.657805	-1.355169
1	-3.784658	2.712114	0.126172
1	-4.045361	-0.069203	-2.446542
1	-5.184130	-1.058365	-1.507537
1	-5.720876	0.495984	-2.197177
1	5.971248	1.087379	0.144712
1	5.560999	1.993833	-1.323448
1	4.711655	2.342369	0.203966
1	3.903044	-1.305435	-2.015244
1	5.082683	-0.118147	-2.634760
1	5.488957	-1.134822	-1.231393
1	2.306599	-1.472158	2.414876
1	3.389166	-0.771016	3.638619
1	2.234790	0.275897	2.776326
1	4.422199	-2.423786	1.165398
1	5.783471	-1.323172	0.848633
1	5.352340	-1.722665	2.523581

HTCH407/mAUG-cc-pVDZ, Structure 7

#HCTH407/maug-cc-pvDz opt=(calcfc,noeigen) optcyc=50 freq SCF=XQC scrf=(pcm,solvent=THF,read) nosymm

EE = -1063.775683 (Hartree)
ZPE_{298} = 1063.160328
E_{298} = 1063.124733
H_{298} = 1063.123788
G_{298} = 1063.229891
S = 223.311 (cal/mol·K)

3	-1.566248	0.074330	-0.144119
8	-0.110003	-1.478243	-0.558779
8	0.090570	1.549895	-0.216237
7	-3.432943	0.088026	0.246062
3	1.544337	-0.098239	-0.023071

7	3.425056	-0.109742	0.317546
6	-0.188902	-2.664494	0.287656
6	0.162797	-3.835989	-0.609657
6	-0.395007	-3.383873	-1.957495
6	-0.084159	-1.896805	-1.958183
6	0.182449	2.235958	-1.502788
6	-0.019577	3.711920	-1.207376
6	0.541436	3.841633	0.207334
6	0.087724	2.544587	0.851520
1	0.502856	-2.522377	1.131426
1	-1.218422	-2.742969	0.677704
1	1.254849	-3.970374	-0.663356
1	-0.281272	-4.775147	-0.250958
1	0.065346	-3.899074	-2.812046
1	-1.482871	-3.550834	-2.001678
1	0.923428	-1.684115	-2.357593
1	-0.822041	-1.293020	-2.509581
1	1.181651	2.031556	-1.926823
1	-0.580049	1.808593	-2.173179
1	0.494847	4.350645	-1.938938
1	-1.091323	3.965686	-1.224165
1	1.641494	3.900727	0.187287
1	0.160928	4.722183	0.743634
1	0.755809	2.183932	1.648105
1	-0.939741	2.619446	1.248870
6	-3.741081	-0.555557	1.506942
6	-4.555119	0.607836	-0.502272
6	4.213171	0.829006	-0.457391
6	4.151131	-0.898071	1.290577
1	-2.769729	-0.942117	1.889486
6	-4.253302	0.393314	2.621535
6	-4.657117	-1.803809	1.446641
1	-5.472707	0.678111	0.123523
6	-4.287227	2.041808	-0.997716
6	-4.953225	-0.267933	-1.716161
1	3.502317	1.310346	-1.165707
6	4.819565	2.006654	0.348850
6	5.312105	0.219825	-1.364028
1	5.175357	-0.499595	1.461569
6	3.464224	-0.862658	2.669710
6	4.354355	-2.374868	0.872611
1	-5.234287	0.831531	2.370541
1	-4.370436	-0.127535	3.589318
1	-3.548650	1.228078	2.769155
1	-4.261759	-2.539694	0.727688
1	-4.734813	-2.300025	2.430810
1	-5.683179	-1.549888	1.131696
1	-3.382776	2.072666	-1.632779
1	-5.121763	2.444663	-1.599198
1	-4.116360	2.724730	-0.150133

1	-4.113090	-0.340888	-2.431105
1	-5.203155	-1.292287	-1.402225
1	-5.824256	0.139043	-2.264568
1	5.574536	1.665217	1.077060
1	5.316080	2.744212	-0.307651
1	4.032119	2.532180	0.913462
1	4.888402	-0.565195	-2.011144
1	5.774639	0.983250	-2.015535
1	6.125585	-0.239008	-0.776987
1	2.425594	-1.236081	2.598554
1	3.983272	-1.484903	3.421099
1	3.415924	0.168579	3.054582
1	3.380710	-2.879700	0.739048
1	4.892026	-2.444634	-0.084899
1	4.927439	-2.950730	1.623923

HTCH407/mAUG-cc-pVTZ, Structure 7

#HCTH407/maug-cc-pvTz opt=(calcfc,noeigen) optcyc=50 freq SCF=XQC scrf=(pcm,solvent=THF,read) nosymm

EE = -1064.034832 (Hartree)
ZPE_{298} = -1063.419382
E_{298} = -1063.383617
H_{298} = -1063.382673
G_{298} = -1063.489124
S = 224.045 (cal/mol·K)

3	-1.574661	0.075740	-0.124462
8	-0.106360	-1.487084	-0.554095
8	0.094592	1.562907	-0.225180
7	-3.431016	0.087082	0.256432
3	1.557856	-0.101821	-0.020453
7	3.424783	-0.109341	0.329138
6	-0.187331	-2.672260	0.285874
6	0.141506	-3.844371	-0.615416
6	-0.406991	-3.379304	-1.960681
6	-0.086065	-1.897072	-1.951167
6	0.177123	2.234142	-1.514596
6	-0.019823	3.711447	-1.235839
6	0.541699	3.856447	0.175255
6	0.102258	2.563205	0.831242
1	0.510539	-2.544236	1.115037
1	-1.203891	-2.743839	0.685238
1	1.221822	-3.998301	-0.669934
1	-0.317344	-4.768953	-0.263946
1	0.051082	-3.888774	-2.808817
1	-1.486965	-3.537080	-2.010673
1	0.913078	-1.693063	-2.351743
1	-0.815668	-1.292068	-2.494330

1	1.163976	2.029150	-1.943603
1	-0.585176	1.807385	-2.170725
1	0.494103	4.335179	-1.967775
1	-1.081747	3.967498	-1.255531
1	1.632100	3.925445	0.152673
1	0.156134	4.731132	0.699759
1	0.777937	2.217063	1.615554
1	-0.910636	2.639301	1.240445
6	-3.741677	-0.556965	1.511687
6	-4.547045	0.607243	-0.493278
6	4.207754	0.833514	-0.438623
6	4.151669	-0.903628	1.290955
1	-2.780548	-0.939163	1.898645
6	-4.263540	0.386805	2.624106
6	-4.654945	-1.805441	1.447467
1	-5.459908	0.672781	0.124083
6	-4.281394	2.041097	-0.983788
6	-4.939159	-0.261427	-1.712282
1	3.502800	1.322413	-1.133614
6	4.824606	1.999422	0.373799
6	5.299806	0.232441	-1.356704
1	5.169639	-0.511644	1.456026
6	3.476487	-0.877959	2.673954
6	4.348970	-2.377475	0.865901
1	-5.237584	0.815481	2.369980
1	-4.379564	-0.135377	3.580995
1	-3.566874	1.216112	2.773517
1	-4.257760	-2.532990	0.734593
1	-4.730699	-2.296272	2.424503
1	-5.671588	-1.552244	1.133168
1	-3.385301	2.071736	-1.614360
1	-5.112135	2.439335	-1.577329
1	-4.112475	2.713867	-0.139381
1	-4.102296	-0.324741	-2.417539
1	-5.184987	-1.279239	-1.405491
1	-5.802598	0.146107	-2.253966
1	5.581397	1.649966	1.082234
1	5.309097	2.735575	-0.278143
1	4.048735	2.514435	0.946662
1	4.871456	-0.536251	-2.005367
1	5.754891	0.998801	-1.994784
1	6.106932	-0.230888	-0.781738
1	2.448844	-1.255119	2.608870
1	4.004226	-1.495439	3.409980
1	3.427555	0.143680	3.058481
1	3.380378	-2.873688	0.741334
1	4.874440	-2.441494	-0.088468
1	4.923017	-2.949229	1.606238

HTCH407/JUL-cc-pVDZ, Structure 7

#HCTH407/jul-cc-pvDz opt=(calcfc,noeigen) optcyc=50 freq SCF=XQC scrf=(pcm,solvent=THF,read) nosymm

EE = -1063.807968 (Hartree)
ZPE_{298} = -1063.191667
E_{298} = -1063.155830
H_{298} = -1063.154886
G_{298} = -1063.262335
S = 226.146 (cal/mol·K)

3	-1.567559	0.070792	-0.146301
8	-0.107843	-1.479342	-0.562728
8	0.092318	1.548286	-0.217723
7	-3.429914	0.085411	0.245132
3	1.544771	-0.097284	-0.024842
7	3.420498	-0.109130	0.321060
6	-0.189154	-2.666319	0.282951
6	0.156712	-3.840447	-0.613895
6	-0.385763	-3.384654	-1.967718
6	-0.080665	-1.895972	-1.963364
6	0.184915	2.230049	-1.507386
6	-0.012174	3.708620	-1.219675
6	0.532720	3.845413	0.201576
6	0.085934	2.546268	0.847336
1	0.504887	-2.527082	1.122155
1	-1.214557	-2.741924	0.675998
1	1.244489	-3.988475	-0.657923
1	-0.301157	-4.772346	-0.260653
1	0.088566	-3.895136	-2.814544
1	-1.469123	-3.556297	-2.028808
1	0.922579	-1.678467	-2.363119
1	-0.820498	-1.295828	-2.511270
1	1.179533	2.021267	-1.932378
1	-0.579352	1.803811	-2.172394
1	0.515847	4.338904	-1.945466
1	-1.078842	3.969396	-1.253037
1	1.629222	3.917646	0.194359
1	0.136535	4.721567	0.729007
1	0.756899	2.191548	1.640859
1	-0.937885	2.616811	1.246844
6	-3.736905	-0.562196	1.503762
6	-4.552191	0.616110	-0.493968
6	4.211431	0.826043	-0.454481
6	4.144002	-0.895009	1.296918
1	-2.768267	-0.954656	1.880496
6	-4.239661	0.384830	2.624371
6	-4.660094	-1.805203	1.441356
1	-5.462030	0.693823	0.137739

6	-4.273990	2.048181	-0.989115
6	-4.967732	-0.254151	-1.705976
1	3.504578	1.305737	-1.164326
6	4.817381	2.005164	0.350051
6	5.311577	0.213138	-1.357424
1	5.162535	-0.491985	1.474602
6	3.447631	-0.864232	2.671377
6	4.358414	-2.370392	0.879000
1	-5.215851	0.829425	2.378774
1	-4.355561	-0.143269	3.585546
1	-3.527116	1.208973	2.773493
1	-4.270792	-2.536256	0.718126
1	-4.730410	-2.302967	2.422586
1	-5.684072	-1.543817	1.135834
1	-3.374407	2.069734	-1.626414
1	-5.108350	2.455459	-1.583403
1	-4.095096	2.724442	-0.141384
1	-4.135590	-0.329754	-2.425509
1	-5.222540	-1.273955	-1.390702
1	-5.839022	0.162467	-2.241447
1	5.568902	1.665795	1.078447
1	5.313185	2.736178	-0.310182
1	4.028557	2.531632	0.906658
1	4.886663	-0.571402	-1.999934
1	5.769259	0.975679	-2.009241
1	6.123070	-0.240244	-0.768854
1	2.412096	-1.235446	2.590252
1	3.962844	-1.488714	3.419840
1	3.400537	0.163871	3.056890
1	3.390383	-2.879660	0.745170
1	4.898057	-2.433858	-0.074650
1	4.934179	-2.936904	1.631670

MP2/mAUG-cc-pVDZ, Structure 7

#MP2/maug-cc-pvDz opt=(calcfc,noeigen) optcyc=50 freq SCF=XQC scrf=(pcm,solvent=THF,read) nosymm

EE = -1060.296022 (Hartree)
ZPE_{298} = -1059.665692
E_{298} = -1059.631237
H_{298} = -1059.630292
G_{298} = -1059.733898
S = 218.057 (cal/mol·K)

3	-1.548121	-0.025529	-0.065709
8	-0.096808	-1.467126	-0.405343
8	-0.079576	1.396927	-0.137986
7	-3.388506	-0.017881	0.340775
3	1.301073	-0.096399	0.236633

7	3.174035	0.079711	0.321130
6	-0.194694	-2.689761	0.399073
6	0.472539	-3.764997	-0.446366
6	0.005593	-3.368699	-1.854157
6	0.115513	-1.844936	-1.812795
6	0.143380	1.988426	-1.466016
6	0.231269	3.498306	-1.241389
6	0.757325	3.583204	0.199153
6	-0.026749	2.463122	0.868356
1	0.300281	-2.492139	1.361575
1	-1.263643	-2.908424	0.568214
1	1.570250	-3.690198	-0.362586
1	0.156595	-4.777348	-0.151139
1	0.622103	-3.797840	-2.658594
1	-1.042910	-3.674711	-2.008336
1	1.122659	-1.498101	-2.100430
1	-0.642375	-1.330061	-2.423840
1	1.089415	1.576237	-1.857746
1	-0.690915	1.684740	-2.118895
1	0.891318	3.979595	-1.979184
1	-0.769593	3.956950	-1.309297
1	1.838465	3.364711	0.236441
1	0.569339	4.559226	0.672507
1	0.452565	2.043880	1.766248
1	-1.060787	2.766811	1.104697
6	-3.926507	-1.034120	1.244707
6	-4.397209	0.894402	-0.186823
6	3.950440	0.522962	-0.836938
6	3.915103	-0.138779	1.552141
1	-3.078243	-1.708708	1.496331
6	-4.424917	-0.466348	2.596682
6	-5.028853	-1.941787	0.649575
1	-5.327218	0.877639	0.427154
6	-3.877777	2.342004	-0.154238
6	-4.823526	0.563218	-1.636538
1	3.225741	1.024301	-1.519387
6	5.029432	1.583673	-0.535769
6	4.571979	-0.604288	-1.703574
1	4.482054	0.773770	1.871296
6	2.903576	-0.423995	2.672221
6	4.948967	-1.290595	1.542025
1	-5.266712	0.234022	2.446277
1	-4.775815	-1.268827	3.274276
1	-3.610998	0.085249	3.098879
1	-4.676770	-2.422249	-0.280305
1	-5.323749	-2.734243	1.363857
1	-5.935566	-1.357300	0.408787
1	-2.922386	2.403515	-0.710943
1	-4.585289	3.053553	-0.621834
1	-3.685655	2.664616	0.883843

1	-3.946674	0.654190	-2.306623
1	-5.197888	-0.470576	-1.710792
1	-5.611860	1.249416	-2.006697
1	5.862636	1.162124	0.054623
1	5.457858	1.981495	-1.474099
1	4.600720	2.427094	0.035193
1	3.797462	-1.346684	-1.966237
1	4.991993	-0.194908	-2.644355
1	5.381534	-1.133882	-1.173969
1	2.339813	-1.349419	2.441658
1	3.397095	-0.565442	3.650481
1	2.181389	0.407392	2.768889
1	4.460907	-2.229075	1.216073
1	5.786944	-1.085252	0.856828
1	5.376479	-1.446161	2.552333

MP2/mAUG-cc-pVTZ, Structure 7

#MP2/maug-cc-pvTz MAXDISK=3000GB opt=(mndofc,noeigen) optcyc=50 freq=numer SCF=XQC scrf=(pcm,solvent=THF,read) nosymm

EE = -1061.367895 (Hartree)

3	-1.413404	0.364945	0.773264
8	-0.256095	-1.376010	0.770306
8	0.176676	1.414344	-0.034222
7	-3.208663	0.160897	0.259332
3	1.410694	-0.135340	0.632706
7	3.268839	0.034141	0.446119
6	-0.314567	-2.285750	1.904944
6	-0.100881	-3.662323	1.311653
6	-0.857930	-3.547959	-0.010749
6	-0.537417	-2.129477	-0.456112
6	0.146803	1.510492	-1.488207
6	-0.161917	2.964445	-1.772993
6	0.632867	3.666205	-0.672698
6	0.435144	2.742430	0.520542
1	0.452948	-1.978440	2.610855
1	-1.297588	-2.199493	2.369979
1	0.959659	-3.835036	1.133513
1	-0.484011	-4.449365	1.956269
1	-0.540598	-4.280210	-0.748607
1	-1.928151	-3.660009	0.155506
1	0.357583	-2.089353	-1.076579
1	-1.366576	-1.634320	-0.957692
1	1.127453	1.228181	-1.873220
1	-0.607203	0.811997	-1.842955
1	0.144879	3.258340	-2.773691
1	-1.228532	3.153090	-1.659305

1	1.686065	3.715872	-0.944305
1	0.278041	4.672033	-0.463454
1	1.309302	2.674680	1.164376
1	-0.435413	3.023932	1.111227
6	-3.819857	-1.131279	0.522247
6	-4.114934	1.126247	-0.328807
6	3.774031	1.151495	-0.338866
6	4.266043	-0.817698	1.052833
1	-3.016835	-1.785468	0.885484
6	-4.874406	-1.111315	1.645067
6	-4.427785	-1.842575	-0.699344
1	-5.167180	0.873591	-0.122620
6	-3.868749	2.511389	0.272051
6	-3.985163	1.239769	-1.859036
1	2.959967	1.888174	-0.376646
6	4.969171	1.892877	0.273549
6	4.086369	0.845405	-1.819343
1	4.962301	-0.245357	1.693230
6	3.553667	-1.809882	1.967565
6	5.152865	-1.623419	0.085872
1	-5.716652	-0.471670	1.376303
1	-5.269906	-2.110816	1.844798
1	-4.434559	-0.721649	2.563015
1	-3.695324	-1.925830	-1.503134
1	-4.773002	-2.846340	-0.438700
1	-5.285927	-1.287002	-1.081728
1	-2.814742	2.772886	0.146523
1	-4.466497	3.287682	-0.211732
1	-4.088234	2.511321	1.339516
1	-2.977284	1.577510	-2.110756
1	-4.142532	0.275635	-2.337648
1	-4.699309	1.955778	-2.277760
1	5.868885	1.276112	0.262335
1	5.186535	2.801798	-0.290719
1	4.763509	2.170152	1.308320
1	3.251799	0.310894	-2.274191
1	4.251165	1.770149	-2.381017
1	4.975679	0.226440	-1.927398
1	2.879523	-2.434046	1.374806
1	4.257274	-2.468133	2.479642
1	2.964074	-1.284796	2.720734
1	4.522941	-2.195391	-0.599523
1	5.798079	-0.977356	-0.505566
1	5.794545	-2.318522	0.634841

MP2/JUL-cc-pVDZ, Structure 7

#MP2/jul-cc-pvDz opt=(calcfc,noeigen) optcyc=50 freq SCF=XQC scrf=(pcm,solvent=THF,read) nosymm

EE =	-1060.431137 (Hartree)
ZPE_{298} =	-1059.803841
E_{298} =	-1059.768939
H_{298} =	-1059.767995
G_{298} =	-1059.872916
S =	220.824 (cal/mol·K)

3	-1.546293	0.103113	1.045014
8	-0.237097	-1.471363	0.686844
8	-0.020485	1.442710	0.887861
7	-3.201591	-0.020842	0.140543
3	1.313092	-0.146271	0.999388
7	3.035106	0.005733	0.207315
6	-0.206051	-2.664960	1.542778
6	0.197403	-3.801837	0.611514
6	-0.551344	-3.421961	-0.677797
6	-0.380191	-1.903163	-0.725669
6	0.184220	2.023212	-0.461682
6	0.268567	3.532332	-0.246047
6	0.891106	3.619487	1.157680
6	0.146375	2.507642	1.888277
1	0.505896	-2.468936	2.358102
1	-1.214051	-2.820903	1.962487
1	1.286783	-3.790536	0.447819
1	-0.095200	-4.784537	1.009610
1	-0.134939	-3.902432	-1.575347
1	-1.616615	-3.689574	-0.596446
1	0.544567	-1.611866	-1.246701
1	-1.244211	-1.359664	-1.134600
1	1.132970	1.613145	-0.841166
1	-0.660535	1.698930	-1.085311
1	0.880540	4.015838	-1.021549
1	-0.735061	3.986937	-0.252138
1	1.968513	3.392728	1.113610
1	0.745916	4.597494	1.639416
1	0.688937	2.079090	2.743553
1	-0.854836	2.834855	2.215159
6	-3.794380	-1.358188	0.099149
6	-4.045239	1.027256	-0.418237
6	3.129979	-0.192874	-1.240816
6	4.234386	0.497631	0.864695
1	-3.005555	-2.054142	0.459692
6	-4.990925	-1.545730	1.066522
6	-4.213301	-1.874713	-1.299332
1	-5.127411	0.761043	-0.368160

6	-3.865921	2.322397	0.395063
6	-3.740183	1.352185	-1.903318
1	2.090318	-0.120597	-1.636476
6	3.922537	0.891798	-2.002703
6	3.607413	-1.597904	-1.694222
1	4.589532	1.463249	0.421451
6	3.889963	0.802170	2.331188
6	5.462795	-0.445950	0.848554
1	-5.821737	-0.869582	0.800675
1	-5.380496	-2.580865	1.039072
1	-4.681516	-1.317105	2.099694
1	-3.375424	-1.806250	-2.013222
1	-4.539959	-2.929884	-1.247515
1	-5.055411	-1.285072	-1.701066
1	-2.790899	2.580571	0.430781
1	-4.405945	3.176869	-0.052653
1	-4.219077	2.185355	1.430441
1	-2.707845	1.735917	-1.990356
1	-3.820820	0.456202	-2.535934
1	-4.428351	2.122285	-2.303178
1	5.001106	0.847301	-1.776571
1	3.804825	0.761596	-3.093060
1	3.560043	1.899395	-1.733786
1	3.006853	-2.374412	-1.190775
1	3.488220	-1.722354	-2.788242
1	4.665865	-1.773290	-1.444782
1	3.553619	-0.123018	2.835568
1	4.761339	1.194647	2.883965
1	3.079841	1.548999	2.395976
1	5.187057	-1.425570	1.279489
1	5.836677	-0.613653	-0.172861
1	6.294081	-0.020419	1.442783

LDA – THF – *tert*-Butyl Pyrrolidine *N*-Oxide Dimers 8-10

Ground State Structure 8

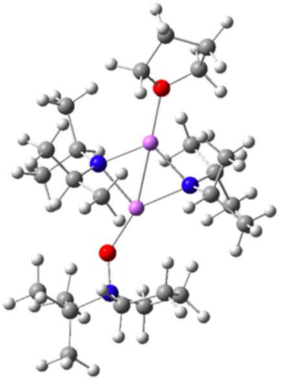

Figure S15. Representative ball and stick model of ground state structure **8**.

#m062x/jul-cc-pvDz opt=(calcfc,noeigen) optcyc=50 freq SCF=XQC scrf=(pcm,solvent=THF,read) nosymm

EE = -1275.817049 (Hartree)
ZPE_{298} = -1275.057530
E_{298} = -1275.017838
H_{298} = -1275.016894
G_{298} = -1275.127695
S = 233.200 (cal/mol·K)

3	0.000000	0.000000	0.000000
7	0.000000	0.000000	1.926000
3	1.955865	0.000000	1.354324
7	1.893509	0.080035	-0.656618
8	-1.588632	-0.204937	-1.090914
6	-2.669727	0.711572	-0.826827
6	-3.529620	0.710716	-2.087607
6	-2.499352	0.398196	-3.176532
6	-1.609770	-0.614896	-2.470509
1	-2.241530	1.706168	-0.631109
1	-3.197315	0.369420	0.071202
1	-4.044171	1.665677	-2.235504
1	-4.281265	-0.087477	-2.036175
1	-1.921440	1.296824	-3.430559
1	-2.944381	-0.005670	-4.091762
1	-0.575355	-0.631868	-2.832933
1	-2.031448	-1.629277	-2.529161
6	-0.382137	-1.259282	2.552487
6	-0.144608	1.192419	2.732868
1	0.187739	-2.048782	2.022463
6	0.019692	-1.395194	4.028751
6	-1.859726	-1.662174	2.358249

1	0.439486	1.120742	3.678514
6	0.455890	2.367472	1.955797
6	-1.581803	1.565196	3.143911
1	-0.573447	-0.731714	4.675095
1	-0.136097	-2.426234	4.377759
1	1.080421	-1.134624	4.146565
1	-2.113310	-1.614050	1.289886
1	-2.037601	-2.690807	2.711972
1	-2.542113	-0.994842	2.901239
1	-0.058876	2.484820	0.987824
1	0.359191	3.311759	2.510459
1	1.524846	2.203882	1.756021
1	-2.220143	1.642328	2.249678
1	-2.019545	0.813534	3.811353
1	-1.599919	2.532011	3.671609
8	3.019997	0.115528	2.870819
7	4.236982	0.566063	3.302123
6	5.309134	-0.113572	2.494592
6	4.386115	2.007306	2.900389
6	4.352580	0.327361	4.824290
1	5.074547	-1.179017	2.477440
6	5.231447	0.567947	1.126856
1	6.276829	0.053134	2.977345
6	4.573593	1.942916	1.387923
1	3.491064	2.539788	3.228070
1	5.276381	2.413935	3.389626
6	3.123669	0.946807	5.485489
6	4.361859	-1.183696	5.046932
6	5.618511	0.955540	5.410556
6	2.110044	1.341261	-1.358860
6	2.128440	-1.114649	-1.454490
1	2.036603	2.132027	-0.590105
6	3.477319	1.531024	-2.040030
6	1.012367	1.679627	-2.390032
1	1.865612	-0.950479	-2.522960
6	3.576161	-1.649162	-1.432536
6	1.233420	-2.250494	-0.939688
1	4.302112	1.332518	-1.344056
1	3.583151	0.853266	-2.900067
1	3.578722	2.560329	-2.414527
1	0.021517	1.671272	-1.913770
1	1.171045	2.676396	-2.829387
1	1.004961	0.952352	-3.215669
1	4.304071	-0.893748	-1.745721
1	3.829764	-1.962147	-0.407124
1	3.684923	-2.524796	-2.092049
1	1.447694	-2.425951	0.126428
1	0.163595	-2.016703	-1.036005
1	1.416986	-3.191760	-1.476868
1	5.705290	0.605749	6.446561

1	6.533459	0.651811	4.890018
1	5.564962	2.049265	5.438815
1	3.200052	0.777276	6.566514
1	3.076971	2.030134	5.321254
1	2.206620	0.487498	5.108305
1	4.213905	-1.377820	6.115716
1	3.548022	-1.654922	4.486590
1	5.319633	-1.631414	4.754687
1	6.232680	0.661605	0.693260
1	4.609464	-0.012922	0.441334
1	3.610241	2.000103	0.871789
1	5.192222	2.778369	1.044235

Ground State Structure 9

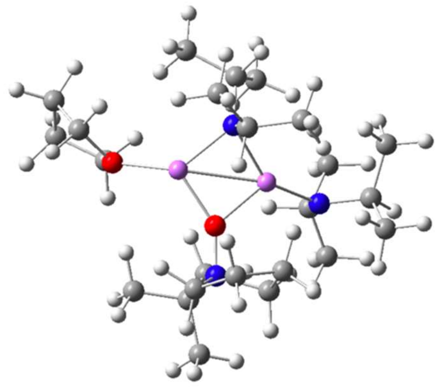

Figure S16. Representative ball and stick model of ground state structure **9**.

#m062x/jul-cc-pvDz opt=(calcfc,noeigen) optcyc=50 freq SCF=XQC scrf=(pcm,solvent=THF,read) nosymm

EE = -1275.809630 (Hartree)
ZPE_{298} = -1275.050705
E_{298} = -1275.010931
H_{298} = -1275.009987
G_{298} = -1275.121049
S = 233.750 (cal/mol·K)

3	-0.095640	-0.094379	-0.000611
7	-0.173758	-0.542195	1.887270
3	1.837996	-0.217082	1.726628
8	1.665204	-0.564075	-0.223133
8	-1.012096	1.237718	-1.061476
6	-0.653361	2.623025	-0.891192
6	-1.837788	3.412410	-1.431360
6	-3.014181	2.508304	-1.053826
6	-2.434853	1.115224	-1.279331
1	0.280687	2.800139	-1.436941
1	-0.490151	2.819671	0.179467

1	-1.762266	3.510870	-2.521918
1	-1.902608	4.412672	-0.991495
1	-3.906705	2.684011	-1.662857
1	-3.278501	2.646389	0.002064
1	-2.591901	0.770563	-2.310172
1	-2.827289	0.364797	-0.581993
6	-0.422166	-1.981917	1.857088
6	-0.918861	0.174305	2.909613
1	0.103788	-2.358586	0.957970
6	0.191232	-2.745422	3.045764
6	-1.896179	-2.399322	1.699345
1	-1.215106	-0.497970	3.740453
6	-0.039908	1.269027	3.520185
6	-2.210191	0.825649	2.379073
1	1.277600	-2.587060	3.077938
1	-0.228218	-2.392367	3.999311
1	0.000667	-3.827433	2.974858
1	-2.484588	-2.109132	2.582193
1	-2.346068	-1.917592	0.819001
1	-1.986160	-3.489397	1.581831
1	0.306428	1.946338	2.720315
1	-0.584106	1.873165	4.262062
1	0.851814	0.833726	3.988528
1	-2.882445	0.081707	1.934031
1	-2.758988	1.358409	3.172177
1	-1.943180	1.555078	1.597712
7	3.505340	0.226397	2.626422
6	3.935539	1.612160	2.624683
6	4.081231	-0.547255	3.708290
1	3.343670	2.126875	1.844406
6	3.673665	2.407213	3.922308
6	5.418782	1.804681	2.232883
1	5.007540	-0.075486	4.098833
6	3.141405	-0.710265	4.922393
6	4.493053	-1.941295	3.219115
1	2.613062	2.347432	4.199733
1	4.269985	2.007324	4.755882
1	3.945002	3.467341	3.797943
1	5.629203	1.305478	1.276806
1	5.683633	2.870062	2.138744
1	6.082033	1.365242	2.992573
1	2.868839	0.267548	5.337947
1	2.212193	-1.207577	4.605233
1	3.602912	-1.311750	5.723717
1	3.618086	-2.459543	2.797903
1	5.251788	-1.860049	2.427683
1	4.896526	-2.564394	4.032459
7	2.651191	-0.893198	-1.116643
6	3.726144	-1.610039	-0.339603
6	2.116308	-1.966658	-2.024144

6	3.126221	0.368213	-1.866552
1	3.998001	-0.983459	0.520369
6	3.049982	-2.926089	0.027653
1	4.574168	-1.782834	-1.008912
1	1.125091	-1.649930	-2.352331
1	2.783355	-2.051046	-2.887713
6	2.125269	-3.240357	-1.169906
6	3.328246	1.464444	-0.827305
6	4.440942	0.107432	-2.602296
6	2.033884	0.770092	-2.855093
1	3.864591	2.294941	-1.303159
1	2.368371	1.830919	-0.450310
1	3.913604	1.103398	0.024469
1	4.618641	0.957545	-3.271699
1	5.288304	0.045220	-1.910643
1	4.414494	-0.797053	-3.221455
1	2.247720	1.788288	-3.202325
1	2.013891	0.113971	-3.733439
1	1.048529	0.767694	-2.374218
1	3.797143	-3.706762	0.202533
1	2.459147	-2.806567	0.940954
1	1.116370	-3.492018	-0.829611
1	2.497942	-4.081685	-1.762865

Ground State Structure 10

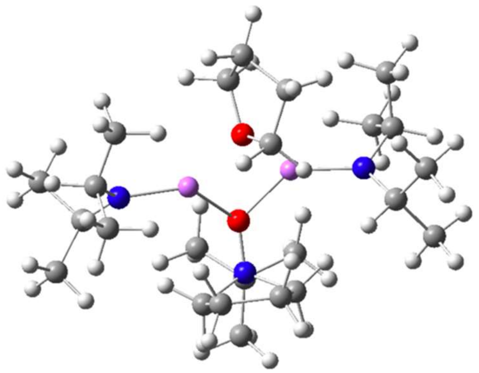

Figure S17. Representative ball and stick model of ground state structure **10**.

#m062x/jul-cc-pvDz opt=(calcfc,noeigen) optcyc=50 freq SCF=XQC scrf=(pcm,solvent=THF,read) nosymm

EE = -1275.803617 (Hartree)
ZPE298 = 1275.044365
E298 = 1275.004487
H298 = 1275.003543
G298 = 1275.115334
S = 235.284 (cal/mol·K)

3	-0.379735	-0.574348	0.226706
8	-0.337577	-0.049566	2.204385
3	1.629014	-0.459052	2.108426
8	1.488509	-0.708180	0.249347
7	-1.689228	-0.015538	-0.989128
6	-1.752360	1.305546	-1.581494
6	-2.858243	-0.836117	-1.239806
1	-0.854999	1.851864	-1.231252
6	-1.663247	1.301476	-3.122731
6	-2.955426	2.170724	-1.155682
1	-3.427648	-0.476805	-2.121220
6	-2.440217	-2.277423	-1.556581
6	-3.854729	-0.860609	-0.062257
1	-2.537594	0.801397	-3.564118
1	-1.622531	2.322944	-3.531822
1	-0.765729	0.757268	-3.448391
1	-3.899119	1.725509	-1.503850
1	-3.002421	2.253050	-0.060340
1	-2.887395	3.184120	-1.579616
1	-1.838376	-2.308158	-2.474113
1	-1.819962	-2.677715	-0.736275
1	-3.306590	-2.945586	-1.676978
1	-3.359204	-1.286238	0.825529
1	-4.183748	0.154411	0.191945
1	-4.745225	-1.471109	-0.284426
6	-1.101068	-0.777484	3.202148
6	-1.926416	0.269297	3.941177
6	-2.112573	1.352834	2.875623
6	-0.770193	1.335135	2.161428
1	-1.737929	-1.501425	2.677660
1	-0.391134	-1.310891	3.844734
1	-2.874062	-0.143619	4.301683
1	-1.367780	0.663309	4.799022
1	-2.910113	1.071197	2.176337
1	-2.339587	2.337444	3.296937
1	-0.841859	1.628590	1.106434
1	-0.018316	1.949474	2.676960
7	2.978827	-0.423109	3.389239
6	4.116357	-1.310203	3.236131
6	3.047620	0.434268	4.554652
1	3.973582	-1.837024	2.272269
6	5.489331	-0.613796	3.148969
6	4.190797	-2.427286	4.298373
1	3.754545	0.038978	5.312471
6	3.511323	1.869823	4.233476
6	1.675918	0.514641	5.233827
1	5.486872	0.153804	2.361945
1	5.734584	-0.118769	4.100398
1	6.290479	-1.336535	2.932152

1	4.317882	-2.005179	5.305932
1	3.262049	-3.012998	4.289103
1	5.036094	-3.108367	4.112885
1	2.773321	2.352104	3.572246
1	3.608802	2.484390	5.143270
1	4.476420	1.864103	3.712247
1	0.932165	0.846399	4.491991
1	1.369373	-0.472564	5.605864
1	1.663344	1.229554	6.070846
7	2.372570	-0.468851	-0.776424
6	3.611052	0.153351	-0.191724
6	2.626679	-1.775797	-1.562227
6	1.795757	0.642454	-1.613368
1	3.934883	-0.458335	0.653458
1	4.376039	0.179532	-0.971299
6	3.169687	1.562444	0.196241
6	1.350322	-2.112245	-2.329584
6	3.796962	-1.620660	-2.535516
6	2.948083	-2.874031	-0.552723
1	0.749640	0.390912	-1.813587
6	1.993010	1.892066	-0.752597
1	2.365135	0.698520	-2.546263
1	1.425011	-3.147156	-2.683593
1	0.461927	-2.021004	-1.696862
1	1.222806	-1.464513	-3.205796
1	3.785917	-2.493778	-3.198683
1	3.713853	-0.731045	-3.170081
1	4.764082	-1.609046	-2.021600
1	3.209125	-3.778847	-1.114620
1	3.806714	-2.610032	0.075904
1	2.088512	-3.088264	0.087338
1	2.859601	1.579649	1.245814
1	4.005056	2.260725	0.082836
1	2.199990	2.757630	-1.389423
1	1.086566	2.110084	-0.180274

Calculated Energies for Transition and Ground State Structures 11

Ground State Structure 11a

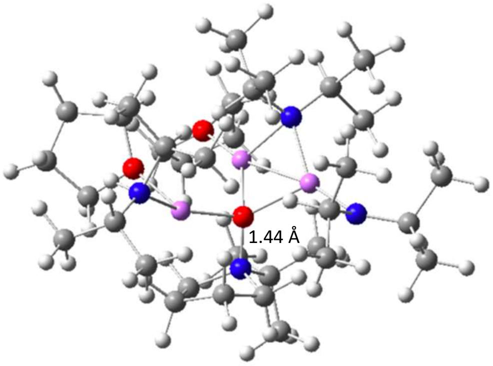

Figure S18. Representative ball and stick model for ground state structure **11a**.

#m062x/jul-cc-pVDZ opt=(calcfc,noeigen) optcyc=50 freq scrf=(pcm,solvent=THF,read) nosymm

EE = -1807.457626 (Hartree)
ZPE_{298} = -1806.380590
E_{298} = -1806.324305
H_{298} = -1806.323360
G_{298} = -1806.466288
S = 300.816 (cal/mol·K)

7	0.405505	0.036393	2.347175
8	-0.013445	0.162599	0.978479
6	-0.241378	1.180211	3.154384
6	-0.072034	-1.319800	2.785859
6	1.888961	-0.025203	2.505185
6	0.925690	-2.258978	2.129245
6	2.264438	-1.498038	2.257343
1	-1.114005	-1.442017	2.465531
1	0.025356	-1.378532	3.871870
1	0.641625	-2.443380	1.083144
1	0.938383	-3.235241	2.626758
1	2.902879	-1.678914	1.372729
1	2.838155	-1.918158	3.098177
1	2.304755	0.690357	1.782484
6	-1.705791	1.325015	2.751192
6	-0.154555	0.886060	4.652556
6	0.519752	2.467376	2.859397
1	-2.220958	0.356676	2.724813
1	-2.208356	1.961854	3.490646
1	-1.817835	1.810789	1.775372
1	-0.909732	0.158274	4.970756
1	0.847666	0.530261	4.912568

1	-0.349266	1.821625	5.190973
1	1.522061	2.434843	3.297718
1	0.611356	2.645321	1.783792
1	-0.039247	3.306310	3.293225
3	1.529276	-0.591182	0.103984
8	2.531747	0.845664	-0.876797
6	3.900813	0.838860	-0.415010
6	4.748501	0.912041	-1.675624
6	3.895019	1.822225	-2.560195
6	2.478015	1.361334	-2.231314
1	4.066505	1.711667	0.232186
1	4.058474	-0.070055	0.180980
1	5.749792	1.307315	-1.478475
1	4.853520	-0.082318	-2.131969
1	4.036888	2.870777	-2.270274
1	4.118074	1.724499	-3.627299
1	1.732550	2.162721	-2.262231
1	2.152848	0.547694	-2.894748
7	2.416168	-2.120530	-1.213966
1	3.091303	-1.409496	-1.495785
6	1.425274	-2.161034	-2.321368
6	3.196099	-3.375490	-1.039934
1	0.878072	-1.208787	-2.256287
6	2.075410	-2.221443	-3.705237
6	0.414849	-3.285615	-2.141415
1	3.163077	-3.946172	-1.980840
6	4.655170	-3.026913	-0.767322
6	2.643914	-4.266715	0.070459
1	2.829015	-1.430259	-3.826546
1	2.562387	-3.189299	-3.881686
1	1.312407	-2.086704	-4.483100
1	-0.065974	-3.241676	-1.156294
1	-0.366196	-3.195187	-2.906217
1	0.889509	-4.268571	-2.259405
1	5.101162	-2.491787	-1.616992
1	4.742489	-2.400119	0.132237
1	5.239074	-3.939415	-0.596806
1	2.859590	-3.839695	1.057255
1	1.561705	-4.409678	-0.012457
1	3.124823	-5.252140	0.013624
7	-3.045481	-1.542740	1.077722
6	-4.362711	-1.822495	1.600845
6	-2.427712	-2.650577	0.377913
1	-4.514102	-2.911907	1.757944
6	-4.541308	-1.159978	2.973102
6	-5.518628	-1.351638	0.692858
1	-1.456206	-2.260930	0.003234
6	-2.065399	-3.867036	1.261282
6	-3.185531	-3.176762	-0.859034
1	-5.533325	-1.364484	3.404587

1	-3.770209	-1.512499	3.672312
1	-4.434709	-0.068509	2.871392
1	-5.465127	-0.258058	0.576145
1	-5.445759	-1.798437	-0.306109
1	-6.504023	-1.607268	1.117076
1	-2.972734	-4.397184	1.584827
1	-1.442049	-4.590226	0.710621
1	-1.519816	-3.550027	2.158044
1	-4.120645	-3.666059	-0.548033
1	-3.441146	-2.351561	-1.536305
1	-2.588501	-3.919234	-1.412373
3	-1.990552	-0.095252	0.423598
3	-0.299991	1.718762	-0.048344
7	-1.899932	1.319115	-1.036090
6	-1.131622	0.909680	-2.209091
6	-3.106477	2.072399	-1.320108
1	-2.886792	2.985603	-1.920583
1	-0.088012	0.731469	-1.853901
6	-4.194520	1.312348	-2.101875
6	-3.707638	2.564811	-0.003444
6	-1.537080	-0.441635	-2.830903
6	-0.987193	1.964063	-3.316976
1	-4.379133	0.336622	-1.629095
1	-5.135126	1.883559	-2.113754
1	-3.901312	1.141495	-3.144425
1	-3.974780	1.708551	0.635950
1	-2.992621	3.192366	0.547446
1	-4.617161	3.155081	-0.180778
1	-2.540515	-0.399739	-3.268622
1	-0.833459	-0.737079	-3.626555
1	-1.536566	-1.225431	-2.062439
1	-0.660260	2.927070	-2.903047
1	-0.244838	1.636516	-4.059193
1	-1.936768	2.121997	-3.845771
8	0.569535	3.362174	-0.539200
6	1.802236	3.916400	-0.059061
6	1.414602	5.135399	0.803332
6	-0.098708	5.308609	0.550404
6	-0.334841	4.466050	-0.697016
1	2.323610	3.127266	0.493118
1	2.412482	4.214904	-0.924520
1	1.626969	4.959369	1.863140
1	1.977223	6.020478	0.489108
1	-0.682663	4.900543	1.384595
1	-0.387615	6.354399	0.405232
1	-1.341848	4.050795	-0.799863
1	-0.066709	5.016816	-1.612909

Ground State Structure 11’

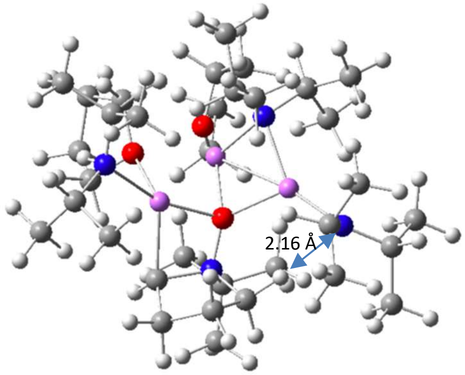

Figure S19. Representative ball and stick model for ground state structure **11’**.

#m062x/jul-cc-pVDZ opt=(calcfc,noeigen) optcyc=50 SCF=XQC freq scrf=(pcm,solvent=THF,read) nosymm

EE = -1807.475263 (Hartree)
ZPE_{298} = -1806.398050
E_{298} = -1806.341963
H_{298} = -1806.341019
G_{298} = -1806.483608
S = 300.104 (cal/mol·K)

7	-0.321388	1.092885	-2.032871
8	-0.302605	0.322175	-0.851116
6	-0.015617	0.145904	-3.208366
6	-1.705346	1.698248	-2.161429
6	0.651457	2.273701	-1.863601
6	-1.564282	3.111284	-1.623146
6	-0.195702	3.507064	-2.173596
1	-2.422148	1.041744	-1.648259
1	-1.953337	1.755466	-3.224388
1	-1.568156	3.106854	-0.523919
1	-2.386712	3.748259	-1.970255
1	0.209289	4.411519	-1.698443
1	-0.311111	3.738649	-3.251703
1	1.442525	2.143215	-2.612183
6	-0.982052	-1.038262	-3.152890
6	-0.164373	0.876126	-4.547898
6	1.424042	-0.357530	-3.102773
1	-0.910997	-1.587279	-4.100839
1	-0.737828	-1.730465	-2.339405
1	-2.018598	-0.708551	-3.018786
1	-1.207930	0.961832	-4.867625
1	0.287392	1.873894	-4.513511
1	0.362243	0.290867	-5.311751
1	2.145267	0.398639	-3.432194

1	1.685981	-0.671612	-2.087404
1	1.525924	-1.223767	-3.767245
3	0.817227	1.579381	0.168390
8	2.436442	0.170339	0.308365
6	3.478002	0.723647	-0.516351
6	4.481103	-0.408176	-0.641541
6	4.464893	-1.007005	0.774797
6	3.103883	-0.568620	1.351392
1	3.018423	1.070123	-1.442342
1	3.929404	1.587651	0.001852
1	4.121823	-1.137517	-1.376672
1	5.472078	-0.055279	-0.944581
1	4.571427	-2.095501	0.757300
1	5.281133	-0.598326	1.380499
1	2.450151	-1.400335	1.628547
1	3.228123	0.085765	2.227387
7	1.713285	2.690210	1.837064
1	2.594232	2.291619	1.511712
6	1.618721	2.290949	3.263485
6	1.797273	4.151920	1.591207
1	1.446070	1.205467	3.250787
6	2.917494	2.546783	4.031240
6	0.429290	2.933539	3.964294
1	2.184209	4.659934	2.489504
6	2.770905	4.400544	0.443669
6	0.424746	4.724152	1.253407
1	3.772100	2.072854	3.527247
1	3.127013	3.620629	4.125659
1	2.846450	2.128538	5.043734
1	-0.500333	2.755727	3.411084
1	0.323396	2.498370	4.966066
1	0.572147	4.015792	4.083577
1	3.786357	4.077023	0.713440
1	2.439558	3.843770	-0.446441
1	2.808492	5.468779	0.198021
1	0.040057	4.229967	0.353909
1	-0.295851	4.581531	2.064308
1	0.505571	5.801188	1.054137
7	-3.654663	-0.238634	-0.426772
6	-4.784060	-0.800787	-1.126295
6	-3.983683	0.874779	0.444160
1	-5.732230	-0.663059	-0.559601
6	-5.030866	-0.192255	-2.528204
6	-4.604563	-2.310957	-1.306114
1	-3.015798	1.312302	0.769990
6	-4.760731	2.048747	-0.190327
6	-4.717191	0.455791	1.736196
1	-5.926024	-0.623600	-3.006596
1	-5.157109	0.894917	-2.482280
1	-4.164896	-0.402149	-3.175116

1	-3.682029	-2.501112	-1.877675
1	-4.507840	-2.806201	-0.331742
1	-5.444208	-2.762286	-1.856943
1	-5.777507	1.735523	-0.470039
1	-4.852508	2.883467	0.521305
1	-4.255277	2.417106	-1.092251
1	-5.702685	0.030525	1.494312
1	-4.135863	-0.308536	2.265840
1	-4.878822	1.310255	2.413314
3	-1.810273	-0.639743	-0.043288
3	0.733049	-1.179982	-0.170575
7	-0.553884	-1.671609	1.232914
6	-0.189322	-0.940833	2.442631
6	-1.099612	-3.000749	1.439626
1	-0.404395	-3.652387	2.019284
1	0.553395	-0.183224	2.120994
6	-2.440960	-3.086712	2.194273
6	-1.269755	-3.655268	0.066391
6	-1.312114	-0.114014	3.098930
6	0.507466	-1.775119	3.530574
1	-2.327480	-2.816159	3.251158
1	-3.181465	-2.413870	1.738050
1	-2.831060	-4.116060	2.158565
1	-2.058419	-3.138163	-0.498135
1	-0.337429	-3.592335	-0.511830
1	-1.557974	-4.712711	0.153832
1	-1.749831	0.583880	2.371973
1	-2.117115	-0.754814	3.476689
1	-0.920183	0.471770	3.944568
1	1.298948	-2.411160	3.110917
1	0.958166	-1.119359	4.289990
1	-0.207263	-2.432483	4.045152
8	2.054322	-2.725217	-0.601520
6	2.157349	-3.724072	0.439865
6	2.175567	-5.070587	-0.271518
6	2.877483	-4.713691	-1.582466
6	2.288640	-3.342223	-1.883399
1	3.097412	-3.561034	0.986335
1	1.312318	-3.581917	1.118467
1	2.701937	-5.834502	0.309918
1	1.152532	-5.417664	-0.464190
1	3.961549	-4.634330	-1.426782
1	2.690449	-5.428864	-2.389958
1	2.959960	-2.697812	-2.462097
1	1.328738	-3.424464	-2.416140

Transition Structure 11a

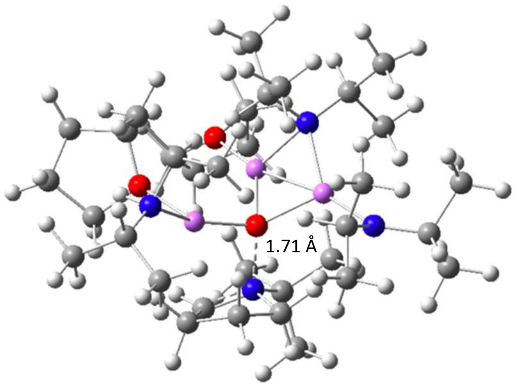

Figure S20. Representative ball and stick model of transition structure **11a**.

#m062x/jul-cc-pVDZ opt=(calcfc,noeigen,ts) optcyc=50 freq scrf=(pcm,solvent=THF,read) nosymm

EE = -1807.450615 (Hartree)
ZPE_{298} = -1806.375110
E_{298} = -1806.318895
H_{298} = -1806.317951
G_{298} = -1806.460943
S = 300.952 (cal/mol·K)

7	0.508170	-0.001326	2.459772
8	-0.027956	0.122221	0.842420
6	-0.151104	1.182829	3.155702
6	-0.070348	-1.338402	2.799565
6	1.866371	-0.121488	2.573832
6	0.946467	-2.304174	2.205375
6	2.291663	-1.545814	2.302272
1	-1.079147	-1.418106	2.380868
1	-0.086908	-1.423359	3.891394
1	0.682199	-2.522721	1.162698
1	0.957076	-3.257717	2.744181
1	2.883049	-1.686390	1.376837
1	2.923483	-1.956518	3.105562
1	2.474118	0.742739	2.323416
6	-1.604576	1.337514	2.715500
6	-0.120934	0.952258	4.672609
6	0.638723	2.443912	2.825388
1	-2.125938	0.373430	2.677168
1	-2.117196	1.978500	3.445579
1	-1.692836	1.818523	1.735983
1	-0.878972	0.226216	4.988116
1	0.870115	0.617691	4.996244
1	-0.351906	1.905503	5.163796
1	1.604463	2.463998	3.343225

1	0.808827	2.522566	1.746609
1	0.055064	3.312985	3.153227
3	1.459101	-0.600417	0.024365
8	2.622603	0.809245	-0.791865
6	3.977947	0.811276	-0.300252
6	4.852992	0.950621	-1.537555
6	3.994490	1.867575	-2.411050
6	2.586859	1.348069	-2.135813
1	4.110414	1.663053	0.382621
1	4.140725	-0.116757	0.263480
1	5.838691	1.365331	-1.304562
1	4.992792	-0.024608	-2.024789
1	4.093063	2.908150	-2.076806
1	4.248409	1.818389	-3.474587
1	1.809543	2.118637	-2.175180
1	2.316196	0.536601	-2.826654
7	2.372605	-2.128304	-1.264646
1	3.056983	-1.413429	-1.513615
6	1.398530	-2.138176	-2.388014
6	3.139489	-3.392138	-1.104289
1	0.863494	-1.177681	-2.322665
6	2.066581	-2.195280	-3.763501
6	0.368310	-3.247316	-2.229783
1	3.103591	-3.952462	-2.051177
6	4.600934	-3.061420	-0.823173
6	2.573853	-4.288316	-0.005393
1	2.835325	-1.416320	-3.865817
1	2.538513	-3.169669	-3.944920
1	1.316034	-2.038012	-4.549171
1	-0.119400	-3.200794	-1.248511
1	-0.404800	-3.137703	-3.000111
1	0.828278	-4.236966	-2.351534
1	5.052596	-2.516091	-1.663320
1	4.690031	-2.448985	0.086145
1	5.177178	-3.981019	-0.665072
1	2.781371	-3.866412	0.985678
1	1.491789	-4.425559	-0.101170
1	3.051231	-5.275540	-0.061853
7	-3.004025	-1.554359	1.060047
6	-4.334569	-1.794181	1.567832
6	-2.379760	-2.707515	0.444379
1	-4.495660	-2.869884	1.796714
6	-4.542527	-1.037108	2.886512
6	-5.470991	-1.384856	0.605697
1	-1.389545	-2.350505	0.091874
6	-2.073456	-3.884317	1.401984
6	-3.099462	-3.290296	-0.791471
1	-5.552823	-1.190268	3.297075
1	-3.803288	-1.356303	3.634758
1	-4.405577	0.042084	2.713871

1	-5.432661	-0.297339	0.440125
1	-5.361849	-1.875785	-0.368874
1	-6.464130	-1.637631	1.013859
1	-2.998655	-4.396934	1.702866
1	-1.427115	-4.634030	0.917234
1	-1.571752	-3.529150	2.310592
1	-4.056414	-3.743226	-0.491567
1	-3.311380	-2.502993	-1.527178
1	-2.497583	-4.075765	-1.276162
3	-1.924661	-0.115710	0.370985
3	-0.343944	1.689033	-0.083636
7	-1.962845	1.348564	-1.070207
6	-1.208941	0.951810	-2.254851
6	-3.181343	2.085012	-1.325107
1	-2.989990	3.014341	-1.914045
1	-0.155918	0.789135	-1.920854
6	-4.267122	1.317008	-2.103262
6	-3.764806	2.541504	0.012784
6	-1.604024	-0.410875	-2.859001
6	-1.100766	2.006223	-3.366842
1	-4.412016	0.325503	-1.650450
1	-5.223570	1.861764	-2.084020
1	-3.988433	1.177645	-3.154821
1	-3.989669	1.666435	0.642188
1	-3.050118	3.175120	0.557348
1	-4.693039	3.112263	-0.130353
1	-2.615805	-0.390498	-3.279925
1	-0.907967	-0.705261	-3.662287
1	-1.577806	-1.184751	-2.080011
1	-0.770122	2.970244	-2.957926
1	-0.373434	1.685448	-4.126925
1	-2.063799	2.159746	-3.872613
8	0.524086	3.363236	-0.587229
6	1.757815	3.957157	-0.167305
6	1.371315	5.125980	0.761795
6	-0.152681	5.275491	0.556549
6	-0.413906	4.444083	-0.694675
1	2.354454	3.174141	0.313644
1	2.292305	4.320365	-1.058406
1	1.615289	4.905499	1.806330
1	1.907882	6.036417	0.475361
1	-0.704032	4.846884	1.402521
1	-0.464316	6.317736	0.434067
1	-1.412165	4.003204	-0.768436
1	-0.192859	5.016198	-1.610414

Transition Structure 11b

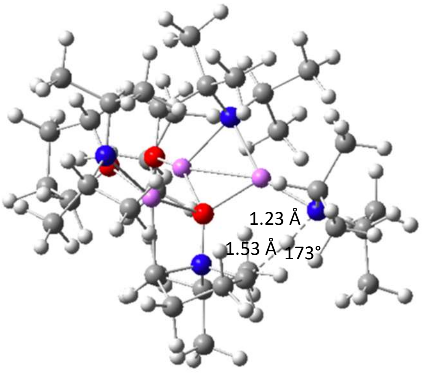

Figure S21. Representative ball and stick model from transition structure **11b**.

#m062x/jul-cc-pVDZ opt=(calcfc,noeigen,ts) optcyc=50 freq scrf=(pcm,solvent=THF,read) nosymm

EE = -1807.451701 (Hartree)
ZPE_{298} = -1806.380436
E_{298} = -1806.324302
H_{298} = -1806.323358
G_{298} = -1806.466290
S = 300.827 (cal/mol·K)

7	-0.401295	0.973410	-2.015815
8	-0.274806	0.198937	-0.827496
6	-0.080626	0.022762	-3.180154
6	-1.832314	1.506899	-2.095737
6	0.551382	2.178644	-1.881030
6	-1.680987	2.972883	-1.698024
6	-0.314252	3.377116	-2.254346
1	-2.819458	0.640075	-1.302891
1	-2.084805	1.505645	-3.164157
1	-1.695551	3.102092	-0.601476
1	-2.509171	3.570117	-2.106536
1	0.076264	4.311788	-1.819460
1	-0.423572	3.553313	-3.344598
1	1.355593	2.032592	-2.615113
6	-1.014644	-1.186634	-3.104389
6	-0.256569	0.725554	-4.530941
6	1.371773	-0.454687	-3.096958
1	-0.926745	-1.758921	-4.037740
1	-0.755431	-1.852088	-2.272447
1	-2.054882	-0.867108	-2.984999
1	-1.305970	0.800232	-4.831403
1	0.184503	1.728191	-4.515365
1	0.265653	0.132868	-5.293490
1	2.073702	0.313540	-3.440584

1	1.656908	-0.758784	-2.085253
1	1.481060	-1.322032	-3.759724
3	0.753331	1.522527	0.141588
8	2.431171	0.080277	0.343591
6	3.429703	0.660997	-0.513232
6	4.453310	-0.447580	-0.681129
6	4.494168	-1.063250	0.728287
6	3.151171	-0.642826	1.359588
1	2.928745	1.003308	-1.419771
1	3.881017	1.531416	-0.005100
1	4.087513	-1.176209	-1.413600
1	5.426754	-0.069825	-1.010135
1	4.607131	-2.150860	0.692833
1	5.329042	-0.656503	1.309360
1	2.522941	-1.483954	1.667203
1	3.301860	0.017493	2.227504
7	1.647610	2.627359	1.799288
1	2.532118	2.237496	1.473763
6	1.556918	2.231927	3.225698
6	1.708751	4.089132	1.547110
1	1.410929	1.142244	3.215922
6	2.845229	2.520439	3.999825
6	0.349354	2.851297	3.916758
1	2.070874	4.607696	2.449756
6	2.695410	4.350929	0.413881
6	0.331653	4.633125	1.181326
1	3.712709	2.063865	3.502065
1	3.029938	3.599065	4.091288
1	2.778257	2.104410	5.013595
1	-0.573937	2.643259	3.363303
1	0.251941	2.426852	4.924007
1	0.463896	3.938412	4.021770
1	3.712129	4.044183	0.698597
1	2.382265	3.787286	-0.478535
1	2.718685	5.419139	0.165921
1	-0.012606	4.149451	0.259321
1	-0.406870	4.452138	1.968769
1	0.389696	5.716567	1.009717
7	-3.536727	-0.010010	-0.548088
6	-4.672619	-0.604889	-1.255677
6	-3.913658	0.964955	0.481983
1	-5.595613	-0.442326	-0.673333
6	-4.906166	0.017689	-2.642564
6	-4.502212	-2.117965	-1.396045
1	-2.959114	1.410936	0.818734
6	-4.773989	2.131125	-0.027144
6	-4.574494	0.318508	1.701847
1	-5.812092	-0.399279	-3.105821
1	-5.016187	1.106820	-2.575862
1	-4.056585	-0.188768	-3.308568

1	-3.583793	-2.343206	-1.958905
1	-4.422239	-2.591371	-0.408637
1	-5.349420	-2.567689	-1.934567
1	-5.765932	1.779835	-0.345954
1	-4.923788	2.873321	0.770019
1	-4.287884	2.624272	-0.877236
1	-5.552754	-0.107833	1.436609
1	-3.946202	-0.485642	2.101742
1	-4.741207	1.060929	2.496114
3	-1.715211	-0.699095	-0.019506
3	0.766797	-1.261855	-0.149211
7	-0.549297	-1.794521	1.248190
6	-0.158699	-1.081560	2.462373
6	-1.075815	-3.131975	1.441789
1	-0.371654	-3.781648	2.011140
1	0.584188	-0.326199	2.137829
6	-2.411462	-3.240129	2.201144
6	-1.245685	-3.774314	0.063424
6	-1.266146	-0.255259	3.144251
6	0.546750	-1.932908	3.530475
1	-2.305103	-2.951840	3.253719
1	-3.166857	-2.587910	1.739290
1	-2.784433	-4.275795	2.177430
1	-2.035730	-3.252811	-0.496477
1	-0.314894	-3.700047	-0.515607
1	-1.530900	-4.833150	0.139924
1	-1.703051	0.460320	2.434010
1	-2.073518	-0.894541	3.520833
1	-0.860226	0.314279	3.994332
1	1.331982	-2.565185	3.094263
1	1.008256	-1.287140	4.291656
1	-0.163484	-2.594611	4.045911
8	2.079566	-2.809334	-0.592738
6	2.198278	-3.808760	0.444988
6	2.226453	-5.154089	-0.268790
6	2.913177	-4.786876	-1.584884
6	2.305845	-3.421836	-1.878149
1	3.138894	-3.636740	0.988151
1	1.354695	-3.678006	1.127654
1	2.765941	-5.913030	0.307160
1	1.205863	-5.512558	-0.453908
1	3.997523	-4.695143	-1.438051
1	2.727730	-5.502940	-2.391969
1	2.962519	-2.768708	-2.463572
1	1.340891	-3.513566	-2.400130

Structures for the Analysis of Aggregation on the First Deprotonation S1-S3

Ground State Structure S1

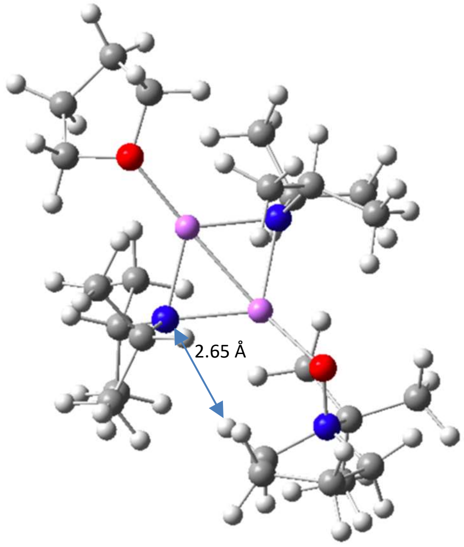

Figure S22. Representative ball and stick model for ground state structure **S1**.

#m062x/jul-cc-pvDZ opt=(calcfc,noeigen,z-matrix) optcyc=50 freq scrf=(pcm,solvent=THF,read)

EE = -1275.820120 (Hartree)
ZPE_{298} = -1275.059163
E_{298} = -1275.020173
H_{298} = -1275.019229
G_{298} = -1275.127786
S = 228.477 (cal/mol·K)

7	3.388031	0.130287	0.113554
8	2.320751	0.532245	0.880018
6	3.442819	0.967527	-1.181697
6	4.623484	0.255956	0.960762
6	3.288662	-1.354823	-0.107947
6	4.535830	-0.921580	1.936906
6	3.566842	-1.930870	1.277362
1	4.589820	1.235668	1.438341
1	5.503291	0.181338	0.313267
1	4.152647	-0.592252	2.906449
1	5.530080	-1.351613	2.095495
1	2.636922	-2.003379	1.847643
1	3.998348	-2.933979	1.194706
1	2.288445	-1.576685	-0.494103
1	4.065572	-1.653095	-0.819029

6	3.828522	2.393022	-0.794642
6	4.440124	0.387843	-2.185845
6	2.044381	0.972358	-1.795619
1	4.892918	2.478078	-0.544950
1	3.629134	3.049080	-1.650529
1	3.219089	2.727179	0.053036
1	5.424594	0.187089	-1.747942
1	4.062005	-0.528200	-2.653913
1	4.581074	1.131529	-2.979448
1	1.627366	-0.039689	-1.872489
1	1.363910	1.604717	-1.215876
1	2.114524	1.384434	-2.809965
3	0.566374	0.201269	0.259625
7	-0.340152	-1.552781	-0.157272
6	-0.225804	-2.489118	0.961224
6	-0.767425	-2.147242	-1.413947
1	0.293821	-1.934574	1.766509
6	-1.587203	-2.912554	1.553226
6	0.596828	-3.768867	0.724722
1	-1.499457	-2.969877	-1.249498
6	-1.470045	-1.082145	-2.268931
6	0.357816	-2.741654	-2.291039
1	-1.459456	-3.600203	2.403308
1	-2.153546	-2.039496	1.902921
1	-2.194102	-3.430616	0.795249
1	0.738355	-4.307799	1.672981
1	0.073962	-4.446232	0.033353
1	1.585647	-3.548689	0.305249
1	-1.769427	-1.480562	-3.248510
1	-2.376102	-0.678719	-1.790963
1	-0.780405	-0.241981	-2.440546
1	0.944347	-3.498823	-1.763106
1	-0.058823	-3.206305	-3.198516
1	1.043964	-1.942337	-2.610133
3	-1.744867	-0.179974	0.231771
7	-0.907269	1.578187	0.351389
6	-1.178415	2.412700	-0.811863
6	-1.078663	2.223409	1.643730
1	-0.810452	1.844398	-1.686259
6	-2.680737	2.638833	-1.077424
6	-0.463610	3.774769	-0.868718
1	-1.914079	2.958867	1.630960
6	-1.439532	1.153993	2.684362
6	0.173454	2.960126	2.165785
1	-3.190797	1.673504	-1.205863
1	-3.152305	3.170816	-0.237150
1	-2.845538	3.238185	-1.986155
1	0.617835	3.664169	-0.709398
1	-0.625108	4.250481	-1.847244
1	-0.853436	4.456440	-0.098699

1	-2.390437	0.658356	2.445306
1	-0.650177	0.383738	2.706026
1	-1.521069	1.573497	3.696862
1	0.465978	3.790975	1.516142
1	0.003685	3.363637	3.176976
1	1.013376	2.250181	2.201901
8	-3.632673	-0.488129	0.466011
6	-4.395290	-1.504978	-0.187180
6	-5.383203	-0.720031	-1.046563
6	-5.666379	0.537684	-0.193549
6	-4.575990	0.502845	0.893508
1	-3.701401	-2.140608	-0.747975
1	-4.911538	-2.113118	0.572980
1	-4.906662	-0.443753	-1.995311
1	-6.287315	-1.295603	-1.270287
1	-5.598785	1.450491	-0.795097
1	-6.663145	0.504180	0.258640
1	-4.029359	1.443343	1.013668
1	-4.989977	0.193929	1.865121

Transition Structure S1

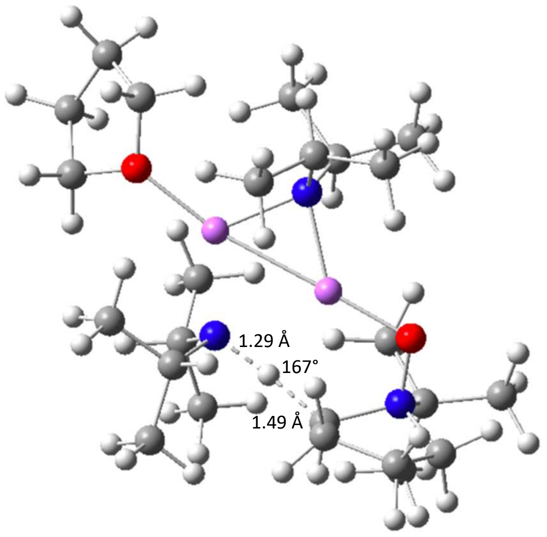

Figure S23. Representative ball and stick model for transition structure **S1**.

#m062x/jul-cc-pvDZ opt=(calcfc,noeigen,z-matrix,ts) optcyc=50 freq scrf=(pcm,solvent=THF,read)

EE = -1275.784886 (Hartree)
ZPE_{298} = -1275.029483
E_{298} = -1274.990374
H_{298} = -1274.989430
G_{298} = -1275.100426

S = 233.611 (cal/mol·K)

7	3.279279	0.307092	0.113456
8	2.470364	1.345393	0.550708
6	3.532710	0.482409	-1.391011
6	4.543970	0.358501	0.925452
6	2.613184	-1.007696	0.488701
6	4.172256	-0.278826	2.266812
6	2.829397	-1.007932	2.007884
1	4.850964	1.404023	0.997879
1	5.313292	-0.219519	0.403704
1	4.072789	0.481494	3.048473
1	4.957128	-0.976960	2.579695
1	2.025342	-0.474940	2.532814
1	2.837240	-2.035798	2.391831
1	1.190617	-1.260415	0.104760
1	3.255939	-1.796641	0.064878
6	4.409582	1.717126	-1.593565
6	4.207860	-0.748951	-1.997020
6	2.179694	0.709236	-2.062260
1	5.453214	1.530016	-1.313023
1	4.389717	1.987511	-2.656590
1	4.015420	2.556146	-1.010242
1	5.117497	-1.039760	-1.459013
1	3.530440	-1.607653	-2.036165
1	4.499403	-0.498357	-3.024820
1	1.460186	-0.057119	-1.751868
1	1.783694	1.700179	-1.819834
1	2.307064	0.633674	-3.149845
3	0.735079	0.719368	0.430199
7	-0.063046	-1.559083	0.058007
6	-0.143639	-2.413355	1.265043
6	-0.412694	-2.263788	-1.190454
1	0.303549	-1.807558	2.068545
6	-1.576046	-2.733683	1.703838
6	0.664284	-3.717223	1.195518
1	-1.101544	-3.094393	-0.954297
6	-1.150276	-1.329958	-2.158698
6	0.788068	-2.869958	-1.938899
1	-1.563479	-3.349920	2.613615
1	-2.149645	-1.824757	1.924062
1	-2.114782	-3.301046	0.931467
1	0.662498	-4.208217	2.178438
1	0.223859	-4.418844	0.472637
1	1.705237	-3.529255	0.906698
1	-1.370102	-1.845450	-3.103611
1	-2.107209	-0.968750	-1.756208
1	-0.531066	-0.453450	-2.394272
1	1.433844	-3.459019	-1.280259

1	0.435589	-3.520145	-2.751864
1	1.398387	-2.076686	-2.390449
3	-1.586749	-0.192204	0.240895
7	-0.985213	1.652544	0.328208
6	-1.134860	2.359176	-0.940712
6	-1.402950	2.362021	1.527020
1	-0.627517	1.724242	-1.691348
6	-2.592303	2.478198	-1.426417
6	-0.465199	3.740039	-1.049086
1	-2.336682	2.944559	1.360104
6	-1.702136	1.324516	2.618122
6	-0.361970	3.340671	2.111617
1	-3.043212	1.481074	-1.528909
1	-3.198527	3.063589	-0.718596
1	-2.646039	2.982105	-2.403260
1	0.567682	3.706125	-0.677373
1	-0.451586	4.073836	-2.097094
1	-1.016850	4.494437	-0.469541
1	-2.521056	0.650952	2.330584
1	-0.801303	0.710547	2.791275
1	-1.972450	1.795411	3.573015
1	-0.142095	4.173717	1.438483
1	-0.714603	3.761487	3.066243
1	0.579247	2.800616	2.297327
8	-3.495202	-0.557483	0.255604
6	-4.233037	-1.646223	-0.305442
6	-5.229929	-0.965028	-1.239545
6	-5.536397	0.366163	-0.515243
6	-4.471242	0.439182	0.592767
1	-3.527136	-2.318893	-0.804348
1	-4.740956	-2.192479	0.505466
1	-4.754394	-0.775936	-2.209685
1	-6.123097	-1.575532	-1.406966
1	-5.455798	1.218085	-1.198857
1	-6.543025	0.372368	-0.084453
1	-3.949249	1.397951	0.658170
1	-4.899806	0.187002	1.574612

Ground State Structure S2

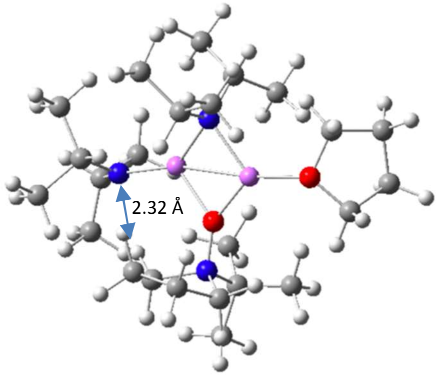

Figure S24. Representative ball and stick model for ground state structure **S2**.

#m062x/jul-cc-pvDZ opt=(calcfc,noeigen,z-matrix) optcyc=50 freq scrf=(pcm,solvent=THF,read)

EE = -1275.809935 (Hartree)
ZPE_{298} = -1275.049925
E_{298} = -1275.010566
H_{298} = -1275.009622
G_{298} = -1275.120024
S = 232.360 (cal/mol·K)

7	-0.289488	-2.211131	0.565446
8	0.175834	-0.945688	0.318336
6	-0.246187	-3.034133	-0.738371
6	0.522156	-2.784633	1.691650
6	-1.661344	-2.080268	1.179635
6	0.042604	-2.012102	2.924611
6	-1.363687	-1.483237	2.552177
1	1.577054	-2.637501	1.453273
1	0.301513	-3.854424	1.765492
1	0.722826	-1.185349	3.150261
1	0.018128	-2.674759	3.795323
1	-1.366688	-0.390065	2.493446
1	-2.128846	-1.788257	3.273203
1	-2.263275	-1.424522	0.535104
1	-2.086298	-3.083744	1.272053
6	1.212097	-3.353282	-1.053763
6	-1.058338	-4.321760	-0.598820
6	-0.834088	-2.163813	-1.846743
1	1.632415	-4.105485	-0.375186
1	1.258420	-3.754471	-2.072917
1	1.817910	-2.440090	-1.021516
1	-0.805424	-4.892696	0.302525
1	-2.135812	-4.123972	-0.609242
1	-0.827818	-4.954510	-1.463979
1	-1.740487	-1.646663	-1.515549

1	-0.113870	-1.407572	-2.174587
1	-1.079770	-2.809215	-2.699329
3	-0.838939	0.605171	-0.290339
7	-2.746570	0.619833	-0.457870
6	-3.103081	1.444263	0.682934
6	-3.845400	0.368416	-1.362938
1	-2.179594	1.553820	1.291413
6	-3.531862	2.884415	0.325281
6	-4.160270	0.838076	1.629813
1	-4.665582	1.102061	-1.220186
6	-3.393598	0.518184	-2.822229
6	-4.505637	-1.018726	-1.182600
1	-3.635128	3.509987	1.226020
1	-2.783845	3.342071	-0.336394
1	-4.500840	2.893254	-0.194135
1	-4.299896	1.458802	2.528610
1	-5.132752	0.763234	1.120196
1	-3.863055	-0.171907	1.944946
1	-4.193506	0.261939	-3.534761
1	-3.068744	1.550461	-3.011429
1	-2.536184	-0.144203	-3.016119
1	-4.810966	-1.168322	-0.139239
1	-5.394619	-1.133046	-1.824210
1	-3.800112	-1.822263	-1.444535
3	1.647511	0.128991	0.322965
7	0.743214	1.838927	0.052699
6	0.698212	2.451059	1.379220
6	1.213914	2.679516	-1.032807
1	0.180653	1.710914	2.022153
6	2.083723	2.669613	2.024002
6	-0.099444	3.758404	1.522267
1	2.058398	3.334911	-0.717453
6	1.737603	1.770383	-2.153260
6	0.151638	3.603037	-1.667767
1	2.633379	1.722638	2.117096
1	2.690794	3.360951	1.420753
1	1.987792	3.106038	3.029868
1	-1.088100	3.670056	1.058060
1	-0.231929	4.007809	2.585539
1	0.435364	4.596167	1.050937
1	2.550957	1.117583	-1.804508
1	0.915765	1.125729	-2.504598
1	2.107201	2.345957	-3.013758
1	-0.686355	2.992121	-2.037906
1	-0.251814	4.329110	-0.955978
1	0.575344	4.160793	-2.518106
8	3.566441	-0.117893	0.479389
6	4.336081	-1.291781	0.200979
6	4.967295	-0.999519	-1.154509
6	5.261229	0.513285	-1.069328

6	4.401016	0.997522	0.114886
1	5.100486	-1.415014	0.984303
1	3.662622	-2.154775	0.216231
1	5.865119	-1.599115	-1.336121
1	4.242299	-1.209820	-1.950659
1	6.321959	0.703076	-0.874507
1	4.992689	1.027804	-1.997984
1	5.021724	1.263741	0.982408
1	3.738695	1.835960	-0.121848

Transition Structure S2

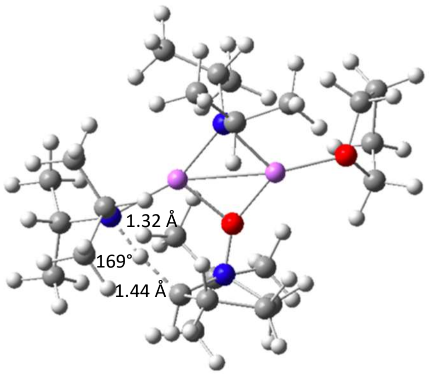

Figure S25. Representative ball and stick model for transition structure **S2**.

#m062x/jul-cc-pvDZ opt=(calcfc,noeigen,z-matrix,ts) optcyc=50 freq scrf=(pcm,solvent=THF,read)

EE = -1275.790609 (Hartree)
ZPE_{298} = -1275.035592
E_{298} = -1274.996458
H_{298} = -1274.995514
G_{298} = -1275.105078
S = 230.597 (cal/mol·K)

7	-0.505291	-2.088272	0.570676
8	0.198153	-0.929943	0.310683
6	-0.526522	-2.943255	-0.707712
6	0.174465	-2.773161	1.724076
6	-1.875642	-1.689151	1.096408
6	-0.236427	-1.954591	2.946304
6	-1.528057	-1.213385	2.511730
1	1.251073	-2.786634	1.536259
1	-0.204840	-3.798442	1.776023
1	0.554504	-1.249148	3.224297
1	-0.405265	-2.617066	3.802391
1	-1.352914	-0.129120	2.536696
1	-2.363028	-1.418692	3.192779

1	-2.391975	-0.584947	0.333982
1	-2.447196	-2.623903	1.173446
6	0.873970	-3.504005	-0.948248
6	-1.538597	-4.083485	-0.591627
6	-0.907582	-2.033176	-1.872069
1	1.133179	-4.301903	-0.241921
1	0.903478	-3.926391	-1.959895
1	1.617517	-2.700583	-0.891741
1	-1.414396	-4.671091	0.325482
1	-2.567767	-3.713497	-0.640766
1	-1.378959	-4.758249	-1.441713
1	-1.743130	-1.384880	-1.599762
1	-0.061504	-1.407305	-2.173500
1	-1.201586	-2.659436	-2.724145
3	-0.660928	0.655650	-0.309809
7	-2.667653	0.547918	-0.286205
6	-2.908971	1.578742	0.730335
6	-3.786260	0.395777	-1.220399
1	-1.977291	1.652298	1.324172
6	-3.149713	2.974428	0.139922
6	-4.033538	1.219065	1.714635
1	-4.592763	1.098474	-0.952547
6	-3.380100	0.741283	-2.657600
6	-4.433749	-0.996855	-1.156924
1	-3.195366	3.729897	0.937823
1	-2.339469	3.244826	-0.549948
1	-4.099309	3.019095	-0.410931
1	-4.122428	1.974371	2.509209
1	-5.001121	1.164772	1.194732
1	-3.839357	0.244091	2.178775
1	-4.225590	0.631049	-3.353695
1	-3.020288	1.777593	-2.706842
1	-2.567641	0.083571	-2.999511
1	-4.751268	-1.224062	-0.130693
1	-5.314474	-1.038822	-1.813647
1	-3.740977	-1.787331	-1.474727
3	1.675223	0.096375	0.524291
7	0.920014	1.845756	0.022011
6	0.964347	2.607232	1.268784
6	1.446566	2.508906	-1.157656
1	0.417848	1.986251	2.006424
6	2.384400	2.792979	1.843827
6	0.266920	3.978183	1.278771
1	2.343999	3.125273	-0.924760
6	1.879312	1.437126	-2.167452
6	0.448786	3.431846	-1.888780
1	2.872689	1.824694	2.019979
1	3.013805	3.372939	1.152834
1	2.355509	3.337398	2.799706
1	-0.742530	3.908868	0.857581

1	0.192176	4.359812	2.307729
1	0.839559	4.713521	0.694642
1	2.604596	0.735541	-1.732361
1	0.997541	0.853183	-2.477129
1	2.322941	1.878264	-3.071319
1	-0.436239	2.843310	-2.178997
1	0.109355	4.261924	-1.262035
1	0.895878	3.854581	-2.802460
8	3.563044	-0.305390	0.640092
6	4.107973	-1.603917	0.384355
6	4.655677	-1.494026	-1.031764
6	5.223083	-0.060835	-1.055383
6	4.507761	0.646305	0.114481
1	4.908039	-1.811775	1.112192
1	3.305300	-2.337839	0.510685
1	5.410693	-2.255741	-1.251616
1	3.834461	-1.599254	-1.752388
1	6.306445	-0.064078	-0.894570
1	5.025868	0.438065	-2.010109
1	5.210725	0.918133	0.914272
1	3.940936	1.534978	-0.181474

Ground State Structure S3

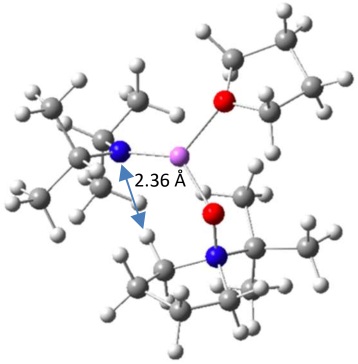

Figure S26. Representative ball and stick model for ground state structure **S3**.

#m062x/jul-cc-pvDZ opt=(calcfc,noeigen,z-matrix) optcyc=50 freq scrf=(pcm,solvent=THF,read)

EE = -976.519032 (Hartree)
ZPE_{298} = -975.956177
E_{298} = -975.927746
H_{298} = -975.926801
G_{298} = -976.014516
S = 184.611 (cal/mol·K)

7	-1.568712	-1.392399	-0.138379
8	-1.183273	-0.381956	-0.988898
6	-2.124832	-0.787581	1.167419
6	-2.559950	-2.247226	-0.876093
6	-0.408268	-2.333663	0.060940
6	-1.707731	-3.037810	-1.874169
6	-0.270051	-3.001925	-1.303186
1	-3.288252	-1.580168	-1.339189
1	-3.056687	-2.905142	-0.155227
1	-1.744940	-2.575316	-2.864099
1	-2.088597	-4.060253	-1.965271
1	0.388988	-2.411837	-1.946351
1	0.162615	-4.002181	-1.195447
1	0.466226	-1.742730	0.363886
1	-0.690365	-3.066416	0.823390
6	-3.470829	-0.140182	0.850250
6	-2.287213	-1.851990	2.253823
6	-1.141373	0.279526	1.643287
1	-4.264129	-0.884286	0.710641
1	-3.752991	0.499025	1.695554
1	-3.389412	0.483990	-0.046855
1	-2.831339	-2.738469	1.907128
1	-1.321214	-2.162656	2.667348
1	-2.870777	-1.407434	3.069034
1	-0.106839	-0.085265	1.632665
1	-1.208077	1.178203	1.019557
1	-1.402464	0.552856	2.673236
3	0.404497	0.571609	-0.603029
7	2.063502	-0.027368	0.070794
6	2.726941	-0.612024	-1.081228
6	2.870016	0.001790	1.269132
1	1.961627	-0.679436	-1.883117
6	3.856041	0.265002	-1.663190
6	3.260116	-2.047497	-0.893794
1	3.954752	-0.046431	1.033905
6	2.654617	1.314220	2.031645
6	2.592836	-1.171963	2.236725
1	4.293625	-0.178200	-2.571776
1	3.459846	1.258199	-1.912275
1	4.666495	0.394402	-0.930577
1	3.647024	-2.453626	-1.840832
1	4.082397	-2.063190	-0.162910
1	2.467321	-2.714376	-0.528064
1	3.221259	1.343174	2.975205
1	2.954227	2.168027	1.409351
1	1.585625	1.428703	2.271957
1	2.715755	-2.137499	1.731349
1	3.265020	-1.149856	3.110111
1	1.556728	-1.115934	2.607269
8	-0.127063	2.417682	-0.966726

6	0.347919	3.481530	-0.117740
6	-0.723737	4.569398	-0.168827
6	-1.989460	3.762166	-0.470500
6	-1.453639	2.716969	-1.438421
1	0.472369	3.079490	0.897568
1	1.325690	3.812491	-0.487184
1	-0.779104	5.135667	0.766719
1	-0.520271	5.270573	-0.988474
1	-2.367214	3.280331	0.442212
1	-2.793791	4.363780	-0.907105
1	-2.009852	1.773528	-1.453912
1	-1.384013	3.123566	-2.458695

Transition Structure S3

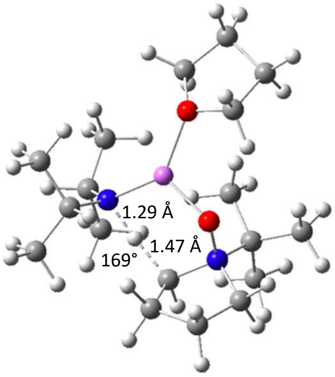

Figure S27. Representative ball and stick model for transition structure **S3**.

#m062x/jul-cc-pvDZ opt=(calcfc,noeigen,z-matrix,ts) optcyc=50 freq scrf=(pcm,solvent=THF,read)

EE = -976.496303 (Hartree)
ZPE_{298} = -975.938235
E_{298} = -975.910195
H_{298} = -975.909251
G_{298} = -975.995285
S = 181.075 (cal/mol·K)

7	0.986391	-1.758684	-0.202362
8	0.031029	-1.252584	-1.073065
6	0.285617	-2.229084	1.083879
6	1.671208	-2.890468	-0.919560
6	2.064524	-0.709626	0.018887
6	2.650936	-2.209776	-1.873567
6	2.836606	-0.779139	-1.303339
1	0.905857	-3.490037	-1.418026
1	2.194262	-3.499149	-0.174996
1	2.247133	-2.184143	-2.891192

1	3.593917	-2.767798	-1.904583
1	2.460969	-0.048553	-2.030785
1	3.894847	-0.543134	-1.131504
1	1.440915	0.613886	0.183130
1	2.711671	-1.114855	0.810334
6	-0.571407	-3.450754	0.752610
6	1.289652	-2.572706	2.186445
6	-0.617663	-1.100191	1.573685
1	0.034020	-4.354511	0.614180
1	-1.258232	-3.630733	1.589120
1	-1.161336	-3.263194	-0.151567
1	2.081145	-3.250549	1.847296
1	1.749727	-1.672106	2.606718
1	0.741356	-3.080212	2.990336
1	-0.072731	-0.151152	1.604345
1	-1.501418	-0.997069	0.933735
1	-0.955530	-1.338915	2.590070
3	-0.547881	0.460394	-0.657509
7	0.776977	1.716540	0.107384
6	1.263993	2.420336	-1.083641
6	0.861700	2.510700	1.334996
1	1.170605	1.700166	-1.916168
6	0.401937	3.632001	-1.456308
6	2.748756	2.808116	-1.011303
1	1.258351	3.514089	1.104578
6	-0.514024	2.730704	1.971458
6	1.830215	1.892195	2.352846
1	0.754080	4.093017	-2.390355
1	-0.645498	3.328427	-1.589599
1	0.436338	4.400813	-0.671152
1	3.092330	3.226612	-1.968285
1	2.923865	3.566000	-0.234373
1	3.359381	1.927005	-0.775919
1	-0.444366	3.364999	2.867863
1	-1.190998	3.212165	1.252925
1	-0.959495	1.770005	2.269976
1	2.833541	1.794352	1.917770
1	1.897091	2.514877	3.256309
1	1.499303	0.888031	2.654464
8	-2.460424	0.528057	-0.898447
6	-3.348453	1.190198	0.023448
6	-4.684083	0.464236	-0.098264
6	-4.238309	-0.955765	-0.457367
6	-3.071926	-0.682065	-1.397338
1	-2.931830	1.096212	1.036948
1	-3.395967	2.251712	-0.245985
1	-5.267571	0.518488	0.826582
1	-5.280194	0.892683	-0.914397
1	-3.891915	-1.487959	0.439286
1	-5.024907	-1.549727	-0.934353

1	-2.296891	-1.457246	-1.405854
1	-3.422261	-0.495910	-2.422787

Calculated Energies for Transition & Ground Structure with Additional THF S4

Ground Structure S4a

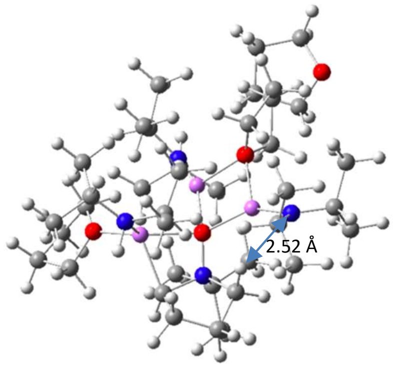

Figure S28. Representative ball and stick model for ground state structure **S4a**.

#m062x/jul-cc-pVDZ opt=(calcfc,noeigen) optcyc=50 freq scrf=(pcm,solvent=THF,read) nosymm

EE = -2039.849200 (Hartree)
ZPE_{298} = -2038.653775
E_{298} = -2038.590974
H_{298} = -2038.590029
G_{298} = -2038.748681
S = 333.910 (cal/mol·K)

7	1.110498	-2.070082	1.260132
6	0.760618	-2.197727	2.751812
6	2.631120	-2.143757	1.170558
6	0.431877	-3.133921	0.372046
1	0.327514	-3.198452	2.859287
1	2.981425	-1.109891	1.131412
1	2.939177	-2.634428	0.245596
6	2.113711	-2.221340	3.464710
6	3.104257	-2.803539	2.451184
1	4.153986	-2.546703	2.646065
1	3.018565	-3.895556	2.400866
1	2.467001	-1.218067	3.763004
1	2.048143	-2.817917	4.384361
6	1.018437	-4.521422	0.645760
6	-1.069421	-3.149466	0.666706
6	0.663031	-2.789040	-1.103034
1	0.938350	-4.803945	1.699561
1	0.441748	-5.245074	0.057573
1	2.065077	-4.600319	0.330824
1	-1.282823	-3.509473	1.677495
1	-1.530744	-2.164009	0.530545

1	-1.543484	-3.839068	-0.043674
1	1.722450	-2.636478	-1.338817
1	0.308786	-3.631546	-1.709749
1	0.099366	-1.907363	-1.422388
7	3.764442	0.526513	-0.622296
6	4.550070	0.508480	-1.836783
6	4.492362	0.965529	0.549347
1	5.635579	0.572250	-1.622055
6	4.330692	-0.807823	-2.594989
6	4.228187	1.677138	-2.787567
1	3.772685	0.939959	1.388925
6	5.651208	0.038900	0.978902
6	5.013416	2.415068	0.482866
1	4.839893	-0.824567	-3.571309
1	4.683707	-1.657974	-1.995584
1	3.250939	-0.944141	-2.771583
1	3.193064	1.575522	-3.147257
1	4.312064	2.638713	-2.263871
1	4.893029	1.694170	-3.666302
1	6.463942	0.045675	0.238531
1	6.079521	0.354149	1.943321
1	5.292959	-0.995032	1.078939
1	5.770669	2.514295	-0.309351
1	4.191031	3.106260	0.249411
1	5.478638	2.726734	1.431282
3	1.947893	0.083843	-0.437521
8	0.700033	-0.797292	0.778931
3	-0.367300	-0.459232	2.550578
7	0.467204	0.975191	3.796328
1	0.781652	0.271834	4.465397
6	1.667944	1.409271	3.051246
6	-0.259402	2.029511	4.538662
6	1.300429	2.386777	1.946051
6	2.789972	1.944252	3.941127
1	2.030519	0.497310	2.558749
6	-1.500882	2.478618	3.773554
6	-0.641822	1.499261	5.915311
1	0.397806	2.902335	4.680581
1	0.498352	1.968201	1.321660
1	0.962624	3.352352	2.347732
1	2.176685	2.569418	1.310634
1	2.507044	2.881153	4.439024
1	3.057453	1.207771	4.711229
1	3.684356	2.144957	3.335801
1	-1.244144	2.869472	2.783130
1	-2.180771	1.625359	3.641993
1	-2.023107	3.269370	4.329094
1	-1.270959	0.603729	5.809361
1	0.250503	1.234110	6.498187
1	-1.209962	2.251286	6.476071

3	-0.767734	-0.084939	-0.369780
8	0.590212	1.129245	-1.457131
7	-2.549044	-0.105683	-1.090607
6	-3.303724	1.034153	-0.605569
6	-3.396762	-1.053599	-1.788030
6	-2.412560	1.878901	0.303573
6	-3.890844	1.960015	-1.689667
1	-4.176454	0.724725	0.017618
6	-2.568711	-2.046756	-2.601612
6	-4.346919	-1.860785	-0.877691
1	-4.055888	-0.538039	-2.517614
1	-2.016830	1.280167	1.138867
1	-1.566325	2.279677	-0.276319
1	-2.952167	2.729796	0.741633
1	-3.081482	2.449262	-2.250723
1	-4.516520	1.412422	-2.405800
1	-4.513749	2.743873	-1.232500
1	-1.968688	-2.678474	-1.935246
1	-3.220072	-2.706242	-3.193452
1	-1.888291	-1.530108	-3.291454
1	-4.966241	-1.204603	-0.250921
1	-5.025793	-2.487522	-1.476167
1	-3.766919	-2.518257	-0.213815
8	-1.327519	1.165379	-6.565829
6	-2.514666	1.922476	-6.776330
6	-3.215817	1.927580	-5.418948
1	-3.141530	1.430593	-7.540752
1	-2.223021	2.914425	-7.138381
6	-2.854724	0.544451	-4.842636
1	-4.296823	2.082108	-5.508200
1	-2.806917	2.729436	-4.790969
6	-1.702367	0.057821	-5.747365
1	-2.558465	0.605685	-3.788509
1	-3.703889	-0.146991	-4.901497
1	-0.809772	-0.261247	-5.194043
1	-2.030167	-0.776323	-6.388381
8	-2.215830	-0.834206	3.044516
6	-2.372897	-1.822269	4.079446
6	-3.873609	-2.060985	4.183579
1	-1.917100	-1.434049	4.997778
1	-1.834977	-2.731424	3.779484
6	-4.305815	-1.883741	2.726774
1	-4.342092	-1.296286	4.817090
1	-4.106901	-3.049262	4.593495
6	-3.437040	-0.713453	2.286511
1	-4.055979	-2.780966	2.143683
1	-5.374267	-1.675741	2.605973
1	-3.180142	-0.725209	1.221120
1	-3.906290	0.250702	2.539374
6	0.278515	0.774940	-2.826187

6	-0.189038	2.075598	-3.450387
6	0.758095	3.095774	-2.814899
6	0.996366	2.530054	-1.413574
1	1.184537	0.383827	-3.312429
1	-0.507010	0.011797	-2.786640
1	-0.150950	2.059549	-4.545198
1	-1.221650	2.262596	-3.132445
1	1.698467	3.150578	-3.374640
1	0.326100	4.100948	-2.771676
1	2.049650	2.554541	-1.107090
1	0.375780	3.024828	-0.659200

Transition Structure S4a

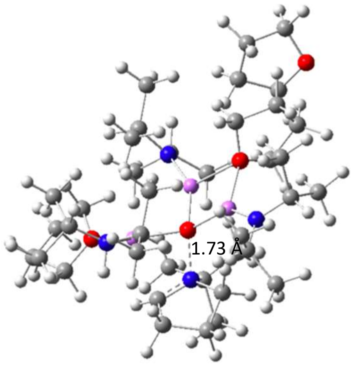

Figure S29. Representative ball and stick model from transition structure **S4a**.

#m062x/jul-cc-pVDZ opt=(calcfc,noeigen,ts) optcyc=50 SCF=XQC freq scrf=(pcm,solvent=THF,read) nosymm

EE = -2039.831494 (Hartree)
ZPE_{298} = -2038.638338
E_{298} = -2038.575127
H_{298} = -2038.574183
G_{298} = -2038.733422
S = 335.147 (cal/mol·K)

7	-1.701068	0.161655	2.138444
6	-2.940384	-0.394624	1.997005
6	-1.912594	1.637584	2.407417
6	-0.718600	-0.531856	3.084553
1	-3.064661	-1.440932	2.232199
1	-1.734474	2.175353	1.478178
1	-1.186745	1.981854	3.145541
6	-4.042269	0.625845	2.073879
6	-3.363805	1.765104	2.848896
1	-3.755532	2.761911	2.611669
1	-3.460804	1.594011	3.928316
1	-4.400930	1.003981	1.098017

1	-4.920916	0.227897	2.597834
6	-1.270665	-0.378954	4.508283
6	-0.610826	-2.014322	2.736997
6	0.671809	0.103800	3.009942
1	-2.289501	-0.773028	4.579338
1	-0.626199	-0.948423	5.188441
1	-1.265832	0.665395	4.843347
1	-1.548961	-2.553958	2.908389
1	-0.282475	-2.158854	1.701710
1	0.145077	-2.455391	3.399557
1	0.636144	1.192065	2.902004
1	1.207561	-0.126386	3.940103
1	1.269834	-0.309488	2.193752
7	-0.687438	3.745083	-0.055836
6	0.264223	4.808871	-0.322952
6	-2.048401	4.234730	0.070701
1	-0.160033	5.803829	-0.081463
6	1.507708	4.657962	0.561228
6	0.682210	4.875179	-1.805055
1	-2.710293	3.350698	0.131826
6	-2.298176	5.038552	1.365915
6	-2.572615	5.057009	-1.124109
1	2.300774	5.378146	0.304046
1	1.235500	4.794602	1.616530
1	1.913114	3.645140	0.446831
1	1.080843	3.898728	-2.117712
1	-0.186757	5.092109	-2.438255
1	1.449811	5.644534	-1.991571
1	-1.697285	5.959712	1.373505
1	-3.356128	5.328004	1.470248
1	-2.010075	4.442689	2.242577
1	-2.029303	6.008524	-1.217147
1	-2.443384	4.500642	-2.062340
1	-3.640559	5.295708	-1.002261
3	-0.118300	1.915152	0.112348
8	-0.803834	0.209560	0.654976
3	-2.130014	-0.945533	-0.045092
7	-3.677108	-0.334863	-1.301266
1	-4.396862	-0.354804	-0.578271
6	-3.296172	1.085433	-1.479969
6	-4.226768	-0.994117	-2.511123
6	-2.159877	1.218685	-2.485534
6	-4.482145	1.985822	-1.821888
1	-2.891737	1.394680	-0.505809
6	-3.325845	-2.140979	-2.955876
6	-5.655735	-1.465835	-2.264091
1	-4.259210	-0.256636	-3.325338
1	-1.320588	0.567935	-2.203508
1	-2.478602	0.955581	-3.503683
1	-1.802628	2.255520	-2.488851

1	-4.922426	1.721781	-2.793361
1	-5.265699	1.903126	-1.055509
1	-4.161452	3.033057	-1.870144
1	-2.295651	-1.789191	-3.093839
1	-3.316437	-2.934957	-2.197193
1	-3.681621	-2.562970	-3.905438
1	-5.688005	-2.180628	-1.428658
1	-6.310937	-0.618827	-2.020366
1	-6.056803	-1.969105	-3.152658
3	0.835009	-0.585192	0.168235
7	1.843332	-2.159066	-0.242447
6	1.606801	-2.592251	-1.605346
6	2.653203	-3.103783	0.498649
6	0.530663	-1.710929	-2.242619
6	2.844264	-2.593133	-2.525900
1	1.210678	-3.634523	-1.651109
6	3.135548	-2.495548	1.814284
6	1.954032	-4.439626	0.834432
1	3.570023	-3.383286	-0.065514
1	-0.400258	-1.747776	-1.658651
1	0.866667	-0.664352	-2.288876
1	0.295430	-2.025796	-3.269035
1	3.222936	-1.570022	-2.664915
1	3.658803	-3.198363	-2.107898
1	2.593272	-3.005258	-3.515330
1	2.277267	-2.266105	2.460699
1	3.788447	-3.195125	2.356103
1	3.695198	-1.567946	1.642896
1	1.549830	-4.930741	-0.061140
1	2.658596	-5.140007	1.308374
1	1.122174	-4.263190	1.533370
8	1.837063	1.240222	-0.275031
6	2.941284	1.310586	0.654068
6	4.046129	2.136393	-0.030833
1	3.269685	0.283664	0.861377
1	2.561915	1.767026	1.573609
6	3.509015	2.388575	-1.451999
1	4.989108	1.580240	-0.053805
1	4.223243	3.077930	0.499219
6	2.410633	1.344071	-1.590333
1	3.089351	3.395719	-1.535741
1	4.278805	2.272163	-2.222532
1	1.603585	1.615324	-2.280774
1	2.823495	0.361681	-1.865394
8	-2.233602	-2.891788	0.061710
6	-3.400677	-3.576490	0.546515
6	-3.162969	-5.058088	0.264266
1	-4.285166	-3.166137	0.043355
1	-3.492513	-3.392778	1.626547
6	-1.635639	-5.144205	0.327746

1	-3.513497	-5.318629	-0.743006
1	-3.672884	-5.700596	0.989283
6	-1.241622	-3.848578	-0.359438
1	-1.290055	-5.141031	1.370909
1	-1.226573	-6.025888	-0.176678
1	-0.260799	-3.449260	-0.080347
1	-1.294471	-3.953183	-1.455443
8	7.074325	0.295292	-0.016453
6	7.289041	-0.071227	-1.375958
6	5.933250	-0.578476	-1.854753
6	5.397433	-1.304046	-0.609343
6	6.202546	-0.685712	0.553550
1	7.656199	0.813436	-1.908048
1	8.049623	-0.869270	-1.437659
1	5.290821	0.271311	-2.121414
1	6.010685	-1.236111	-2.727614
1	4.311297	-1.188860	-0.493651
1	5.601276	-2.379551	-0.676775
1	5.576050	-0.172776	1.296163
1	6.798410	-1.449668	1.076132

Transition Structure S4b

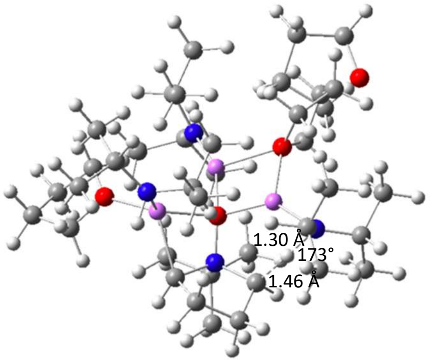

Figure S30. Representative ball and stick model from transition structure **S4b**.

#m062x/jul-cc-pVDZ opt=(calcfc,noeigen,ts) optcyc=50 freq scrf=(pcm,solvent=THF,read) nosymm

EE = -2039.822402 (Hartree)
ZPE_{298} = -2038.632456
E_{298} = -2038.569725
H_{298} = -2038.568781
G_{298} = -2038.728005
S = 335.115 (cal/mol·K)

7	0.200766	0.421977	0.619141
6	0.097821	0.280379	2.150033

6	1.690707	0.426504	0.246123
6	-0.591016	-0.651495	-0.155987
1	-0.232931	-0.752667	2.319061
1	2.371581	1.655362	-0.161987
1	1.804847	-0.205446	-0.640093
6	1.553232	0.354839	2.617331
6	2.379767	-0.194989	1.452694
1	3.441575	0.090782	1.493860
1	2.336713	-1.298664	1.462648
1	1.887093	1.385790	2.834246
1	1.682739	-0.221436	3.545383
6	0.034986	-2.036083	0.042695
6	-2.043473	-0.684544	0.326579
6	-0.568774	-0.320060	-1.653329
1	0.096547	-2.314547	1.098584
1	-0.607598	-2.767254	-0.463081
1	1.034360	-2.103990	-0.399365
1	-2.112798	-0.978304	1.378754
1	-2.544825	0.279792	0.189423
1	-2.583825	-1.429043	-0.274214
1	0.443940	-0.134586	-2.025212
1	-0.974002	-1.179463	-2.202492
1	-1.201460	0.535621	-1.905658
7	2.864136	2.760864	-0.642529
6	3.663857	2.618476	-1.855165
6	3.503628	3.496225	0.442243
1	4.730155	2.811047	-1.641338
6	3.569220	1.190353	-2.400089
6	3.233771	3.602353	-2.950453
1	2.777466	3.471953	1.268381
6	4.786353	2.846941	0.987061
6	3.781188	4.968234	0.117253
1	4.152604	1.084892	-3.324802
1	3.935940	0.464226	-1.662883
1	2.521630	0.939603	-2.629941
1	2.197669	3.381379	-3.247931
1	3.271291	4.639124	-2.592373
1	3.871635	3.518350	-3.843110
1	5.612832	2.908710	0.265691
1	5.112334	3.357518	1.904940
1	4.610812	1.790882	1.223698
1	4.545568	5.052643	-0.668966
1	2.873803	5.476014	-0.236112
1	4.156946	5.501816	1.002618
3	0.894645	2.615293	-0.723628
8	-0.384169	1.679743	0.261408
3	-1.205907	1.912493	2.060798
7	-0.545080	3.361821	3.412391
1	-0.163464	2.612895	3.991405
6	0.589864	3.927701	2.656331

6	-1.274640	4.315902	4.278037
6	0.106681	4.945033	1.631728
6	1.698438	4.487207	3.549489
1	0.993996	3.074830	2.095087
6	-2.706279	4.507622	3.788098
6	-1.239513	3.849207	5.729253
1	-0.770188	5.292293	4.229822
1	-0.655178	4.498045	0.978388
1	-0.326248	5.836854	2.106228
1	0.952450	5.272029	1.012226
1	1.337228	5.319131	4.169121
1	2.086218	3.704310	4.215498
1	2.531782	4.864097	2.941806
1	-2.715285	4.809695	2.733385
1	-3.268088	3.568358	3.876268
1	-3.211729	5.284067	4.378378
1	-1.688826	2.849215	5.819375
1	-0.206859	3.799068	6.099588
1	-1.806377	4.534003	6.372311
3	-1.798765	2.312347	-0.842026
8	-0.309090	3.548463	-1.978645
7	-3.577527	2.359180	-1.529119
6	-4.248639	3.542598	-1.027672
6	-4.494620	1.465458	-2.208560
6	-3.294841	4.308306	-0.111165
6	-4.759411	4.516511	-2.107784
1	-5.138942	3.288841	-0.404785
6	-3.743696	0.407716	-3.015472
6	-5.481128	0.726264	-1.279741
1	-5.126796	2.018017	-2.935950
1	-2.962750	3.677597	0.728041
1	-2.411963	4.628121	-0.687101
1	-3.757229	5.208954	0.315437
1	-3.907974	4.942731	-2.659945
1	-5.416461	4.018317	-2.832057
1	-5.325639	5.344331	-1.654366
1	-3.239124	-0.295096	-2.339684
1	-4.436968	-0.169005	-3.644903
1	-2.985620	0.864726	-3.666325
1	-6.052073	1.421026	-0.648673
1	-6.203796	0.137484	-1.865372
1	-4.929716	0.038929	-0.621028
8	-2.292602	3.463513	-7.034508
6	-3.454832	4.250589	-7.272353
6	-4.147140	4.338820	-5.913387
1	-4.102609	3.746821	-8.011114
1	-3.132337	5.215199	-7.679303
6	-3.828099	2.970875	-5.278088
1	-5.223059	4.524169	-6.004906
1	-3.709559	5.154275	-5.323509

6	-2.701730	2.404739	-6.169171
1	-3.522897	3.067489	-4.228937
1	-4.700624	2.307185	-5.299970
1	-1.817165	2.073256	-5.610339
1	-3.066522	1.558773	-6.773698
8	-3.050437	1.527378	2.500973
6	-3.169724	0.676224	3.655226
6	-4.657402	0.348612	3.782031
1	-2.767591	1.210000	4.526507
1	-2.556308	-0.217174	3.479451
6	-5.137240	0.479274	2.334185
1	-5.161736	1.091560	4.413607
1	-4.823582	-0.644412	4.212317
6	-4.326993	1.674363	1.859297
1	-4.863254	-0.413260	1.754584
1	-6.216825	0.640473	2.243933
1	-4.155372	1.709876	0.777590
1	-4.786460	2.621496	2.189916
6	-0.695349	3.152743	-3.320094
6	-1.012929	4.457344	-4.028866
6	0.027699	5.405503	-3.430679
6	0.088171	4.946961	-1.977406
1	0.144036	2.619224	-3.789153
1	-1.567390	2.495060	-3.222804
1	-0.959624	4.368659	-5.119218
1	-2.024707	4.780066	-3.750983
1	0.999355	5.269092	-3.920310
1	-0.256548	6.459756	-3.508656
1	1.092908	5.009312	-1.540781
1	-0.618295	5.498536	-1.344832

Ground State Structure S4'a

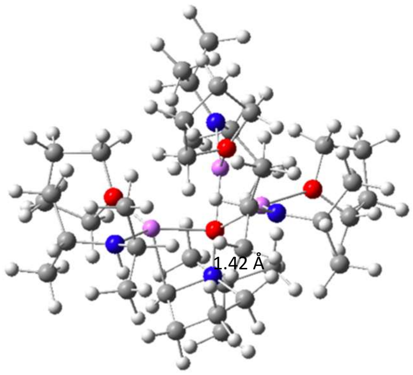

Figure S31. Representative ball and stick model for ground state structure **S4'a**.

#m062x/jul-cc-pVDZ opt=(calcfc,noeigen) optcyc=50 freq scrf=(pcm,solvent=THF,read) nosymm

EE = -2039.848845 (Hartree)
ZPE_{298} = -2038.651405
E_{298} = -2038.589063
H_{298} = -2038.588119
G_{298} = -2038.743561
S = 327.157 (cal/mol·K)

7	0.652523	-2.309937	0.199064
6	0.981255	-2.377621	1.699679
6	1.969041	-2.433050	-0.527789
6	-0.339695	-3.401281	-0.257194
1	0.385977	-3.193853	2.118037
1	1.932948	-1.829087	-1.436580
1	2.120724	-3.480522	-0.799378
6	2.473801	-2.714758	1.743002
6	3.013503	-2.024454	0.491743
1	3.019180	-0.932592	0.596931
1	4.013977	-2.353304	0.186859
1	2.959327	-2.351143	2.661257
1	2.703723	-3.796484	1.676076
6	0.182881	-4.807023	0.065547
6	-1.653152	-3.198790	0.497813
6	-0.521031	-3.290667	-1.767638
1	0.440349	-4.927463	1.121611
1	-0.627828	-5.509254	-0.166592
1	1.042221	-5.096303	-0.547882
1	-1.552110	-3.519920	1.539048
1	-1.986962	-2.156298	0.483407
1	-2.426528	-3.821669	0.032440
1	0.380599	-3.635848	-2.287986
1	-1.348127	-3.940545	-2.078330
1	-0.743039	-2.265008	-2.071679
7	2.764591	1.122932	-1.731579
6	3.468706	1.007485	-2.998823
6	3.427615	2.048908	-0.836356
1	4.435953	1.545147	-2.970129
6	3.831313	-0.448642	-3.321211
6	2.680145	1.638279	-4.161227
1	2.807891	2.124354	0.068891
6	4.814686	1.584199	-0.340593
6	3.546720	3.495647	-1.366410
1	4.331320	-0.550225	-4.297832
1	4.496492	-0.841851	-2.540350
1	2.929510	-1.074949	-3.335779
1	1.674732	1.198785	-4.208270
1	2.562758	2.716592	-3.986427
1	3.176391	1.495732	-5.135426
1	5.530118	1.516449	-1.173066
1	5.236337	2.280090	0.403036

1	4.736392	0.588485	0.117004
1	4.214835	3.547581	-2.238247
1	2.560158	3.868197	-1.677374
1	3.950928	4.172939	-0.596958
3	1.052099	0.239619	-1.506347
8	0.128952	-1.023329	-0.109491
3	0.170837	-0.445718	1.817991
7	1.742040	0.407757	3.169551
1	1.890620	-0.595530	3.048238
6	2.967408	1.070178	2.675815
6	1.419750	0.646315	4.592282
6	2.906080	2.581790	2.848331
6	4.250141	0.495810	3.279787
1	2.995486	0.859437	1.599114
6	0.288551	1.661699	4.730324
6	1.044263	-0.679889	5.244140
1	2.305876	1.041787	5.114436
1	1.973080	2.989351	2.444427
1	2.977226	2.867307	3.906997
1	3.746427	3.046252	2.316292
1	4.285126	0.640085	4.368382
1	4.331906	-0.578675	3.067237
1	5.126609	0.994130	2.844491
1	0.521579	2.593832	4.202442
1	-0.632085	1.242008	4.305370
1	0.111219	1.898532	5.788414
1	0.233602	-1.156209	4.675048
1	1.902968	-1.364949	5.255515
1	0.705682	-0.528230	6.276719
3	-1.620791	-0.050753	-0.724395
8	-0.337637	1.588508	-0.837110
7	-3.525454	0.254687	-0.995222
6	-4.292681	1.356672	-0.444801
6	-4.395264	-0.783845	-1.516536
6	-3.371756	2.537008	-0.160232
6	-5.440711	1.874351	-1.333108
1	-4.782344	1.090461	0.525894
6	-3.582612	-1.833934	-2.262086
6	-5.248227	-1.481620	-0.441333
1	-5.110207	-0.372227	-2.260285
1	-2.475234	2.218181	0.383279
1	-3.050667	2.977554	-1.114631
1	-3.878548	3.315899	0.428546
1	-5.064503	2.077641	-2.348235
1	-6.270285	1.160511	-1.410752
1	-5.844566	2.809248	-0.917662
1	-2.958284	-2.392358	-1.558862
1	-4.232260	-2.552770	-2.782352
1	-2.928046	-1.354997	-3.002290
1	-5.901103	-0.767845	0.079506

1	-5.886754	-2.264890	-0.878614
1	-4.585653	-1.946072	0.306258
8	0.005351	-0.323286	-3.252066
6	0.479454	-1.272560	-4.216288
6	-0.769072	-1.695614	-4.971480
1	0.981013	-2.080538	-3.676403
1	1.204903	-0.788733	-4.888831
6	-1.514896	-0.360135	-5.104498
1	-1.344520	-2.411369	-4.370964
1	-0.536869	-2.152246	-5.939195
6	-1.020397	0.461065	-3.897993
1	-1.240179	0.138516	-6.040802
1	-2.603143	-0.483056	-5.093631
1	-0.586361	1.417799	-4.218169
1	-1.808238	0.644389	-3.155511
8	-1.625006	-0.350252	2.630285
6	-2.154483	-1.472287	3.346247
6	-2.770893	-0.853320	4.592764
1	-1.330587	-2.170625	3.524647
1	-2.923908	-1.963676	2.729725
6	-3.362798	0.461932	4.049889
1	-1.989363	-0.652379	5.335514
1	-3.525070	-1.503203	5.048267
6	-2.630629	0.674996	2.709416
1	-4.441535	0.373672	3.882023
1	-3.196481	1.296536	4.739186
1	-3.302626	0.566170	1.848606
1	-2.124400	1.645047	2.651265
6	-0.328284	2.738000	-1.718421
6	-0.337257	3.960601	-0.812791
6	0.406444	3.480297	0.437414
6	0.168787	1.966935	0.461954
1	0.587023	2.677181	-2.324606
1	-1.216608	2.669216	-2.351593
1	0.163643	4.811428	-1.287376
1	-1.362504	4.254771	-0.568647
1	1.476180	3.692377	0.350130
1	0.033887	3.956239	1.351036
1	1.090711	1.404964	0.652454
1	-0.587878	1.684867	1.207232

Transition Structure S4'a

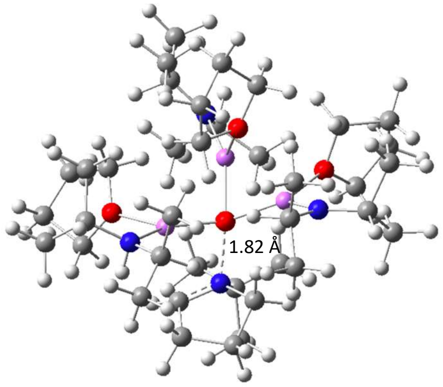

Figure S32. Representative ball and stick model from transition structure **S4'a**.

#m062x/jul-cc-pVDZ opt=(calcfc,noeigen,ts) optcyc=50 freq scrf=(pcm,solvent=THF,read) nosymm

EE = -2039.819532 (Hartree)
ZPE_{298} = -2038.624596
E_{298} = -2038.561866
H_{298} = -2038.560922
G_{298} = -2038.716883
S = 328.248 (cal/mol·K)

7	-0.161316	0.003898	0.208672
6	-0.015180	-0.116940	1.554270
6	1.220360	0.172850	-0.365428
6	-1.062708	-1.016690	-0.478487
1	-0.827145	-0.575607	2.103742
1	1.388306	1.247005	-0.453599
1	1.263106	-0.256936	-1.367023
6	1.426713	-0.250484	1.981059
6	2.155704	-0.477090	0.645772
1	3.151640	-0.019612	0.610971
1	2.263835	-1.550103	0.449221
1	1.850839	0.617577	2.502865
1	1.547079	-1.105474	2.661065
6	-0.418646	-2.396915	-0.301313
6	-2.432246	-0.986232	0.198380
6	-1.200930	-0.728573	-1.962740
1	-0.261064	-2.636460	0.755716
1	-1.095337	-3.145422	-0.732606
1	0.537890	-2.465881	-0.834526
1	-2.417884	-1.518886	1.156056
1	-2.753953	0.050120	0.355455
1	-3.159441	-1.493604	-0.446454
1	-0.247988	-0.803713	-2.499779
1	-1.878035	-1.476438	-2.395587

1	-1.616947	0.262727	-2.113699
7	1.567546	3.051687	-2.434000
6	2.264335	3.088358	-3.700924
6	2.317474	3.629414	-1.337023
1	3.365587	3.109761	-3.559995
6	1.947077	1.818392	-4.496170
6	1.928423	4.306455	-4.590758
1	1.671261	3.533927	-0.444798
6	3.625784	2.889304	-0.970866
6	2.646798	5.130890	-1.472807
1	2.394254	1.826739	-5.502283
1	2.302292	0.931311	-3.954357
1	0.855735	1.739204	-4.599761
1	0.867914	4.272536	-4.880308
1	2.100967	5.249413	-4.060309
1	2.532756	4.313204	-5.513067
1	4.407942	3.056470	-1.724787
1	4.023675	3.240996	-0.005159
1	3.450047	1.807653	-0.902573
1	3.388425	5.288651	-2.270021
1	1.743017	5.697916	-1.735558
1	3.067708	5.542321	-0.542505
3	-0.141594	2.328162	-1.949644
8	-0.892665	1.582676	-0.326189
3	-0.853645	2.007155	1.501826
7	0.533713	2.911792	3.003654
1	0.565492	1.947484	3.337347
6	1.907407	3.213483	2.522408
6	0.092583	3.745605	4.150437
6	2.073023	4.673431	2.116731
6	2.994441	2.832472	3.532882
1	2.042249	2.598720	1.617553
6	-0.881323	4.820917	3.687568
6	-0.543458	2.877976	5.228451
1	0.968915	4.239214	4.597587
1	1.349273	4.977594	1.355072
1	1.976282	5.342174	2.982078
1	3.078807	4.813661	1.701838
1	2.975617	3.497125	4.407210
1	2.877157	1.799833	3.886348
1	3.984700	2.922727	3.067467
1	-0.438680	5.439868	2.900941
1	-1.791455	4.347376	3.294292
1	-1.160306	5.473313	4.525653
1	-1.459936	2.415130	4.842719
1	0.145761	2.090046	5.562704
1	-0.808989	3.490907	6.098431
3	-2.626325	2.464423	-1.008390
8	-1.554050	4.243249	-1.155779
7	-4.601294	2.619388	-1.136588

6	-5.414330	3.550101	-0.381354
6	-5.383397	1.501406	-1.627065
6	-4.618426	4.819936	-0.104745
6	-6.743979	3.961189	-1.040750
1	-5.708253	3.142764	0.621934
6	-4.547090	0.663903	-2.585267
6	-5.953801	0.600559	-0.515081
1	-6.257822	1.846594	-2.219747
1	-3.639347	4.580208	0.324412
1	-4.450545	5.356494	-1.049899
1	-5.149372	5.489039	0.588598
1	-6.560416	4.301999	-2.071616
1	-7.468505	3.137804	-1.075602
1	-7.204751	4.785949	-0.477424
1	-3.682008	0.237367	-2.060255
1	-5.127981	-0.167286	-3.012109
1	-4.178749	1.296765	-3.403833
1	-6.594791	1.173065	0.170183
1	-6.556637	-0.221445	-0.931932
1	-5.130022	0.166697	0.072479
8	-1.510698	1.938589	-3.613117
6	-1.718126	0.877716	-4.571700
6	-2.267819	1.542683	-5.829808
1	-2.411583	0.146086	-4.146530
1	-0.753208	0.387010	-4.759229
6	-1.657018	2.941644	-5.749658
1	-3.362996	1.603559	-5.780123
1	-1.989718	0.995044	-6.736405
6	-1.724226	3.209941	-4.253628
1	-0.612951	2.926564	-6.088920
1	-2.205239	3.691969	-6.329086
1	-0.942436	3.885333	-3.893024
1	-2.715720	3.587700	-3.961570
8	-2.477795	1.732871	2.597475
6	-2.765293	0.639620	3.478639
6	-4.037900	1.057206	4.196127
1	-1.895428	0.487109	4.127517
1	-2.938026	-0.275108	2.891786
6	-4.820284	1.697623	3.045371
1	-3.808878	1.798020	4.973954
1	-4.557771	0.209788	4.654951
6	-3.721609	2.371873	2.217732
1	-5.320266	0.922431	2.450547
1	-5.576306	2.415213	3.379911
1	-3.871646	2.251793	1.135550
1	-3.628546	3.439596	2.460442
6	-1.737155	5.452563	-1.917793
6	-1.732212	6.590387	-0.910159
6	-0.738241	6.075447	0.131673
6	-1.001280	4.569118	0.135521

1	-0.892637	5.556588	-2.615718
1	-2.671885	5.356336	-2.480111
1	-1.419213	7.535747	-1.365694
1	-2.724834	6.723702	-0.466232
1	0.291288	6.278461	-0.186996
1	-0.890300	6.525167	1.117606
1	-0.100636	3.966872	0.278180
1	-1.740155	4.294347	0.905434

Transition Structure S4'b

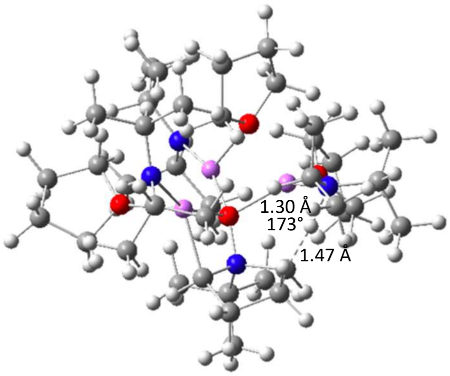

Figure S33. Representative ball and stick model from transition structure **S4b**.

#m062x/jul-cc-pVDZ opt=(calcfc,noeigen,ts) optcyc=50 freq SCF=XQC scrf=(pcm,solvent=THF,read) nosymm

EE = -2039.819943 (Hartree)
ZPE_{298} = -2038.626374
E_{298} = -2038.564948
H_{298} = -2038.564004
G_{298} = -2038.717232
S = 322.495 (cal/mol·K)

7	-0.088094	0.107941	0.014804
8	0.026067	0.132821	1.443961
6	1.349653	0.090033	-0.559293
6	-0.921783	-1.113828	-0.385675
6	-0.884919	1.336338	-0.430654
6	-2.065395	-0.567401	-1.253418
6	-1.695039	0.870250	-1.628144
1	-1.660682	-1.828032	0.669557
1	-0.274959	-1.760125	-0.987040
1	-3.003166	-0.546769	-0.684735
1	-2.240396	-1.204960	-2.133110
1	-2.598661	1.488322	-1.763138
1	-1.163712	0.884937	-2.597363
1	-0.147304	2.095687	-0.722738

6	2.064084	-1.194206	-0.141097
6	1.322751	0.148694	-2.088062
6	2.116920	1.319045	-0.055863
1	1.699426	-2.052235	-0.716338
1	3.136162	-1.090116	-0.351546
1	1.942409	-1.396068	0.924748
1	0.724429	-0.659036	-2.522947
1	0.945439	1.109702	-2.449448
1	2.356702	0.033146	-2.439736
1	1.794734	2.223680	-0.579743
1	1.992048	1.476619	1.019790
1	3.182939	1.178832	-0.274198
3	-1.152903	1.720799	1.568518
8	0.103038	3.249043	2.051946
6	0.377919	4.290133	1.101565
6	0.511528	5.551813	1.940188
6	1.249675	5.010715	3.167514
6	0.627780	3.623869	3.342930
1	1.318784	4.064909	0.578142
1	-0.440152	4.303994	0.374111
1	1.058006	6.345237	1.419837
1	-0.481529	5.932182	2.218121
1	2.321984	4.919268	2.952267
1	1.128416	5.631140	4.061204
1	1.353485	2.864098	3.658128
1	-0.214117	3.646486	4.053255
7	-2.843137	2.955617	2.298019
1	-2.125151	3.671622	2.183350
6	-3.308228	3.085308	3.698074
6	-3.882463	3.213364	1.268256
1	-2.469065	2.747422	4.325045
6	-3.603421	4.534431	4.091007
6	-4.497200	2.177470	3.986975
1	-4.705165	3.795131	1.714523
6	-3.278912	4.041133	0.138915
6	-4.444590	1.906220	0.717477
1	-2.717483	5.165001	3.931670
1	-4.435041	4.949767	3.506466
1	-3.875358	4.593438	5.153017
1	-4.286463	1.139549	3.699947
1	-4.726283	2.205602	5.059797
1	-5.393254	2.510472	3.447257
1	-2.913380	5.009324	0.511559
1	-2.445109	3.483909	-0.312186
1	-4.032020	4.235804	-0.634673
1	-3.635035	1.347331	0.228942
1	-4.875953	1.280819	1.507397
1	-5.231421	2.116893	-0.020068
7	-2.358414	-2.304600	1.658334
6	-2.567304	-3.753631	1.649637

6	-3.604122	-1.546412	1.752797
1	-3.647613	-3.977010	1.642354
6	-1.988627	-4.392091	0.382271
6	-2.002405	-4.418552	2.910316
1	-3.320335	-0.493245	1.590088
6	-4.668783	-1.900497	0.689658
6	-4.280273	-1.642458	3.127189
1	-2.114463	-5.484071	0.392975
1	-2.489179	-3.989555	-0.508357
1	-0.918854	-4.169890	0.288040
1	-0.923666	-4.226059	2.992005
1	-2.488085	-4.000003	3.802746
1	-2.164963	-5.507046	2.903165
1	-5.283990	-2.753337	1.009541
1	-5.349231	-1.050643	0.534405
1	-4.207687	-2.154167	-0.271052
1	-4.533853	-2.688849	3.351306
1	-3.629689	-1.273442	3.927614
1	-5.217129	-1.064539	3.144858
3	-0.535370	-1.560944	2.160670
3	1.246073	0.189274	3.037552
7	2.722612	0.654741	4.192857
6	2.661921	1.425890	5.418937
6	4.087537	0.496664	3.724999
1	2.893306	2.508759	5.246832
1	4.715519	-0.024902	4.481597
8	-0.407860	-0.686280	3.985000
8	1.075125	-2.877661	2.584502
6	-0.503231	-1.609372	5.092643
6	1.904130	-3.082000	3.746665
6	-1.719436	-1.193028	5.915798
6	3.148754	-3.858419	3.271728
6	1.251144	1.385374	5.996936
6	3.632926	0.974955	6.525936
6	4.114868	-0.378097	2.476905
6	4.807621	1.820541	3.408669
1	1.017690	0.370121	6.348225
1	1.144645	2.076673	6.845798
1	0.512866	1.662431	5.237776
1	3.502783	-0.102104	6.716436
1	4.683158	1.151275	6.262287
1	3.427055	1.521272	7.458136
1	3.515602	-1.284610	2.630205
1	3.694581	0.166609	1.624499
1	5.139688	-0.679083	2.214550
1	4.243387	2.369116	2.637280
1	4.887548	2.464683	4.294588
1	5.825974	1.638459	3.032102
6	-1.961512	0.266756	5.510805
6	-1.421152	0.334457	4.085327

1	-0.608380	-2.617095	4.677736
1	0.430712	-1.545876	5.661247
1	-2.582212	-1.818486	5.662809
1	-1.529298	-1.296652	6.988871
1	-3.021182	0.537108	5.552932
1	-1.405082	0.947976	6.163023
1	-2.194079	0.137060	3.331336
1	-0.943267	1.297632	3.859230
6	2.924336	-4.036406	1.761109
6	1.413541	-3.905693	1.646232
1	2.136705	-2.091419	4.158443
1	1.340353	-3.665740	4.487751
1	4.073927	-3.315083	3.491070
1	3.201783	-4.831182	3.772841
1	3.409400	-3.231175	1.195774
1	3.289245	-4.998393	1.386600
1	1.056653	-3.586770	0.663365
1	0.908449	-4.844593	1.927402

LDA – THF – Trimethyl N-oxide Dimer Structures S5-S7

Ground State Structure S5

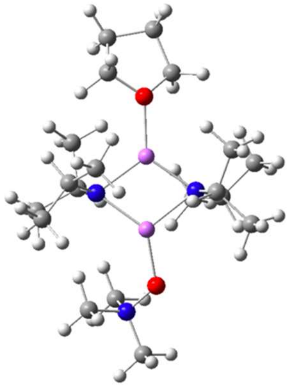

Figure S34. Representative ball and stick model from ground state **S5**.

#m062x/jul-cc-pvDz opt=(calcfc,noeigen,z-matrix) optcyc=50 freq SCF=XQC scrf=(pcm,solvent=THF,read) nosymm

EE = -1080.518444 (Hartree)
ZPE_{298} = -1079.880637
E_{298} = -1079.845630
H_{298} = -1079.844686
G_{298} = -1079.946115
S = 213.476 (cal/mol·K)

3	0.000000	0.000000	0.000000
7	0.000000	0.000000	1.943908
3	1.935830	0.000000	1.360453

7	1.902587	0.113201	-0.647645
8	-1.554596	-0.078415	-1.163275
6	-2.674405	0.780374	-0.868034
6	-3.492633	0.854217	-2.154613
6	-2.421888	0.640326	-3.228014
6	-1.535586	-0.403504	-2.565232
1	-2.284317	1.767854	-0.579425
1	-3.221003	0.351976	-0.019566
1	-4.022278	1.807489	-2.250534
1	-4.228597	0.040700	-2.186271
1	-1.854273	1.565023	-3.397874
1	-2.828913	0.294563	-4.183879
1	-0.492503	-0.385566	-2.900308
1	-1.942425	-1.417200	-2.698280
6	-0.386429	-1.281276	2.525229
6	-0.295262	1.181784	2.727272
1	0.286387	-2.037788	2.072292
6	-0.174158	-1.405124	4.041521
6	-1.806428	-1.757792	2.150642
1	0.183769	1.141080	3.732159
6	0.319909	2.385075	2.008985
6	-1.785516	1.475151	2.984914
1	-0.876119	-0.772303	4.603083
1	-0.328685	-2.443865	4.366585
1	0.846805	-1.104738	4.315439
1	-1.931423	-1.722500	1.058906
1	-1.977478	-2.793767	2.485411
1	-2.581444	-1.123625	2.601298
1	-0.103608	2.472791	0.994523
1	0.121900	3.324302	2.544695
1	1.408617	2.265692	1.917892
1	-2.329345	1.533527	2.029610
1	-2.253458	0.694407	3.595987
1	-1.907934	2.432546	3.515495
8	3.258623	0.336157	2.653386
7	4.511451	0.808612	2.345143
6	5.134523	-0.063236	1.312817
6	4.396836	2.195159	1.817102
6	5.339054	0.806878	3.580019
1	5.211831	-1.067603	1.738046
1	4.456379	-0.064937	0.454072
1	6.124201	0.325913	1.045738
1	3.782996	2.144879	0.913150
1	3.898943	2.793888	2.583889
1	5.393345	2.592126	1.593075
1	4.840766	1.453551	4.306703
1	5.368696	-0.221569	3.948412
1	6.346246	1.172187	3.351873
6	2.144691	1.427776	-1.235317
6	2.108273	-1.013349	-1.546372

1	2.087872	2.146183	-0.396335
6	3.513693	1.649729	-1.904626
6	1.055046	1.887433	-2.227432
1	1.835494	-0.755300	-2.593510
6	3.545545	-1.575007	-1.592130
6	1.199972	-2.171911	-1.111619
1	4.342544	1.378941	-1.236659
1	3.604429	1.046022	-2.819305
1	3.633723	2.705111	-2.190188
1	0.063232	1.856095	-1.754597
1	1.237377	2.917697	-2.568838
1	1.034161	1.243462	-3.119203
1	4.289101	-0.804360	-1.821213
1	3.796526	-2.013184	-0.613627
1	3.633051	-2.369576	-2.349618
1	1.402682	-2.408037	-0.054804
1	0.132522	-1.928835	-1.207632
1	1.385349	-3.081799	-1.699667

Ground State Structure S6

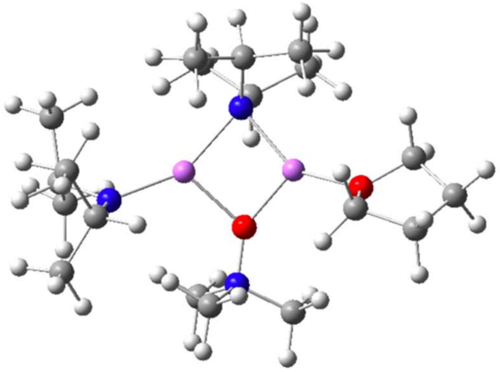

Figure S35. Representative ball and stick model ground state structure **S6**.

#m062x/jul-cc-pvDz opt=(calcfc,noeigen) optcyc=50 freq SCF=XQC scrf=(pcm,solvent=THF,read) nosymm

EE = -1080.506458 (Hartree)
ZPE298 = 1079.869285
E298 = 1079.833803
H298 = 1079.832859
G298 = 1079.937331
S = 219.880 (cal/mol·K)

3	-0.222981	-0.227806	0.325169
7	-0.126761	-0.610539	2.242606
3	1.851192	-0.330560	1.866084
8	1.531004	-0.659700	-0.032680
8	-1.261970	0.761724	-0.971387

6	-0.913785	2.116704	-1.277908
6	-1.746258	2.468700	-2.517125
6	-2.928078	1.470128	-2.463646
6	-2.677223	0.679253	-1.176085
1	0.169371	2.158654	-1.432418
1	-1.181838	2.756449	-0.422476
1	-1.159026	2.328618	-3.430764
1	-2.078550	3.511138	-2.484500
1	-2.912466	0.803830	-3.332641
1	-3.900485	1.972042	-2.437869
1	-2.947155	-0.379867	-1.234133
1	-3.188102	1.140424	-0.316776
6	-0.417108	-2.040307	2.324327
6	-0.917403	0.208759	3.149518
1	0.184046	-2.515037	1.525022
6	0.038647	-2.697937	3.639881
6	-1.883058	-2.425512	2.045696
1	-1.352086	-0.397890	3.968372
6	-0.038825	1.275068	3.809957
6	-2.097977	0.907592	2.447669
1	1.119508	-2.561475	3.773038
1	-0.470106	-2.247514	4.504559
1	-0.178669	-3.777023	3.648873
1	-2.550432	-2.022579	2.821792
1	-2.211543	-2.023681	1.075300
1	-2.011079	-3.518101	2.028358
1	0.475648	1.860456	3.029605
1	-0.625791	1.971015	4.428570
1	0.731525	0.806195	4.436015
1	-2.746842	0.175131	1.949987
1	-2.710877	1.494012	3.150531
1	-1.711296	1.596893	1.678317
7	3.640370	0.209659	2.410958
6	4.070695	1.590728	2.297469
6	4.319557	-0.532289	3.454993
1	3.402728	2.066555	1.551385
6	3.938420	2.456562	3.568526
6	5.508085	1.758551	1.751184
1	5.313852	-0.096355	3.689980
6	3.546965	-0.586738	4.791088
6	4.577415	-1.970857	2.990693
1	2.914225	2.412211	3.963391
1	4.623734	2.103520	4.353562
1	4.187290	3.508262	3.357517
1	5.624420	1.221274	0.799572
1	5.760662	2.818048	1.587833
1	6.243699	1.348670	2.458580
1	3.345716	0.421485	5.171766
1	2.575940	-1.081201	4.632715
1	4.100037	-1.146946	5.563442

1	3.626311	-2.428866	2.670929
1	5.262858	-1.979500	2.131719
1	5.004804	-2.597381	3.788642
7	2.497499	-0.716323	-1.003258
6	3.425841	-1.833122	-0.669825
6	1.852633	-0.944779	-2.321397
6	3.259436	0.563246	-1.005438
1	3.852105	-1.590176	0.309445
1	2.828486	-2.747502	-0.622686
1	4.198941	-1.907546	-1.442142
1	1.148845	-0.123857	-2.487665
1	2.618326	-0.971455	-3.103715
1	1.317756	-1.896484	-2.265605
1	3.664069	0.668504	0.005914
1	4.051728	0.512862	-1.760443
1	2.553182	1.365006	-1.236539

Ground State Structure S7

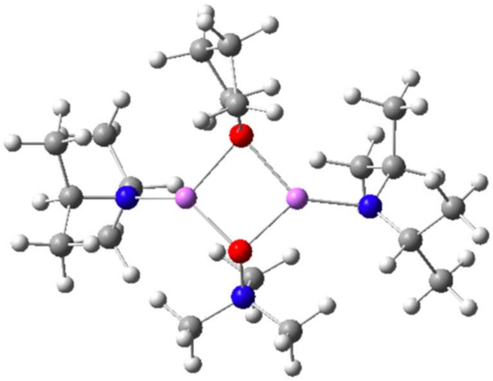

Figure S36. Representative ball and stick model ground state structure **S7**.

#m062x/jul-cc-pvDz opt=(calcfc,noeigen) optcyc=50 freq SCF=XQC scrf=(pcm,solvent=THF,read) nosymm

EE = -1080.502658 (Hartree)
ZPE_{298} = -1079.864983
E_{298} = -1079.829904
H_{298} = -1079.828959
G_{298} = -1079.930663
S = 214.053 (cal/mol·K)

3	-0.338276	-0.951149	0.375218
8	-0.435549	-0.495676	2.366029
3	1.599210	-0.735671	2.273237
8	1.492278	-1.421049	0.507660
7	-1.303504	-0.043659	-0.960578
6	-1.302930	1.386964	-1.195164

6	-2.318875	-0.758528	-1.714234
1	-0.572676	1.829459	-0.488266
6	-0.806014	1.782150	-2.601681
6	-2.636077	2.106665	-0.908322
1	-2.713095	-0.143057	-2.547536
6	-1.739629	-2.024460	-2.360236
6	-3.537397	-1.153756	-0.857291
1	-1.475035	1.382958	-3.378026
1	-0.758657	2.874793	-2.728808
1	0.196467	1.365785	-2.775331
1	-3.422473	1.764983	-1.597170
1	-2.972662	1.898989	0.117721
1	-2.535316	3.195737	-1.029572
1	-0.969094	-1.762340	-3.097639
1	-1.270800	-2.663904	-1.593860
1	-2.515049	-2.621099	-2.864590
1	-3.212202	-1.825293	-0.045379
1	-3.989528	-0.266133	-0.396916
1	-4.310080	-1.675146	-1.445558
6	-1.101131	-1.493780	3.186088
6	-2.218006	-0.754429	3.915182
6	-2.538393	0.386380	2.946067
6	-1.152389	0.764177	2.454128
1	-1.496215	-2.269701	2.516425
1	-0.357521	-1.937920	3.857881
1	-3.072932	-1.407187	4.117714
1	-1.850820	-0.351851	4.867654
1	-3.150563	0.026657	2.108303
1	-3.048808	1.228473	3.424536
1	-1.141877	1.214128	1.455880
1	-0.633531	1.412576	3.172119
7	3.043313	0.070420	3.158730
6	4.273736	-0.623786	3.487832
6	2.840975	1.300887	3.900987
1	4.256981	-1.577061	2.924504
6	5.551083	0.117302	3.037379
6	4.430180	-1.023026	4.969017
1	3.783629	1.661358	4.359341
6	2.361703	2.419557	2.966618
6	1.832064	1.142727	5.056348
1	5.497807	0.351876	1.964955
1	5.666628	1.066029	3.581820
1	6.455527	-0.483160	3.220587
1	4.495824	-0.131086	5.610094
1	3.564974	-1.615131	5.297889
1	5.343018	-1.617057	5.128923
1	1.459952	2.093278	2.422844
1	2.112926	3.342860	3.511950
1	3.134784	2.649693	2.220662
1	0.869867	0.787810	4.653372

1	2.185238	0.393965	5.776361
1	1.656684	2.089272	5.593331
7	2.360038	-0.920075	-0.443538
6	3.739557	-1.355021	-0.099887
6	1.954103	-1.436810	-1.778217
6	2.288798	0.569173	-0.436746
1	3.744350	-2.447966	-0.086816
1	4.436072	-0.969704	-0.852310
1	3.957260	-0.946250	0.891997
1	0.934994	-1.073347	-1.957278
1	2.654947	-1.065458	-2.533554
1	1.979243	-2.528806	-1.726310
1	1.250407	0.835714	-0.657686
1	2.594527	0.895497	0.563868
1	2.963966	0.963486	-1.203880

Structures for Confirmation of Activation Energy with Higher Order Theory S8-S10

Transition Structure S8 – First Deprotonation of Anti-planar Hydrogen with No THF

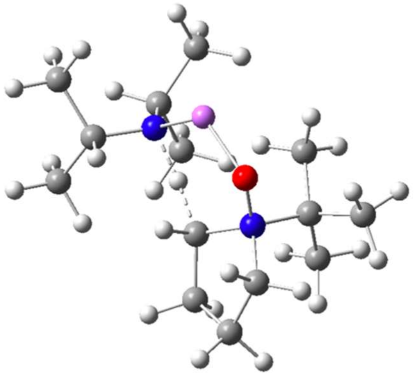

Figure S37. Representative ball and stick model for anti **TS S8**

M06-2X/jul-cc-pVDZ, Transition Structure S8

#m062x/jul-cc-pvDz opt=(calcfc,noeigen,z-matrix,ts) optcyc=50 freq scrf=(pcm,solvent=THF)

EE = -744.093123 (Hartree)
ZPE_{298} = -743.653008
E_{298} = -743.631684
H_{298} = -743.630740
G_{298} = -743.700503
S = 146.829 (cal/mol·K)

7	0.000000	0.000000	0.000000
8	0.000000	0.000000	1.385640
6	1.480269	0.000000	-0.455442
6	-0.763424	-1.200243	-0.518168
6	-0.836003	1.180916	-0.494105
6	-1.426058	-0.788103	-1.838611
6	-1.350720	0.765869	-1.869950
1	-1.502874	-1.375801	0.266562
1	-0.117152	-2.077002	-0.588275
1	-2.464904	-1.136552	-1.848268
1	-0.929113	-1.242370	-2.703733
1	-2.344269	1.204198	-2.026530
1	-0.726073	1.104513	-2.704504
1	-1.705224	1.052378	0.175689
1	-0.417454	2.429221	0.063475
6	2.129754	-1.326818	-0.055462

6	1.619504	0.213426	-1.960679
6	2.196375	1.135258	0.270524
1	1.904792	-2.135522	-0.760837
1	3.218108	-1.188001	-0.053988
1	1.813416	-1.614587	0.952812
1	1.052181	-0.518629	-2.544920
1	1.321412	1.226759	-2.249093
1	2.679669	0.084853	-2.213525
1	1.622244	2.062532	0.208581
1	2.382531	0.877861	1.318930
1	3.166777	1.301335	-0.214696
3	0.116679	1.750577	2.042222
-1	0.084210	2.100000	0.423956
7	-0.298067	3.392664	0.966630
6	0.507845	4.536866	0.531350
6	-1.695476	3.739835	1.247392
1	-0.067548	5.468740	0.664270
6	1.772618	4.694809	1.380725
6	0.865238	4.469046	-0.962166
1	-2.198004	2.787125	1.493673
6	-1.861010	4.648391	2.470477
6	-2.448316	4.316887	0.037807
1	2.374052	5.553708	1.048215
1	1.503146	4.845904	2.434690
1	2.402043	3.795281	1.314802
1	1.388578	5.381923	-1.281254
1	1.520964	3.613527	-1.178355
1	-0.043134	4.356587	-1.568326
1	-2.924310	4.827125	2.686230
1	-1.399619	4.189528	3.355207
1	-1.385585	5.626431	2.308465
1	-2.371706	3.633510	-0.818746
1	-3.512056	4.463875	0.272784
1	-2.034641	5.290478	-0.261771

M06-2X/jul-cc-pVTZ, Transition Structure S8

#m062x/jul-cc-pvTz opt=(calcfc,noeigen,z-matrix,ts) optcyc=50 freq scrf=(pcm,solvent=THF)

EE = -744.284644 (Hartree)
ZPE_{298} = -743.843513
E_{298} = -743.822281
H_{298} = -743.821337
G_{298} = -743.890836
S = 146.273 (cal/mol·K)

7	0.000000	0.000000	0.000000
8	0.000000	0.000000	1.385818
6	1.479219	0.000000	-0.454202
6	-0.764290	-1.196415	-0.520759

6	-0.835741	1.179481	-0.491612
6	-1.424967	-0.781418	-1.837376
6	-1.343653	0.770214	-1.867482
1	-1.500636	-1.372760	0.256403
1	-0.123362	-2.068036	-0.592947
1	-2.458463	-1.122641	-1.845472
1	-0.933972	-1.232899	-2.697806
1	-2.327203	1.210309	-2.029875
1	-0.714665	1.102623	-2.690985
1	-1.701280	1.053163	0.170424
1	-0.424932	2.425965	0.078641
6	2.128152	-1.325655	-0.059567
6	1.623061	0.217746	-1.955426
6	2.196561	1.130698	0.272179
1	1.902903	-2.127937	-0.760741
1	3.209385	-1.188233	-0.061395
1	1.817377	-1.614796	0.942228
1	1.057151	-0.504868	-2.539804
1	1.330847	1.226822	-2.239196
1	2.675967	0.087510	-2.206189
1	1.628187	2.053650	0.208339
1	2.379244	0.879514	1.315262
1	3.161439	1.296493	-0.207940
3	0.109221	1.740719	2.048041
-1	0.075138	2.095674	0.440356
7	-0.314909	3.383325	0.990630
6	0.484424	4.533845	0.566989
6	-1.712857	3.717064	1.275478
1	-0.084678	5.457550	0.719037
6	1.756507	4.678855	1.403209
6	0.831733	4.489443	-0.927084
1	-2.208448	2.766690	1.509169
6	-1.884999	4.611295	2.505120
6	-2.467527	4.306528	0.076241
1	2.347915	5.540302	1.085046
1	1.501822	4.804346	2.456545
1	2.383638	3.789606	1.310988
1	1.368552	5.390314	-1.231459
1	1.464476	3.630280	-1.158724
1	-0.074648	4.404962	-1.527578
1	-2.941589	4.776271	2.725397
1	-1.417575	4.155520	3.379009
1	-1.424716	5.589010	2.349906
1	-2.388682	3.640202	-0.784314
1	-3.524448	4.447953	0.309773
1	-2.058058	5.278202	-0.207615

M06-2X/aug-cc-pVDZ, Transition Structure S8

#m062x/aug-cc-pvDz opt=(calcfc,noeigen,z-matrix,ts) optcyc=50 freq scrf=(pcm,solvent=THF)

EE = -744.106408 (Hartree)
ZPE_{298} = -743.666157
E_{298} = -743.645007
H_{298} = -743.644063
G_{298} = -743.713144
S = 145.393 (cal/mol·K)

7	0.000000	0.000000	0.000000
8	0.000000	0.000000	1.385658
6	1.480174	0.000000	-0.456806
6	-0.768850	-1.197491	-0.515594
6	-0.830902	1.182738	-0.495955
6	-1.416516	-0.787999	-1.843885
6	-1.347443	0.767486	-1.871819
1	-1.517475	-1.356158	0.262226
1	-0.129983	-2.079265	-0.571469
1	-2.453054	-1.140309	-1.865729
1	-0.905302	-1.238216	-2.701463
1	-2.342102	1.201716	-2.023487
1	-0.725265	1.108880	-2.705040
1	-1.698751	1.063974	0.174246
1	-0.397533	2.427411	0.059305
6	2.124433	-1.337073	-0.084473
6	1.621013	0.245603	-1.956986
6	2.201011	1.118226	0.291818
1	1.895052	-2.129475	-0.805491
1	3.212502	-1.202521	-0.082119
1	1.808315	-1.643359	0.917471
1	1.057728	-0.476020	-2.556080
1	1.319384	1.263190	-2.222645
1	2.680931	0.125783	-2.209927
1	1.627165	2.044577	0.249485
1	2.389259	0.840729	1.333414
1	3.167592	1.298443	-0.193116
3	0.128399	1.750522	2.041549
-1	0.102362	2.093411	0.417842
7	-0.263040	3.393465	0.953681
6	0.561562	4.516309	0.498918
6	-1.655303	3.766115	1.224920
1	0.001034	5.457753	0.610634
6	1.826432	4.673886	1.348947
6	0.925271	4.411416	-0.991575
1	-2.172341	2.826805	1.484348
6	-1.809662	4.698819	2.431243

6	-2.394115	4.335610	0.002531
1	2.435487	5.521572	1.005361
1	1.555553	4.841817	2.398937
1	2.447087	3.768847	1.295375
1	1.450818	5.314450	-1.329706
1	1.582470	3.551929	-1.180143
1	0.020518	4.283906	-1.598422
1	-2.869628	4.892875	2.644544
1	-1.351659	4.251161	3.322408
1	-1.324692	5.667400	2.248672
1	-2.321968	3.637458	-0.841237
1	-3.455707	4.500960	0.229004
1	-1.964724	5.297952	-0.307330

MP2/jul-cc-pVDZ, Transition Structure S8

#MP2/jul-cc-pvDz opt=(calcfc,noeigen,z-matrix,ts) optcyc=50 freq scrf=(pcm,solvent=THF)

EE = -742.096911 (Hartree)
ZPE_{298} = -741.658269
E_{298} = -741.636419
H_{298} = -741.635474
G_{298} = -741.706484
S = 149.452 (cal/mol·K)

7	0.000000	0.000000	0.000000
8	0.000000	0.000000	1.405570
6	1.482994	0.000000	-0.455076
6	-0.771417	-1.211314	-0.510516
6	-0.831395	1.186449	-0.498564
6	-1.413953	-0.806342	-1.848635
6	-1.353026	0.758533	-1.877197
1	-1.523154	-1.360529	0.275099
1	-0.126423	-2.097126	-0.555727
1	-2.453821	-1.166985	-1.878268
1	-0.891866	-1.256298	-2.707086
1	-2.356927	1.188504	-2.030869
1	-0.732539	1.102835	-2.718592
1	-1.701983	1.062838	0.181435
1	-0.403777	2.479534	0.030666
6	2.137481	-1.332747	-0.062259
6	1.622269	0.221793	-1.966028
6	2.196383	1.140086	0.277662
1	1.895920	-2.146467	-0.763547
1	3.230764	-1.194144	-0.084911
1	1.842675	-1.618675	0.957371
1	1.070741	-0.521932	-2.558338
1	1.308228	1.234538	-2.254960

1	2.691056	0.111771	-2.214683
1	1.609826	2.065172	0.224798
1	2.390711	0.878730	1.327506
1	3.165959	1.318771	-0.216500
3	0.029675	1.803041	2.072060
-1	0.104120	2.162973	0.393750
7	-0.318801	3.463129	0.879944
6	0.519262	4.599447	0.448927
6	-1.739757	3.834794	1.055075
1	-0.053594	5.543320	0.545633
6	1.756508	4.752294	1.348918
6	0.943995	4.497245	-1.030977
1	-2.269040	2.887764	1.286629
6	-1.970428	4.777068	2.249100
6	-2.407059	4.400269	-0.216426
1	2.385947	5.601562	1.027296
1	1.448304	4.923580	2.393299
1	2.375924	3.839823	1.318341
1	1.520399	5.389548	-1.334692
1	1.578285	3.611912	-1.202472
1	0.063665	4.414560	-1.687680
1	-3.047150	4.979419	2.390016
1	-1.576841	4.329190	3.176379
1	-1.466391	5.746729	2.097467
1	-2.285472	3.697887	-1.056966
1	-3.487085	4.560992	-0.050549
1	-1.966151	5.368975	-0.506138

Transition Structure S9 – First Deprotonation of Syn-planar Hydrogen

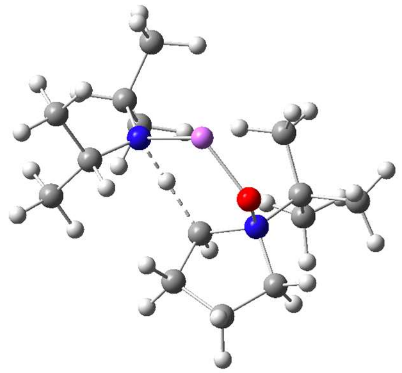

Figure S38. Representative ball and stick model for syn **TS S9**.

M06-2x/jul-cc-pVDZ, Transition Structure S9

#m062x/jul-cc-pvDz opt=(calcfc,noeigen,z-matrix,ts) optcyc=50 freq scrf=(pcm,solvent=THF)

EE = -744.104646 (Hartree)
ZPE_{298} = -743.665256
E_{298} = -743.643618
H_{298} = -743.642673
G_{298} = -743.713055
S = 148.130 (cal/mol·K)

7	0.000000	0.000000	0.000000
8	0.000000	0.000000	1.389366
6	1.454716	0.000000	-0.501786
6	-0.742642	-1.235207	-0.432748
6	-0.835334	1.168753	-0.499236
6	-2.216822	-0.886130	-0.247338
6	-2.261624	0.664606	-0.243178
1	-0.400586	-2.072801	0.179606
1	-0.505226	-1.422413	-1.485024
1	-2.596603	-1.297610	0.694001
1	-2.810982	-1.316610	-1.061645
1	-2.649542	1.011461	0.722738
1	-2.938548	1.053746	-1.014835
1	-0.560217	2.374019	0.323185
1	-0.678980	1.197710	-1.586799
6	2.089699	-1.349468	-0.166993
6	1.537006	0.257535	-2.008463
6	2.204680	1.109333	0.229252
1	1.739854	-2.145736	-0.835058
1	3.176426	-1.261267	-0.289444
1	1.875666	-1.622511	0.871909
1	0.871941	-0.391380	-2.589531
1	1.315394	1.302023	-2.251946
1	2.565956	0.046293	-2.326312
1	1.636037	2.043714	0.199343
1	2.407017	0.829205	1.268609
1	3.166951	1.273959	-0.271676
3	0.268255	1.646482	2.168048
-1	-0.104428	2.007525	0.707813
7	-0.400793	3.261789	1.234515
6	-1.743453	3.535994	1.764064
6	0.280712	4.460691	0.733138
1	-2.147463	2.558931	2.081670
6	-1.729530	4.425178	3.012495
6	-2.718228	4.089821	0.714249
1	-0.337451	5.350255	0.938756
6	1.615079	4.694394	1.447784
6	0.479011	4.429758	-0.789441
1	-2.744533	4.543468	3.418077
1	-1.093668	3.983866	3.791642
1	-1.342721	5.429048	2.786748

1	-3.733372	4.173508	1.127986
1	-2.411782	5.090701	0.377584
1	-2.750785	3.427600	-0.160409
1	2.110767	5.606371	1.083454
1	1.450922	4.795687	2.529008
1	2.299106	3.848906	1.282774
1	-0.484645	4.305733	-1.300562
1	0.943439	5.363397	-1.137281
1	1.126462	3.595863	-1.094465

M06-2x/jul-cc-pVTZ, Transition Structure S9

#m062x/jul-cc-pvTz opt=(calcfc,noeigen,z-matrix,ts) optcyc=50 freq scrf=(pcm,solvent=THF)

EE = -744.296553 (Hartree)
ZPE_{298} = -743.856323
E_{298} = -743.834639
H_{298} = -743.833694
G_{298} = -743.904499
S = 149.021 (cal/mol·K)

7	0.000000	0.000000	0.000000
8	0.000000	0.000000	1.389329
6	1.452783	0.000000	-0.503800
6	-0.743889	-1.231540	-0.434040
6	-0.836502	1.166976	-0.496252
6	-2.211384	-0.886032	-0.229677
6	-2.261183	0.662203	-0.250013
1	-0.397708	-2.069274	0.162622
1	-0.519621	-1.408405	-1.483631
1	-2.565573	-1.275765	0.722117
1	-2.816903	-1.332304	-1.016691
1	-2.656943	1.017516	0.700875
1	-2.927987	1.035350	-1.027650
1	-0.572223	2.363016	0.341580
1	-0.672870	1.212321	-1.573924
6	2.087529	-1.349297	-0.179600
6	1.533800	0.264465	-2.006744
6	2.208437	1.101346	0.227668
1	1.728412	-2.139719	-0.837899
1	3.165471	-1.265027	-0.316752
1	1.889399	-1.621950	0.855182
1	0.869978	-0.374932	-2.586085
1	1.315222	1.303850	-2.243421
1	2.554718	0.054767	-2.326400
1	1.650315	2.034418	0.197028
1	2.406633	0.825655	1.261446
1	3.166098	1.260597	-0.268344
3	0.258249	1.632884	2.177712
-1	-0.118326	1.993648	0.725695

7	-0.417985	3.245733	1.261729
6	-1.755409	3.500650	1.808498
6	0.248003	4.452334	0.764506
1	-2.145894	2.524654	2.119271
6	-1.737436	4.379552	3.061328
6	-2.744968	4.058218	0.778246
1	-0.367752	5.330783	0.983418
6	1.588830	4.688052	1.461025
6	0.431867	4.435322	-0.757445
1	-2.740282	4.480790	3.480679
1	-1.086748	3.948133	3.823122
1	-1.371934	5.383336	2.837002
1	-3.749233	4.133351	1.199871
1	-2.448207	5.056621	0.450120
1	-2.783852	3.409456	-0.097283
1	2.071391	5.600004	1.102136
1	1.442104	4.775168	2.538540
1	2.270191	3.854203	1.278336
1	-0.529635	4.316753	-1.258418
1	0.893327	5.362936	-1.101336
1	1.069356	3.606528	-1.070596

M06-2x/aug-cc-pVDZ, Transition Structure S9

#m062x/aug-cc-pvDz opt=(calcfc,noeigen,z-matrix,ts) optcyc=50 freq scrf=(pcm,solvent=THF)

EE = -744.117814 (Hartree)
ZPE_{298} = -743.678530
E_{298} = -743.656963
H_{298} = -743.656019
G_{298} = -743.726062
S = 147.419 (cal/mol·K)

7	0.000000	0.000000	0.000000
8	0.000000	0.000000	1.389386
6	1.454384	0.000000	-0.501871
6	-0.745293	-1.233763	-0.432813
6	-0.833785	1.169609	-0.499721
6	-2.218706	-0.881523	-0.243157
6	-2.259817	0.670270	-0.234006
1	-0.402702	-2.070584	0.178534
1	-0.509959	-1.418275	-1.484999
1	-2.594561	-1.294013	0.698219
1	-2.814496	-1.306558	-1.057965
1	-2.635306	1.016295	0.735637
1	-2.940007	1.063128	-0.998937
1	-0.545871	2.370170	0.324006

1	-0.682569	1.194028	-1.586600
6	2.087170	-1.353072	-0.177123
6	1.536474	0.268800	-2.006583
6	2.205068	1.103422	0.237826
1	1.733165	-2.143686	-0.848568
1	3.172727	-1.266115	-0.302424
1	1.875156	-1.630852	0.860196
1	0.872187	-0.377010	-2.590377
1	1.312202	1.313803	-2.241363
1	2.564849	0.062269	-2.325537
1	1.630052	2.032186	0.221423
1	2.415025	0.814105	1.271962
1	3.161058	1.282711	-0.267417
3	0.278069	1.640221	2.176116
-1	-0.090794	1.999386	0.705352
7	-0.374767	3.257005	1.233356
6	-1.715360	3.548850	1.757399
6	0.321418	4.443812	0.724171
1	-2.129409	2.580190	2.080469
6	-1.695988	4.449453	2.997829
6	-2.680626	4.105395	0.699682
1	-0.284972	5.340879	0.922169
6	1.658835	4.666986	1.437398
6	0.523002	4.399534	-0.798182
1	-2.708940	4.575577	3.403300
1	-1.060830	4.012671	3.778863
1	-1.306955	5.448730	2.760647
1	-3.696452	4.197951	1.106548
1	-2.364328	5.101936	0.362587
1	-2.711773	3.439084	-0.170764
1	2.160616	5.573849	1.072021
1	1.496109	4.769587	2.517679
1	2.334682	3.816769	1.270248
1	-0.438871	4.274758	-1.310068
1	0.991717	5.327615	-1.151000
1	1.169896	3.562550	-1.091951

MP2/jul-cc-pVDZ, Transition Structure S9

#MP2/jul-cc-pvDz opt=(calcfc,noeigen,z-matrix,ts) optcyc=50 freq scrf=(pcm,solvent=THF)

EE = -742.107923 (Hartree)
ZPE_{298} = -741.669311
E_{298} = -741.647375
H_{298} = -741.646431
G_{298} = -741.717476
S = 149.527 (cal/mol·K)

7	0.000000	0.000000	0.000000

8	0.000000	0.000000	1.408445
6	1.458155	0.000000	-0.501871
6	-0.746101	-1.247440	-0.429525
6	-0.835151	1.174680	-0.492137
6	-2.230111	-0.883252	-0.280044
6	-2.255120	0.672847	-0.175360
1	-0.417029	-2.076153	0.209773
1	-0.483553	-1.451281	-1.477714
1	-2.663132	-1.356035	0.614157
1	-2.793728	-1.242500	-1.155190
1	-2.568393	0.957840	0.840789
1	-2.980221	1.118860	-0.876405
1	-0.555045	2.442428	0.279993
1	-0.716350	1.178269	-1.592986
6	2.095236	-1.356377	-0.171061
6	1.529591	0.261445	-2.014503
6	2.215492	1.109161	0.233710
1	1.739220	-2.157840	-0.836620
1	3.185696	-1.267723	-0.305366
1	1.892079	-1.627780	0.875031
1	0.871885	-0.403083	-2.595135
1	1.288308	1.305764	-2.258821
1	2.565842	0.066615	-2.337149
1	1.645357	2.046902	0.214012
1	2.428418	0.824398	1.273946
1	3.176745	1.276542	-0.280114
3	0.120466	1.716378	2.182952
-1	-0.086584	2.100303	0.671778
7	-0.430472	3.370497	1.140620
6	-1.813156	3.709473	1.549691
6	0.306220	4.538564	0.614753
1	-2.276155	2.756214	1.871776
6	-1.862004	4.654848	2.763118
6	-2.689038	4.256453	0.403456
1	-0.296980	5.454859	0.766694
6	1.624538	4.760032	1.373931
6	0.565993	4.440552	-0.901870
1	-2.905059	4.828407	3.082010
1	-1.306631	4.223255	3.612166
1	-1.418121	5.636900	2.528195
1	-3.733277	4.386868	0.738720
1	-2.326083	5.238388	0.055413
1	-2.682151	3.561513	-0.451003
1	2.164963	5.644526	0.990887
1	1.424508	4.912718	2.447101
1	2.288645	3.885553	1.268528
1	-0.379666	4.317016	-1.453107
1	1.067356	5.353943	-1.268364
1	1.210517	3.580201	-1.144241

Ground State Structure S10 – First Deprotonation Reactant Complex

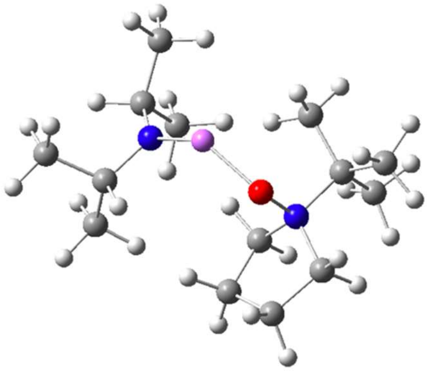

Figure S39. Representative Ball and Stick Model for shared ground state structure **S10**

M06-2x/jul-cc-pVDZ, Structure S10

#m062x/jul-cc-pvDz opt=(calcfc,noeigen,z-matrix) freq scrf=(pcm,solvent=THF)

EE = -744.129565 (Hartree)
ZPE_{298} = -743.685850
E_{298} = -743.663555
H_{298} = -743.662611
G_{298} = -743.735610
S = 153.640 (cal/mol·K)

7	0.000000	0.000000	0.000000
8	0.000000	0.000000	1.375778
6	1.448727	0.000000	-0.529612
6	-0.804114	-1.189733	-0.443454
6	-0.835383	1.157698	-0.486540
6	-2.255114	-0.793118	-0.153868
6	-2.252677	0.753114	-0.093732
1	-0.453868	-2.052065	0.125155
1	-0.631462	-1.347480	-1.513249
1	-2.588279	-1.218799	0.796560
1	-2.912353	-1.173247	-0.942622
1	-2.479176	1.104703	0.916792
1	-2.978793	1.199672	-0.781134
1	-0.480812	2.073372	0.003056
1	-0.736747	1.215685	-1.574514
6	2.056416	-1.370317	-0.241000
6	1.497255	0.299258	-2.029167
6	2.220030	1.077726	0.228924
1	1.674455	-2.140392	-0.921878
1	3.141649	-1.302549	-0.383215

1	1.858147	-1.663197	0.795713
1	0.811436	-0.325331	-2.613574
1	1.292704	1.354452	-2.242473
1	2.514317	0.081044	-2.376439
1	1.669849	2.026121	0.265192
1	2.442892	0.750100	1.249283
1	3.170896	1.252129	-0.289053
3	0.372110	1.577888	2.295156
-1	0.003852	1.702421	0.345833
7	-0.100365	3.372216	2.056401
6	-1.490319	3.555106	2.440004
6	0.573980	4.590814	1.665753
1	-1.902730	2.541028	2.619687
6	-1.681283	4.317615	3.768522
6	-2.398693	4.190292	1.367851
1	0.074327	5.487285	2.089269
6	2.009494	4.601501	2.204554
6	0.623980	4.815296	0.137924
1	-2.743633	4.379289	4.052343
1	-1.133553	3.810064	4.573830
1	-1.297754	5.345564	3.690723
1	-3.451420	4.188662	1.689066
1	-2.108290	5.234941	1.180236
1	-2.323438	3.640349	0.419348
1	2.574694	5.482899	1.864466
1	2.004266	4.586611	3.302887
1	2.539261	3.700958	1.854712
1	-0.381168	4.781049	-0.299143
1	1.079699	5.786098	-0.116207
1	1.226032	4.026372	-0.339864

M06-2x/jul-cc-pVTZ, Structure S10

#m062x/jul-cc-pvTz opt=(calcfc,noeigen,z-matrix) freq scrf=(pcm,solvent=THF)

EE = -744.324404 (Hartree)
ZPE_{298} = -743.879374
E_{298} = -743.857282
H_{298} = -743.856338
G_{298} = -743.928876
S = 152.668 (cal/mol·K)

7	0.000000	0.000000	0.000000
8	0.000000	0.000000	1.374732
6	1.450915	0.000000	-0.524147
6	-0.800426	-1.190530	-0.444537
6	-0.837759	1.150620	-0.493507
6	-2.247254	-0.800935	-0.147615

6	-2.252888	0.743504	-0.107288
1	-0.447712	-2.048525	0.114890
1	-0.632892	-1.343024	-1.508547
1	-2.562464	-1.210699	0.807419
1	-2.906926	-1.194463	-0.917243
1	-2.484337	1.103536	0.891482
1	-2.973469	1.176404	-0.797226
1	-0.500063	2.065427	-0.008324
1	-0.730973	1.208755	-1.573481
6	2.058597	-1.365780	-0.227261
6	1.505049	0.287695	-2.023161
6	2.223073	1.079806	0.225310
1	1.672745	-2.137611	-0.892464
1	3.135508	-1.301702	-0.378321
1	1.869486	-1.647470	0.806754
1	0.828148	-0.339429	-2.601552
1	1.298067	1.333366	-2.244660
1	2.517624	0.073299	-2.362988
1	1.687969	2.029411	0.231378
1	2.424534	0.782206	1.251639
1	3.176030	1.232162	-0.280985
3	0.127929	1.563411	2.359691
-1	-0.014588	1.701220	0.340485
7	-0.359843	3.351834	2.186668
6	-1.758429	3.509325	2.539202
6	0.303453	4.579730	1.816139
1	-2.159263	2.496963	2.704747
6	-1.994205	4.263778	3.862926
6	-2.650091	4.137577	1.452566
1	-0.206068	5.461145	2.238701
6	1.731854	4.598219	2.365772
6	0.374042	4.819553	0.293652
1	-3.055060	4.300011	4.125224
1	-1.452536	3.774639	4.673418
1	-1.637725	5.293630	3.793479
1	-3.703474	4.119428	1.743360
1	-2.371668	5.180208	1.281065
1	-2.541593	3.599717	0.508938
1	2.288157	5.483344	2.046700
1	1.722314	4.562882	3.455987
1	2.268013	3.714909	2.005665
1	-0.616494	4.783164	-0.157341
1	0.827509	5.786360	0.054199
1	0.982801	4.041357	-0.175296

M06-2x/aug-cc-pVDZ, Structure S10

#m062x/aug-cc-pvDz opt=(calcfc,noeigen,z-matrix) freq scrf=(pcm,solvent=THF)

EE = -744.134602 (Hartree)
ZPE_{298} = -743.689849
E_{298} = -743.668151
H_{298} = -743.667207
G_{298} = -743.738412
S = 149.865 (cal/mol·K)

7	0.000000	0.000000	0.000000
8	0.000000	0.000000	1.374533
6	1.479518	0.000000	-0.482917
6	-0.816697	-1.178800	-0.477935
6	-0.794928	1.173330	-0.537026
6	-1.414797	-0.813671	-1.833598
6	-1.425746	0.735556	-1.860092
1	-1.593068	-1.254746	0.286201
1	-0.212747	-2.084260	-0.454353
1	-2.424609	-1.226322	-1.911074
1	-0.832732	-1.225451	-2.662268
1	-2.444648	1.128115	-1.921900
1	-0.873864	1.118150	-2.721657
1	-1.553177	1.332452	0.232676
1	-0.165966	2.063974	-0.569657
6	2.092432	-1.367507	-0.190653
6	1.599218	0.322318	-1.971777
6	2.221563	1.065236	0.326725
1	1.805525	-2.121815	-0.931786
1	3.182675	-1.264270	-0.230470
1	1.811410	-1.703121	0.812762
1	1.033488	-0.368766	-2.602255
1	1.305018	1.353354	-2.194854
1	2.656051	0.215718	-2.240223
1	1.656933	2.000842	0.397791
1	2.438023	0.703770	1.336029
1	3.173189	1.277175	-0.173423
3	0.239845	1.631609	2.249761
-1	0.370542	1.694486	-0.313460
7	-0.114483	3.425405	1.847568
6	0.658496	4.598728	1.510220
6	-1.542861	3.658192	1.967153
1	0.153309	5.531042	1.836974
6	2.011360	4.562378	2.230631
6	0.925874	4.769006	-0.004711
1	-2.012378	2.662598	2.096835
6	-1.943196	4.470332	3.218476
6	-2.230621	4.286192	0.736374
1	2.655515	5.407316	1.947228
1	1.864582	4.575464	3.317791
1	2.537342	3.631232	1.968976
1	1.436646	5.720083	-0.220820

1	1.572012	3.955336	-0.366673
1	-0.006392	4.741598	-0.577927
1	-3.035097	4.575658	3.305337
1	-1.566002	3.974796	4.121903
1	-1.513042	5.481070	3.179183
1	-2.009126	3.710115	-0.171635
1	-3.321152	4.319957	0.869685
1	-1.881489	5.316573	0.580402

MP2/jul-cc-pVDZ, Structure S10

#MP2/jul-cc-pvDz opt=(calcfc,noeigen,z-matrix) optcyc=50 freq scrf=(pcm,solvent=THF)

EE = -742.131517 (Hartree)
ZPE_{298} = -741.688535
E_{298} = -741.665897
H_{298} = -741.664953
G_{298} = -741.738873
S = 155.577 (cal/mol·K)

7	0.000000	0.000000	0.000000
8	0.000000	0.000000	1.391882
6	1.453212	0.000000	-0.530445
6	-0.800652	-1.203248	-0.442180
6	-0.844748	1.158469	-0.489606
6	-2.256079	-0.814757	-0.136271
6	-2.268110	0.739873	-0.103051
1	-0.435241	-2.066914	0.123491
1	-0.635592	-1.352002	-1.519376
1	-2.571428	-1.228507	0.830393
1	-2.922961	-1.216930	-0.913021
1	-2.515328	1.108716	0.900529
1	-2.992831	1.167789	-0.811712
1	-0.505972	2.080035	0.010865
1	-0.735041	1.221403	-1.581444
6	2.075014	-1.363920	-0.210518
6	1.495928	0.262644	-2.043922
6	2.223406	1.106384	0.198846
1	1.692816	-2.157863	-0.870457
1	3.162827	-1.290297	-0.367824
1	1.891066	-1.633402	0.839284
1	0.821947	-0.394078	-2.614738
1	1.271498	1.311423	-2.288071
1	2.522565	0.054132	-2.385030
1	1.686675	2.066267	0.170320
1	2.420654	0.833555	1.244663
1	3.190528	1.241028	-0.312300
3	0.102375	1.651326	2.344835

-1	-0.020825	1.713401	0.357582
7	-0.395841	3.468324	2.030177
6	-1.809675	3.668328	2.359324
6	0.285623	4.689787	1.622609
1	-2.230627	2.658121	2.566489
6	-2.048084	4.489669	3.652811
6	-2.682386	4.267061	1.227786
1	-0.234808	5.599523	2.003655
6	1.710496	4.722755	2.205140
6	0.389281	4.864643	0.084628
1	-3.125372	4.571869	3.891413
1	-1.537533	4.008589	4.503186
1	-1.650292	5.514035	3.549776
1	-3.750833	4.275969	1.512885
1	-2.386268	5.308544	1.012857
1	-2.575116	3.680419	0.300490
1	2.289821	5.592424	1.843157
1	1.676461	4.754957	3.306558
1	2.248954	3.805209	1.905954
1	-0.601685	4.814563	-0.391158
1	0.854584	5.832602	-0.185925
1	1.012096	4.059236	-0.344098

THF and *tert*-Butyl Amine *N*-Oxide Structures

Ground Structure - THF

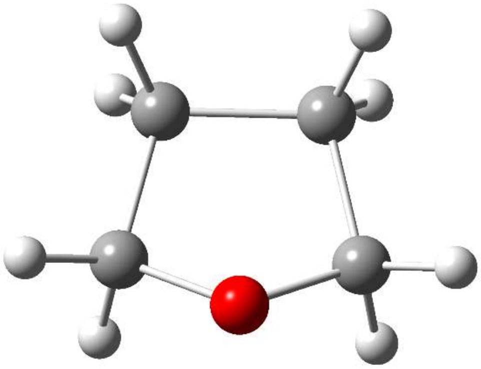

Figure S40. Representative ball and stick model for THF.

#m062x/jul-cc-pVDZ opt=(calcfc,noeigen,z-matrix) optcyc=50 freq scrf=(pcm,solvent=THF) nosymm volume=tight

EE = -232.372504 (Hartree)
ZPE_{298} = -232.255132
E_{298} = -232.250251
H_{298} = -232.249307
G_{298} = -232.283470
S = 71.901 (cal/mol·K)
Volume = 724.148 $bohr^3$/mol

8	0.000493	-1.199094	-0.301829
6	-1.129540	-0.467888	0.161374
6	-0.774526	1.010993	-0.052816
6	0.773011	1.012356	-0.050713
6	1.130467	-0.466697	0.159810
1	-2.006407	-0.803833	-0.402152
1	-1.288755	-0.679292	1.232803
1	-1.162682	1.365657	-1.014143
1	-1.196298	1.643524	0.736093
1	1.163353	1.371335	-1.009527
1	1.191205	1.642878	0.741707
1	2.006465	-0.800247	-0.406487
1	1.292694	-0.679859	1.230405

Ground Structure - Tert-butyl amine *N*-oxide

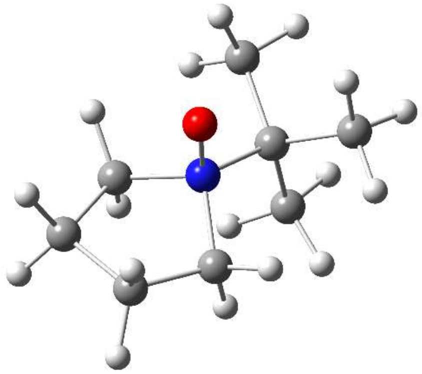

Figure S41. Representative ball and stick model for *tert*-butyl amine *N*-oxide.

#m062x/jul-cc-pVDZ opt=(mndofc,noeigen,z-matrix) optcyc=50 freq scrf=(pcm,solvent=THF,read)

EE =	-444.847206 (Hartree)
ZPE_{298} =	-444.599170
E_{298} =	-444.588569
H_{298} =	-444.587625
G_{298} =	-444.634007
S =	97.620 (cal/mol·K)

7	0.178584	-0.008221	-0.252290
8	0.341160	-0.030309	-1.610080
6	-1.322383	0.001979	0.112265
6	0.937074	-1.162343	0.343155
6	0.931752	1.178013	0.287147
6	2.396421	-0.778466	0.120829
6	2.404008	0.767877	0.179212
1	0.709583	-1.222432	1.412779
1	0.623114	-2.074669	-0.167364
1	3.032996	-1.229206	0.889511
1	2.735294	-1.127899	-0.857572
1	2.963784	1.143102	1.042548
1	2.855730	1.186486	-0.724051
1	0.636743	1.351743	1.327729
1	0.662381	2.037216	-0.328231
6	-1.997944	-1.124859	-0.664449
6	-1.555545	-0.202232	1.611354
6	-1.890937	1.347938	-0.331577
1	-1.558790	-2.101698	-0.428645
1	-3.055490	-1.152712	-0.372727
1	-1.922225	-0.949567	-1.740221
1	-1.356772	-1.233698	1.923217
1	-0.967967	0.480501	2.236158
1	-2.614224	0.003863	1.812254

1	-1.567857	1.570534	-1.353805
1	-2.985824	1.289922	-0.310698
1	-1.584528	2.161090	0.338174

Energy Decomposition Analyses on Solvent Aggregates

Energy Decomposition Analysis on Dissociation Transitions

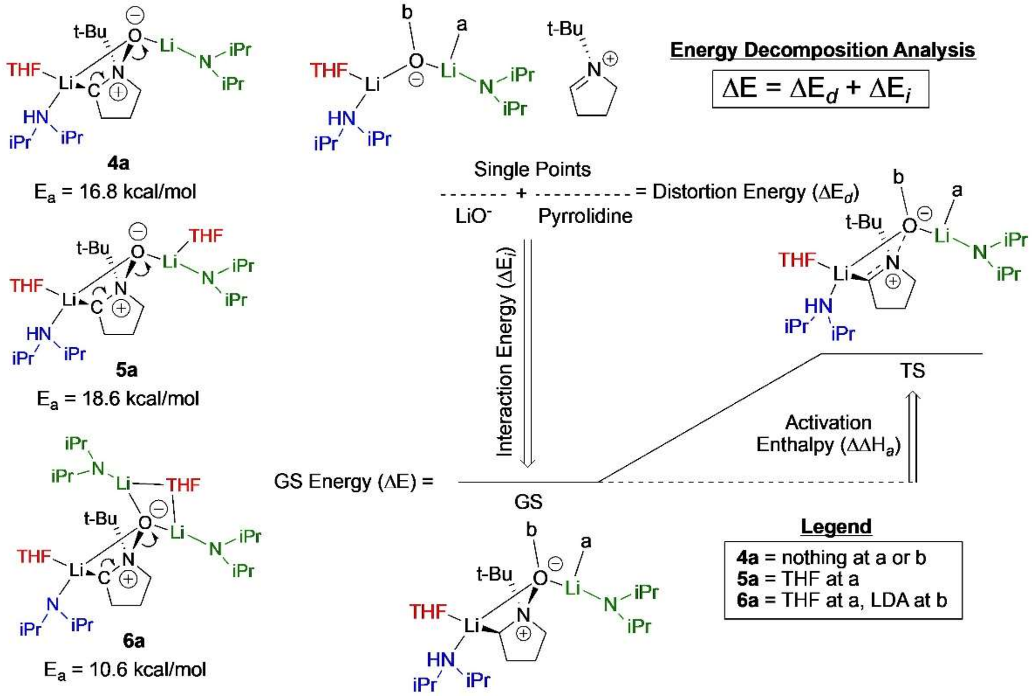

Figure S42. Diagram depicting the energy decomposition analysis completed on the dissociation transition structures in Figure 7.

We utilized an energy decomposition to further extract the details of the energy barriers for the N-O dissociation transition structure for the different solvation models (**4a**-**6a**), and we conducted an energy decomposition analysis of the LiO^- anion and iminium components using the distortion/interaction model of reactivity depicted in Figure S42. The difference between the total distortion energy of two components in the ground structure minus the calculated electronic energy of the intact ground structure provides us with an interaction energy of the four-member Li-O-C-N ring that is being broken during the N-O dissociation transition (Table S9). The addition of THF coordinating to LDA in **5a** increased the interaction energy compared to **4a**, signifying the stability provided by THF coordination on the Li-O-C-N ring and contributing to the increase in activation energy between the two transition structures. When that THF is bridged between two lithiums, as seen in **6a**, the stabilization of the Li-O-C-N ring is negated, as indicated by the decrease in interaction energy compared to **5a**. However, the distortion energies of both LiO- and tert-butyl pyrrolidine components in the ground structure of **6a** are significantly higher than those seen in either **4a** or **5a**, leading to an overall decrease in activation energy in **6a**.

Table S9. Energy decomposition analysis to determine the distortion and interaction energies for each dissociation transition structure in Figure S42.

Structure	GS Distortion Enthalpy		Total Distortion Enthalpy	Total Interaction Enthalpy	Activation Enthalpy
	LiO^-	Pyrrolidine			
**4a**	10.4	82.7	93.1	-116.5	16.8
**5a**	9.4	82.3	91.7	-125.0	18.6
**6a**	18.8	93.0	111.8	-117.4	10.6

Frequency Calculations for the Energy Decomposition on 4a

Lithium Oxide segment

EE = -906.811487 (Hartree)
ZPE_{298} = -906.288854
E_{298} = -906.261946
H_{298} = -906.261002
G_{298} = -906.346235
S = 344.842 (cal/mol·K)

Pyrrolidine segment

EE = -368.911654 (Hartree)
ZPE_{298} = -368.679816
E_{298} = -368.670152
H_{298} = -368.669208
G_{298} = -368.713777
S = 93.803 (cal/mol·K)

Frequency Calculations for the Energy Decomposition on 5a

Lithium Oxide segment

EE = -1139.188349 (Hartree)
ZPE_{298} = -1138.547845
E_{298} = -1138.516190
H_{298} = -1138.515246
G_{298} = -1138.612818
S = 205.357 (cal/mol·K)

Pyrrolidine segment

EE = -368.913847 (Hartree)
ZPE_{298} = -368.681698

E_{298} = -368.672045
H_{298} = -368.671101
G_{298} = -368.715700
S = 93.867 (cal/mol·K)

Frequency Calculations for the Energy Decomposition on 6a

Lithium Oxide segment

EE = -1438.507103 (Hartree)
ZPE_{298} = -1437.668085
E_{298} = -1437.621685
H_{298} = -1437.620741
G_{298} = -1437.748495
S = 268.881 (cal/mol·K)

Pyrrolidine segment

EE = -368.891988 (Hartree)
ZPE_{298} = -368.660854
E_{298} = -368.651031
H_{298} = -368.650087
G_{298} = -368.694778
S = 94.060 (cal/mol·K)

Energy Decomposition on LDA/THF Dimers

Table S10. Energy decomposition analysis to determine the distortion and interaction energies for each LDA-THF dimers **2** and **7**.

Structure	Distortion Energies				Total Distortion Energy	Interaction Energy
	LDA	LDA	THF	THF		
**2**	6.5	6.3	0.3	0.1	13.3	-59.2
**7**	3.9	4.5	0.5	0.5	9.5	-36.4

Frequency Calculations for the Energy Decomposition on 2

1st LDA segment

EE = -299.247224 (Hartree)
ZPE_{298} = -299.054174
E_{298} = -299.043645
H_{298} = -299.042701
G_{298} = -299.088556

S = 96.510 (cal/mol·K)

2nd LDA segment

EE = -299.247567 (Hartree)
ZPE_{298} = -299.055427
E_{298} = -299.045420
H_{298} = -299.044475
G_{298} = -299.089206
S = 94.144 (cal/mol·K)

1st THF segment

EE = -232.372067 (Hartree)
ZPE_{298} = -232.255030
E_{298} = -232.250125
H_{298} = -232.249181
G_{298} = -232.283273
S = 71.753 (cal/mol·K)

2nd THF segment

EE = -232.372345 (Hartree)
ZPE_{298} = -232.255291
E_{298} = -232.250379
H_{298} = -232.249435
G_{298} = -232.283395
S = 71.474 (cal/mol·K)

Frequency Calculations for the Energy Decomposition on 7

1st LDA segment

EE = -299.251380 (Hartree)
ZPE_{298} = -299.058264
E_{298} = -299.047693
H_{298} = -299.046748
G_{298} = -299.093103
S = 97.562 (cal/mol·K)

2nd LDA segment

EE = -299.250427 (Hartree)
ZPE_{298} = -299.056582

E_{298} = -299.046406
H_{298} = -299.045462
G_{298} = -299.090398
S = 94.576 (cal/mol·K)

1st THF segment

EE = -232.371653 (Hartree)
ZPE_{298} = -232.254623
E_{298} = -232.249773
H_{298} = -232.248828
G_{298} = -232.282452
S = 70.767 (cal/mol·K)

2nd THF segment

EE = -232.371660 (Hartree)
ZPE_{298} = -232.254641
E_{298} = -232.249823
H_{298} = -232.248879
G_{298} = -232.282357
S = 70.462 (cal/mol·K)

Frequency Calculations on Optimized Segments

Optimized LDA

EE = -299.257659 (Hartree)
ZPE_{298} = -299.064134
E_{298} = -299.053792
H_{298} = -299.052848
G_{298} = -299.098670
S = 96.441 (cal/mol·K)

Optimized THF

EE = -232.372504 (Hartree)
ZPE_{298} = -232.255132
E_{298} = -232.250251
H_{298} = -232.249307
G_{298} = -232.283470
S = 71.901 (cal/mol·K)

Energy Decomposition on LDA/THF/*N*-Oxide Dimers

Frequency Calculations for the Energy Decomposition on 8

1st LDA segment

EE = -299.249430 (Hartree)
ZPE_{298} = -299.056630
E_{298} = -299.046014
H_{298} = -299.045070
G_{298} = -299.091392
S = 97.492 (cal/mol·K)

2nd LDA segment

EE = -299.248289 (Hartree)
ZPE_{298} = -299.055441
E_{298} = -299.044953
H_{298} = -299.044009
G_{298} = -299.089663
S = 96.089 (cal/mol·K)

THF segment

EE = -232.372349 (Hartree)
ZPE_{298} = -232.255300
E_{298} = -232.250391
H_{298} = -232.249447
G_{298} = -232.283394
S = 71.446 (cal/mol·K)

N-Oxide segment

EE = -444.846924 (Hartree)
ZPE_{298} = -444.598902
E_{298} = -444.588191
H_{298} = -444.587247
G_{298} = -444.634241
S = 98.907 (cal/mol·K)

Frequency Calculations for the Energy Decomposition on 9

1st LDA segment

EE = -299.247438 (Hartree)
ZPE_{298} = -299.054977

E_{298} = -299.044246
H_{298} = -299.043302
G_{298} = -299.089931
S = 98.140 (cal/mol·K)

2nd LDA segment

EE = -299.250146 (Hartree)
ZPE_{298} = -299.057675
E_{298} = -299.046996
H_{298} = -299.046052
G_{298} = -299.092319
S = 97.377 (cal/mol·K)

THF segment

EE = -232.372315 (Hartree)
ZPE_{298} = -232.255308
E_{298} = -232.250381
H_{298} = -232.249437
G_{298} = -232.283441
S = 71.569 (cal/mol·K)

N-Oxide segment

EE = -444.846203 (Hartree)
ZPE_{298} = -444.598126
E_{298} = -444.587623
H_{298} = -444.586679
G_{298} = -444.632755
S = 96.976 (cal/mol·K)

Frequency Calculations for the Energy Decomposition on 10

1st LDA segment

EE = -299.251331 (Hartree)
ZPE_{298} = -299.058730
E_{298} = -299.047910
H_{298} = -299.046966
G_{298} = -299.094847
S = 100.775 (cal/mol·K)

2nd LDA segment

EE = -299.251575 (Hartree)
ZPE_{298} = -299.059541
E_{298} = -299.050204
H_{298} = -299.049259
G_{298} = -299.092841
S = 91.724 (cal/mol·K)

THF segment

EE = -232.371925 (Hartree)
ZPE_{298} = -232.254940
E_{298} = -232.250043
H_{298} = -232.249099
G_{298} = -232.282897
S = 71.134 (cal/mol·K)

N-Oxide segment

EE = -444.846577 (Hartree)
ZPE_{298} = -444.598605
E_{298} = -444.587962
H_{298} = -444.587018
G_{298} = -444.633558
S = 97.951 (cal/mol·K)

Frequency Calculations for the Optimized Segments

Optimized tert-Butyl Pyrrolidine N-Oxide

EE = -444.847206 (Hartree)
ZPE_{298} = -444.599170
E_{298} = -444.588569
H_{298} = -444.587625
G_{298} = -444.634007
S = 97.620 (cal/mol·K)

Energy Decomposition on Transition Structures

Frequency Calculations for the Energy Decomposition on 6a

1st LDA segment

EE = -299.250688 (Hartree)

ZPE_{298} = -299.058255
E_{298} = -299.048188
H_{298} = -299.047244
G_{298} = -299.092491
S = 95.232 (cal/mol·K)

2nd LDA segment

EE = -299.254683 (Hartree)
ZPE_{298} = -299.061841
E_{298} = -299.052170
H_{298} = -299.051226
G_{298} = -299.095050
S = 92.235 (cal/mol·K)

THF segment

EE = -232.371695 (Hartree)
ZPE_{298} = -232.254586
E_{298} = -232.250446
H_{298} = -232.249502
G_{298} = -232.281621
S = 67.602 (cal/mol·K)

N-Oxide segment

EE = -976.466399 (Hartree)
ZPE_{298} = -975.906273
E_{298} = -975.878817
H_{298} = -975.877873
G_{298} = -975.961758
S = 176.550 (cal/mol·K)

Frequency Calculations for the Energy Decomposition on 6b

1st LDA segment

EE = -299.246988 (Hartree)
ZPE_{298} = -299.055211
E_{298} = -299.045647
H_{298} = -299.044703
G_{298} = -299.088679
S = 92.556 (cal/mol·K)

2nd LDA segment

EE = -299.255316 (Hartree)
ZPE_{298} = -299.063155
E_{298} = -299.053042
H_{298} = -299.052098
G_{298} = -299.097396
S = 95.338 (cal/mol·K)

THF segment

EE = -232.371855 (Hartree)
ZPE_{298} = -232.254823
E_{298} = -232.249900
H_{298} = -232.248956
G_{298} = -232.282876
S = 71.391 (cal/mol·K)

N-Oxide segment

EE = -976.444344 (Hartree)
ZPE_{298} = -975.887445
E_{298} = -975.859601
H_{298} = -975.858657
G_{298} = -975.944242
S = 180.130 (cal/mol·K)

Frequency Calculations for the Energy Decomposition on Ground Structure 6a

1st LDA segment

EE = -299.250965 (Hartree)
ZPE_{298} = -299.058371
E_{298} = -299.048400
H_{298} = -299.047456
G_{298} = -299.092209
S = 94.190 (cal/mol·K)

2nd LDA segment

EE = -299.255000 (Hartree)
ZPE_{298} = -299.062439
E_{298} = -299.052557
H_{298} = -299.051613
G_{298} = -299.096116

S = 93.665 (cal/mol·K)

THF segment

EE = -232.371458 (Hartree)
ZPE_{298} = -232.254383
E_{298} = -232.250229
H_{298} = -232.249285
G_{298} = -232.281439
S = 67.673 (cal/mol·K)

N-Oxide segment

EE = -976.499723 (Hartree)
ZPE_{298} = -975.937796
E_{298} = -975.910297
H_{298} = -975.909353
G_{298} = -975.994784
S = 179.806 (cal/mol·K)

Frequency Calculations for the Energy Decomposition on 11a

1st LDA segment

EE = -299.248560 (Hartree)
ZPE_{298} = -299.056321
E_{298} = -299.045454
H_{298} = -299.044509
G_{298} = -299.091718
S = 99.359 (cal/mol·K)

2nd LDA segment

EE = -299.245566 (Hartree)
ZPE_{298} = -299.052585
E_{298} = -299.042089
H_{298} = -299.041144
G_{298} = -299.086850
S = 96.196 (cal/mol·K)

THF segment

EE = -232.372141 (Hartree)
ZPE_{298} = -232.254931

E_{298} = -232.250001
H_{298} = -232.249057
G_{298} = -232.283536
S = 72.567 (cal/mol·K)

N-Oxide segment

EE = -976.460484 (Hartree)
ZPE_{298} = -975.899742
E_{298} = -975.871488
H_{298} = -975.870544
G_{298} = -975.958327
S = 184.755 (cal/mol·K)

Frequency Calculations for the Energy Decomposition on 11b

1st LDA segment

EE = -299.246951 (Hartree)
ZPE_{298} = -299.054903
E_{298} = -299.044673
H_{298} = -299.043729
G_{298} = -299.089102
S = 95.495 (cal/mol·K)

2nd LDA segment

EE = -299.247045 (Hartree)
ZPE_{298} = -299.054533
E_{298} = -299.043784
H_{298} = -299.042840
G_{298} = -299.089165
S = 97.500 (cal/mol·K)

THF segment

EE = -232.372349 (Hartree)
ZPE_{298} = -232.255220
E_{298} = -232.250341
H_{298} = -232.249397
G_{298} = -232.283165
S = 71.070 (cal/mol·K)

N-Oxide segment

EE = -976.429223 (Hartree)
ZPE_{298} = -975.873627
E_{298} = -975.846018

H_{298} = -975.845074
G_{298} = -975.930661
S = 180.133 (cal/mol·K)

Frequency Calculations for the Energy Decomposition on Ground Structure 11a

1st LDA segment

EE = -299.249676 (Hartree)
ZPE_{298} = -299.056717
E_{298} = -299.046055
H_{298} = -299.045111
G_{298} = -299.091990
S = 98.666 (cal/mol·K)

2nd LDA segment

EE = -299.247771 (Hartree)
ZPE_{298} = -299.054991
E_{298} = -299.044327
H_{298} = -299.043383
G_{298} = -299.089541
S = 97.147 (cal/mol·K)

THF segment

EE = -232.372334 (Hartree)
ZPE_{298} = -232.255212
E_{298} = -232.250335
H_{298} = -232.249391
G_{298} = -232.283145
S = 71.042 (cal/mol·K)

N-Oxide segment

EE = -976.494137 (Hartree)
ZPE_{298} = -975.930890
E_{298} = -975.903237
H_{298} = -975.902293
G_{298} = -975.987873
S = 180.118 (cal/mol·K)

NBO Analyses on Solvent Aggregates

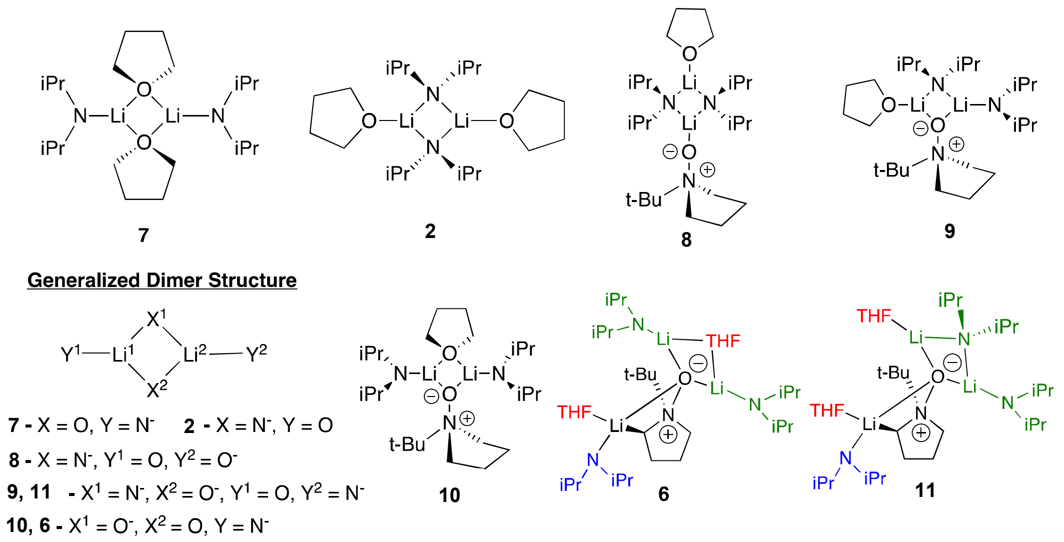

Figure S43. Ground structures that were analyzed with NBO and a generalized structure for report the geometric and second perturbation calculations.

NBO Analysis on LDA/THF Dimers 2 and 7

Table S11. Data on the structure and second perturbation analysis of energy exchange between NBO of structures **2** and **7**, using the generalized structure depicted in Figure S43. Where the energy exchanged by orbitals of the bridging atoms (X^1 and X^2) into the lithiums is summed in the first section for each structure, and the energy exchanged by the orbitals of the non bridging atoms (Y^1 and Y^2) is summed in the second section for each structure.

Ionic Bond	Structure **2**		Structure **7**	
	Distance (Å)	Energy Exchanged (kcal/mol)	Distance (Å)	Energy Exchanged (kcal/mol)
		**Bridging Atoms**		
**X^1 --- Li^1**	1.95	9.88	2.02	4.22
**X^2 --- Li^1**	1.95	9.04	2.00	4.56
**X^1 --- Li^2**	2.00	8.18	2.01	7.47
**X^2 --- Li^2**	1.99	8.03	2.03	6.24
**Total**		35.1		22.5
		**Non Bridging Atoms**		
**Y^1 --- Li^1**	1.92	9.00	1.86	8.95
**Y^2 --- Li^2**	1.93	7.77	1.87	11.5
**Total**		16.8		20.5

NBO Analysis on LDA/THF/*N*-Oxide 8, 9, and 10

Table S12. Data on the structure and second perturbation analysis of energy exchange between NBO of structures **8**, **9**, and **10**; using the generalized structure depicted in Figure S43. Where the energy exchanged by orbitals of the bridging atoms (X^1 and X^2) into the lithiums is summed in the first section for each structure, and the energy exchanged by the orbitals of the non bridging atoms (Y^1 and Y^2) is summed in the second section for each structure.

Ionic Bond	Structure **8**		Structure **9**		Structure **10**	
	Distance (Å)	Energy Exchanged (kcal/mol)	Distance (Å)	Energy Exchanged (kcal/mol)	Distance (Å)	Energy Exchanged (kcal/mol)
			**Bridging Atoms**			
**X^1 --- Li^1**	1.93	14.1	1.94	4.07	1.87	6.43
**X^2 --- Li^1**	2.01	10.2	1.84	4.26	2.05	4.12
**X^1 --- Li^2**	2.04	4.32	2.04	6.94	1.88	5.90
**X^2 --- Li^2**	2.01	4.83	1.99	8.11	2.01	3.59
**Total**		33.5		23.4		20.0
			**Non Bridging Atoms**			
**Y^1 --- Li^1**	1.94	1.20	1.93	3.38	1.87	4.81
**Y^2 --- Li^2**	1.86	7.12	1.95	5.06	1.86	5.61
**Total**		8.32		8.44		10.4

NBO Analysis on key Ground Structures 6 and 11

Table S13. Data on the structure and second perturbation analysis of energy exchange between NBO of structures **6** and **11**; using the generalized structure Figure S43. Where the energy exchanged by orbitals of the bridging atoms (X^1 and X^2) into the lithiums is summed in the first section for each structure, and the energy exchanged by the orbitals of the non bridging atoms (Y^1 and Y^2) is summed in the second section for each structure.

Ionic Bond	Structure **6**		Structure **11**	
	Distance (Å)	Energy Exchanged (kcal/mol)	Distance (Å)	Energy Exchanged (kcal/mol)
		**Bridging Atoms**		
**X^1 --- Li^1**	1.96	8.23	1.97	5.17
**X^2 --- Li^1**	2.08	4.52	1.95	7.33
**X^1 --- Li^2**	1.97	5.62	2.07	5.17
**X^2 --- Li^2**	2.01	1.93	1.96	9.56
**Total**		20.3		27.2
		**Non Bridging Atoms**		
**Y^1 --- Li^1**	1.91	3.60	2.08	2.84
**Y^2 --- Li^2**	1.90	2.18	1.93	4.49
**Total**		5.78		7.33

Aggregation Effects on First Deprotonation

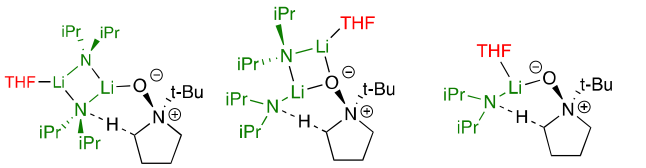

Figure S44. Three different LDA / THF / *N*-oxide aggregate transition structures for the first deprotonation of *tert*-butyl pyrrolidine *N*-oxide and their resulting activation enthalpy in kcal/mol.

Similar to the results generated for the second deprotonation transition, when the solvent aggregation of our model system reflects a mixed LDA / *N*-oxide dimer, then activation energy is decreased. For the first deprotonation moving to a mixed LDA dimer aggregate **S2** lowered the activation energy for the transition by 2.3 kcal/mol as compared to non-aggregate **S3**. While this represents a significant decrease, it is not as important to our conclusions as the results for the second deprotonation as discussed in the paper. This is because this first deprotonation is not the rate limiting transition nor does it have any alternative mechanisms pathways. Additionally, we calculated the first deprotonation activation energy when the aggerate has both nitrogens bridging as is reported in the literature (**S1**). As mentioned in the paper, this solvent structure is seen as non-reactive due to both lone pairs of LDA being involved in the dimer and would require decomplexation to deprotonate the *alpha*-hydrogen. In support of this, a transition structure was found but at a significantly higher barrier than either of the other first deprotonation transitions. This is due to the fact that dimer in **S1** has to separate to free up at least one lone pair causing an increase in distortion energy within the transition structure. Further geometric and thermodynamic details are available on all the structures below.

Confirmation of Activation Energy with Higher Order Theory

Calculations at higher levels of theory for the first deprotonation structures, both anti-planar (**S8**) and syn-planar (**S9**) orientation, were run to confirm the previous calculation performed at M06-2X/jul-cc-pVDZ level of theory. DFT calculations were done at M06-2X/aug-cc-pVDZ and M06-2X/jul-cc-pVTZ. Additionally, second-order Møller-Plesset calculations were done at MP2/jul-cc-pVDZ level of theory. Across these different calculations there was not large deviations in the activation energies calculated with each transition structure. Utilizing MP2 lowered both activation enthalpies by approximately 1 kcal/mol. When examining the difference in activation enthalpies between two orientations across the different levels of theory, the trend of approximately 7.5 kcal/mol difference holds. Therefore, calculations done at M06-2X/jul-cc-pVDZ level of theory represent good approximations for activation enthalpies and relative enthalpic differences.

Table S14. Activation enthalpies (ΔH) in kcal/mol for anti-planar (**S8**) and syn-planar (**S9**) first deprotonation at different levels of theory, and the difference in activation enthalpies between the two orientations.

Level of Theory	S8	S9	ΔE_a
M06-2X/jul-cc-pVDZ	20.0	12.5	7.5
M06-2X/jul-cc-pVTZ	22.0	14.2	7.8
M06-2X/aug-cc-pVDZ	20.2	12.7	7.5
MP2/jul-cc-pVDZ	18.5	11.6	6.9

Procedures for the synthesis of 7-Azanorbornane S45

Procedure using a 1:1 ratio of *N*-oxide to *trans*-stilbene:

Tert-butyl N-oxo pyrrolidine (0.4 mL, 0.2 mmol, 1 equiv.) was transferred from a 0.5M stock solution in DCM to a test tube and dried under vacuum. Trans-stilbene (38 mg, 0.2 mmol, 1 equiv.) was then added to the test tube and after purging, the reagents were dissolved in dry THF (2 mL, 0.1 M). The reaction was cooled to -78 °C with a dry ice/acetone bath and 0.88 M LDA (0.68 mL, 0.6 mmol, 3 equiv.) was added dropwise. The reaction was allowed to warm slowly to RT over the course of 18 h. The reaction was quenched with saturated ammonium chloride solution, extracted with EtOAc (3 x's), dried over MgSO4, and concentrated. The residue was purified by FCC. Solvent conditions: 6:1 hexanes/EtOAc, Rf = 0.53 (56 % yield, 34 mg, 0.11 mmol). ^{1}H NMR (400 MHz, Chloroform-d) δ 7.62 – 7.50 (m, 2H), 7.33 – 7.20 (m, 6H), 7.20 – 7.10 (m, 2H), 3.96 – 3.88 (m, 1H), 3.61 (dt, J = 4.5, 1.1 Hz, 1H), 3.39 (t, J = 5.2 Hz, 1H), 2.74 (d, J = 6.1 Hz, 1H), 1.74 (dd, J = 12.1, 4.8 Hz, 1H), 1.57 – 1.42 (m, 3H), 1.19 (s, 9H). ^{13}C NMR (101 MHz, Chloroform-d) δ 147.88, 141.66, 128.42, 128.26, 128.14, 127.73, 126.01, 125.97, 64.14, 60.91, 59.42, 54.37, 51.78, 32.11, 30.35, 24.35. IR: 3059, 3024, 2968, 2922, 2867, 1713, 1602, 1493, 1449, 1387, 1230, 1175, 1060, 1029, 751, 739, 697, 590, 530 cm-1. HRMS (ESI) m/z: $[M+H]^+$ Calc'd for $C_{22}H_{28}N^+$ 306.2216; Found 306.2263.

Procedure using a 1:0.5 ratio of *N*-oxide to *trans*-stilbene:

Tert-butyl N-oxo pyrrolidine (0.143 g, 1 mmol, 1 equiv.) was weighed out and transferred to a flame dried 25 mL round bottom flask. The flask was then sealed with a rubber septum and flushed with nitrogen gas. Dry THF (2.5 mL) was then added via a syringe and the mixture was stirred until the N-oxide was completely dissolved. Once dissolved the reaction mixture was cooled to -78 °C before adding 1.51M LDA (2 mL, 3.0 mmol, 3 equiv.) dropwise. In a separate flask, trans-stilbene (0.0895 g, 0.5 mmol, 0.5 equiv.) was dissolved in dry THF (2.5 mL) and purged in a nitrogen environment. Once complete, the trans-stilbene solution was added to the mixture of N-oxide via a syringe dropwise. The reaction mixture was allowed to warm to room temperature and the mixture was stirred for three hours. The reaction was then quenched with a saturated ammonium chloride solution and then extracted with diethyl ether, then dried with Na2SO4, and concentrated. The resulting oil was then purified by FCC. Solvent conditions 4:1 hexanes/EtOAc, Rf = 0.73 (44 % yield, 67.7 mg, 0.22 mmol)

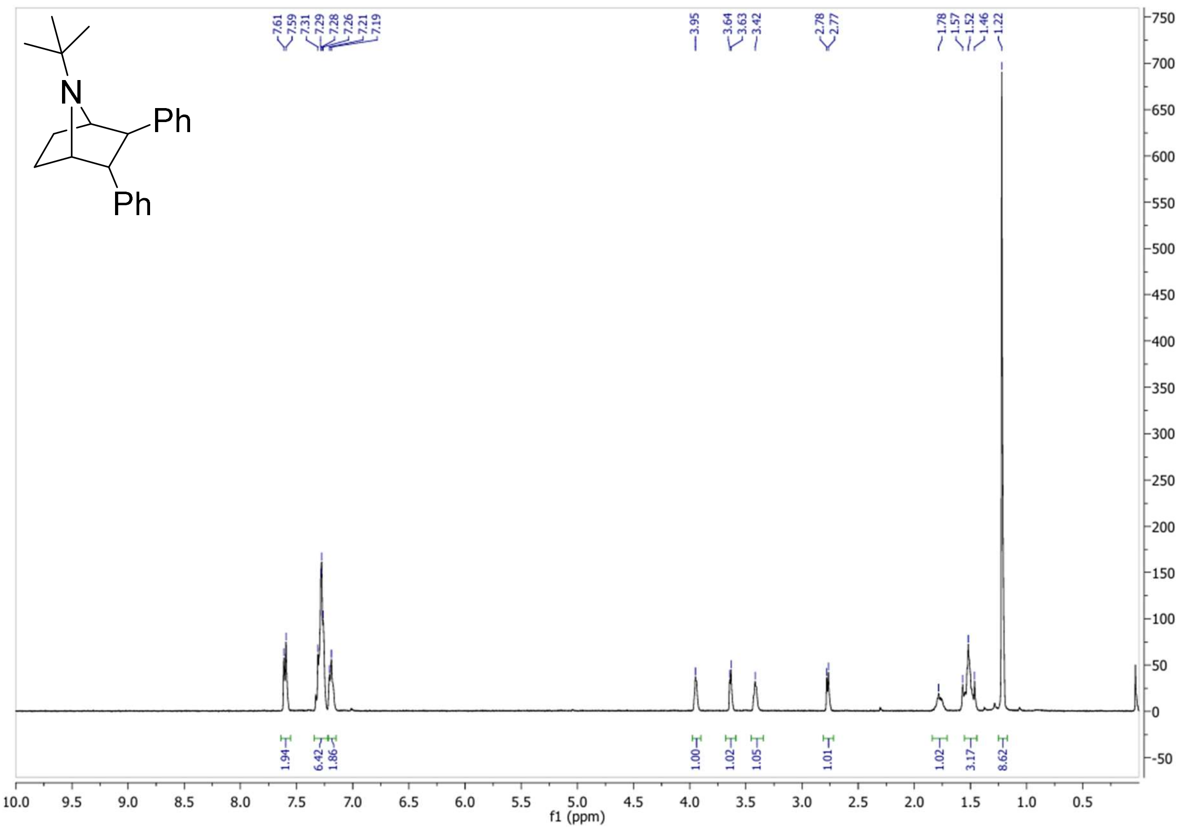

Figure S45. ^{1}H-NMR of : (1R,3R,4S)-7-(tert-butyl)-2,3-diphenyl-7-azabicyclo[2.2.1]heptane

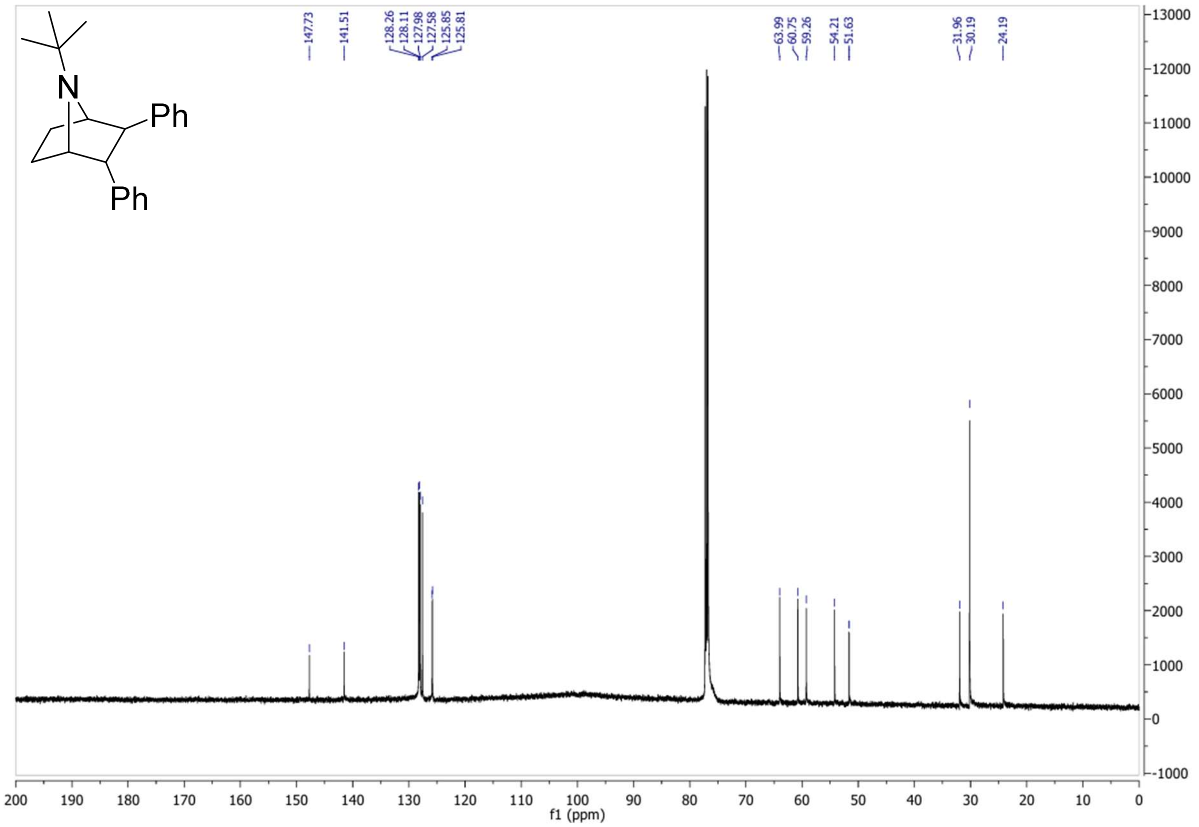

Figure S46. ^{13}C-NMR of : (1R,3R,4S)-7-(tert-butyl)-2,3-diphenyl-7-azabicyclo[2.2.1]heptane

Pertinant Free Energy Diagrams

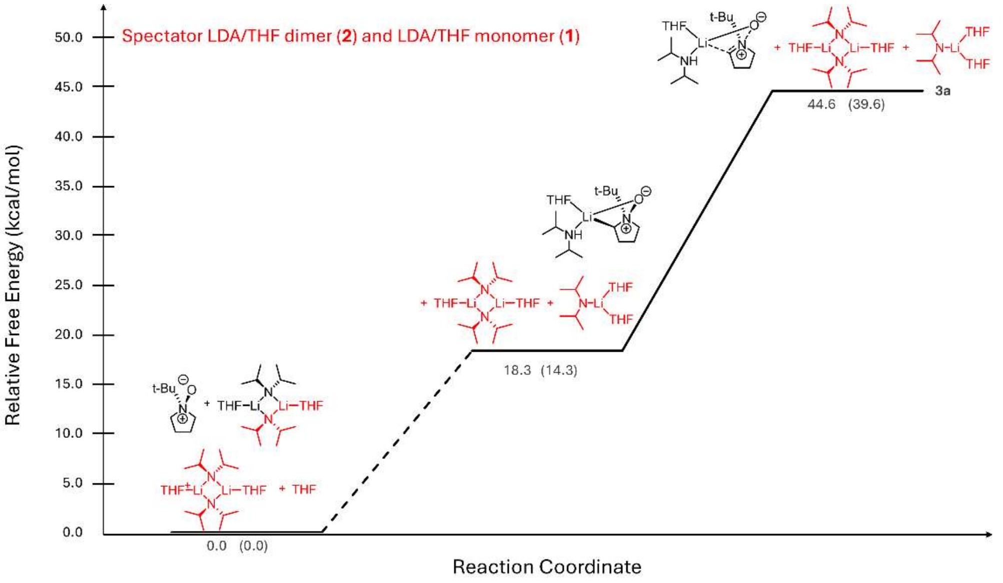

Figure S47a. Relative energy diagram for **3a** relative to the common ground state. The first value in the pair of numbers below each structure displays the relative corrected free energies (kcal/mol), and the second value in paranthesis, gives the relative enthalpies (kcal/mol). The dashed line indicates intermediate steps in the mechanism.

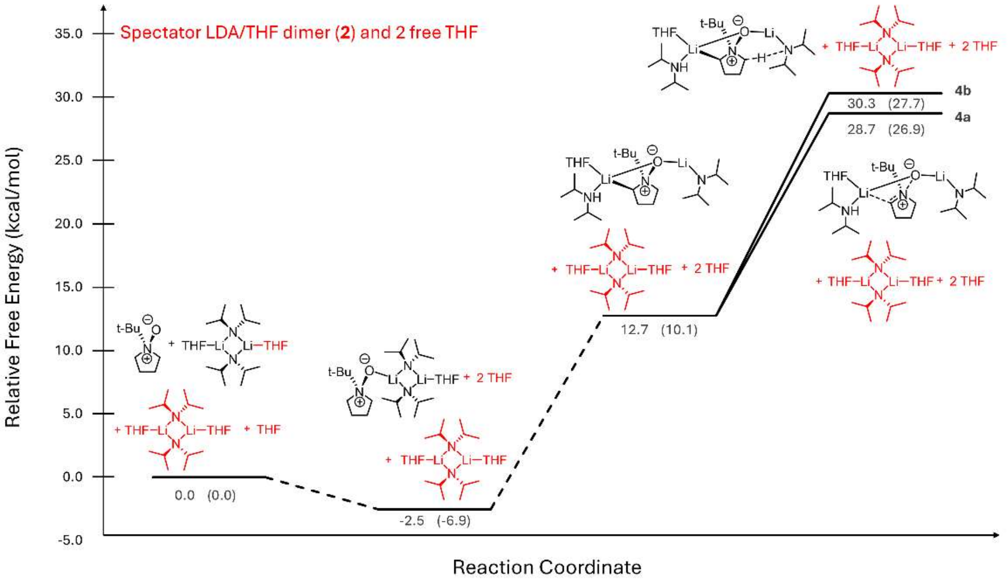

Figure S47b. Relative energy diagram for **4a**/**4b** relative to the common ground state. The first value in the pair of numbers below each structure displays the relative corrected free

energies (kcal/mol), and the second value in paranthesis, gives the relative enthalpies (kcal/mol). The dashed line indicates intermediate steps in the mechanism.

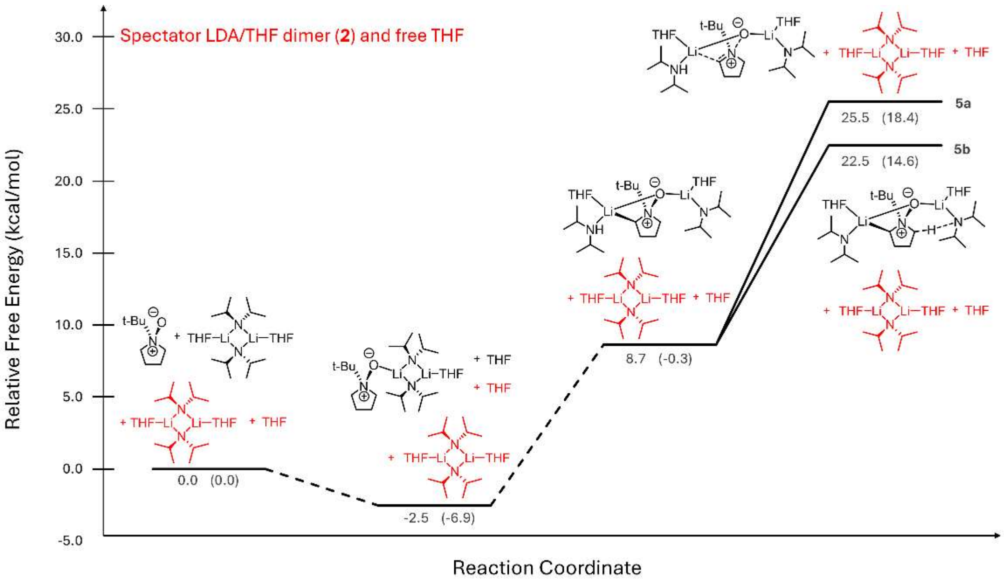

Figure S47c. Relative energy diagram for **5a**/**5b** relative to the common ground state. The first value in the pair of numbers below each structure displays the relative corrected free energies (kcal/mol), and the second value in paranthesis, gives the relative enthalpies (kcal/mol). The dashed line indicates intermediate steps in the mechanism.

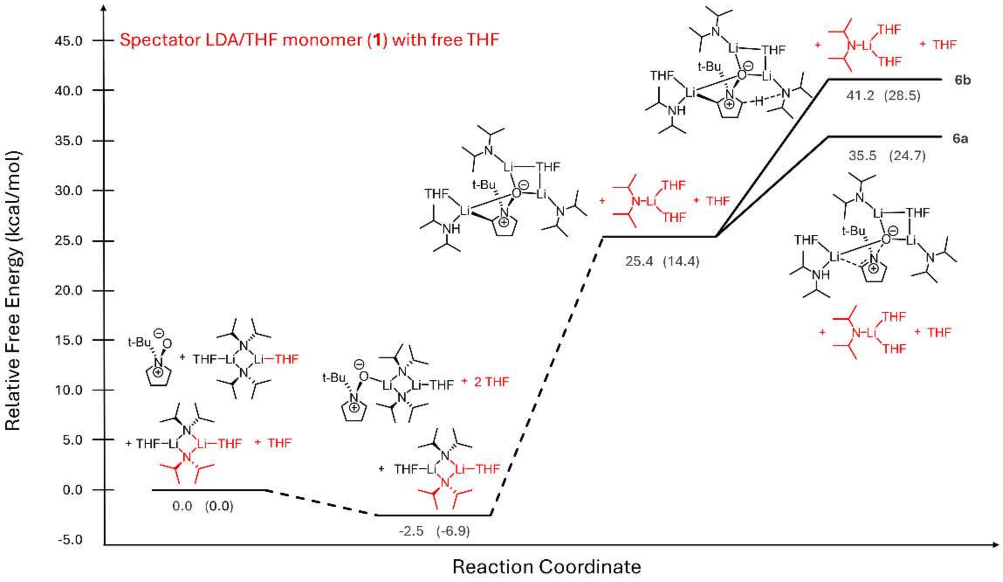

Figure S47d. Relative energy diagram for **6a**/**6b** relative to the common ground state. The first value in the pair of numbers below each structure displays the relative corrected free

energies (kcal/mol), and the second value in paranthesis, gives the relative enthalpies (kcal/mol). The dashed line indicates intermediate steps in the mechanism.

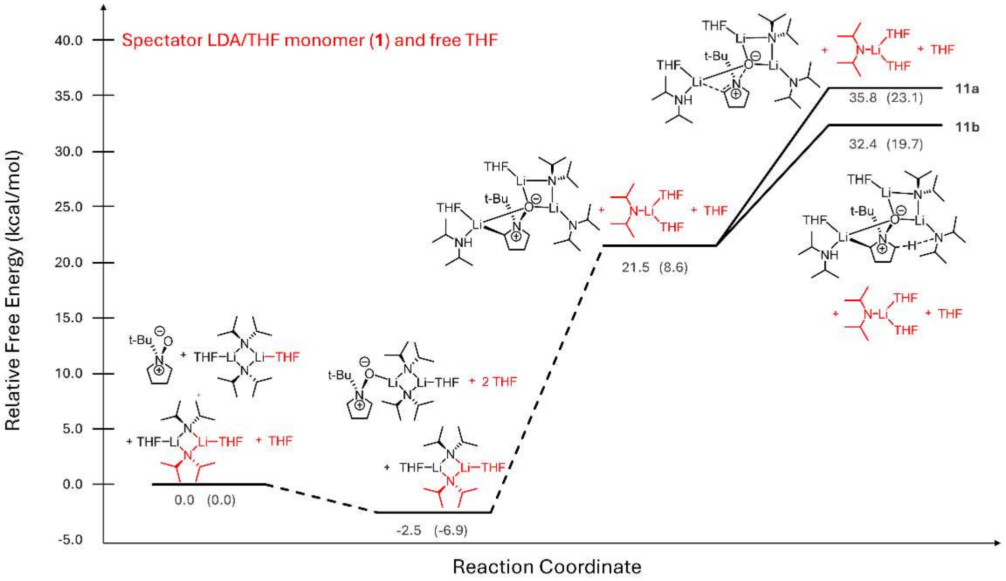

Figure S47e. Relative energy diagram for **11a/11b** relative to the common ground state. The first value in the pair of numbers below each structure displays the relative corrected free energies (kcal/mol), and the second value in paranthesis, gives the relative enthalpies (kcal/mol). The dashed line indicates intermediate steps in the mechanism.
